# Systems analysis identifies melanoma-enriched pro-oncogenic networks controlled by the RNA binding protein CELF1

**DOI:** 10.1038/s41467-017-02353-y

**Published:** 2017-12-21

**Authors:** Metehan Cifdaloz, Lisa Osterloh, Osvaldo Graña, Erica Riveiro-Falkenbach, Pilar Ximénez-Embún, Javier Muñoz, Cristina Tejedo, Tonantzin G. Calvo, Panagiotis Karras, David Olmeda, Belén Miñana, Gonzalo Gómez-López, Estela Cañon, Eduardo Eyras, Haihong Guo, Ferdinand Kappes, Pablo L. Ortiz-Romero, Jose L. Rodríguez-Peralto, Diego Megías, Juan Valcárcel, María S. Soengas

**Affiliations:** 1 Melanoma Laboratory, Molecular Oncology Programme, Spanish National Cancer Research Center (CNIO), 28029 Madrid, Spain; 2 Bioinformatics Unit, CNIO, 28029 Madrid, Spain; 3 Instituto de Investigación i+12, Hospital 12 de Octubre Medical School, Universidad Complutense, 28041 Madrid, Spain; 4 Proteomics Core Unit, CNIO, Madrid, Spain; 5 Centre de Regulació Genòmica (CRG), The Barcelona Institute of Science and Technology, Barcelona, 08003 Spain; 6 Department of Experimental and Health Sciences, Universidad Pompeu Fabra, Barcelona, 08002 Spain; 7 Institució Catalana de Recerca i Estudis Avançats (ICREA), Barcelona, 08010 Spain; 8 Institute of Biochemistry and Molecular Biology; Medical School, RWTH Aachen University, Aachen, 52074 Germany; 9 Department of Biological Sciences, Xi’an Jiaotong-Liverpool University, No. 111, Ren Ai Road, Dushu Lake Higher Education Town, Suzhou Industrial Park (SIP), Suzhou, 215123 China; 10 Confocal Microscopy Unit, (CNIO), Madrid, 28029 Spain

## Abstract

Melanomas are well-known for their altered mRNA expression profiles. Yet, the specific contribution of mRNA binding proteins (mRBPs) to melanoma development remains unclear. Here we identify a cluster of melanoma-enriched genes under the control of CUGBP Elav-like family member 1 (CELF1). CELF1 was discovered with a distinct prognostic value in melanoma after mining the genomic landscape of the 692 known mRBPs across different cancer types. Genome-wide transcriptomic, proteomic, and RNA-immunoprecipitation studies, together with loss-of-function analyses in cell lines, and histopathological evaluation in clinical biopsies, revealed an intricate repertoire of CELF1-RNA interactors with minimal overlap with other malignancies. This systems approach uncovered the oncogene DEK as an unexpected target and downstream effector of CELF1. Importantly, CELF1 and DEK were found to represent early-induced melanoma genes and adverse indicators of overall patient survival. These results underscore novel roles of CELF1 in melanoma, illustrating tumor type-restricted functions of RBPs in cancer.

## Abstract (web summary)

Hundreds of mRNA binding proteins (mRBPs) have been described in mammalian cells. Here, the authors identify RBPs differentially regulated in melanoma, and show the RBP CELF1 controlling a distinct set of protumorigenic factors.

## Introduction

RNA binding proteins (RBPs) have long raised attention in the oncology field for their potential to modulate the stability, localization and/or alternative splicing of transcripts coding for virtually all known oncogenes and tumor suppressors^1,2^. Moreover, large-scale genomic and transcriptomic analyses have identified a broad spectrum of mutations, copy number variations and mRNA expression changes in multiple RBPs across a variety of tumor types, ranging from glioblastoma to breast, colon, kidney, lung, prostate or thyroid carcinomas^3,4^. Nevertheless, the assignment of individual RBPs to specific roles in malignant transformation remains a daunting challenge. A recent census in human cells has reported over 1500 RBPs, with 692 mRNA binding proteins (mRBPs)^5^, most of which have yet to be functionally characterized. Consequently, comprehensive networks and functional annotation of downstream targets of RBPs in cancer are particularly scarce.

A disease where RBPs have the potential to drive malignancy is cutaneous melanoma. These lesions are characterized by the largest mutational rate described to date^6,7^, with the potential to impinge on multiple RNA regulators, particularly in the context of alternative splicing^8^. Moreover, melanomas are characterized by extensive changes in mRNA expression profiles^9,10^. However, mechanistic information on the specific contribution of RBPs to melanoma initiation and progression is rather limited. With respect to mRNA splicing modulators, ESRP1, PTBP1, and U2AF2 have been linked to altered exon usage in the pro-invasive glycoprotein CD44^11–13^. Other pro-tumorigenic events have been related to changes in exon inclusion/exclusion, mediated by SRSF3 on *MDM4* ^14^. MAGOH, SNRPE, SNRPD1, or USP39 are additional regulators of spliceosome functions required for the survival of melanoma cells, although their specific mode of action has yet to be defined^15–17^.

Expression studies in clinical biopsies, and comprehensive transcriptomic and proteomic analyses of RBP-dependent functions become more important in the light of a broad spectrum of synonymous mutations in melanoma cells, not only at intergenic sites, but at untranslated (UTR) regions of mRNAs^7,18^. Of those, the least understood are 3′ UTR-related events. We have recently identified two 3′ end-interacting factors (the cytoplasmic polyadenylation protein CPEB4, and the translation modulator UNR) with key roles in melanoma progression^19,20^. Nevertheless, genome-wide analyses of the genomic landscape of RBPs in melanoma are still pending.

Here, we mined clinical data sets for an unbiased characterization of the genomic status (mutations, amplifications, deletions, translocations) of all known mRBPs in human melanomas. These studies failed to reveal characteristic copy number changes reported in less genetically altered cancers. Instead, comparative genome-wide RNA sequencing and RBP-focused arrays revealed a subset of RBPs subject to post-transcriptional deregulation in melanoma cells. CELF1 was identified as a top-scoring factor among RBPs with no previous links to melanoma. Proteomic, transcriptomic, histological and functional analyses, together with studies of patient prognosis revealed new roles and melanoma-enriched targets of CELF1, which provide insight on selective RBPs fueling tumor development.

## Results

### Distinct landscape of RPBs in melanoma cells

**Fig. 1:**
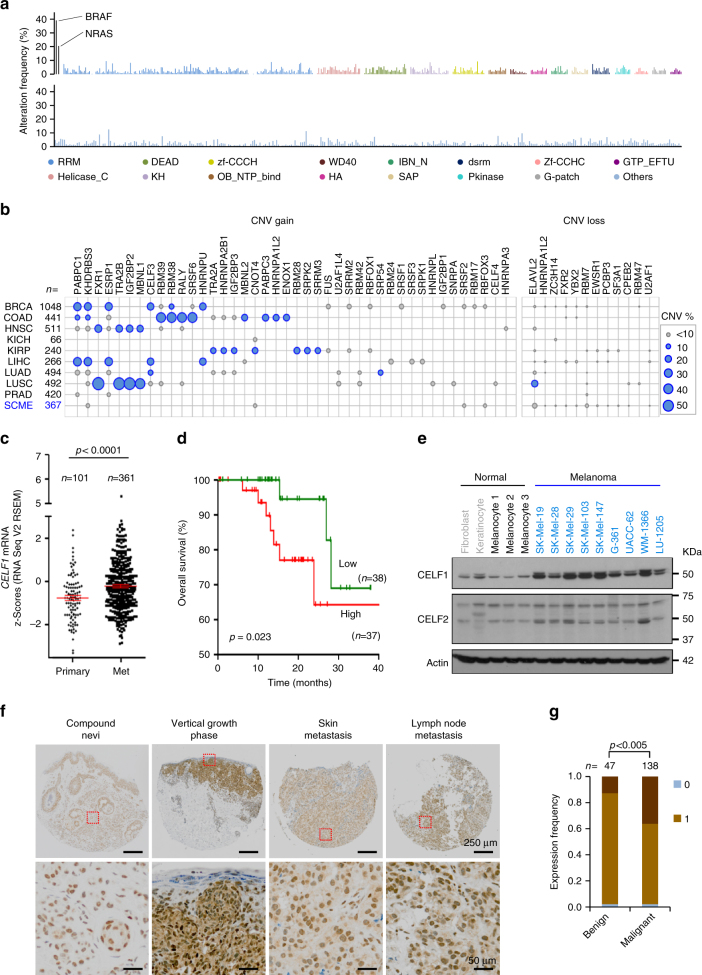
CELF1 overexpression in melanoma within a distinct landscape of RBPs. **a** Frequency (%) of melanoma patients with mutations and/or copy number alterations (deletions or amplifications) in all known mRBPs (*n* = 692), classified according to their RNA binding domains (Pfam nomenclature). Data were retrieved from the melanoma TCGA database using cBioPortal. *BRAF* and* NRAS* are included as references for classical melanoma-associated oncogenes (see Supplementary Table 1 for additional information). **b** Copy-number variation (CNV), gain and loss of the indicated RBPs in different tumor types showed on a bubble chart: BRCA breast invasive carcinoma, COAD colon adenocarcinoma, HNSC head and neck squamous cell carcinoma, KICH kidney chromophobe, KIRP kidney renal papillary carcinoma, LIHC liver hepatocellular carcinoma, LUAD lung adenocarcinoma, LUSC lung squamous cell carcinoma, PRAD prostate adenocarcinoma, SCME skin cutaneous melanoma. Circle size represents percentage of biopsies with CNV alterations. **c** *CELF1* mRNA expression levels in TCGA melanoma samples categorized by disease stage. **d** Overall survival of melanoma patients separated as a function of high vs. low *CELF1* mRNA (these considered with respect to the median expression of all data in the TCGA melanoma data set). Indicated are *p*-values estimated with the Gehan–Breslow–Wilcoxon test; *p*-(Log-rank test)=0.048. **e** Immunoblots illustrating CELF1 expression in normal skin cells (fibroblasts, keratinocytes and three independent pools of melanocytes) and the indicated melanoma cell lines. CELF2 was indicated as a reference for homolog ELAV-family member. **f** Representative micrographs of benign nevi, primary melanomas (vertical growth phase), and metastatic melanomas, showing a heightened CELF1 expression (brown staining) during tumor progression. Nuclei were counterstained in blue with hematoxilin. **g** Distribution of benign and malignant specimens (% of analyzed cases) separated as a function of CELF1 scoring, and pooled for vertical growth phase melanoma, skin metastases and lymph node metastases

We have recently reported a broad spectrum of mutations and copy number variations (CNVs) of RBPs in a series of non-melanoma solid tumors^3^. To interrogate whether these alterations also apply to malignant melanoma, the 692 known human mRBPs^5^ were mined throughout The Cancer Genome Atlas (TCGA), which includes comprehensive genomic and expression information from 479 human cutaneous melanomas^9^. Intriguingly, even if pooling mutations, genomic amplifications and deletions, the frequency of genomic alterations per mRBP was rather low, with an average of 2.4% affected patients per gene (Fig. 1a; see Supplementary Data 1 for detail). This is contrast, for example, to over 50% of CNVs in splicing factors for example in lung or colon carcinomas (Fig. 1b).

RNA sequencing (RNA-Seq) was then set to assess gene expression differences in normal skin melanocytes and the well-characterized UACC-62, SK-Mel-147, and SK-Mel-28 cell lines, representative of metastatic melanomas with prototypical mutations in *BRAF*, *NRAS*, and *p53* respectively^21^ (Supplementary Table 1). Reads (over 34 million per sample) were aligned to the human genome (Ref Seq GRCh37/hg19) for analysis of differential expression. Protein Analysis THrough Evolutionary Relationships (PANTHER)^22^ was used for the identification of biological functions specifically enriched in all melanoma cell lines (Bonferroni corrected binomial test *p*<0.05; see Supplementary Fig. 1a for a schematic flow chart of the procedures followed). Focusing on RBPs, 22% of these family members (348 out of 1542) were found to be significantly deregulated in melanoma cells, with a particular enrichment for factors controlling rRNA metabolism, RNA splicing, and mRNA processing (Supplementary Fig. 1b, c).

Curiously, out of the 348 RBPs altered in melanoma, only seven (CSTF2, DDX3X, DKC1, EIF1AX, GNL3L, MEX3C, and RBMX) were included in a RBP-cancer signature recently described in 16 non-melanoma tumor types^4^ (Supplementary Table 2). Analyses through the TCGA melanoma data set supported an increased mRNA expression of *DDX3X*, *EIF1AC*, *GNL3L*, and* RBMX* in skin or lymph node metastases, but with no significant correlation with overall patient survival (Supplementary Table 2). Therefore, the results above support a distinct expression of RBPs in melanoma cells.

### CELF1 is an early-induced RBP in melanoma cells and biopsies

To narrow down the RBPs for in-depth characterization in melanoma cells, additional transcriptomic analyses were performed on a high coverage array customized to monitor mRBPs with key roles in splicing and translational control, as well as factors known to recognize snRNAs, snoRNA, ncRNA, and tRNA (180 genes in total)^23^. The array also included 302 additional cancer-associated genes with essential functions in survival, proliferation, adhesion, and apoptosis^23^. RNA was isolated from SK-Mel-19 and SK-Mel-103 (as representative examples for BRAF- and NRAS-mutated melanoma cell lines^21^), for comparative studies with respect to pools of normal skin melanocytes (Supplementary Fig. 1a). Gene Ontology (GO) functional term enrichment identified 23 GO gene clusters (*p*-value corrected for multiple testing <0.05) related to RNA splicing and spliceosome formation, ribonucleoprotein complex assembly, mRNA 3′-end processing and modulation of RNA transcription (Fig. 1d; Supplementary Data 2).

The gene expression analyses described above reduced the set of RBPs upregulated in melanoma to 50 genes (Supplementary Fig. 2a). These included *MAGOH*, *PTBP1*, *SNRPD1*, *SRSF3*, and *SNRPE*, validating previous results^14,15,17^. A 4-point filter was then applied to select candidates for functional studies: (i) no previous links to malignant melanoma; (ii) potential to impact on the GO clusters we also found enriched in the cancer-associated factors analyzed (Supplementary Fig. 1e); (iii) broad roles on mRNA metabolism, particularly on mRNA stability, translational regulation and/or alternative splicing, and (iv) physiological significance. As summarized in Supplementary Fig. 2a, b, scoring through these filters were *CELF1* (Elav-like family member 1), *KHDRBS1* (KH RNA Binding Domain Containing, Signal Transduction Associated 1), and *FUBP1* (Far Upstream Element Binding Protein 1). mRNA upregulation of these three genes was also confirmed in melanoma-TCGA database (see for *CELF1* in Fig. 1c, and for *KHDRBS1* and *FUBP1* in Supplementary Fig. 3a, b, respectively). However, only high *CELF1* mRNA expression significantly correlated with poor prognosis of primary melanoma patients in this set (Fig. 1d; Supplementary Fig. 3c, d). CELF1, also referred to as CUGBP1 for its characteristic binding to GU-rich elements (GREs)^24^, was interesting for its well-known roles in the control of alternative splicing^25,26^. Moreover, CELF1 has been found upregulated in multiple cancer types^27–32^. Integrated transcriptomic and proteomic analyses have yet to be performed in these tumors, but RNA immunopreciptation analyses^33,34^ support a key role of CELF1 favoring mRNA decay, particularly of apoptotic factors and other potential suppressive signals^24^.

To further define the rationale for the selection of CELF1 as a novel pro-tumorigenic RBP in melanoma, we set to assess whether its upregulation was specific or reflected global amplifications of the chromosomal locus where this gene maps. As summarized in Supplementary Fig 4a, b the allelic status of *CELF1* was largely similar to 13 flanking genes at chromosome 11p11.2 band, as defined by exploring TCGA melanomas. However, significant positive correlations to *CELF1* mRNA (*r* > 0.45) were only identified for kelch repeat and BTB domain containing four (*KBTBD4)*, but the mRNA levels of this factor were not found to separate patients with good vs. poor prognosis (Supplementary Fig. 4c–e). Other immediate *CELF1* neighbors (e.g., *RAPSN*) even had an opposing prognostic trend (Supplementary Fig. 4c–e). These data indicate that *CELF1* mRNA overexpression is largely uncoupled from its neighboring genes, supporting a distinct active selection during melanoma development.

Immunoblot analyses confirmed the upregulation of CELF1 in melanoma cells also at the protein level (see comparative data to primary melanocytes, as well as skin fibroblasts and keratinocytes in Fig. 1e). This was not the case for other CELF1 homologs such as CELF2 (Fig. 1e), supporting specificity in CUG-binding factors. Further analyses of the melanoma TCGA specimens stratified for tumor stage suggested an early induction of *CELF1* mRNA during melanoma progression (Supplementary Fig. 5a). Consistent with these data, normal melanocytes had a significantly lower CELF1 protein expression than Mel-STV immortalized melanocytes, classical surrogates for early transformation events in this disease^35^ (Supplementary Fig. 5b). Moreover, a retrospective series of benign nevi (*n* = 47) and clinically annotated primary and metastatic melanoma biopsies (*n* = 138) confirmed a significant accumulation of CELF1 in malignant lesions, particularly in vertical growth phase cutaneous melanomas (two-tailed Student’s *t* test *p* < 0.005; Fig. 1f, g; see scoring system in Supplementary Fig. 5c). Together, these results identify CELF1 as an early-induced RBP during melanoma progression and as a putative adverse indicator of patient prognosis.

### Defective proliferation of CELF1-depleted melanoma cells

**Fig. 2:**
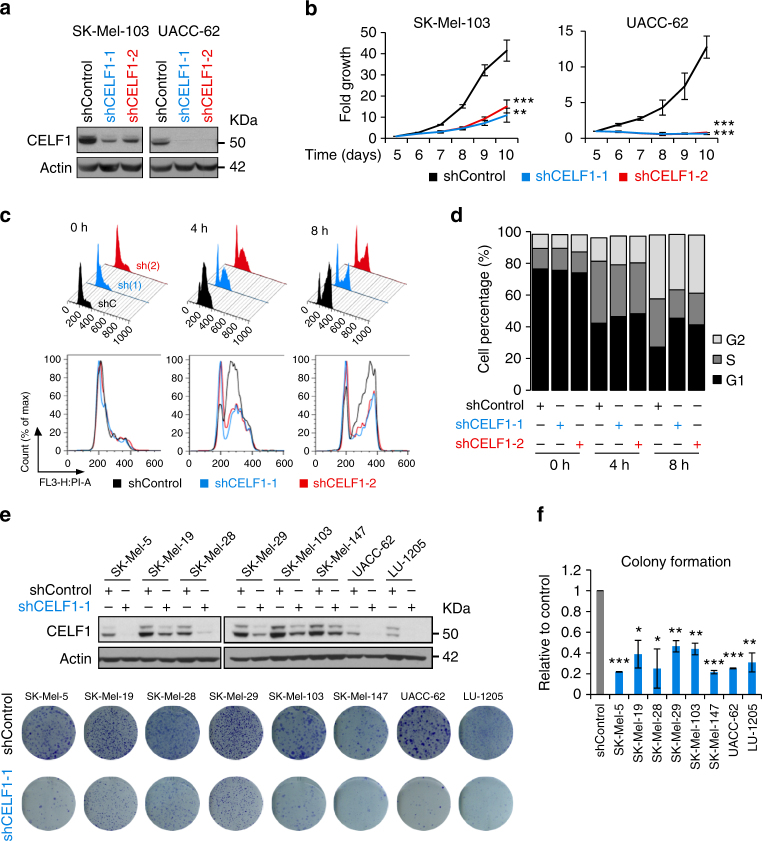
Cell cycle delay after CELF1 depletion in melanoma cells. **a** Efficiency of lentiviral-based transfer of CELF1 shRNAs (shCELF1-1 and 2) in the indicated cell lines visualized by immunoblotting with respect to control shRNA. **b** Impaired growth of SK-Mel-103 and UACC-62 cells upon CELF1 depletion. Time points represent days after lentiviral infection. Error bars correspond to SEM of three independent experiments in triplicate. **c** Cell cycle profiles of shControl and shCELF1 transduced SK-Mel-103 cells at the indicated times after release from thymidine block. **d** Quantification of data in **c**. **e** Melanoma cells expressing shControl or shCELF1 (upper panel) analyzed for colony formation capability (crystal violet staining) 12 days after cell seeding. **f** Quantification of inhibited colony formation of cells as in **e**. Data are represented as the ratio of inhibition with respect to shControl transduced cells (for simplicity here shown as a gray bar only for SK-Mel-5). Error bars correspond to SEM of two independent experiments in duplicates

Depletion of CELF1 with validated shRNAs^36^ (Fig. 2a) resulted in a markedly reduced cell proliferation (see for SK-Mel-103 or UACC-62 in Fig. 2b). G1/S-arrested cells (by a standard double thymidine block) had also a compromised ability to regain proliferation in the absence of CELF1 (Fig. 2c, d). This reduced proliferation was also evident in long-term colony assays performed in various melanoma cell lines after CELF1 depletion by shRNA (Fig. 2e, f) or by siRNA (Supplementary Fig. 5d, e). No significant cell death was observed even at late times after CELF1 depletion, in contrast to HeLa or cells from laryngeal or oral squamous cell carcinoma, where CELF1 was found to control pro-apoptotic genes^31,33,37^.

### Known CELF1 targets are not shared by melanoma cell lines

Characteristic intron inclusion/exclusion events modulated by CELF1 (i.e., involving *INSR*, *SERCA1*, *FXR1*, *FAM188A*, *ANK2*, *ACTN1*, *MEF2A*, and *PPFIBP1)* ^38^ were addressed by quantitative reverse transcription-PCR (qRT-PCR) in eight independent melanoma cell lines. However, as summarized in Supplementary Fig. 6a–c no obvious changes in the splicing expression was found in melanoma cells following CELF1 depletion.

Next, published genome-wide analyses of CELF1-bound transcripts were mined to assess additional targets of this protein which we could use as a guideline in melanoma. RNA immunoprecipitation-sequencing (RIP-Seq) was found for two studies in HeLa^33,34^ and an additional report in T cells^39^, that identified 1439, 322, and 1131 CELF1-bound transcripts, respectively. In addition, mining the ENCODE project^40^, we identified two unpublished data sets of putative CELF1-bound transcripts in K562 and GM12878 cell lines (2359 and 147 transcripts, respectively). Curiously, no common factor was found when comparing these data sets that could point to a consensus CELF1 signature (Supplementary Fig. 6d).

### Novel CELF1-bound transcripts in melanoma found by RIP-Seq

**Fig. 3:**
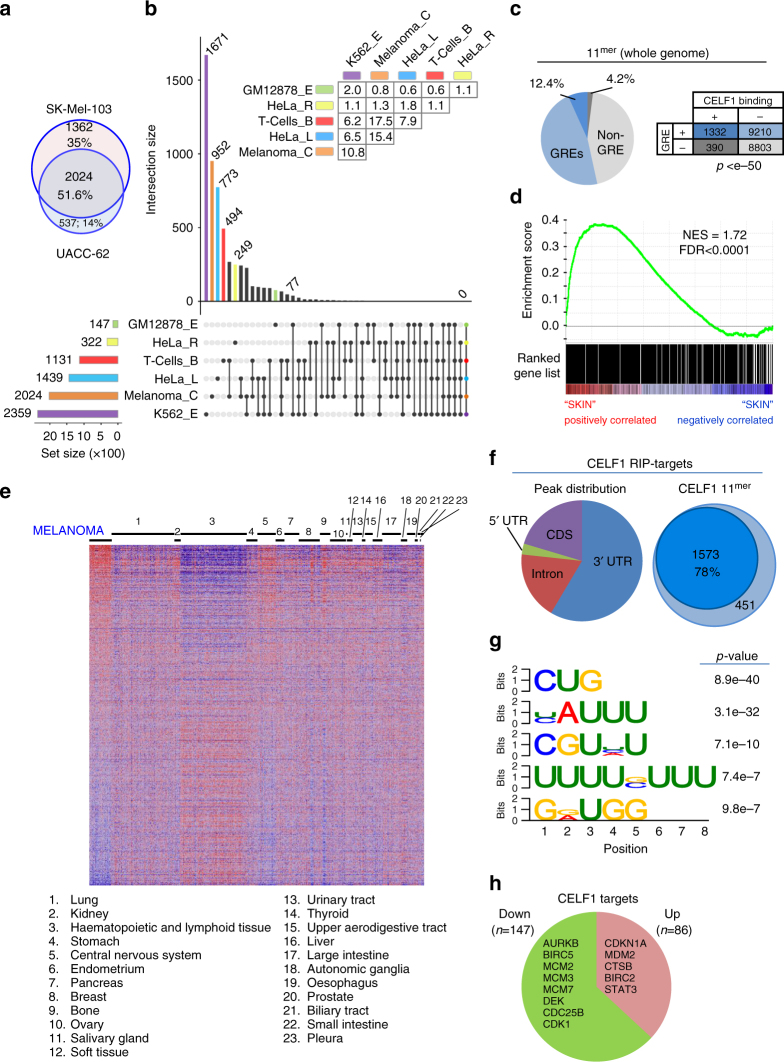
CELF1-bound targets in melanoma cells differ from other systems. **a** Venn diagram showing the numbers and corresponding overlap (%) of RNA immunoprecipitated transcripts. See Supplementary Fig. 1 and Supplementary Data 7 for additional information on experimental procedures and statistical analyses, respectively. **b** UpSet plot revealing the minimal overlap between the CELF1-RNA interactome in melanoma cells relative to the five publicly available cancer data sets. Each study is color coded. The horizontal bars summarize the number of genes. Circles and vertical lines in the *x*-axis mark the corresponding data sets being compared (the right-most data corresponding to the overlap of the six data sets). The vertical bar chart (*y*-axis) indicates the number of genes identified for each comparison. The number of unique genes for each data set are indicated on top of the corresponding bar. The inset table shows the overlap percentage in one-to-one comparisons. **c** Percentage of human-coding genes containing (blue) or lacking (gray) in their 3′ UTRs CELF1 consensus GREs (UGUUUGUUUGU and UGUGUGUGUGU, two mismatches allowed). The fraction of genes found by RIP-Seq to be bound by CELF1 in melanoma is indicated in darker colors. The contingency table summarizes the number of genes in each category. **d** Enrichment score plot of “melanoma-only” CELF1-bound targets across 24 tumor types of the CCLE (*n* = 54 and *n* = 752 melanoma and other cancers, respectively). NES normalized enrichment score. FDR false discovery rate. **e** Heatmap plot to visualize enrichment of CELF1-RIP targets in melanoma vs. the rest of tumors. **f** Localization of the melanoma CELF1 targets according to the peak density of the RIP-Seq analysis defined by the Piranha software. The Venn diagrams represent the fraction of genes with the 11mer GREs of **c** (see Supplementary Data 4 for detail). **g** Enriched sequences from the melanoma CELF1 RIP-Seq data defined by the DREME algorithm (*p* < 0.005). **h** Fraction of CELF1 targets (identified by RIP-Seq) found by HJAY to be downregulated (green) or upregulated (pink) in CELF1 depleted SK-Mel-103 and UACC-62 cells relative to the control transduced cells. Representative genes with roles in cell cycle progression and cell survival are indicated

Individual-nucleotide resolution crosslinking and immunoprecipitation sequencing (iCLIP-seq), a useful technique for the identification of consensus binding motifs of other RBPs as we recently reported in melanoma^19^, has been previously demonstrated to be biased for specific U-rich sequences^41^. For CELF1, this would represent the risk of trapping non-specific protein–RNA interactions^42^. Moreover, X-ray^43^ and nuclear magnetic resonance studies^44^ revealed that CELF1 is organized in complex 3D oligomeric structures with open and close conformations that could result in ambiguous readings of iCLIP. Therefore, we selected to perform RNA immunoprecipitations (RIP) using protocols we have proven efficient for RBPs such as the cytoplasmic polyadenylation factor CPEB4 in melanoma cells^20^. RIP was then followed by RNA sequencing (RIP-Seq), resulting in at least 10 million reads/sample aligned to the human genome (GRCh37/hg19). Filtering for significance (corrected *p*-value<0.05), this approach rendered 2024 CELF1-bound transcripts both in SK-Mel-103 and UACC-62 melanoma cells (representing a 51.6% overlap as depicted in the Venn diagrams of Fig. 3a). These CELF1-bound transcripts in melanoma were merged with the five available genome-wide analyses for CELF1 in human cancer cells described above. As summarized in the UpSet plot^45^ of Fig. 3b (see figure legend for explanation), the overlap with the different data sets of HeLa, T cells, K562, and GM12878 was very low, with no common targets to all these cell lines (see inset table in Fig. 3b for quantifications, Supplementary Fig. 7a for the corresponding Venn Diagrams, ad Supplementary Data 3 for additional information). This lack of binding conservation was rather striking, considering that derivatives of 11^mer^ UGUUUGUUUGU and UGUGUGUGUGU sequences that represent characteristic consensus GREs recognized by CELF1 in other systems^33^ were found in nearly half of the 3′ UTR of the human genome (Fig. 3c, blue sections in the pie chart, with data generated allowing two mismatches to account for the known variability of CELF1 GREs^42^).

The “melanoma-only” CELF1 RIP-Seq transcripts were then analyzed to determine whether they could represent a signature enriched in this malignancy. To this end, gene expression analyses were performed across the Cancer Cell Line Encyclopedia (CCLE), which contains transcriptomic profiles of 54 melanoma cell lines and over 750 cell lines of 23 tumor types^46^. As shown in the enrichment plots of Fig. 3d, the CELF1 melanoma-bound transcripts are indeed differentially expressed in this disease (Kolmogorov–Smirnoff test *p*-value of melanoma vs. other tumors <0.05; see heatmaps in Fig. 3e).

### CELF1 preferentially stabilizes transcripts with 3′ UTR GREs

The CELF1 RIP-seq data was then analyzed with the Piranha software^47^ to assign reads (peak calling) to 3′ UTRs, 5′ UTRs, introns or coding regions. This approach revealed a preference of CELF1 for 3′ UTR binding (Fig. 3f, left pie chart). Sequence Searcher^48^ confirmed the presence of UGUUUGUUUGU and UGUGUGUGUGU derivatives in 78% of the CELF1 RIP-seq transcripts (Fig. 3f, right chart). These corresponded to 12.4% of all genes with GREs at 3′ UTR sites (Fig. 3c, dark blue). Therefore, these data indicate that while the spectrum of genes with putative GREs is rather large, those recognized by CELF1 represent a restricted subset. The selectivity of CELF1 binding is illustrated in Supplementary Fig. 7b, with examples of cell cycle regulators with (*BIRC5*) or without (*AURKA*) detectable CELF1 binding, despite the presence of GRE sequences in both their 3′ UTRs (see also Supplementary Fig. 7b and Supplementary Data 4 for additional examples of cell cycle regulators such as MCM3 with no GREs and no CELF1 binding).

Interestingly, we found CELF1 bound to 4.2% of genes with no identifiable 11^mer^-GRE derivatives in their 3′ UTRs (Fig. 3c, dark gray). Therefore, an unbiased domain search was performed with the DREME motif discovery algorithm^49^. Five highly significant CELF1 binding sites were identified in melanoma cells, all of which were all GU-rich (Fig. 3g, Fisher’s exact test *p*-values ranging from 10^−7^ to 10^−40^). Together, these data confirm the GU-rich preference for sequence recognition of CELF1, but also emphasize a restricted and melanoma-selective subset of binding targets (Fig. 3a, c, d).

### Transcriptomic and proteomic analyses of CELF1 effectors

Next, genome-wide human junction arrays (HJAY, 5.4 million probes), were used to identify CELF1-bound targets whose expression (and alternative splicing as discussed below) indeed depends on CELF1. With this approach, 233 CELF1 RIP-Seq targets were found to undergo significant changes in mRNA expression after transduction of CELF1 shRNA (Fisher’s test *p*-value<0.05; see Supplementary Data 5). Consistent with previous roles of CELF1 in mRNA decay^33^, 37% of transcripts indeed accumulated in melanoma cells expressing shRNA (Fig. 3h and Supplementary Data 5). However, instead of apoptotic inducers (i.e., as *BAD*)^31^, we found higher levels of survival factors (e.g., *BIRC2* or *STAT3*). Moreover, over two thirds of CELF1 RIP-Seq targets were downregulated upon CELF1 depletion (Fig. 3h). These included a large set of cell cycle and cell division modulators (e.g., *BIRC5, AURKB, MCM2-3, CDC6*, or *CDK1*, among others). Interestingly, *DEK*, an oncogene we had previously linked to melanoma^50^ was also found as a binding target regulated by CELF1 (Fig. 3h). These data provide a mechanistic explanation as to why CELF1 depletion in melanoma cells results in an inhibited cell proliferation, instead of exiting from quiescence as reported in activated T cells^51^, or instead of apoptosis as described for laryngeal^37^, hepatocellular^33,52^ or oral squamous cell carcinoma^31,53^.

**Fig. 4:**
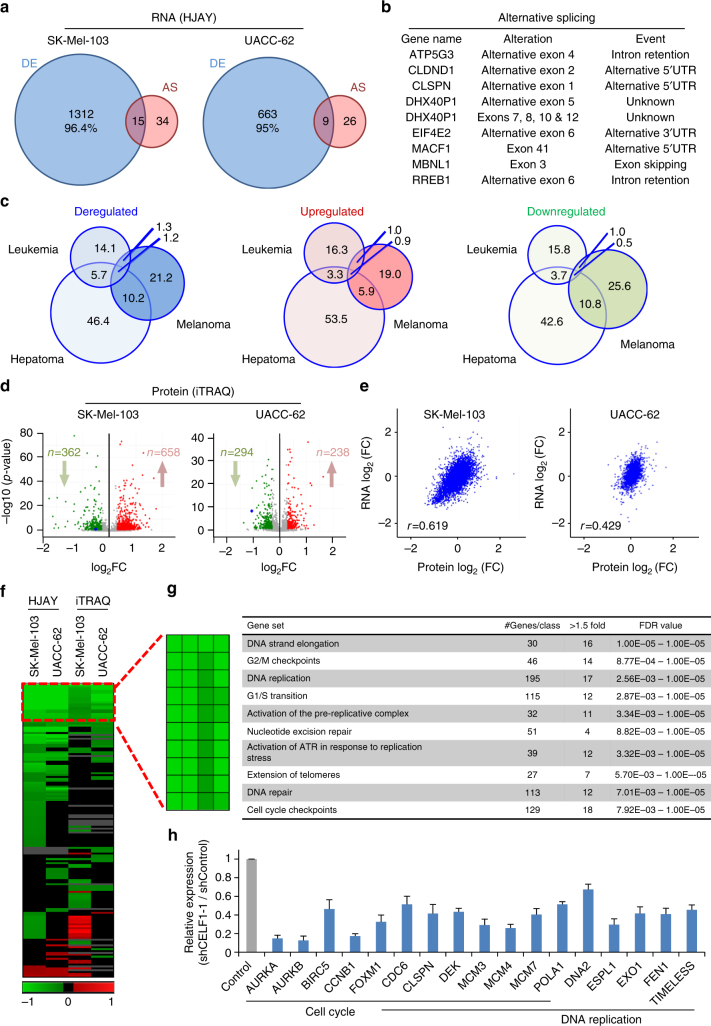
Genome-wide HJAY and iTRAQ LC–MS/MS identify new CELF1-regulated targets. **a** Differentially expressed (DE) and alternatively spliced (AS) genes identified by HJAY in SK-Mel-103 or UACC-62 cells transduced with shCELF1-1 estimated with respect to shRNA controls. The specific overlap in the mRNA expression changes is shown in Supplementary Fig. 7c, d. **b** Splicing alterations identified by the HJAY in SK-Mel-103 and UACC-62 upon CELF1 depletion. **c** Overlap (%) in changes in mRNA expression after depletion of CELF1 in melanoma cells (SK-Mel-103 and UACC-62) with respect to similar transcriptomic data available for K562 leukemia and HepG2 hepatoma cell lines (ENCODE ENCSR605MFS and ENCSR695XOD, respectively). The diameter of the Venn diagrams is proportional to the genes with mRNA changes in each data set. **d** Up- and downregulated proteins identified by iTRAQ LC–MS/MS analyses in SK-Mel-103 or UACC-62 cells upon shCELF1-1 transduction estimated with respect to shRNA controls. Up- and downregulated proteins (red and green, respectively) are shown as volcano plots. Factors indicated in green and red are those with significant changes in protein expression. Non significant changes in expression are labeled in gray (additional information for the relative overlap in the iTRAQ data for the cell lines analyzed is summarized in Supplementary Fig. 7e, f). **e** Correlation of global changes in RNA and protein levels upon CELF1 depletion in SK-Mel-103 and UACC-62 melanoma cell lines. **f** Heatmap of gene sets found enriched (Reactome pathway database) both at RNA and protein levels in shCELF1-1 expressing melanoma cells, listed as a function of FDR values. **g** Top-10 downregulated cellular functions identified in the two cell lines analyzed. Listed are enriched gene sets, the total number of genes in each category, the number of genes deregulated in both cell lines, and the range of FDR values (<0.25). **h** Expression levels of representative modulators of cell cycle and/or DNA replication upon CELF1 depletion in UACC-62 cells, as validation of data obtained from HJAY. Error bars correspond to SEM of three experiments in triplicate

An attractive challenging feature of RBPs is their potential to act as signal amplifiers or “regulator of regulators”^24^. To this end, we extended the analysis of human exon arrays (HJAY) shown above for CELF1-bound transcripts, to the whole genome. In addition, we performed proteomic analyses using the 4-plex isobaric tag for absolute and relative quantification (iTRAQ). HJAY identified 1361 and 698 altered genes upon CELF1 depletion in SK-Mel-103 and UACC-62, respectively (Fig. 4a, see Supplementary Fig. 7c, d for the correlation and overlap in upregulated and downregulated genes in the two cell lines). Of those changes, only 5% corresponded to alternative splicing events (see Fig. 4a, b and Supplementary Table 3 for additional information). However, structure-based analyses or immunoblotting failed to identify functional consequences of these splicing alterations in protein isoforms (not shown).

The vast majority of alterations detected by HJAY corresponded to differential gene expression (Fig. 4a, blue circles), with particular downregulation of pro-tumorigenic cell cycle regulators (see below in Fig. 4f–h). To determine whether these changes in mRNA expression are also selective for melanoma cells, we performed comparative analyses to data in leukemia and hepatocellular cancer cell lines available in ENCODE (ENCSR605MFS and ENCSR695XOD, respectively). Intriguingly, and as the case for CELF1-RNA interactome (Fig. 3a), the overlap in these CELF1-controlled transcriptomic sets was minimal (Fig. 4c).

With respect to iTRAQ, LC–MS/MS (liquid chromatography–mass spectrometry) revealed 1020 and 532 proteins deregulated by CELF1 depletion in SK-Mel-103 and UACC-62 cell lines, respectively (Student’s *t* test *p* < 0.05; see Volcano plots in Fig. 4d; and the specific overlaps in Supplementary Fig. 7e, f). Interestingly, proteomic changes were positively correlated with transcriptomic profiles for both cell lines (Fig. 4e). These results also separate melanomas from epithelial tumors, where CELF1 has been reported to control the translation of metastatic genes^54^.

The transcriptomic and proteomic data sets were then subjected to gene set enrichment analyses (GSEA), using GO and the Reactome database (Fig. 4f). Collectively, the top ten GO terms controlled by CELF1 at the mRNA and protein levels were related to DNA replication, cell cycle control and DNA repair (Fig. 4g; see validation in Fig. 4h).

### Omics data to build CELF1-regulated networks

**Fig. 5:**
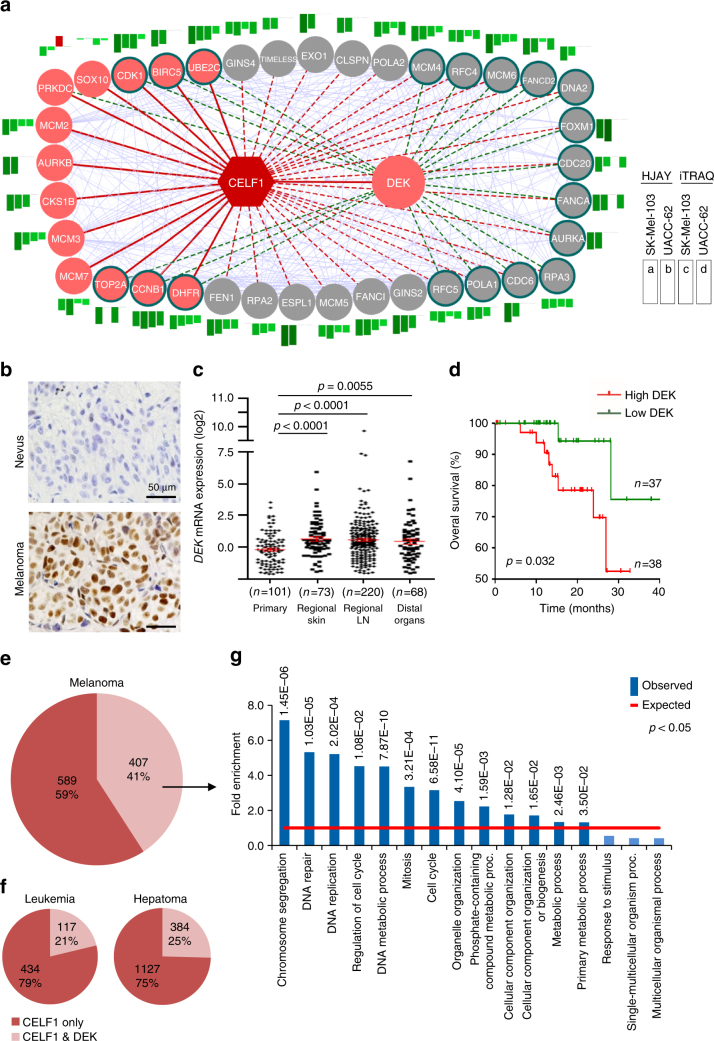
Downstream effectors of CELF1. **a** Gene network identified by comparing HJAY, iTRAQ and RIP-Seq data, followed by manual curation to confirm gene function on the basis of literature search (see Supplementary Data 6 for additional detail). Protein interactions (light blue lines) were extracted from the STRING database. CELF1 direct interactors (RIP-Seq) are indicated in pink. Genes with deregulated protein and mRNA levels in CELF1-depleted melanoma cells, but not found bound to this RBP by RIP-Seq are labeled in gray. DEK regulated genes are marked with green borders. Bar charts refer to changes in RNA (**a**, SK-Mel-103; **b**, UACC-62) and protein (**c**, SK-Mel-103; **d**, UACC-62) levels in shCELF1-1 vs. shControl cells. **b** Representative micrographs of benign nevi and primary melanomas showing an elevated DEK protein expression (brown staining) during tumor progression. Nuclei were counterstained in blue with hematoxilin. **c** *DEK* mRNA expression levels of melanoma samples grouped by disease stage (data retrieved from TCGA database). **d** Overall survival of melanoma patients (shown in a Kaplan–Meier survival plot) with DEK levels above (high) or below (low) the median expression identified in the TCGA data set for primary melanomas. Indicated are *p*-values estimated with the Gehan–Breslow–Wilcoxon test; *p*-(Log-rank test)=0.041. **e**, **f** Distribution of genes deregulated at the mRNA level upon CELF1 depletion in each indicated cancer type, plotted on pie-charts to visualize the fraction (light colors) of genes altered also by DEK depletion as defined by cDNA microarrays in this study. **g** Identification of overrepresented biological processes in genes downregulated both by CELF1 and DEK depletion using all genome as background (*p* < 0.05). Observed vs. expected ratios were defined using the statistical overrepresentation test on the PANTHER database (see Table S8 for additional information)

CELF-1 regulated pathways at protein and RNA levels (Fig. 4f) were then integrated with the RIP-Seq data to define direct vs. indirect targets of CELF1 in melanoma cells. Association networks were identified with the STRING database^55^, followed by manual curation to ensure proper functional annotation. This strategy revealed a highly interconnected network of 37 genes with dual mRNA/protein downregulation upon CELF1 depletion (Fig. 5a; see the magnitude of the changes as bar graphs in the insets, and the specific gene functions in Supplementary Data 6). These genes were classified in two categories. The first group corresponded to 14 direct CELF1 targets, which included AURKB, BIRC5, CDC6, CDK1, MCM2-3, DEK, and additional modulators of cell division and cell proliferation (Fig. 5a, pink circles). The second group was constituted by secondary targets of CELF1, namely, factors not bound to CELF1, but being significantly inhibited at the protein and RNA levels when this RBP is downregulated (Fig. 5a, gray circles). These included essential components of the pre-replication complex (e.g., CDC6, MCM4/6), as well as critical factors for initiation and progression of DNA replication (including DNA2, POLA1, POLA2, RPA2/3, RFC4/5, CDC20, and GINS2/4), precisely the phenotype observed for CELF1 depletion in melanoma cells (Fig. 2c, d). Additional relevant CELF1-controlled genes included the DNA repair factors FANCA, FANCD2, and FANCI (Fig. 5a), key also for an appropriate progression through cell cycle.

### DEK is an amplifier of proliferative roles of CELF1

A pending question in the RNA biology field is to identify “signaling amplifiers” that further expand the impact of selective RBPs in the control of pro-tumorigenic signals. The oncogene DEK was an interesting candidate to act downstream of CELF1. Mechanistically, we had demonstrated critical roles of DEK in cell cycle progression and cell survival^50,56^, which resemble our findings described here for CELF1. Moreover, the overexpression of DEK protein we had reported in malignant melanomas^56,57^ (see examples in Fig. 5b), was now found to reflect a significant mRNA upregulation at early stages of melanoma progression (Fig. 5c), correlating with poor prognosis (Fig. 5d) as the case for CELF1 (Fig. 1c, d; Supplementary Fig. 5a). Therefore, we next questioned to which extent DEK could contribute to mRNA-expression changes driven by CELF1 in genes that this RPB does not bind directly. To this end, DEK was depleted in SK-Mel-103, and changes in RNA expression were defined by cDNA arrays. DEK depletion did not alter CELF1 protein or mRNA, but reduced mRNA expression of 41% of genes also downregulated by shCELF1 (see pie chart in Fig. 5e). PANTHER enrichment test^22^ on these CELF1 and DEK dually regulated genes (*n* = 407), revealed a highly significant over-representation of pathways involved in DNA replication, regulation of cell cycle, mitosis and DNA metabolism (Bonferroni-corrected binomial test *p*-values ranging from 10^−05^ to 10^−11^; Fig. 5g and Supplementary Table 4). As these are general processes deregulated in multiple cancers, we questioned overlaps with other systems. We thus crossed the DEK-modulated transcriptome with information available in ENCODE for CELF1 (i.e., in K562 leukemia cells, ENCSR605MFS; and in the HepG2 hepatoma cell line, ENCSR695XOD). Interestingly, the CELF1–DEK overlap in these systems (21 and 25%, respectively) was approximately half of that in melanoma cells (Fig. 5e, f). Together, these data identify a new distinct link between a RBP (CELF1) and an oncogene (DEK) in the control of DNA synthesis and cell cycle progression modulators in melanoma.

### DEK is a physiologically relevant CELF1 target

**Fig. 6:**
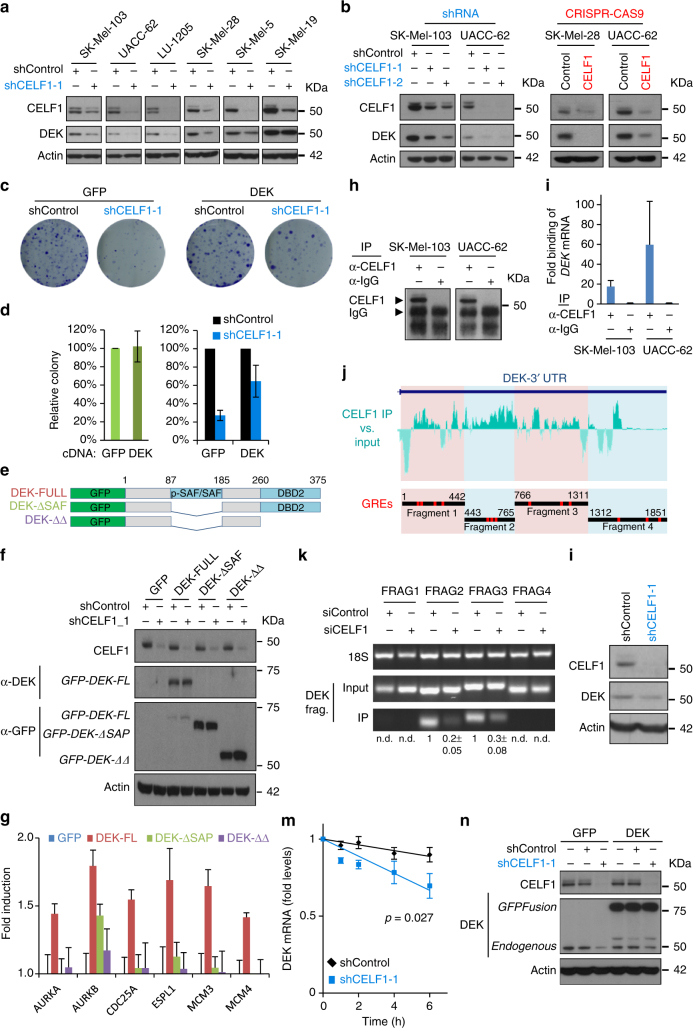
*DEK* is a bona fide CELF1 target. **a** CELF1 and DEK expression levels in indicated melanoma cell lines, detected by protein immnoblotting. **b** Immunoblots of the indicated cell populations to confirm DEK downregulation upon CELF1 depletion, for two shRNAs (blue) or CRISPR/Cas9 system (red). **c** Staining of colonies formed in the indicated cell populations transduced with full length DEK (or an empty GFP plasmid) and infected with control or shCELF1-1. **d** Quantification of data in **c**. Green bars represent the number of colonies of GFP vs. DEK in basal conditions. Those in the presence or absence of shCELF1 are shown in blue. Error bars correspond to SEM of three experiments. **e** Schematic representation of full length DEK and the GFP-tagged mutants to assess the impact of the DNA binding domain in the rescue of CELF1-depletion defects. **f** Expression levels of the different DEK constructs visualized with antibodies against GFP or the DEK central domain. **g** Differential impact of full length DEK vs. mutants to compensate for the inhibitory effect of CELF1 depletion. Data correspond to mRNA expression by qPCR represented relative to GFP or indicated DEK constructs infected CELF1-depleted cells. Error bars correspond to SEM of three independent experiments in triplicate. **h** CELF1 immunoprecipitation for subsequent qPCR-based validation of binding to *DEK* 3′ UTR, as quantified in **i**. Error bars correspond to SEM of two experiments. **j** Upper, histogram of RIP-Seq peak calling from identifying CELF1 binding enrichment at the 3′ UTR of the *DEK* mRNA, relative to input controls. Bottom panel, *DEK* 3′ UTR fragments cloned into lentiviral vectors to define CELF1 recognition. GREs are labeled in red. **k** Amplification of *DEK* 3′ UTR fragments after CELF1 RNA-IP, determined by semiqRT-PCR. **l** Protein depletion efficiency of shCELF1-1 shown by protein immunoblots compared to shControl induced UACC-62 cells. **m** *DEK* mRNA levels after actinomycin D treatment in indicated UACC-62 populations expressed as a function of time. Error bars correspond to SEM of three experiments in triplicate. **n** Transduction of UACC-62 melanoma cells with GFP or GFP-DEK cDNA (i.e., lacking 3′ UTR) for subsequent transduction of control or CELF1 shRNA, followed by protein immunoblotting

CELF1 depletion (by shRNA) reduced DEK expression in 5 out of 6 cell lines (Fig. 6a) further confirming the transcriptomic and proteomic data on SK-Mel-103. Similar results were obtained for an alternative shRNA and by CRISPR–Cas9 (Fig. 6b and data not shown). To demonstrate that the downregulation of DEK downstream of CELF1 depletion was the cause, not the consequence of cell cycle arrest, representative melanoma cells (UACC-62) were treated with well-known pharmacological inhibitors of BRAF (vemurafenib), MEK (U0126), or PI3K (GDC-0941). Importantly, none of these compounds had a significant impact on DEK (or CELF1) expression, although they induced a marked cell cycle arrest (see immunoblots and cell cycle profiles in Supplementary Fig. 8a, b).

Next, we tested the ability of DEK to rescue the proliferative defects of CELF1-depleted cells. Interestingly, ectopic expression of DEK cDNA largely counteracted the inhibitory effect of CELF1 shRNA on colony formation (Fig. 6c), without increasing basal proliferation (Fig. 6d). To further address this rescue activity, melanoma cells were transduced with DEK mutants lacking DNA binding activity (Fig. 6e, f). Specifically, we tested (i) DEK constructs deleted for amino acids 87–186, which encompass a distinct pseudo-SAF/SAF-box (scaffold attachment factor domain) with potent DNA binding and supercoiling effects^58^ and (ii) mutants devoid also of amino acids 260–350, a domain that binds DNA, but displays weak supercoiling activity^58^ (see schematic in Fig. 6e). Cells expressing full length or either of these mutants were then transduced with CELF1 shRNA (Fig. 6f). While full length DEK (1–375) maintained the expression of cell cycle regulators otherwise inhibited by CELF1, this was not the case for the double deletion mutant DEK-(Δ87-186)-(Δ260-375), called DEK-Δ−Δ for simplicity (Fig. 6g). This lack of activity was similar for DEK-(Δ87-186) (Fig. 6g), supporting the SAF domain as a main mediator of the DEK effects in this system.

### CELF1 stabilizes *DEK* mRNA by binding to GREs at the 3′ UTR

Immunoprecipitation and amplification by RT-PCR confirmed the binding of endogenous CELF1 to the 3′ UTR of *DEK* (see Fig. 6h, i for results in SK-Mel-103 and UACC-62). The 3′ UTR of *DEK* (found to contain 13 GREs as marked in red in Fig. 6j) was cloned in four fragments to experimentally define the domains recognized by CELF1 (Fig. 6j). Four isogenic UACC-62 cell populations were then generated to express these individual *DEK* 3′ UTR fragments, for subsequent transduction of control or CELF1 siRNAs (Supplementary Fig. 8c). As shown in the immunoprecipitations of Fig. 6k and Supplementary Fig. 8c, d, CELF1 was found to bind the central area (fragments 2 and 3) of *DEK* 3′ UTR.

To demonstrate that CELF1 controls the mRNA stability of DEK, transcription was blocked with actinomycin D in control and CELF1-depleted cells, and *DEK* mRNA half-life was measured thereafter by RT-PCR. As summarized in Fig. 6m, CELF1 depletion significantly shortened *DEK* mRNA half-life. Importantly, this destabilizing effect of CELF1 shRNA was lost in DEK constructs that lack the 3′ UTR (Fig. 6n). Together, these data reveal a new mechanistic interplay between CELF1 and the DEK oncogene whereby CELF1 stabilizes the *DEK* mRNA by binding at its 3′ UTR ultimately favoring a sustained expression of DEK protein.

### DEK–CELF1 correlation in melanoma and other tumor types

**Fig. 7:**
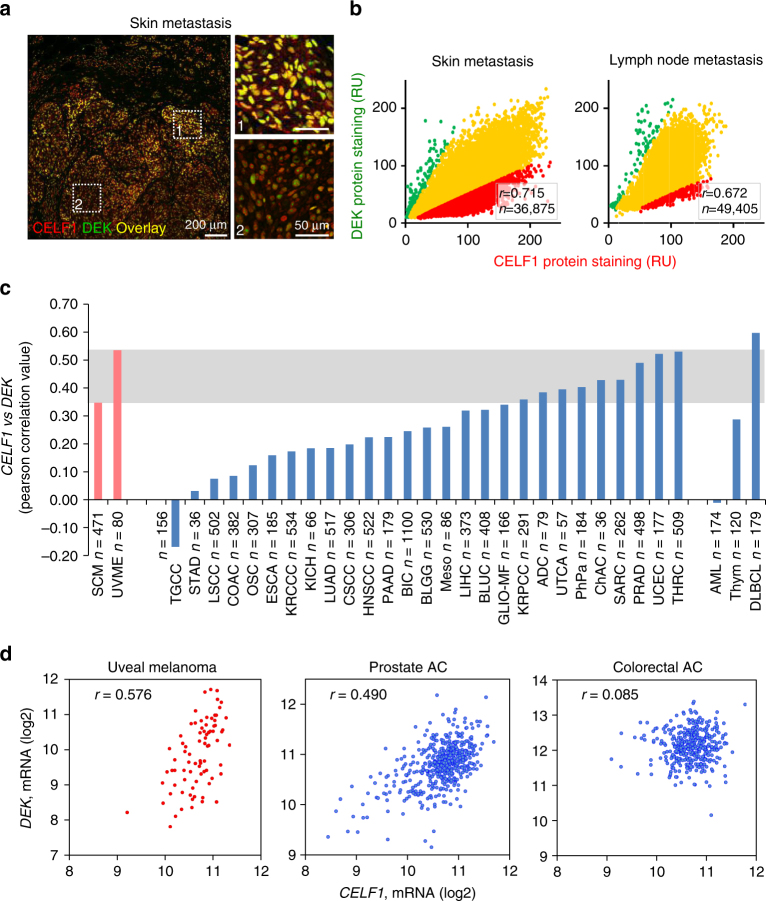
DEK expression correlates with CELF1 expression in vivo. **a** Single-cell quantification analysis of CELF1 (red) and DEK (green) protein expression by confocal microscopy in a representative skin metastasis of human melanoma. Images on the right show higher magnification of selected areas of the lesion with high or low expression of both proteins (insets 1 and 2, respectively). **b** Quantification of nuclear staining of CELF1 and DEK in skin and lymph node melanoma metastasis human biopsies, respectively. Relative nuclear staining of CELF1 and DEK plotted in a maximum range of RGB color scale (0–255). Data points were pseudo-colored based on red/green signal intensity ratio (red, ratio≥2; green, ratio≤0.5; yellow, 0.5 < ratio <2). *r*, Pearson correlation; *n*, total number of quantified cells in the whole tissue. **c** *DEK* vs. *CELF1* mRNA expression in various cancer types plotted as a function of corresponding significance (Pearson correlation value) ADC adrenocortical carcinoma, AML acute myeloid leukemia, BIC breast invasive carcinoma, BLGG brain lower grade glioma, BLUC bladder urothelial carcinoma, ChAC cholangiocarcinoma, COAC colorectal adenocarcinoma, CSCC cervical squamous cell carcinoma and endocervical adenocarcinoma, ESCA esophageal carcinoma, GLIO-MF glioblastoma multiforme, HNSCC head and neck squamous cell carcinoma, KICH kidney chromophobe, KRCCC kidney renal clear cell carcinoma, KRPCC kidney renal papillary cell carcinoma, LIHC liver hepatocellular carcinoma, LNDLBCL lymphoid neoplasm diffuse large B-cell lymphoma, LSCC lung squamous cell carcinoma, LUAD lung adenocarcinoma, Meso mesothelioma, OSC ovarian serous cystadenocarcinoma, PAAD pancreatic adenocarcinoma, PhPa pheochromocytoma and paraganglioma, PRAD prostate adenocarcinoma, SARC sarcoma, SCM skin cutaneous melanoma, STAD stomach adenocarcinoma, TGCC testicular germ cell cancer, THRC thyroid carcinoma, Thym thymoma, UCEC uterine corpus endometrial carcinoma, UTCA uterine carcinosarcoma, UVME uveal melanoma. *n*, number of samples. **d** *DEK* vs. *CELF1* mRNA in uveal melanoma (*n* = 80), prostate adenocarcinoma (*n* = 498) and colorectal adenocarcinoma (*n* = 382) patients (data obtained from TCGA) shown on correlation scatter plots

A corollary of our findings on the CELF1–DEK functional interplay is that both genes should be positively correlated in human melanoma biopsies. This hypothesis was confirmed by automated single-cell histological analyses of both proteins in paraffin-embedded melanoma sections (see examples for skin metastasis in Fig. 7a; and quantifications of this correlation in skin and lymph node metastases in Fig. 7b).

Genome-wide data for DEK in cancer cells are scarce^59,60^, although this gene is overexpressed in multiple tumor types^50,61,62^. Therefore, we questioned to which extent the CELF1–DEK correlation found here in cutaneous melanomas was a generalized feature of malignant cells. To this end, we performed a meta-analysis of the TCGA platform, which archives mRNA information for both genes in uveal melanoma and 30 different tumor types, in addition to cutaneous melanoma. This strategy allowed an analysis of a total of *n* = 9341 clinical biopsies as summarized in the *X*-axis of Fig. 7c. Interestingly, we found an even higher *CELF1–DEK mRNA* correlation in uveal melanomas than in cutaneous melanomas (see red bars in Fig. 7c, and correlation plots in Fig. 7d, left panel). Moreover, ten cancer types were found with positive CELF1–DEK correlations in the range of those for skin and uveal melanomas (Fig. 7c; see examples for prostate adenocarcinoma in Fig. 7d, middle panel). However, in the rest of the tumors, these correlations were weak or negligible (Fig. 7d, right panel), and for cases such as acute myeloid leukemia and testicular germ cell cancer, there were even inverse (Fig. 7c). Together, these results expand the impact of our results to other pathologies, but also emphasize tumor-type selectivity in the regulation of CELF1 and DEK.

## Discussion

Recent genome-wide analyses have reported a broad spectrum or RBPs undergoing frequent mutation and gene amplification in multiple tumor types^3,4^. Here we show that melanomas, despite presenting with the largest mutational rate described to date^6^, and accumulating a plethora of transcriptomic changes^9^, largely spare the more than 650 mRBPs from mutations or CNVs. Instead, using computational analyses, genome-wide RNA sequencing and customized oligo arrays, we identified CELF1 within a selective set of mRBPs regulated in melanoma cells specifically at the mRNA level. CELF1 stood out among these mRBPs for three features that supported a relevant and selective function in melanoma: (1) no previous links to cutaneous pathologies, (2) an early induction in melanoma biopsies, and (3) a positive correlation with patient prognosis not shared by other RBPs or by neighboring at chromosome 11p11.2. Transcriptomic, proteomic, and RNA-immunoprecipitation studies, together with loss-of-function analyses and evaluation of patient prognosis, confirmed the relevance of CELF1 as a driver of cutaneous melanoma, with the oncogene DEK as a signal amplifier. Positive correlations between *CELF1* and *DEK* mRNA expression in uveal melanoma and ten additional tumor types (including diffuse large B cell lymphoma, thyroid carcinoma or prostate adenocarcinoma) further reinforce the translational relevance of our results.

We had initially selected CELF1 for its potential to modulate splicing, translation and mRNA stability^33,39^. In particular, we paid attention to CELF1 targets repressed (by mRNA decay) in the context of pro-apoptotic genes described in HeLa^33^ or in cells from oral squamous carcinoma^31^ and laryngeal cancer^37^. However, we found only 5% of genes undergoing alternative splicing in CELF1-depleted melanoma cells, and no significant accumulation of death regulators. Instead, the combination of transcriptomic and proteomic analyses (RIP-seq, RNA-Seq and iTRAQ) identified a key role of CELF1 in the stabilization of a distinct and specific set of mRNAs in melanoma. Thus, while over half of protein coding genes in the human genome contained GREs that could represent putative CELF1 recognition sites, only 12% of these transcripts were bound by CELF1 in melanoma. Moreover, these CELF1-immunoprecipitated transcripts corresponded to genes enriched particularly in this disease. In fact, perhaps one of the most unanticipated findings of this study was the minimal overlap in the RNA-interactome of CELF1 in melanoma with respect to data reported in the literature for HeLa^33,34^ and T cells^39^, or to data sets available in ENCODE (i.e., K562 and GM12878 cells lines). The mechanisms underlying this variability will have to be defined in future studies. However, it is tempting to speculate a tumor type-dependent expression or usage of 3′-UTRs, as well as differential post-transcriptional modifications (such as phosphorylation) that can alter the stability and affinity of CELF1 for GRE sites^39^.

The systems approach in this study (namely, the combination of RIP-seq, RNA-Seq and iTRAQ, with computational studies, functional analyses and evaluation of patient prognosis) further illustrated a selective role of CELF1 in the control of cell cycle and proliferation genes. In particular, these techniques were proven as a powerful platform to identify new drivers of a long-proposed “regulator of regulators” function of CELF1^24^. Here we showed that via the oncogene DEK, CELF1 could control the mRNA and protein expression of key DNA replication factors such as MCM4, MCM6, RFC4, RFC5, CDC6, or POLA1, without direct binding to its transcripts. Mechanistically, we found that CELF1 extends the half life of the *DEK* mRNA by binding to GU-rich regions at its 3′ UTR, a role distinct from mRNA decay reported in other systems^51^. Whether this stabilizing function of CELF1 reflect tumor-specific protection against deadenylases or miRNAs^63^ deserves future attention.

It should be noted that while this study focuses on CELF1, RNA-Seq and the oligo-arrays described here identified multiple RBPs upregulated in melanoma cells. Therefore, it will be interesting to address to which extent this melanoma RBP landscape is shared with other tumor types. In particular, genes modulating 3′ UTR-associated functions may provide insight into the control of lineage specification as we recently reported for the cytoplasmic polyadenylation factor CPEB4^20^. In this context, unbiased transcriptomic and proteomic data sets generated here may serve as a tractable platform for the identification of new roles of RBPs controlling the still elusive mechanisms underlying lineage specificity or tumor-type identity.

## Methods

### Cell culture

The human melanoma cell lines SK-Mel-5, SK-Mel-19, SK-Mel-28, SK-Mel-29, SK-Mel-103, and SK-Mel-147 were initially obtained from David Polsky from the Memorial Sloan Kettering cell repository. G-361 and UACC-62 were from the ATCC. WM-1366 and LU-1205 were provided by Meenhard Herlyn’s group at the Wistar Institute (see Supplementary Table 1 for genetic backgrounds). These cells were cultured in Dulbecco’s modified Eagle’s medium (DMEM, Lonza) supplemented with 10% fetal bovine serum (FBS, Lonza) and 100 μg/mL penicillin/streptomycin (Invitrogen). Cell lines have been authenticated using GenePrint 10 Loci Service. Primary human melanocytes, keratinocytes, and fibroblasts were isolated from foreskins of healthy donors by differential tripsinization^20^. Melanocytes were cultured in Medium 254 (Invitrogen) supplemented with 1% melanocyte growth factors (HMGS, Invitrogen), 0.2 mM CaCl_2_, and 100 μg/mL penicillin/streptomycin; keratinocytes were cultured in Epilife Medium (Invitrogen) supplemented with 1% keratinocyte growth factors (HKGS, Invitrogen), 0.2 mM CaCl_2_ and 100 μg/mL penicillin/streptomycin and fibroblasts were cultured in DMEM, supplemented with 10% FBS, and 100 μg/mL penicillin/streptomycin. All cell cultures were tested for mycoplasma contamination routinely.

### Protein immunoblotting

Cells were harvested and total cell lysates were obtained using Laemmli buffer (62.5 mM Tris–HCl pH 6.8, 2% SDS, 10% glycerol, and 5% β-mercaptoethanol) and boiled at 95 °C for 7 min. Protein immunoblots were performed according to standard procedures using Immobilon-P membranes (Millipore) and Mini Trans-Blot Cell system (Bio-Rad Laboratories). For primary and secondary antibodies (providers and dilutions used), see Supplementary Table 5. Uncropped scans of the most important blots are provided Supplementary Figs. 9 and 10.

### Gene silencing (shRNAs, CRISPR gRNAs, and siRNAs)

Virus production for shRNA and gRNA infections were performed in 293FT cells by classical calcium phosphate precipitation^20^. Downregulation efficacy was determined after puromycin selection (1 µg/mL) by protein immunoblotting or RT-qPCR. For CRISPR/Cas9 gRNA cloning, the forward/reverse pair of oligos were phosphorylated, annealed, and ligated with the BsmBI (Thermo Fisher) for cloning into lentiCRISPR v2 plasmid^64^. Lentivirus was produced and cells were infected as described previously^65^. All targeting sequences are listed in Supplementary Table 6.

### RNA extraction, PCR, and quantitative RT-PCR

Total RNA was extracted and purified from cell pellets using RNeasy Mini-Kit (Qiagen) and reverse-transcribed into cDNA using the high capacity cDNA reverse transcriptase kit (Applied Biosystems) following the manufacturer’s instructions. 20 ng of the total cDNA were subjected to quantitative RT-PCR (60 °C annealing temperature) using Power SYBR Green PCR Master Mix (Applied Biosystems). Assays were run in triplicates on the 7900HT Fast Real-Time PCR system (Applied Biosystems) or QuantStudio 6 Flex Real-Time PCR System (Applied Biosystems). HPRT and GAPDH were used as loading control to normalize mRNA expression. The primer sequences and annealing temperatures listed in Supplementary Table 7. When indicated, ImageJ software was used to quantify protein band intensities.

### Cloning of *DEK* 3′ UTR fragments and deletion mutants

*DEK* 3′ UTR fragments were amplified with the indicated primers listed in Supplementary Table 6 to obtain the following fragments (UG repeats underlined).

- Fragment 1
  gauagaggacagagaagaugacucguucccauagauuugaagaucugauuuauaccauuauaccagcaaagagaauguauuuccuuuucuaaauccuuguuaagcaacguuaguagaacuuacugcugaccuuuuuaucuugaguguuaugugaauuugaguuugcuguuuuaaauugcauuucuaugccauuuuuaguuuaaaaucuugcauggcauuaauuguuccuugcuuuuauaguuguauuuuguacauuuuggauuucuuuauauaaggucauagauucuugagcuguugugguuuuuagugcacuuaauauuagcuugcuuaaggcauacuuuuaaucaaguagaacaaaaacuauuaucaccaggauuuauacauacagagauuguaguauuuaguauaugaaauauuuugaauacacaucucugucagugug
- Fragment 2
  aaaauucagcggcaguguguccaucauauuaaaaauauacaagcuacaguuguccagaucacugaauuggaacuuuucuccugcauguguauauaugucaaauugucagcaugacaaaagugacagauguuauuuuuguauuuuuaaaaaacaauugguuguauauaaaguuuuuuuauuucuuuugugcagaucacuuuuuaaacucacauagguagguaucuuuauaguuguagacuauggaaugucaguguucagccaaacaguaugauggaacagugaaagucaauucagugauggcaacacugaaggaacaguuaccc
- Fragment 3
  ugcuuugccucgaaagugucaucaauuuguaauuuuaguauuaacucuguaaaagugucuguagguacguuuuauauuauauaaggacagaccaaaaaucaaccuaucaaagcuucaaaaacuuugggaaagggugggauuaaguacaagcacauuuggcuuacaguaaaugaacugauuuuuauuaacugcuuuugcccauauaaaaugcugauauuuacuggaaaccuagccagcuucacgauuaugacuaaaguaccagauuauaaugccagaauauaaugugcaggcaaucguggaugucucugacaaagugugucucaaaaauaauauacuuuuacauuaaagaaauuuaauguuucucuggaguuggggcucuuggcuuucagaguuugguuaaucaguguugauucuagaugaucaacauaauggaccacuccugaaugagacuuaauuuugucuuucaaauuuacugucuuaaaucaguuuauuaaaucugaauuuuaaaacaugcuguuuaugacacaaugacacauuuguugcacc
- Fragment 4
  aauuaaguguugaaaaauaucuuugcaucauagaacagaaauauauaaaaauauauguugaauguuaacagguauuuucacagguuuguuucuugauaguuacucagacacuagggaaagguaaauacaagugaacaaaauaagcaacuaa augagaccuaauaauuggccuucgauuuuaaaua uuuguucuuauaaaccuugucaauaaaaauaaaucuaaaucacu gguguuuuaagucacuugcauuugauaucuuauagguguauauagcauuuccuuauggggaauaauucu gaaaagggauuuuuaaauugauucagcccuauaaccuauacaauuuggaauacucuuuugugguaauggacca uuuucuuggagugacuucacaaaacugaauaauuaagguuaauuuuaugaccauuugcuauaaagaagg uaaguagucaugcauagaguauuuggaagugaggaaauggaguuguucuguauga aaguucuugaugaguacgcacacuuauuaaauggauguau cacca

PCR products were purified with PCR purification kit (Qiagen) following manufacturer instructions. Amplified fragments and empty pLV-CMV-SV40-Puro vector were digested by XhoI (NEB Inc.) and BamHI (NEB Inc.) with NEBuffer 3 (NEB Inc.) and BSA (NEB Inc.). Ligation was performed (3:1, insert:vector molar ratio) with T4 DNA Ligase (NEB Inc.). Insertion was checked by sequencing.

Full-length DEK and the Δ-SAF and the double SAF-C end deletion mutants (see schematic of constructs in Fig. 6e) were cloned in a pTRIPZ-EGFP vector as follows: The pTRIPZ vector (Open Biosystems), a doxycycline inducible vector originally used for inducible expression of miRNAs, was custom tailored for protein overexpression. First, the tRFP cassette of the pTRIPZ vector was replaced by a new MCS, where the eGFP cDNA derived from the pEGFP-N1 vector was inserted via new XhoI/SnaBI sites. Next, the DEK cDNA was cloned into the pTRIPZ-EGFP vector, digested with EcoRI/SnaBI via a 3-fragment ligation: DEK bp 1–471 was isolated by restriction digest of the pGEX4T1-DEK vector using EcoRI/NsiI; DEK bp 472–1125 was produced from the pGEX4T1-DEK vector via PCR using the primer pair 5′-GATGCTTAAGCTTCTCGAGGCCACCATGGTGAGCAAGGGCGAG-3′ and 5′-GCGGTACGTAGCGGCCGCTCAAGAAATTAGCTCTTTTACAG-3′ and digestion with NsiI/SnaBI. The resulting pTRIPZ-EGFP-DEK plasmid was analyzed by sequencing. For cloning of the Δ-SAP construct a NLS-site was added to the pTRIPZ backbone, and a PCR product from the pGEX4T1-DEK using the primer pair 5′-CAA GGA TCC GAA TTC AGG CGC GCC CCA AAG CCT TCT GGC AAA CCA TTG CCG-3′ and 5′-CTT GGC GCG CCG TTT CTG CCC CTT TCC TTG TGC AAT TGT AAA TGG C-3′, was cloned in using EcoRI/SnaBI. A similar procedure was carried for the DEK (Δ−Δ) derivative.

### Growth curves and colony formation assays

For growth curves, after CELF1 depletion, 1 × 10^3^ melanoma cells were plated in 96-well optical bottom plates at day 4 after lentiviral transduction of CELF1 shRNAs. At the indicated time intervals (Day 0 is the following day after seeding), cells were fixed with 4% paraformaldehyde (Electron Microscopy Sciences) and stained with DAPI (Invitrogen). For each time point, total cell number was quantified in triplicates by automated high throughput confocal detection of DAPI-stained nuclei using the OPERA HCS platform and the Acapella Analysis Software (Perkin Elmer). Low confluency colony formation assays were performed by seeding 1 × 10^3^ (SK-Mel-103 and SK-Mel-147) or 5 × 10^3^ (SK-Mel-5, SK-Mel-19, SK-Mel-28, SK-Mel-29, UACC-62, and LU-1205) cells per well onto six-well plates. Cells were allowed to grow for 10–14 days, for subsequent fixation with cold methanol for 10 min. Colonies were stained with 0.4 g/L crystal violet (Sigma). The number of colonies was counted from micrographs of the plates using the ImageJ software.

### Analyses of cell cycle profiles

Cell synchronization at the G1/S phase of the cell cycle was performed in SK-Mel-103 cells by incubation with 2.5 mM thymidine (Sigma, T1895). Cells were fixed at 0, 4, and 8 h after release of the thymidine block. For BrdU pulse, exponentially growing cells were incubated with 10 µM BrdU (Sigma) for 1 h. Cells were fixed with ice cold 70% ethanol and processed for stainigng with FITC-conjugated anti-BrdU antibody (BD Pharmigen), and DNA was counterstained with 50 µg/mL propidium iodide (Sigma, P4864). Data were acquired using a FACS Calibur flow cytometer (Becton Dickinson). Cell aggregates were excluded using pulse processing and a minimum of 20,000 single events were measured. The FlowJo 9.6.4 software (Treestar) was used to define the percentage of cells in the G0/G1, S or G2/M phases of the cell cycle.

### Immunohistochemistry

Tissue microarrays were generated from paraffin-embedded specimens obtained from the i+12 Biobank (RD09/0076/00118) of the Hospital 12 Octubre and the Spanish National Biobank Network, with the corresponding informed consent and ethical protocols approved by their Clinical Investigation Ethical Committees. A total of 185 human specimens (47 benign nevi, 138 malignant melanomas stages II–IV) were stained with CELF1 antibody using the Bond Automated System (Leica Microsystems). After automated dewaxing and rehydration of the samples, heat-induced antigen retrieval was performed using Bond Epitope Retrieval Solution 2 (Leica Biosystems) and immunodetection was performed with Bond Polymer Refine Detection (Leica Microsystems) following the manufacturer’s instructions. CELF1 protein expression was scored blinded according to staining intensity by two independent dermatologists. The score system used for staining intensity was: 0 (no detectable), 1 (intermediate), and 2 (high). Digital images of IHC-stained sections were obtained at ×40 magnification (0.12 μm/pixel) using a whole slide scanner (Mirax scan, Zeiss) fitted with a 40×/0.95 Plan Apochromat objective lens (Zeiss).

### Immunofluorescence

Paraffin-embedded malignant melanoma tissues were processed for dual immunostaining of CELF1 (Abcam, ab9549) and DEK (Abcam, ab166624). Antigen retrieval was performed using 10 mmol/L sodium citrate buffer at pH 6. Secondary antibodies used were anti-rabbit Alexa Fluor 555 and anti-mouse Alexa Fluor 488 (Life Technologies) and DNA was counterstained with DAPI (Invitrogen). Negative controls were obtained by omitting the primary antibody. Image mosaics were acquired at 40xHCX PL APO 1.2 N.A. oil immersion objective using a TCS-SP5 (AOBS-UV) confocal microscope and “intelligent matrix screening remote control” (iMSRC) tool. Images were subsequently analyzed with Definiens XD software to determine CELF1 and DEK nuclear intensities per cell.

### RNA stability (actinomycin D treatment) assays

Control or CELF1 depleted cells were seeded on day 4 after infection (2.5 × 10^5^ cells/6 cm plate). Cells were treated with Actinomycin D (Sigma Aldrich, S9415) at 5 μg/ml concentration. After 30 min of pre-treatment, cells were washed with ice cold PBS and collected via scraping at indicated time points (0, 1, 2, 4, or 6 h). RNA extraction, quantification, reverse transcription and qPCR were performed as described above.

### Cell treatments

In order to check whether the regulation of DEK is cell cycle dependent, UACC-62 cells were treated with BRAF inhibitor vemurafenib (10 μM, Selleckchem, S1267), MEK inhibitor U0126 (5 μM, Calbiochem, 662005), PI3K inhibitor GDC0941 (Axon Medchem, 1377) or DMSO as a solvent control (1:1000, Merck, 103562) for 8 h or 24 h.

### Gene expression, CNV, and analysis of patient prognosis

Data for all available tumor types and annotated number of samples for each type were retrieved from the TCGA database using cBioPortal. For CNV, charts of indicated genes and scatter plots for dual-gene expression, data were downloaded and processed in Microsoft Excel. Data for disease stage CELF1, DEK, FUBP1, and KHDRBS1 expression was from TCGA repository and plotted on GraphPad Prism Software. The expression distribution of the different genes was assessed considering only mRNA expression of diploid genes (mRNA expression *z-*scores RNAseq V2 RSEM). Data for 479 melanoma patients were sorted by the stage at diagnosis: primary patients and metastatic patients (regional cutaneous metastasis, regional lymph node metastasis and distal organ metastasis.). *p*-Values were estimated by two-tailed unpaired Student's *t* test. Data for Kaplan–Meier survival curves for *CELF1*, *DEK*, *FUBP1*, *KHDRBS1*, *RAPSN*, *PTPMT1*, and *KBTBD4* were downloaded from cBioportal and evaluated with GraphPad Prism software. Overall survival of primary melanoma patients with annotated clinical information were studied up to 40 months. High and low mRNA expressions of each gene were defined with the statistical median calculation. *p*-Values were estimated by the log-rank test and the Gehan–Breslow–Wilcoxon test.

### RNA-Seq

Total RNA from SK-Mel-28, SK-Mel-147, and UACC-62 melanoma cells and from freshly isolated primary melanocytes was extracted and purified from cell pellets using RNeasy Mini-Kit (Qiagen). A volume of 1 µg of total RNA samples was reverse-transcribed into cDNA using the high capacity cDNA reverse transcriptase kit (Applied Biosystems) following the manufacturer’s instructions. Average sample RNA Integrity Number was 9.8 when assayed on an Agilent 2100 Bioanalyzer. PolyA+fraction was purified and randomly fragmented, converted to double stranded cDNA and processed through subsequent enzymatic treatments of end-repair, dA-tailing, and ligation to adapters as in Illumina’s “TruSeq Stranded mRNA Sample Preparation Rev. D” kit (catalog number 15031047). Adapter-ligated library was completed by PCR with Illumina PE primers (10 cycles). The resulting purified cDNA library was applied to an Illumina flow cell for cluster generation and sequenced on an Illumina HiSeq2500 following manufacturer’s protocols. Image analysis, per-cycle base calling and quality score assignment was performed with Illumina Real Time Analysis software. Conversion of Illumina BCL files to bam format was performed with the Illumina2bam tool (Wellcome Trust Sanger Institute—NPG). 50 bp single-end reads generated from the RNA-Seq experiment were analyzed with the next*presso* pipeline (http://bioinfo.cnio.es/nextpresso/), as follows: sequencing quality was checked with FastQC. Fastq files were randomly down-sampled to generate data sets with similar number of reads in all the samples.

### RNA-IP, sequencing, and computational analyses

Cells grown at 80% confluence were fixed in 1% formaldehyde for 10 min at room temperature, and fixation was stopped by adding 1 M glycine for 5 min. After washing with ice cold PBS, cells were collected by scraping and lysed with in NT2 buffer (50 mM Tris–HCl pH 7.5, 150 mM NaCl, 1 mM MgCl_2_, 0.5% Nonidet P-40, and 1 mM EDTA) supplemented with protease and RNase inhibitors (Applied Biosystems). For the solubilization of crosslinked complexes, lysates were sonicated in a Bioruptor Standard (Diagenode) for 10 min at medium intensity. After preclearing by protein A Dynabeads for 30 min at 4 °C, samples were quantified and equal amount of proteins were immunoprecipitated using CELF1 antibody or mouse IgG coupled to protein A Dynabeads (Invitrogen) for 3 h at 4 °C. RNA elution was done by two consecutive incubations at 55 °C for 30 min and at 65 °C for 45 min in NT2 buffer containing 50 µg proteinase K (Roche Applied Science), 1% SDS, 200 mM NaCl and 10 mM EDTA. Supernatants were collected and digested with DNase I for 10 min at RT. RNA was extracted with the TRI Reagent (Sigma) following manufacturer's protocol. For validation, independent RIP assays were performed. The total amount of RNA immunoprecipitated and 1 µg of RNA extracted from inputs were retrotranscribed using cDNA reverse transcriptase kit and qPCR were performed as described before. Sequencing was performed by the CNIO Genomics Unit. Integrity of RNA was evaluated by Agilent 2100 Bioanalyzer using RNA 6000 Pico kit following manufacturer’s recommendations. An estimated mass of 20 ng RNA per sample (1 μg for input samples) was processed with Ribo-Zero Gold Kit (Epicentre, RZHM11106/RZG1224) for removal of ribosomal RNAs. RNAs were randomly fragmented, converted to double stranded cDNA and processed through subsequent enzymatic treatments of end-repair, dA-tailing, ligation to adapters and amplification by PCR with Illumina PE primers. The purified cDNA library was applied to an Illumina flow cell for cluster generation (TruSeq cluster generation kit v5, Illumina, GD-203-5001) and sequenced on Illumina Genome Analyzer IIx with SBS TruSeq v5 reagents following manufacturer’s protocols. Fold binding enrichment of target mRNAs in the immunoprecipitated fraction was calculated after normalization with the gene expression from the inputs.

### Bioinformatic analyses for RNA-Seq and RIP-Seq

Procedures for RNA-seq and RIP-seq have been described before^20^ (see Supplementary Data 7 for the bioanalytic tools used in this study). Specifically, Fastq files with 40-nt single-end sequenced reads were quality-checked with FastQC v0.11.0 and aligned to the human genome (GRCh37/hg19) with TopHat-2.0.10, using Bowtie 1.0.0 and Samtools 0.1.1.9, allowing two mismatches with the following parameters for Tophat:--bowtie1 --read-edit-dist 2 --read-gap-length 2 --GTF Homo_sapiens/UCSC/hg19/Annotation/Genes/genes.gtf --no-coverage-search max-multihits 20 --library-type fr-firststrand --read-mismatches 2 --segment-mismatches 1 --segment-length 25 --splice-mismatches 0.

Transcript quantification and differential expression were calculated with Cufflinks 2.2.1, using the human GRCh37/hg19 transcript annotations from https://ccb.jhu.edu/software/tophat/igenomes.shtml, with the following parameters for Cuffdiff:-c 10 --library-type fr-firststrand --frag-bias-correct Homo_sapiens/UCSC/hg19/Sequence /BowtieIndex/genome.fa --multi-read-correct --max-bundle-frags 1000000 --seed 123L --FDR 0.05 --library-norm-method geometric and the following parameters for Cuffnorm: --library-type fr-firststrand --seed 123L --library-norm-method geometric Homo_sapiens/UCSC/hg19/ Annotation/Genes/genes.gtf.

RIP-seq peaks were called with Piranha 1.2.1 using the ZeroTruncatedPoissonRegression distribution, with a bin size of 20 and 0.05 FDR. From the total number of peaks obtained, only those with more than 30 reads were considered for further analysis. Peak annotation was performed with PeakAnalyzer 1.4, using the Homo sapiens GRCh37.72 annotation from Ensembl. Common peaks among the three samples were obtained with BEDtools 2.16.2and their corresponding nucleotide sequences were retrieved with the Ensembl API. CELF1 binding sequences in the binding region of the target were analyzed by Sequence Searcher allowing for two mismatches. Motifs enriched in the peaks were assessed with the DREME algorithm.

RNA-Seq and RIP-seq data have been deposited in NCBI’s Gene Expression Omnibus with accession number GSE88741 and GSE83231, respectively.

### Customized microarrays for RBP analyses

Melanoma cell lines SK-Mel-19 and SK-Mel-103, and three preparations of primary human melanocytes were harvested by trypsinization at confluency of ~70%. Pellets were stored at −80 °C. Total RNA was purified using RNeasy Mini kit (Qiagen) and digested with DNase (Qiagen). Quality assessment of Total RNA samples were analyzed using the Agilent Bioanalyzer 2100 and the RNA 6000 LabChip Kit (Agilent) with the Eukaryote Total RNA Nano Assay (Agilent). Cy5-Cy3 labeled cRNA were generated from the total RNA using the Agilent Low RNA Input fluorescent linear amplification kit (Agilent) and cyanine 5-CTP and cyanine 3-CTP (Perkin-Elmer); the cRNA was purified using the RNeasy Mini kit (Qiagen). Quality control was assessed by Agilent Bioanalyzer 2100. In total, 6 μg of each cRNA were used for the hybridization to a previously described array^23^ using an Agilent in situ Hybridization Kit Plus. Three biological replicates of melanoma cells were hybridized to pools of melanocytes, with both direct and dye-reversal hybridizations. Agilent hybridization oven was set to 60 °C.

Fluorescent images were obtained using the G2565BA Microarray Scanner System (Agilent) with 100% laser power and 100% PMT settings and 16-bit TIFF images, one for each channel, were quantified using GenePix Pro 6.0 microarray analysis software (Molecular Devices). Mean foreground and background intensities were extracted from the red (Cy5) and green (Cy3) channels for every spot on the microarray. The background intensities are used to correct the foreground intensities for local variation on the array surface, resulting in corrected red and green intensities. Raw data were processed essentially as previously described^66^ using SAPO and CGEM alternative splicing analysis tools, obtaining Lowess normalized log_2_ ratios. General gene expression values represent the average of log_2_ ratios for all the probes of a locus. Statistical analyses were carried out with Linear Models for Microarray Data^67,68^. The background correction method used in the analysis was Normexp^69^. Locally weighted linear regression analysis was used as a normalization method^70^. Data has been deposited in NCBI’s Gene Expression Omnibus with accession number GSE83678.

### Whole genome human junction arrays

Affymetrix Human Junction Arrays (HJAY, catalog number GPL15106; see Supplementary Data 7) were hybridized by GenoSplice Technology according to Affymetrix (Santa Clara) labeling and hybridization recommendations. Total RNAs RIN values were between 9.8 and 10.0 (average: 9.98). Raw data are controlled with Expression console (Affymetrix). Affymetrix HJAY data set analysis was performed by GenoSplice technology. Data were normalized using quantile normalization. Background corrections were made with antigenomic probes and probes were selected according to their %GC, cross-hybridization status and potential overlap with repeat regions. Only probes targeting exons and exon–exon junctions annotated from FAST DB transcripts (release fastdb_2013_1) were selected. Only probes with a DABG *p*-value ≤ 0.05 in at least half of the arrays were considered for statistical analysis. Only genes expressed in at least one compared condition were analyzed. To be considered to be expressed, the DABG *p*-value was selected to be ≤0.05 for at least half of the gene probes. Student’s *t* test was performed to compare gene intensities between experimental conditions. Genes were considered significantly regulated when fold-change was ≥1.5 and *p*-value ≤ 0.05 (unadjusted *p*-value). Analysis at the splicing level was first performed taking into account only exon probes in order to potentially detect new alternative events that could be differentially regulated (i.e., without taking into account exon–exon junction probes). Analysis at the splicing level was also performed by taking into account exon–exon junction probes using the FAST DB splicing patterns annotation (i.e., for each gene, all possible splicing patterns were defined and analyzed). Expression and alternative splicing analyses were performed using unpaired Student’s *t* test on the splicing-index. Results were considered statistically significant for *p*-values ≤ 0.05 and fold-changes ≥ 1.5 for alternative splicing analysis; and *p*-values ≤ 0.05 and fold-changes ≥ 2.0 for expression analysis. Data have been deposited in NCBI’s Gene Expression Omnibus with accession number GSE83590.

### Isobaric tag for relative and absolute quantitation (iTRAQ)

Bioinformatic tools used for iTRAQ are summarized in Supplementary Data 7.

Sample preparation: Pellets obtained from control or CELF1 depleted SK-Mel-103 and UACC-62 cells were washed three times with cold PBS containing protease inhibitors (Halt Protease & Phosphatase inhibitor cocktail, EDTA-free) and then resuspended in 500 μL of ice-cold RIPA lysis buffer (150 mM NaCl, 1.0% NP-40, 0.5% sodium deoxycholate, 0.1% SDS, 1 mM EDTA, 1 mM EGTA, 50 mM Tris, pH 8.0 plus protease inhibitors) and 0.1% Benzonase Nuclease (Novagen). Samples were vortexed, sonicated, and clarified by centrifugation at 4 °C and 16,100 × *g* for 15 min. The supernatants containing the protein fraction were collected and cleaned-up by methanol-chloroform precipitation. Pellets were dissolved in 7 M urea 2 M tiourea. Protein concentration was determined using the Pierce 660 nm Protein Assay (Bio-Rad) using BSA as standard.

Protein digestion and labeling: Samples were digested using the filter aided sample preparation (FASP) method. Briefly, the equivalent to 100 μg of each sample was loaded on the filter, reduced with 10 mM DTT 1 h at 37 °C and alkylated using 55 mM iodoacetamide for 20 min in the dark. The excess of reduction and alkylation reagents was washed. The proteins were digested overnight using endoproteinase Lys-C (Wako) with 1:100 enzyme to protein ratio. Finally, trypsin (Promega) was added and samples were subjected to a second digestion for 6 h. Each tryptic digest was labeled according to the manufacturer’s instructions (Applied Biosystems) with one 4-plex isobaric amine-reactive tag per cell line. After 1 h incubation, labeled samples were pooled, and evaporated to dryness in a vacuum centrifuge. The iTRAQ sample was cleaned up using a Sep-Pak C18 cartridge for SPE (Waters Corp., Milford, MA). Eluted peptides were vacuum-dried and reconstituted in 8 M urea, 5% glycerol and 1% ampholytes pH 3–10 prior to electrofocusing.

OFFGEL fractionation: For pI-based peptide separation, we used the 3100 OFFGEL Fractionator system (Agilent Technologies, Böblingen, Germany) with a 24-well set-up. The IPG gel strips of 24 cm-long (GE Healthcare, München, Germany) with a 3–10 linear pH range were rehydrated for 15 min with the Peptide IPG Strip Rehydratation Solution according to the protocol of the manufacturer. Subsequently, 150 μL of sample was loaded in each well. Electrofocusing of the peptides was performed at 20 °C and 50 μA until the 50 kVh level was reached. After focusing, the 24 peptide fractions were withdrawn and the wells rinsed with 100 μL of a solution of 0.1%TFA. Rinsing solutions were pooled with their corresponding peptide fraction. All fractions were evaporated by centrifugation under vacuum. Solid phase extraction and salt removal was performed with home-made columns based on Stage Tips with C8 Empore Disks (3M, Minneapolis, MN) filled with R3 resin (Applied Biosystems). Eluates were evaporated to dryness and maintained at 4 °C. Just prior nano-LC, the fractions were resuspended in H_2_O with 0.1% (v/v) FA.

Peptide analysis by nanoLC–MS/MS: Digested samples were separated by on-line reversed-phase nanoscale capillary LC and analyzed by electrospray MS/MS. The experiments were performed on an Eksigent nano LC system (Eksigent technologies) coupled to an LTQ Orbitrap Velos mass spectrometer (Thermo Scientific, Bremen) equipped with a nanoelectrospray ion source (Proxeon Biosystems). Peptides were resuspended in 0.1% FA and loaded from a cooled nanoLC AS-2 autosampler (Eksigent). In order to pre-concentrate and desalt the samples before switching the pre-column in line with the separation column, 5 μL from each sample was loaded onto a reversed-phase ReproSil Pur C18-Aq 5 µm 0.3 × 10 mm trapping cartridge (SGE Analytical), and washed for 10 min at 2.5 μL/min with loading buffer (0.1% FA). The peptides were eluted from a RP ReproSil Pur C18-AQ 3 µm 250 × 0.075 mm (Dr. Maisch GmbH, Ammerbuch-Entringen) by application of a binary gradient consisting of 4% ACN in 0.1% FA (buffer A) and 100% ACN in 0.1%FA (buffer B), with a flow rate of 300 nL/min. Peptides were separated using the following gradient: 0–5 min 4% B, 5–150 min 40% B, and 150–165 min 98% B. The column was operated at a constant temperature of 40 °C. The LTQ Orbitrap Velos was operated in positive ionization mode. The MS survey scan was performed in the FT analyzer scanning a window between 250 and 1750 *m*/*z*. The resolution was set to 30,000 FWHM at *m/z* 400. The *m*/*z* values triggering MS/MS with a repeat count of 1 were put on an exclusion list for 40 s. The minimum MS signal for triggering MS/MS was set to 1000 counts. The lock mass option was enabled for both MS and MS/MS mode and the polydimethylcyclosiloxane ions (PDMS, protonated (Si(CH_3_)_2_O)_6_; *m*/*z* 445.120025) were used for internal recalibration of the mass spectra. In all cases, one microscan was recorded. For the HCD, up to the 15 most abundant isotope patterns with charge ≥2 from the survey scan were selected with an isolation window of 2*m*/*z* and fragmented in the C-trap collision cell. Normalized collision energy was set to 42.5, the *Q* value to 0.25 and an activation time to 0.10 ms. Waveform filter was activated. The resulting fragments were detected in the Orbitrap system with a resolution of 7500 FWHM at *m/z* 400. The maximum ion injection times for the survey scan and the MS/MS scans were 500 and 250 ms respectively and the ion target values were set to 1E6 and 7E4, respectively for each scan mode.

Data analysis: Raw files were processed using the Proteome Discoverer 1.4 software suite (Thermo Scientific). The fragmentation spectra were searched against the UniProtKB/Swiss-Prot human database (December 2013, 20,584 sequences plus a list of common contaminants) using Sequest-HT as the search engine with the precursor and fragment mass tolerances set to 25 ppm and 0.025 Da, respectively, and with up to two missed cleavages. Lysine and peptide N-termini labeling with iTRAQ-4plex reagent as well as carbamidomethylation of cysteine were considered as fixed modifications, while oxidation of methionine was chosen as variable modification for database searching. Peptide identification was validated with Percolator and filtered at 1% false discovery rate (FDR) using the target-decoy strategy. Further filters included: rank 1 peptides and ≥6 amino acids length. The PSM table was exported as.csv and imported into Isobar for statistical analysis. Proteins with a *p*-value of less than 0.05 and with a log2 ratio at least >0.3 or <−0.3 were classified as up- or downregulated, respectively. Protein classification enrichment analysis (molecular function, biological process and protein class) was performed by PANTHER software, using the entire list of identified proteins as the reference data set to analyze the regulated proteins. A ranked protein list using the calculated averaged ratio of the two cell lines was imported into GSEA for ontology enrichment. Hallmarks and Canonical Pathways gene sets (http://www.broadinstitute.org/gsea/msigdb, mSigDb v5.0) were tested. Proteomics and transcriptomics data were put together using Microsoft Excel. The mass spectrometry proteomics data have been deposited to the ProteomeXchange Consortium via the PRIDE partner repository with the data set identifier PXD003112.

### DEK cDNA microarray

SK-Mel-103 cell line was grown in DMEM medium supplemented with 10% fetal bovine serum, 1% penicillin and streptomycin, and maintained at 37 °C in humidified 5% CO_2_ atmosphere. Cells were plated at 70% confluence and infected with lentivirus either KH1-GFP scramble control or KH1-GFP-shDEK(2) virus. At day 2 cells were selected with puromycin selection. At day 4.5 cells were harvested. RNA was isolated by Qiagen RNeasy Kit following manufacturer instructions. In total, 1 µg of RNA was labeled by commercial “Two-Color Microarray-Based Gene Expression Analysis (Quick Amp Labeling)” kit following manufacturer instructions. Amplification was performed by RNA polymerases (Agilent manual G4140-90050 Ver. 5.7 March 2008). Briefly, MMLV-RT retrotranscription of samples from a T7 promoter primer was followed by a T7 RNA pol catalyzed in vitro transcription reaction in the presence of either Cy3-CTP or Cy5-CTP fluorophores. Labeled samples were purified with silica-based RNeasy spin columns (Qiagen). 825 ng of sample in 100 μl was used in SureHyb hybridization chamber (Agilent) for hybridization at 65 °C for 17 h. Human WHG 4X44K (Agilent—GPL6480) platform was used for microarray. Array was scanned on an G2565C DNA microarray scanner (Agilent). Hybridized microarray images were analyzed by Agilent Feature Extraction Software (ver. 10.1), which performed feature quantification, background subtraction (by spatial detrending) and dye bias normalization (after bias detection by linear and Lowess curve fitting methods). Data were normalized using loess within array normalization and quantiles for between-arrays normalization. Normexp method was applied for background correction. Data have been deposited in NCBI’s Gene Expression Omnibus with accession number GSE83614.

### Whole genome GRE motif search

In order to search for GRE motifs (UGUGUGUGUGU and UGUUUGUUUGU) in the whole genome, the EMBOSS fuzznuc program (http://emboss.sourceforge.net/apps/cvs/emboss/apps/fuzznuc.html) was used allowing for two mismatches. Obtained genomic coordinates were intersected with the 3′ UTR coordinates of the human genes (Ensembl75/GRCh37) using BEDtools, requiring the full motif to be contained within the 3′ UTR sequence of the genes.

### Comparative analyses of RIP-Seq analyses in melanoma (this paper) vs. other tumor types (ENCODE)

BAM files containing the read alignments of the samples belonging to two CELF1 RNA-seq experiments were downloaded from ENCODE database repertoire: K562 shCELF1 RNA-seq (ENCSR605MFS) and shCELF1 HepG2 RNA-seq (ENCSR695XOD). For each experiment, the files corresponding to two replicates of the shCELF1 and the non-specific target control (shControl) were downloaded. Transcripts quantification and differential expression were calculated with Cufflinks 2.2.1, using the human GRCh37/hg19 transcript annotations from https://ccb.jhu.edu/software/tophat/igenomes.shtml. PANTHER was used for the identification of biological functions specifically enriched in set of genes which were common to be downregulated upon CELF1 and DEK depletion in melanoma cells (adjusted *p*-value<0.05).

### GSEA, networks, heatmaps, and Venn diagrams

Significantly enriched (*p* ≤ 0.05) GO Biological Processes (database 02.10.2015) between melanoma cells and primary melanocytes were identified by using Cytoscape v3.2.1 and the ClueGO v2.1.7 plug-in. GSEA was performed using annotations from the Reactome pathways. Genes were ranked using the *t*-statistic. After Kolmogorov–Smirnoff correction for multiple testing, only those pathways bearing a FDR < 0.25 were considered significant. Heatmap and correlation graphs for RNA and protein levels were created by Perseus v1.5.1.6. Protein networks were created by using Search Tool for the Retrieval of Interacting Genes/Proteins v10 (STRING) and Cytoscape v3.2.1. Venn diagrams were created by using online tools InteractiVenn and jvenn (see Supplementary Data 7 for references on the bioanalytic tools used in this study).

### Additional statistical analyses

CELF1 protein expression in human benign and malignant melanocytic lesions and cell proliferation curves were evaluated by two-tailed unpaired Student's *t* test. Comparative analyses of mRNA expression for *CELF1* and *DEK*, as well as the RBPs and the chromosome 11p11.2-mapping genes in this study were performed from data extracted from TCGA (either melanoma or the indicated tumor data sets). For analyses of changes in gene expression during melanoma progression, specimens were separated as a function of tumor stage (i.e., primary vs. metastatic cases; analyzed by unpaired Student's *t*-test), or anatomical location (primary cutaneous lesions or skin, lymph node or visceral metastasis as indicated). Overall Survival curves were estimated with Kaplan-Meier product-limit method for patients stratified in two categories according to mRNA expression: below or above the median for the gene set analyzed (labeled as “low” or “high” in the plots, respectively). Survival curves were compared using log-rank (Mantel Cox) or Gehan–Breslow–Wilcoxon tests as indicated, to monitor correlations to long-term vs. short term survival. *p* < 0.05 was considered significant. CELF1 and DEK co-expression in human melanoma specimens was evaluated by Pearson test. *r* stands for Pearson correlation value. Statistical analyses of RNA-Seq, iTRAQ, splicing sensitive arrays, HJAY, and RIP-Seq are indicated above.

### Data availability

Data sets generated for CELF in melanoma cells are as follows: RNA-Seq (GSE88741), RIP-seq (GSE83231), HJAY (GSE83590), iTRAQ (PXD003112), splicing-arrays (GSE83678), and DEK cDNA arrays (GSE83614). For expression analyses of CELF1 function in other tumor types, RIP+Microarray data with identifiers ENCSR000AYU (K562) and ENCSR000AYA (GM12878), and transcriptomic data with identifiers ENCSR605MFS (K562) and ENCSR695XOD (HepG2) were extracted from the ENCODE database.

## Electronic supplementary material

Supplementary Information

Description of Additional Supplementary Files

Supplementary Data 1

Supplementary Data 2

Supplementary Data 3

Supplementary Data 4

Supplementary Data 5

Supplementary Data 6

Supplementary Data 7
