# Supplementary material for: Systems analysis identifies melanoma-enriched pro-oncogenic networks controlled by the RNA binding protein CELF1
Source: Nat Commun. 2017 Dec 21;8:2249. doi: 10.1038/s41467-017-02353-y (PMC5740069; doi:10.1038/s41467-017-02353-y)
Supplement: Supplementary file 1 — Supplementary Information [file 41467_2017_2353_MOESM1_ESM.pdf]

Supplementary Figure 1

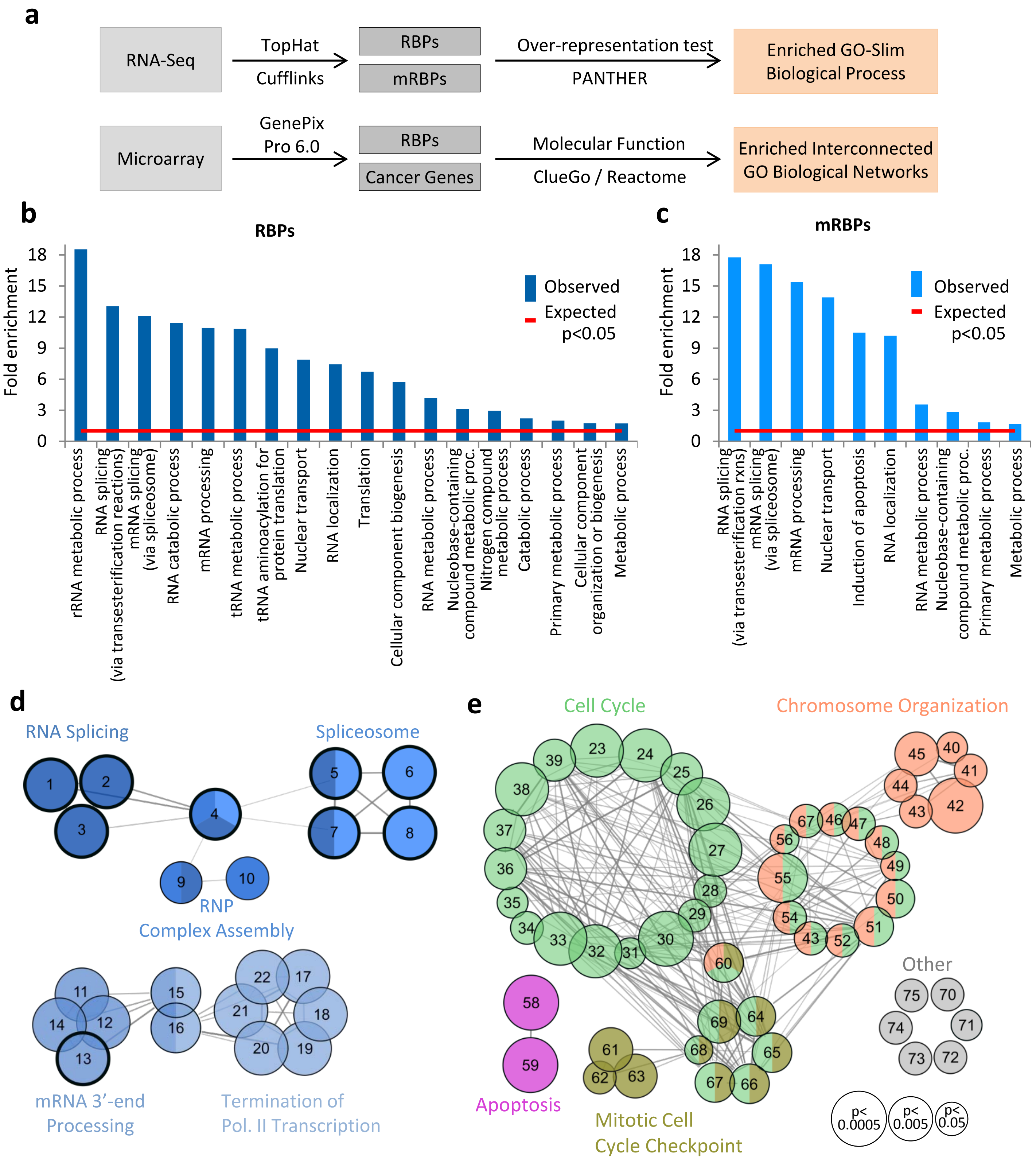

Supplementary Figure 1 | The RBP landscape in melanoma cells. a, Workflow of enrichment tests performed to assess gene expression and splicing-associated changes of RBPs in melanoma. **b-c,** Identification of biological processes associated to RBPs **(b)** and mRBPs **(c)** found by RNA-Seq to be significantly deregulated in melanoma cells compared to normal melanocytes (*p*<0.05). Data were analyzed using the Statistical Overrepresentation Test on the PANTHER database. **d,** Interaction networks of RNA-associated functions found by RBP-enriched arrays to be deregulated in SK-Mel-19 and SK-Mel-103 cell lines compared to normal melanocytes. The networks were identified using Cytoscape and ClueGo. Bold-bordered terms refer to GO terms containing CELF1 (see Supplementary Table 3 for gene lists in each gene cluster). Size of the nodes reflects the statistical significance of the terms as indicated: *p*<0.0005, *p*<0.005 or *p*<0.05. **e,** Network analyses as in (**d**), but for cancer-associated genes.

Supplementary Figure 2

a

Gene	#	Tumorigenic Role	mRNA Stability	Translation	Splicing	Melanoma related function (ref.)
CELF1	9	P	+	+	+	-
KHDRBS1	6	P	+	+	+	-
FUBP1	0	P	+	+	+	-
SRSF2	15	P	+	-	+	-
SNRPB	10	P	+	-	+	-
TRA2B	8	P	+	-	+	-
LSM1	4	P	+	-	+	-
SRSF9	18	P	-	+	+	-
HNRNPA1	9	P	-	+	+	-
CDK9	5	P	-	+	+	-
DDX39B	12	P	-	-	+	-
SRRM1	11	P	-	-	+	-
RBM17	5	P	-	-	+	-
DDX46	4	P	-	-	+	-
EFTUD2	4	P	-	-	+	-
NOP56	2	P	-	-	+	-
BUB3	0	P	-	-	+	-
HNRNPK	4	P/A	+	+	+	-
PRPF4B	4	P/A	-	-	+	-
MBNL1	12	A	+	+	+	-
CELF2	8	A	+	+	+	-
PABPC4	1	A	+	+	+	-
SF3A3	8	A	-	-	+	-
DHX15	3	A	-	-	+	-
U2AF1	11	N/A	+	-	+	-
LSM7	4	N/A	+	-	+	-
HNRNPDL	1	N/A	+	-	+	-
STAU1	1	N/A	+	-	+	-
SRSF7	14	N/A	-	-	+	-
PSIP1	8	N/A	-	-	+	-
SNRPD2	8	N/A	-	-	+	-
SNRNP200	7	N/A	-	-	+	-
NUDT21	6	N/A	-	-	+	-
NHP2L1	5	N/A	-	-	+	-
SF3B6	4	N/A	-	-	+	-
SNRPA1	4	N/A	-	-	+	-
SMN2	2	N/A	-	-	+	-
FNBP1	0	N/A	-	-	+	-
RBM7	0	N/A	-	-	+	-
SSRP1	0	P	-	-	+	-
SUPT16H	0	P	-	-	+	-
PTBP1	9	P	+	+	+	Marzese et al. 2015
MAGOH	15	P	-	-	+	Silver et al. 2013
SRSF3	11	P	-	+	+	Dewaele et al. 2016
EIF6	5	P	-	+	+	Pinzaglia et al.2015
C1QBP	9	P	-	-	+	Prakash et al. 2011
SNRPD1	8	P	-	-	+	Quidville et al. 2013
DEK	0	P	-	-	+	Khodadoust et al. 2009
DYRK1A	9	P/A	+	-	+	de Wit et al. 2002
SNRPE	11	P/A	-	-	+	Quidville et al. 2013

b

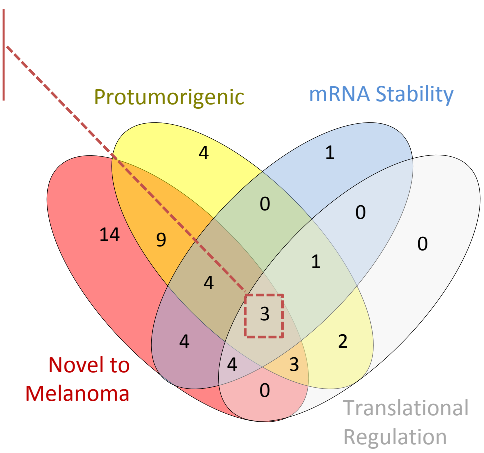

Supplementary Figure 2 | RBP-enriched arrays identify CELF1 as a novel upregulated gene in melanoma. a, Genes upregulated (red) or downregulated (green) in SK-Mel-19 or SK-Mel-103 cell lines with respect to primary melanocytes identified by the RBP-enriched microarray described in the text. Indicated are the number of GO terms (#) that contain the genes listed. Functions of the genes are labeled as follows: P: protumorigenic; A: antitumorigenic; P/A: pro/antitumorigenic, N/A: not available). The "+" and "-" symbols refer to reported or not reported functions), respectively. References for those genes analyzed in melanoma are also indicated. **b**, Venn diagram showing the overlap of genes with the indicated features. mRNA expression levels and prognostic value of genes with functions similar to CELF1 (highlighted in light blue) are shown in **Supplementary Figure 3**.

Supplementary Figure 3

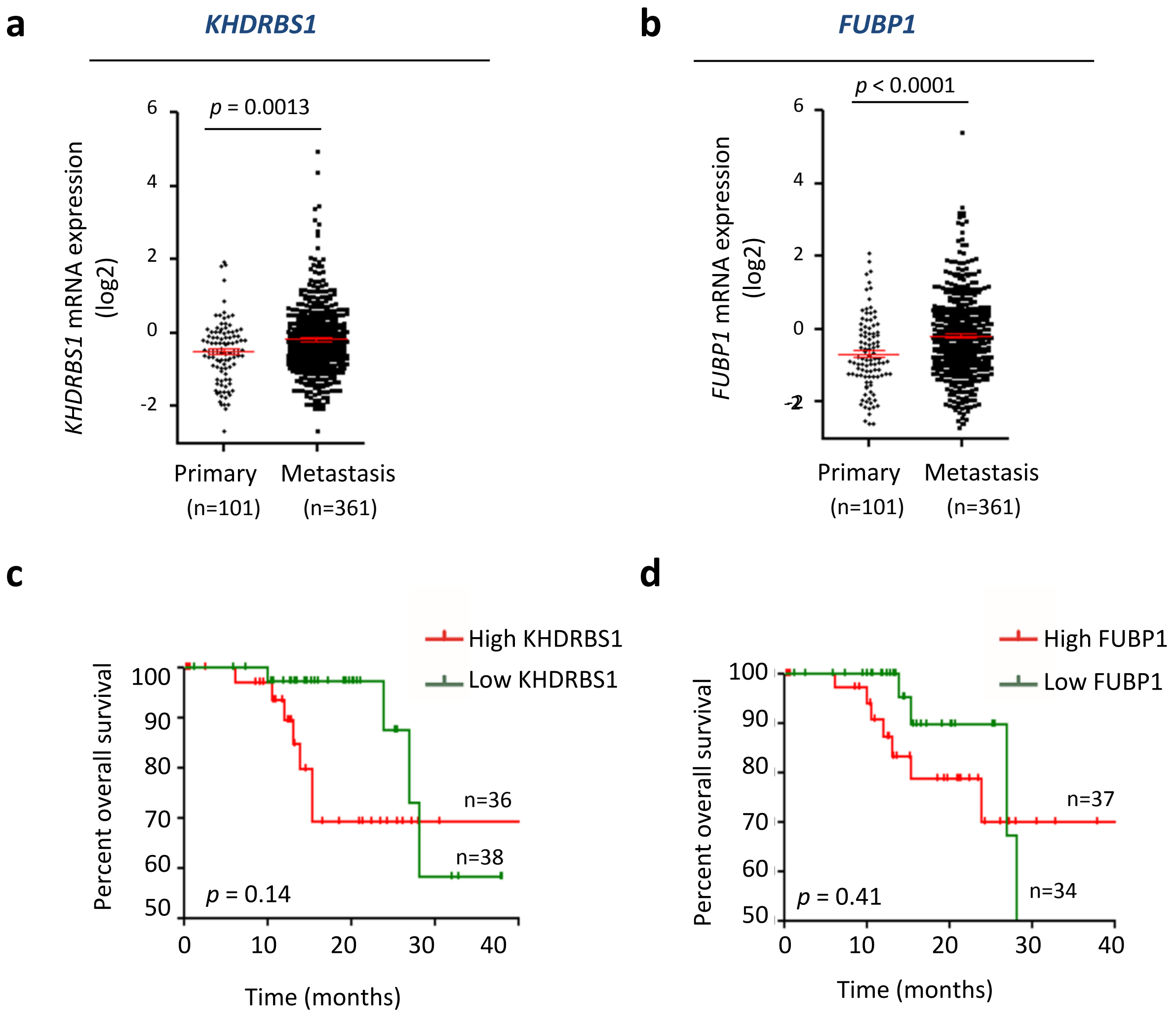

Supplementary Figure 3 | mRNA expression and prognostic value of RBPs sharing functional features with CELF1. a-b, *FUBP1* and *KHDRBS1* mRNA expression levels of melanoma samples grouped by disease stage plotted on dot plot (data retrieved from TCGA database). **c-d,** Overall survival of melanoma patients (data retrieved from TCGA database) plotted in Kaplan-Meier survival plot for *FUBP1* and *KHDRBS1* mRNA expression levels, respectively. “High” and “low” refers to mRNA values above or below the median expression for all specimens in the TCGA melanoma dataset. Indicated are log-rank *p*-values. *P*-values for Gehan-Breslow-Wilcoxon test are 0.045 (*KHDRBS1*) and 0.09 (*FUBP1*).

Supplementary Figure 4

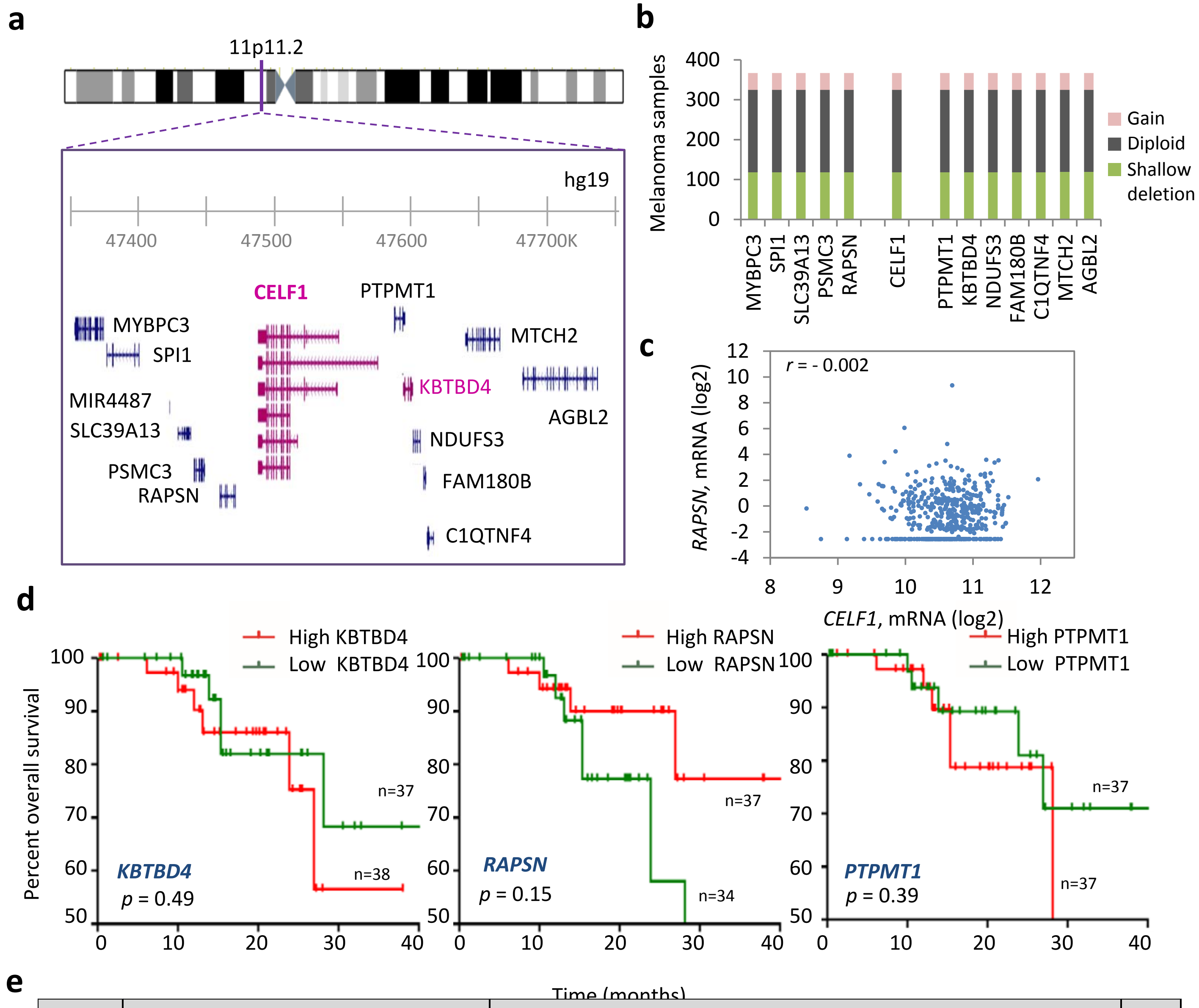

e

SYMBOL	GENE NAME	CANCER-RELATED FUNCTIONS	*r*
MYBPC3	Myosin-Binding Protein C, Cardiac-Type	No role described in cancer	0.002
SPI1	SFFV Proviral Integration Oncogene spi1	Transcription factor. Oncogenic functions in hematopoyetic tumors	-0.148
MIR4487	Micro RNA 4487	No direct role in cancer described. Can control ULK	0
SLC39A13	Solute carrier family 39 member 13	No role in cancer. Zinc transporter.	-0.041
PSMC3	Proteasome 26S Subunit, ATPase 3	Not direct role in cancer. Controls the half life of FRA1 in tumor cells	-0.046
RAPSN	Receptor Associated Protein Of The Synapse	Hypomethylated in DNA of breast cancer patients	-0.002
CELF1	CUGBP Elav-Like Family Member 1	Upregulated in various tumor types. Not conserved downstream targets	1
PTPMT1	Protein Tyrosine Phosphatase, Mitochondrial 1	Induced by HIF-2α. Regulates the proliferation and glucose metabolism in	0.063
**KBTBD4**	Kelch Repeat And BTB Domain Containing 4	No roles described in cancer, but in-frame mutations in a subset of medulloblastomas	**0.541**
NDUFS3	NADH:Ubiquinone Oxidoreductase Core	Tumor supressor? Reduced expression of NDUFS3 in serous ovarian cancer	-0.117
FAM180B	Family With Sequence Similarity 180 Member B	No functional annotation	-0.159
C1QTNF4	C1q And TNF Related 4	Possible cytokine. No fuctional characterization. No roles in melanoma	0.025
MTCH2	Mitochondrial Carrier 2	Proposed as tumor suppressor by interfering with MET HGF/SF	0.144
AGBL2	ATP/GTP Binding Protein Like 2	Proposed indicator of bad prognosis in gastric cancer. No mechanism known	0.275

Supplementary Figure 4 | Differential expression and function of genes flanking the *CELF1* locus. a, Schematic of the chromosomal band 11p11.2 spanning an area of 400 Kpb centered on *CELF1*. Genes with Pearson correlation *r* >0.45 with respect to CELF1 mRNA are indicated in red. **b,** Genomic status of indicated genes in melanoma patients (data obtained from TCGA, n=479). **c,** *RAPSN* vs. *CELF1* mRNA levels in melanoma patients (data obtained from TCGA, n=479) shown on a correlation scatter plot. *r*: Pearson correlation coefficient. **d,** Overall survival of melanoma patients plotted in a Kaplan-Meier plot for RAPSN, PTPMT1 and KBTBD4, respectively. High/low expression refers to the median expression of all data in the TCGA melanoma dataset. *P*-values, Log-rank test. **e,** List of cancer related functions of *CELF1* neighboring genes on chromosome 11p11.2. r indicates the Pearson correlation of mRNA expression each gene with respect to *CELF1* in melanoma (data retrieved from TCGA).

Supplementary Figure 5

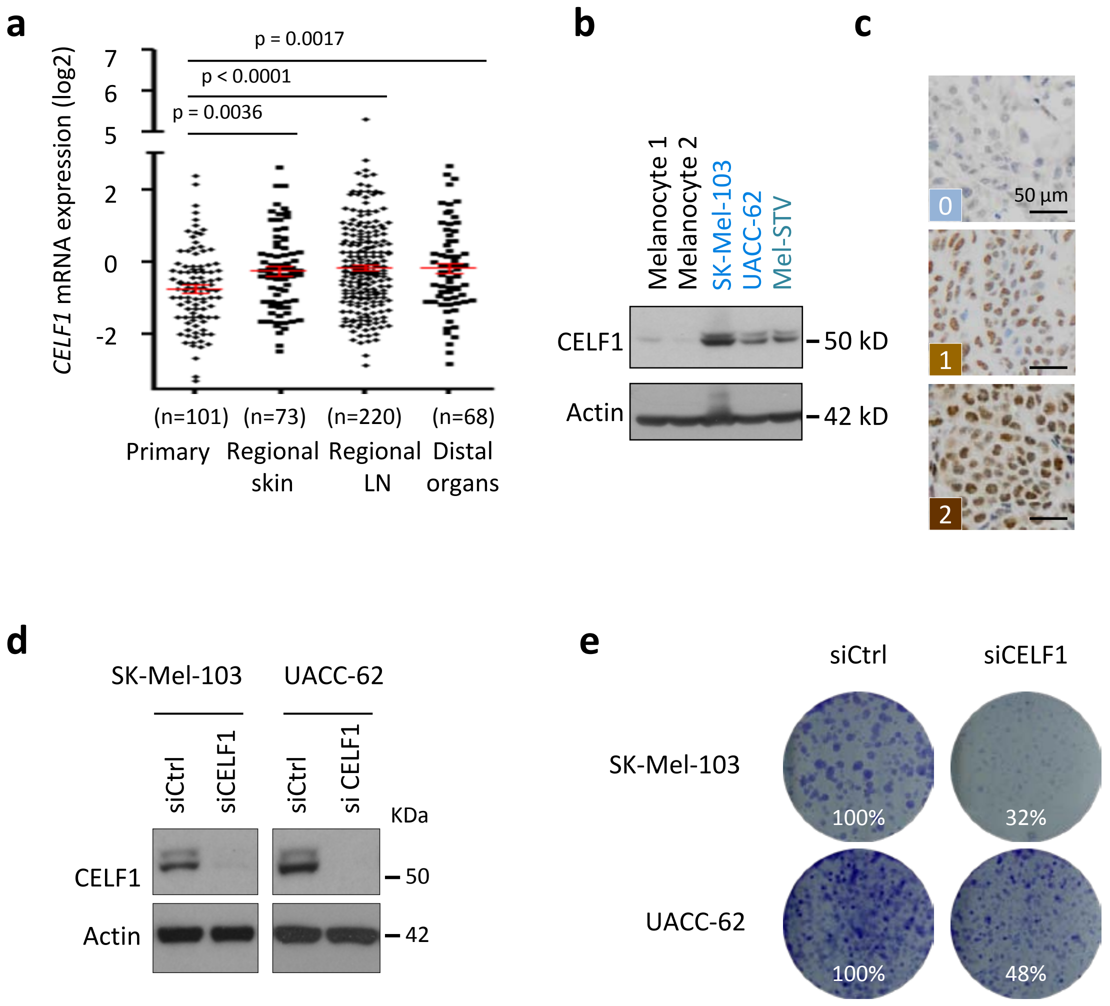

Supplementary Figure 5 | CELF1 is an early-induced melanoma gene. a, *CELF1* mRNA expression levels of melanoma samples grouped by disease stage and represented on a dot plot (data retrieved from the TCGA database). **b,** CELF1 protein expression in immortalized Mel-STV melanocytes shown in comparison to normal melanocytes and two melanoma cell lines. **c,** Representative examples of paraffin-embedded melanomas stained for CELF1 (brown) to illustrate negative, intermediate and high-expression levels (i.e. scoring 0, 1, 2, respectively). **d,** CELF1 depletion by siRNA in the indicated cell lines, defined by protein immunoblotting, with siRNA controls as reference. **e,** Reduced colony formation ability of siCELF1 transfected cells determined by crystal violet 12 days after cell seeding (data represented as percentages referred to siControl transfected cells).

Supplementary Figure 6

a

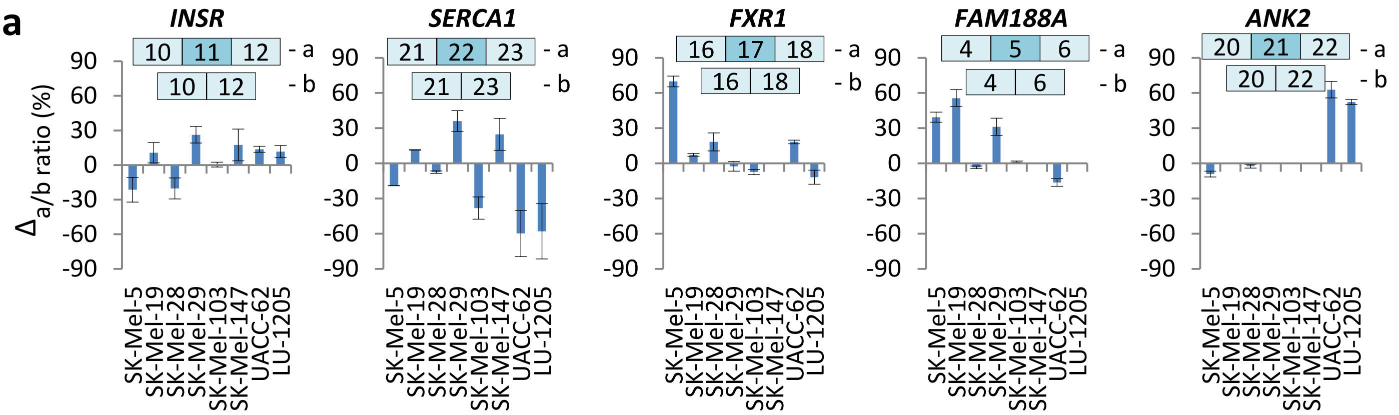

b

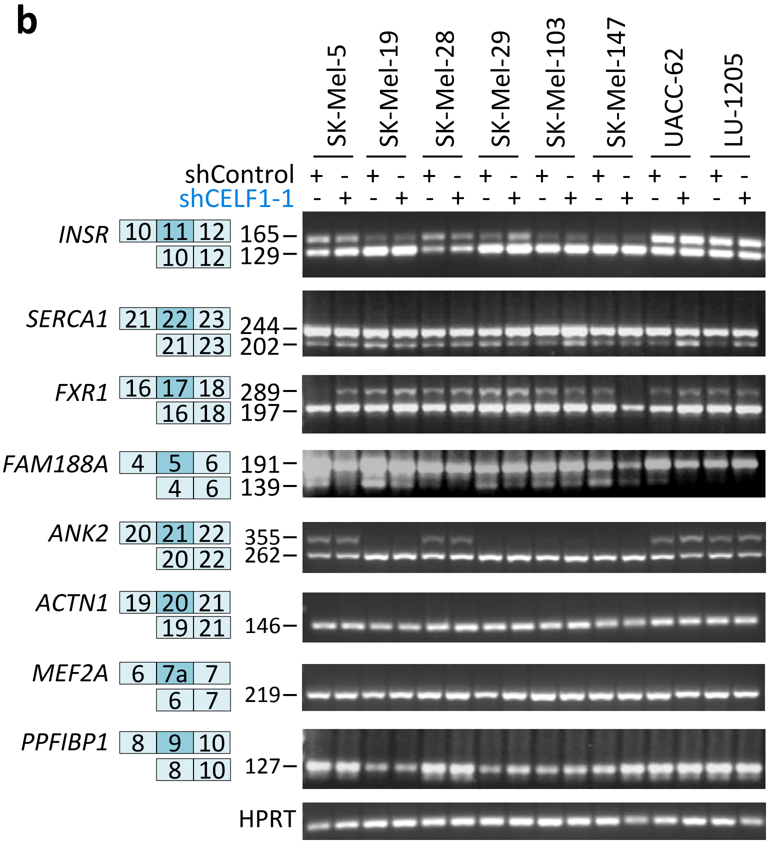

d

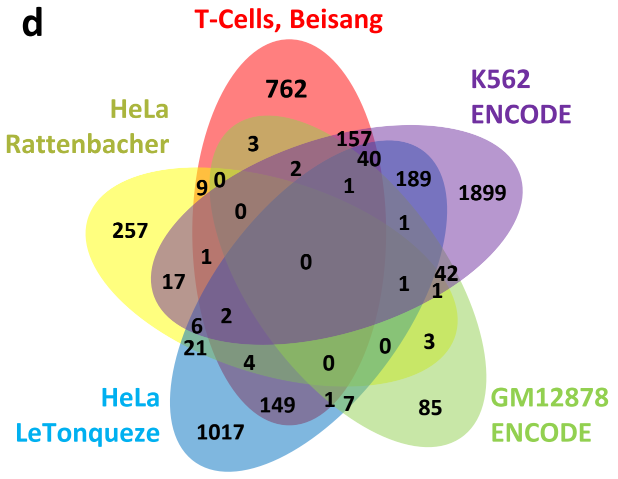

%	GM12878 ENCODE	K562 ENCODE	T-Cells Beisang	HeLa LeTon.
HeLa_Ratten.	1.08	1.06	1.11	1.82
HeLa_LeTon.	0.64	6.50	7.85	
T-Cells_Beisang	0.55	6.18		
K562_ENCODE	1.95			

c

Cell line	shRNA	PSI ± SEM				
		INSR	SERCA1	FXR1	FAM188A	ANK2
SK-Mel-5	shControl	47.1 ± 1.14	69.8 ± 12.5	20.4 ± 5.83	65.6 ± 1.51	46.3 ± 11.3
	shCELF1-1	41.0 ± 4.42	65.6 ± 13.5	29.9 ± 7.05	72.6 ± 1.95	44.0 ± 10.6
SK-Mel-19	shControl	28.2 ± 9.08	62.3 ± 13.6	34.0 ± 5.17	61.6 ± 2.36	n.d.
	shCELF1-1	29.9 ± 7.84	64.6 ± 13.3	35.5 ± 5.03	71.3 ± 1.09	n.d.
SK-Mel-28	shControl	48.7 ± 1.77	66.7 ± 13.8	31.3 ± 6.45	76.5 ± 4.90	34.0 ± 14.7
	shCELF1-1	43.0 ± 4.54	65.2 ± 13.9	34.8 ± 5.37	75.9 ± 4.78	33.5 ± 14.9
SK-Mel-29	shControl	31.2 ± 7.51	63.1 ± 12.6	34.9 ± 2.12	63.5 ± 0.94	n.d.
	shCELF1-1	36.2 ± 6.82	69.1 ± 12.7	34.3 ± 1.15	69.5 ± 0.31	n.d.
SK-Mel-103	shControl	29.3 ± 8.34	74.2 ± 13.4	39.9 ± 13.4	69.0 ± 0.94	n.d.
	shCELF1-1	29.4 ± 8.77	65.7 ± 13.1	38.3 ± 13.7	69.2 ± 1.08	n.d.
SK-Mel-147	shControl	25.9 ± 9.95	70.6 ± 13.5	n.d.	n.d.	n.d.
	shCELF1-1	28.4 ± 8.37	73.8 ± 14.1	n.d.	n.d.	n.d.
UACC-62	shControl	48.0 ± 0.03	75.7 ± 15.4	29.1 ± 9.76	76.0 ± 4.23	36.3 ± 13.7
	shCELF1-1	51.2 ± 0.53	57.7 ± 10.2	32.4 ± 10.1	72.5 ± 5.38	47.0 ± 13.9
LU-1205	shControl	50.1 ± 1.86	75.2 ± 20.1	24.5 ± 3.46	n.d.	37.7 ± 14.3
	shCELF1-1	52.8 ± 0.68	61.8 ± 17.2	22.4 ± 4.40	n.d.	47.0 ± 15.0

Supplementary Figure 6 | Known CELF1 targets are not shared with melanoma cells. a, Ratio of alternatively spliced variants of the indicated genes depicted as their expression ratio in CELF1 shRNA vs. shControl transduced cells. Data were generated by semi-quantitative PCRs. Error bars correspond to SEM of two experiments. **b,** Analysis of gene isoforms by agarose gel electrophoresis after semi-qRT-PCR as in (**a**). **c,** Quantification of PSI ± SEM of three experiments as in (b). n.d.: non-determined. **d,** Overlap of CELF1-bound transcripts in the indicated cell types and studies. The table summarizes the corresponding overlaps as percentages.

Supplementary Figure 7

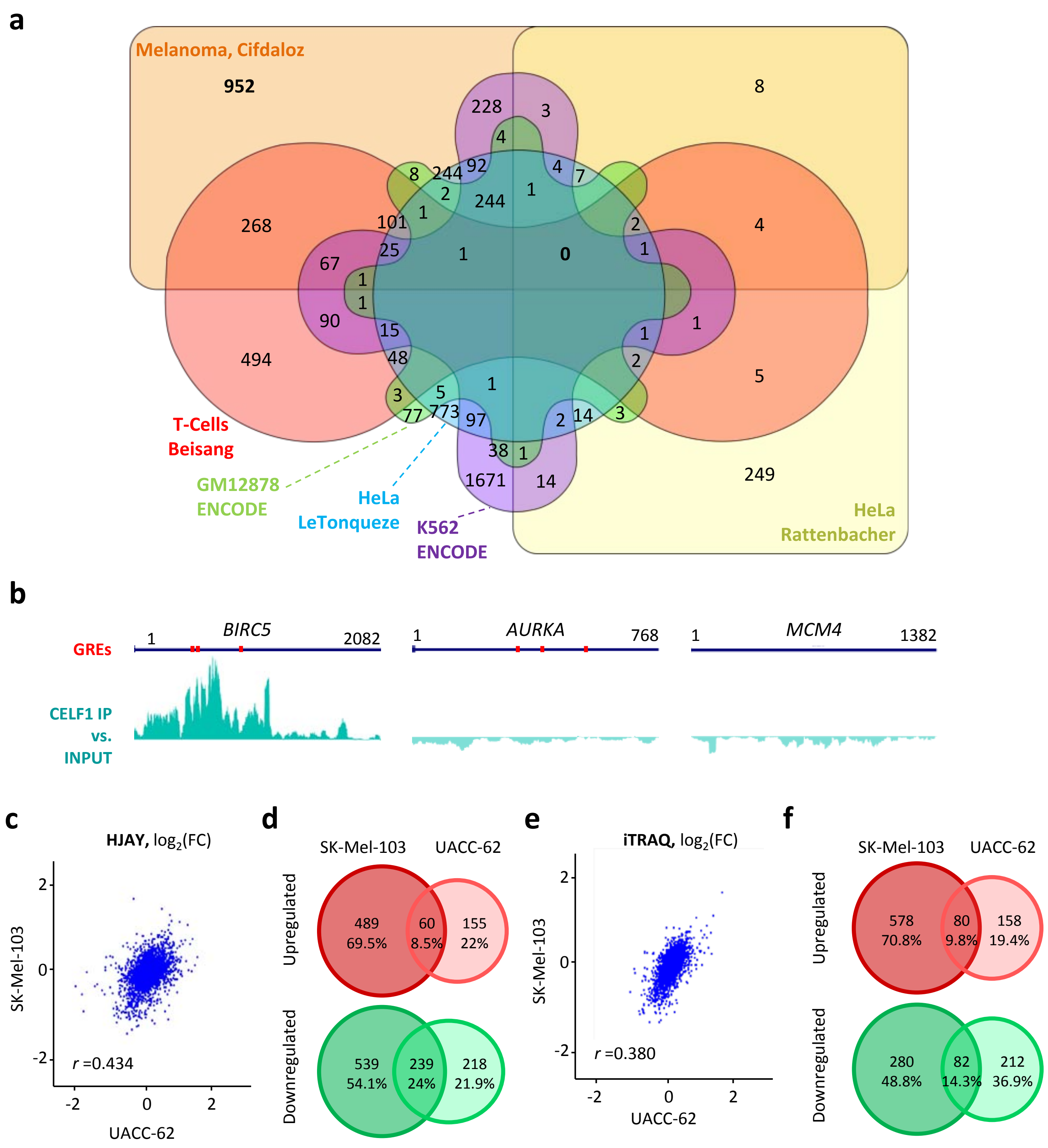

Supplementary Figure 7 | Minimal conservation in CELF1-bound transcripts across tumor types. a, Venn diagram depict the overlap of CELF1-binding targets in the indicated cell types and studies. For details, see Fig. 3a and Supplementary Table 4. **b,** Histogram of peak calling from RIP-Seq data corresponding to CELF1 binding at the 3'UTR of the *BIRC5, AURKA and MCM4* mRNAs, represented with respect to input controls. GREs are marked in red. **c,** Scatter plot showing the correlation of global RNA changes in SK-Mel-103 and UACC-62 cell lines upon CELF1 depletion identified by HJAY. r: Pearson correlation coefficient. **d,** Venn diagrams showing the corresponding overlap in up- and downregulated genes of panel **c**. **e,** Correlation of global protein changes in SK-Mel-103 and UACC-62 cell lines upon CELF1 depletion identified iTRAQ, with Venn diagrams the corresponding overlaps in panel (**f**).

Supplementary Figure 8

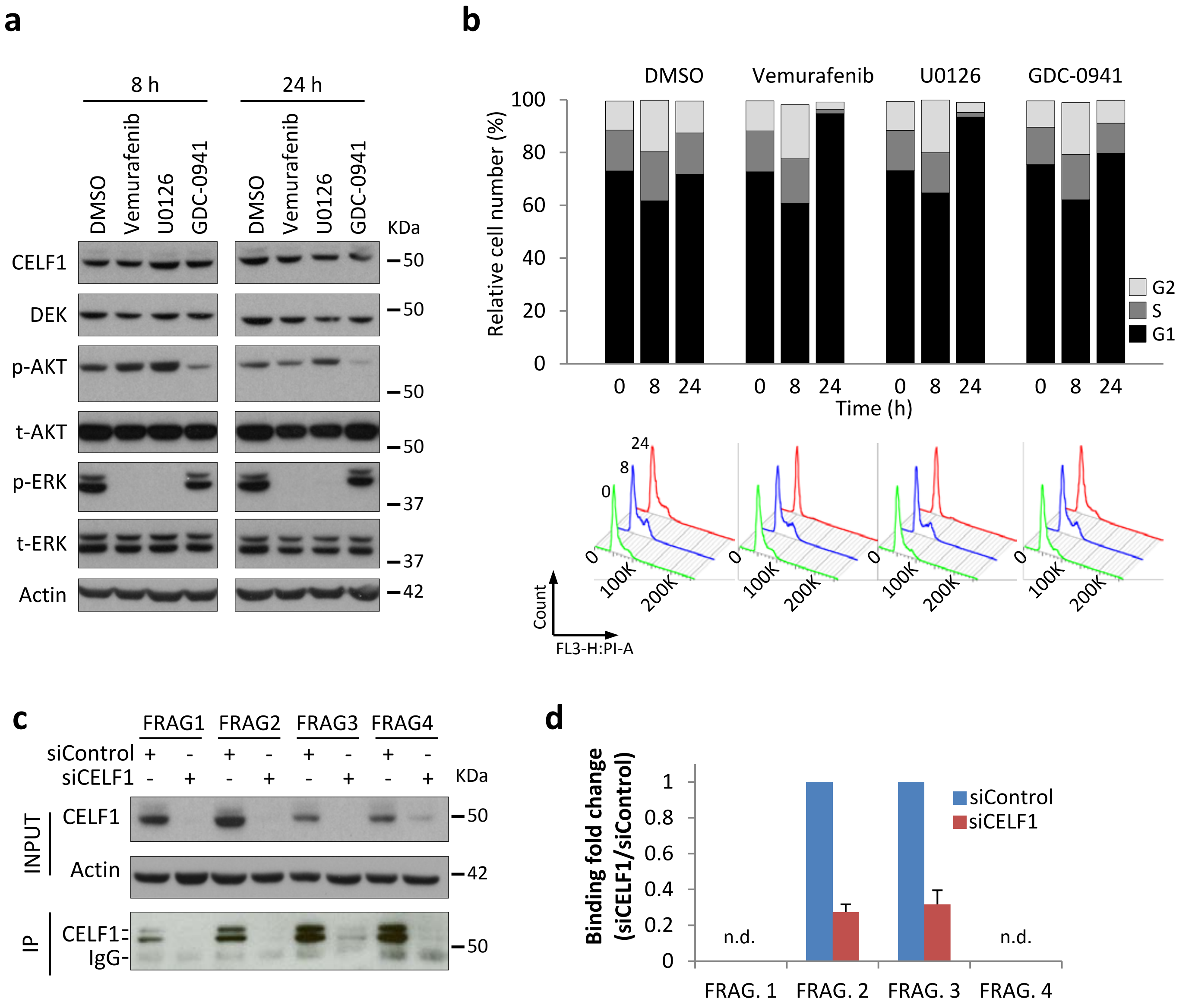

Supplementary Figure 8 | CELF1-mediated regulation of DEK is independent of cell cycle arrest. **a,** CELF1 and DEK protein levels in UACC-62 treated with indicated inhibitors as visualized by protein immunoblotting. Total ad phosphorylated AKT and ERK are included as controls for inhibition efficiency. **b,** Cell cycle profiles UACC-62 cells treated with indicated drugs defined by BrdU incorporation. DMSO is used as control. **c,** Input and immunoprecipitated CELF1 levels in UACC-62 expressing the indicated *DEK* 3'UTR fragments. As a control for specificity of the CELF1 antibodies, similar precipitations were performed in cells expressing CELF1 siRNA. **d,** Quantification of *DEK* 3'UTR fragments determined by semi-qRT-PCR after CELF1 pulldown (blots on Fig. 6k). Errors correspond to SEM of two independent experiments in triplicate.

Supplementary Figure 9

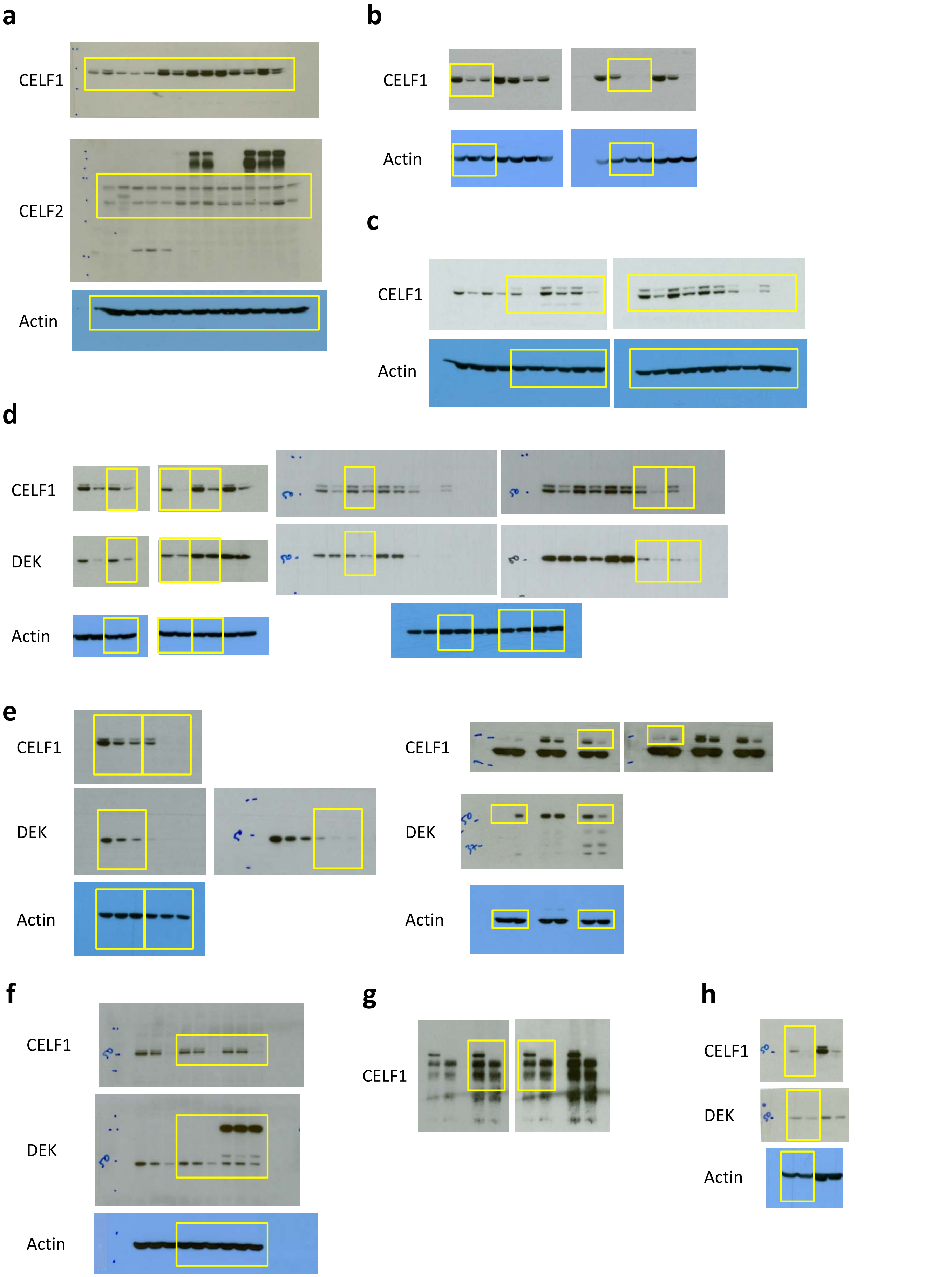

Supplementary Figure 9 | Uncropped western blot images corresponding to Figure 1e (a), Figure 2a (b), Figure 2e (c), Figure 6a (d), Figure 6b (e), Figure 6c (f), Figure 6i (g) and Figure 6m (h).

Supplementary Figure 10

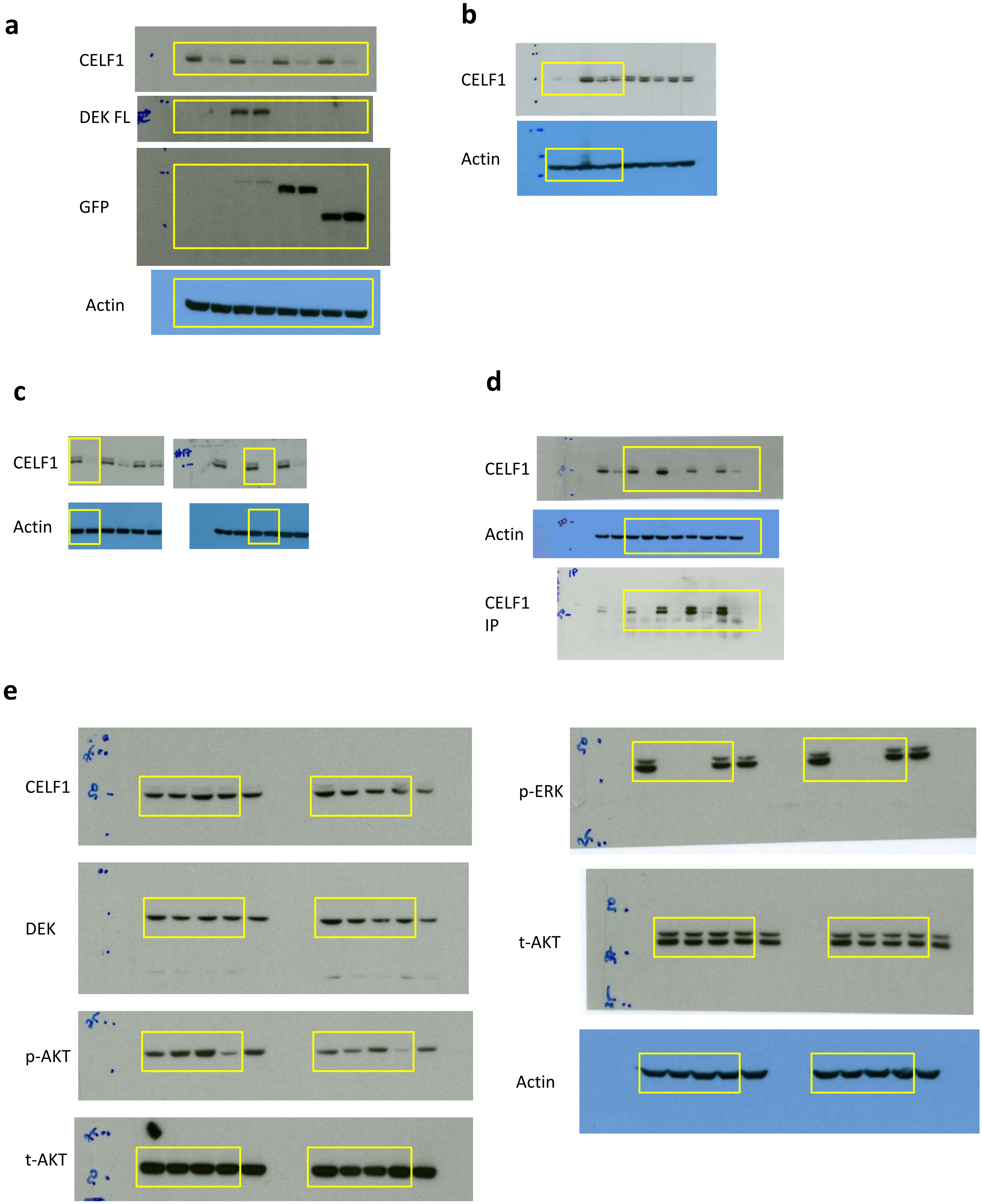

Supplementary Figure 10 | Uncropped western blot images corresponding to Figure 6g (a), Supplementary Figure 5b (b), Supplementary Figure 5d (c), Supplementary Figure 8c (d) and Supplementary Figure 8a (e).

CELL LINE	SOURCE	BRAF	NRAS	PTEN*	TP53	DEK*
SK-Mel-5	MSKCC cell bank	V600E	WT	+	WT^R	++
SK-Mel-19	MSKCC cell bank	V600E	WT	+	WT	++
SK-Mel-28	MSKCC cell bank	V600E	WT	+	R273H	++
SK-Mel-29	MSKCC cell bank	V600E	WT	-	WT	++
SK-Mel-103	MSKCC cell bank	WT	Q61R	+	WT^R	+++
SK-Mel-147	MSKCC cell bank	WT	Q61R	+	WT^R	++
G-361	MSKCC cell bank	WT/V600E	WT	-	WT^R	+
UACC-62	MSKCC cell bank	V600E	WT	-	WT	+
WM-1366	Wistar Institute cell bank	WT	Q61R	nd	nd	+++
LU-1205	Wistar Institute cell bank	V600E	WT	nd	nd	+

Supplementary Table 1. Human melanoma cell lines used in this study. (*:levels of the indicated proteins defined by immunoblotting; WT: wild type p53; ^{R}: samples with p53 polymorphism P72R.nd: not determined.

RNA-Seq				mRNA Expression in TCGA Melanoma				Overall Survival in TCGA Melanoma	
Gene	SK-Mel-28 vs. Melanocytes	SK-Mel-147 vs. Melanocytes	UACC-62 vs. Melanocytes	ANOVA	Primary vs. Regional Skin Mets	Primary vs. Regional LN Mets	Primary vs. Distal Mets	Log-rank (Mantel-Cox) Test	Gehan-Breslow-Wilcoxon test
CSTF2	1.760	3.116	1.832	n.s.	n.s.	n.s.	n.s.	0.585	0.575
DDX3X	1.414	1.711	1.963	**0.039**	**0.025**	**0.004**	n.s.	0.767	0.640
DKC1	2.075	2.087	2.756	n.s.	n.s.	n.s.	n.s.	0.330	**0.043**
EIF1AX	1.985	2.326	5.548	n.s.	**0.031**	n.s.	n.s.	0.166	**0.050**
GNL3L	1.357	1.806	1.807	**0.015**	**0.012**	**0.001**	n.s.	0.718	0.443
MEX3C	2.204	1.662	1.541	n.s.	n.s.	n.s.	n.s.	0.112	**0.041**
RBMX	1.424	1.669	1.464	**0.015**	**0.012**	**0.001**	n.s.	0.718	0.443
SLC25A5	1.797	0.519	1.807						
CCDC124	1.471	1.091	1.414						
FLNA	0.912	1.813	0.637						
FMR1	0.829	0.727	0.484						
LAS1L	1.218	1.660	1.902						
MBNL3	1.676	0.311	1.298						
MECP2	1.425	0.599	0.765						
RBM3	1.420	1.611	1.222						
RBMX2	0.681	0.464	0.948						
RPGR	0.948	1.738	1.909						
RPS4X	1.015	1.401	1.365						
UBA1	1.226	1.309	2.033						
GSPT2	0.694	1.050	0.697						
HNRNPH2	0.350	0.449	0.459						
RBM10	1.032	1.240	1.405						
RPL10	0.933	1.477	0.805						
UPF3B	1.112	0.599	1.202						
UTP14A	1.092	0.875	1.243						
FAM120C	1.299	1.243	1.280						
NKRF	1.180	0.723	1.368						
NONO	1.102	1.301	1.045						
PHF6	1.203	0.865	1.137						
HUWE1	0.953	0.814	1.193						
HTATSF1	0.612	0.298	0.710						

Supplementary Table 2. Cancer-associated RBPs in melanoma. RNA-Seq based analysis of the cancer-associated RBP signature defined by Kechavarzi et al. (Genome Biology, 2014) in the indicated melanoma cell lines compared to normal skin melanocytes. Red and green correspond to fold changes (log2 scale) in mRNA expression, representing up or downregulation, respectively. Factors with statistically significant changes in the three cell lines with respect to melanocytes ($p<0.05$) are highlighted in light blue. Highlighted in orange are p-values of mRNA expression levels for these genes compared as indicated using data from TCGA melanomas. In grey is the prognostic value (overall survival) of melanoma patients expressing mRNA levels below or above the median of each of the indicated genes, and estimated from the TCGA dataset using Log-rank or Gehan-Breslow-Wilcoxon tests.

				SK-Mel-103			UACC-62		
**Gene Name**	**Alteration**	**Event**	**Exon Coordinates (hg19)**	**Regulation**	**FCSI**	**P-Value SI**	**Regulation**	**FC SI**	**P-Value SI**
**ATP5G3**	Alternative Exon 4	Intron retention	chr2(-):176043238-176043702	up	3.14	8.20E-03	up	2.11	1.82E-02
**CLDND1**	Alternative Exon 2	Alternative first exon	chr3(-):98240586-98241172	up	2.17	5.10E-03	up	3.07	2.72E-03
**CLSPN**	Alternative Exon 1	Alternative first exon	chr1(-):36235568-36235595	up	2.22	2.98E-02	up	2.21	9.80E-03
**DHX40P1**	Alternative Exon 5	Unknown	chr17(-):58079020-58079052	down	2.27	8.76E-03	down	2.22	3.63E-02
**DHX40P1**	Exon 7	Unknown	chr17(-):58075499-58075598	down	2.03	1.43E-02	down	2.21	3.79E-02
**DHX40P1**	Exon 8	Unknown	chr17(-):58073314-58073400	down	2.77	1.16E-02	down	2.27	2.08E-02
**DHX40P1**	Exon 10	Unknown	chr17(-):58064808-58064965	down	3.04	5.70E-04	down	2.41	2.95E-02
**DHX40P1**	Exon 12	Unknown	chr17(-):58053385-58053493	down	2.52	7.95E-03	down	2.48	3.20E-02
**EIF4E2**	Alternative Exon 6	Alternative last exon	chr2(+):233431947-233432364	up	3.07	2.01E-02	up	2.40	8.66E-03
**MACF1**	Exon 41	Alternative first exon	chr1(+):39796810-39802325	down	3.09	1.18E-02	down	2.87	1.27E-02
**MBNL1**	Exon 3	Exon skipping	chr3(+):152052900-152053019	up	2.02	4.57E-02	up	2.14	3.29E-02
**RREB1**	Alternative Exon 6	Intron retention	chr6(+):7181515-7181962	up	2.44	1.49E-02	up	3.60	5.38E-03

Supplementary Table 3. Splicing events modulated by CELF1 in melanoma cells. The table lists events associated with alternative splicing detected upon CELF1 depletion both in SK-Mel-103 and UACC-62 melanoma cell lines by human exon-junction arrays (HJAY). FCSI: Fold change splicing index. SI: Splicing index.

PANTHER GO-Slim Biological Process	Homo sapiens - (21002)	Input (412)	Input (Enrichment)	*p*-value
chromosome segregation (GO:0007059)	107	15	7.146	0.000001
DNA repair (GO:0006281)	163	17	5.316	0.000010
DNA replication (GO:0006260)	137	14	5.209	0.000202
regulation of cell cycle (GO:0051726)	124	11	4.522	0.010800
DNA metabolic process (GO:0006259)	362	32	4.506	0.000000
mitosis (GO:0007067)	336	22	3.338	0.000321
cell cycle (GO:0007049)	856	53	3.156	0.000000
organelle organization (GO:0006996)	786	39	2.529	0.000041
phosphate-containing compound metabolic process (GO:0006796)	847	37	2.227	0.001590
cellular component organization (GO:0016043)	1499	52	1.768	0.012800
cellular component organization or biogenesis (GO:0071840)	1672	56	1.707	0.016500
metabolic process (GO:0008152)	6314	165	1.332	0.002460
primary metabolic process (GO:0044238)	5425	140	1.316	0.035000
response to stimulus (GO:0050896)	2804	30	0.545	0.016600
single-multicellular organism process (GO:0044707)	1856	15	0.412	0.006640
multicellular organismal process (GO:0032501)	1874	15	0.408	0.005260
nucleobase-containing compound metabolic process (GO:0006139)	3019	86	1.452	0.053100
sensory perception (GO:0007600)	614	2	0.166	0.107000
cell surface receptor signaling pathway (GO:0007166)	1174	9	0.391	0.147000
meiosis (GO:0007126)	52	6	5.882	0.155000
immune response (GO:0006955)	671	3	0.228	0.195000
fatty acid biosynthetic process (GO:0006633)	42	5	6.069	0.380000
cellular process (GO:0009987)	7765	182	1.195	0.385000
chromatin organization (GO:0006325)	228	12	2.683	0.517000
nitrogen compound metabolic process (GO:0006807)	2105	60	1.453	0.540000
system development (GO:0048731)	987	8	0.413	0.630000
developmental process (GO:0032502)	1843	21	0.581	0.795000

Supplementary Table 4. Enrichment analyses of transcriptomic profiles modulated by both CELF1 and DEK in SK-Mel-19 and SK-Mel-103 melanoma cell lines. Transcriptomic profiles deregulated after depletion of CELF1 (HJAY data) or DEK (cDNA microarray data) were compared, and genes shared by these two datasets in both cell lines were analyzed by the PANTHER Database Overrepresentation Test to identify enriched biological processes. Gene Ontology cellular processes with Bofferroni-corrected *p*-value <0.05 are highlighted in blue (Fig. 5g). To illustrate the selectivity of the pathways identified, the table also includes pathways (not highlighted) that although with the potential to impinge in cancer cell biology, were not found as significantly deregulated in this comparative analysis.

ANTIBODY	DILUTION	COMPANY	CATALOGUE #
Mouse monoclonal anti CELF1 (clone 3B1)	1:500	Abcam	ab9549
Rabbit polyclonal anti CELF2	1:500	Sigma-Aldrich	AV40323
Mouse monoclonal anti CCND1 (clone A-12)	1:500	Santa Cruz Biotech.	sc-8396
Mouse monoclonal anti DEK (Clone 2)	1:1000	BD Biosciences	610948
Rabbit monoclonal anti DEK (clone EPR11034)	1:500	Abcam	ab166624
Rabbit polyclonal anti GFP	1:1000	Abcam	ab290
Rabbit polyclonal anti phospho-Akt (Ser473)	1:1000	Cell Signaling	9271
Rabbit polyclonal anti Akt (total)	1:1000	Cell Signaling	9272
Rabbit polyclonal anti phospho-ERK1/2 (Thr202/Tyr204)	1:1000	Cell Signaling	9101
Rabbit polyclonal anti ERK1/2 (total)	1:1000	Cell Signaling	9102
IgG from mouse serum	1:500	Sigma-Aldrich	I5381
Mouse monoclonal anti-β-actin (clone AC-15)	1:5000	Sigma-Aldrich	A5441
Donkey anti-Mouse IgG (H+L) Secondary Antibody, Alexa Fluor 555	1:400	Thermo Scientific	A-31570
Donkey anti-Rabbit IgG (H+L) Secondary Antibody, Alexa Fluor 488	1:400	Thermo Scientific	A-21206
FITC-conjugated anti-BrdU	1:100	BD Biosciences	556028

Supplementary Table 5. Antibodies used in this study. Indicated are the corresponding dilutions used, provider and catalogue number.

SEQUENCE	STUDY/COMPANY	CATALOGUE #
shRNA targeting CELF1 sequence: GCTGCATTAGAAGCTCAGAAT	Arnal-Estape et al. *Cancer Res* 70, 9927-9936 (2010)	TRCN0000017284
shRNA targeting CELF1 sequence: CGAGTCATGTTCTCTTCGTTT	Arnal-Estape et al. *Cancer Res* 70, 9927-9936 (2010)	TRCN0000017285
shRNA non-targeting control sequence: CAACAAGATGAAGAGCACCAA	Sigma-Aldrich	SHC002
shDEK double targeting sequences: GCTTGCTTAAGGCATACTTTT, AACCCCCTACAGATGAAGAGTTAA	Khodadoust et al.[2] *Cancer Res* 69, 6405-6413 (2009)	N/A
siRNA targeting CELF1 sequences (SMARTpool: ON-TARGETplus): GAGCCAACCUGUUCAUCUA, ACUCGGGUAUCCAGCAAUA, GCUGUUUAUUGGUAUGAUU, UGAAGAAUGCCGGAUAUUG	Dharmacon	L-020166
siRNA targeting non-targeting control sequences (ON-TARGETplus Non-targeting Control Pool): UGGUUUACAUGUCGACUAA, UGGUUUACAUGUUGUGUGA, UGGUUUACAUGUUUUCUGA, UGGUUUACAUGUUUUCCUA	Dharmacon	D-001810-10-20
gRNA targeting CELF1 sequence: GCACAACATGAAAGTCCTCCC	This paper	N/A
gRNA non-targeting control sequence: GCACTACCAGAGCTAACTCA	This paper	N/A

Supplementary Table 6. Gene targeting sequences for gene depletion in this study and the corresponding references and providers

GENE	FORWARD PRIMER (5' – 3')	REVERSE PRIMER (5' – 3')	°C
ACTN1	CGGGCCTCCTTCAACCACTTT	TCCACAATGCTCATGATGCGG	54
ANK2	TACCTCCAGACCCCAACATCC	TCTTTACCACGGTGTGTCCAT	54
AURKA	GGAGAGCTTAAAATTGCAGATTTTG	GCTCCAGAGATCCACCTTCTCAT	60
AURKB	AGGAGAACTCCTACCCCTGG	GCTCATGAGGACAAGTGCAG	60
BIRC5	CCCTTCTTGGAGGGCTGC	TGGGGTCGTCATCTGGCT	60
CCNB1	TCTGGATAATGGTGAATGGACA	CGATGTGGCATACTTGTTCTTG	60
CDC6	TCAGCCCCAGGAAACGTCT	GTCAAATACCAATCTTCGTCCC	60
CELF1	GTGGAAGACAGGAAGCTGTTT	GCCGTCTGTGCCATGGCTCTTG	60
CLSPN	GCTCCGTCCCTAGTGGAGC	GCTTCCTCTTGTGAAATGACG	60
DEK	GCCATGTTAAAGAGCATCTGTG	CAGAAGGCTTTGGATGCATTA	60
DNA2	GGGATGGTCGAAGTTAATAC	GTTTGGCTCTGGTTATAGCAAC	60
ESPL1	GGTGAAGCCAGGCACTTATC	GGGCCTGTTTACCCAGTTTC	60
EXO1	CACTCCACCTAGGACGAGAAAT	GGCTGGATGCTCACTTTGTT	60
FAM188A	GAGGAAACTGCTAGTATTTC	CCAAATTTATTTCCCCACAT	54
FEN1	AAGGGAGAGCGAGCTTAGGA	ATTCCCATGGCAACACAGAG	60
FOXM1	CAAGCCAGGCTGGAAGAACT	CTGGTCCAATGTCAAGTAGCG	60
FXR1	GATAATACAGAATCAGATCAG	CTGAAGGACCATGCTCTTCAA	54
GAPDH	GAAGGTGAAGGTCGGAGTCAAC	TGATTTTGGAGGGATCTCGCTC	60
HPRT	CCTGGCGTCGTGATTAGTGAT	AGACGTTCAGTCCTGTCCATAA	60
INSR	CCAAAGACAGACTCTCAGAT	AACATCGCCAAGGGACCTGC	54
MCM3	GGCCACCTACATTGCAGAAG	CCAGTGTTCGGGCTGTAACT	60
MCM4	CCTGGGGACAGAGTGAATGT	GACGTTTTGCATCCGTTTTC	60
MCM7	CATACATTGATCGACTGGCG	CCCCACCCTCTAAGGTCAGT	60
MEF2A	GAATGAACAGTAGGAAACCAG	GCTGGTCAGTGAATAATCAGT	54
POLA1	CATTTTTGATGCGGAGTGTG	CTTCGGGACAAGAATTGCTC	60
PPFIBP1	TAGTGAAATGGACAGTGAGA	CAATTTTTCTTGTAGCCTTT	54
SERCA1	GATGATCTTCAAGCTCCGGGC	CAGCTCTGCCTGAAGATGTG	54
TIMELESS	CAGCTCGTCTGCTGAGGAG	CTTTTCTGCCATCTCTCGCTG	60
DEK 3'UTR Fragment 1	see FLAG Forward Primer	GGTCAGCAGTAAGTTCTACTAAC	58
DEK 3'UTR Fragment 2	see FLAG Forward Primer	CATCTGTCACTTTTGTCATGCTG	58
DEK 3'UTR Fragment 3	see FLAG Forward Primer	GTGCTTGTACTTAATCCCACCC	58
DEK 3'UTR Fragment 4	see FLAG Forward Primer	CCTTTCCCTAGTGTCTGAGTAAC	58
FLAG	GATTACAAGGATGACGACGATAAG		58
	CLONING PRIMERS		
FRAGMENT	FORWARD PRIMER (5' – 3')	REVERSE PRIMER (5' – 3')	°C
DEK 3'UTR Fragment 1	AGCCCTCGAGGATTACAAGGATGACGA CGATAAGGATAGAGGACAGAGAAGAT	TAGGGGATCCCACACTGACAGAGA TGTGTATTC	58
DEK 3'UTR Fragment 2	AGCCCTCGAGGATTACAAGGATGACGA CGATAAGAAAATTCAGCGGCAGTGTG	TAGGGGATCCGGGTAACTGTTCCT TCAGTGTTGCC	58
DEK 3'UTR Fragment 3	AGCCCTCGAGGATTACAAGGATGACGA CGATAAGTGCTTTGCCTCGAAAGTGT	TAGGGGATCCGGTGCAACAAATG TGTCATTGTGTC	58
DEK 3'UTR Fragment 4	AGCCCTCGAGGATTACAAGGATGACGACG ATAAGAATTAAGTGTTGAAAAATATCTTTGC	TAGGGGATCCTGGTGATACATCC ATTTAATAAGTGTGCG	58
CELF1 gRNA	CACCGCACAACATGAAAGTCCTCCC	AAACGGGAGGACTTTCATGTTGTGC	-
CRISPR scrambled	CACCGCACTACCAGAGCTAACTCA	AAACTGAGTTAGCTCTGGTAGTGC	-

Supplementary Table 7. Primers for PCR/qPCR and cloning used in this study. °C: annealing temperature.
