# Supplementary material for: Systems analysis identifies melanoma-enriched pro-oncogenic networks controlled by the RNA binding protein CELF1
Source: Nat Commun. 2017 Dec 21;8:2249. doi: 10.1038/s41467-017-02353-y (PMC5740069; doi:10.1038/s41467-017-02353-y)
Supplement: Supplementary file 2 — Description of Additional Supplementary Files [file 41467_2017_2353_MOESM2_ESM.pdf]

Description of Additional Supplementary Files

File Name: Supplementary Data 1
Description: **Genetic alterations of mRPBs in melanoma.** Listed are known 692 mRBPs, with the corresponding domains as defined in Gerstberger et al. (Nat. Rev. Genet. 2014). The fraction of melanoma patients included in the TCGA database (n=479) found with mutations or copy number variations are indicated as percentages. Data was extracted from cBioPortal.

File Name: Supplementary Data 2
Description: **Gene Ontology (GO) functional terms associated to genes found deregulated in melanoma by customized microarray.** Indicated are the GO identification code (ID) for each term, the number of deregulated genes per category (#DEG), and the corresponding p-values generated in comparative analyses of melanoma cell lines (SK-Mel-19 and SK-Mel-103) defined with respect to pools of normal melanocytes. RNA-associated processes are highlighted in light blue, and cancer-associated functions in light pink.

File Name: Supplementary Data 3
Description: **Comparative analysis of genome-wide analyses of CELF1 targets in human cells.** Listed are the experimental settings as well as the authors and techniques of the different datasets. The upper table summarizes the total number of genes identified per study, and those unique for each of the system analyzed. In the bottom table, genes are listed in alphabetical order, being labeled as 1 or 0 depending on whether or not they were identified as CELF1-bound transcripts in the indicated studies, respectively.

File Name: Supplementary Data 4
Description: **CELF1-bound transcripts in melanoma.** List of CELF1-bound transcripts found by RIP-Seq in melanoma cell lines SK-Mel-103 and UACC-62, sorted by region of binding, and classified depending on the presence (or absence) of canonical GU-rich elements (GREs). Genes not previously linked to CELF1 in any dataset are labelled in yellow.

File Name: Supplementary Data 5
Description: **Transcripts that are targets and mRNA-regulated effectors of CELF1 in melanoma.** Tables list CELF1-bound transcripts whose expression was found by transcriptomic analyses (HJAY) to be upregulated (red) or deregulated (green) upon CELF1 depletion

File Name: Supplementary Data 6
Description**: Functional information of the CELF1-contolled network depicted** in Fig. 5a, which was built upon comparative analyses of transcriptomic and proteomic analyses (HJAY and iTRAQ, respectively). Data were manually curated by mining the Human Gene Database (GeneCards) and PubMed (National Center for Biotechnology). Downregulated genes upon CELF1 depletion validated by RT-PCR are shown in green. In bold are those genes confirmed to be regulated also by DEK as demonstrated by RT-PCR or cDNA microarrays.

File Name: Supplementary Data 7
Description: **Bioinformatic tools and techniques used in this study, with the corresponding references**
