# Supplementary material for: Systems analysis identifies melanoma-enriched pro-oncogenic networks controlled by the RNA binding protein CELF1
Source: Nat Commun. 2017 Dec 21;8:2249. doi: 10.1038/s41467-017-02353-y (PMC5740069; doi:10.1038/s41467-017-02353-y)
Supplement: Supplementary file 3 — Supplementary Data 1 [file 41467_2017_2353_MOESM3_ESM.docx]

| **Gene** | **Domain [count]** | **%** |
| --- | --- | --- |
| A1CF | RRM_1[2], DND1_DSRM[1], RRM_6[1] | 7.32 |
| ABCF1 | ABC_tran_2[1], ABC_tran[2] | 4.81 |
| ABT1 | RRM_6[1] | 4.18 |
| ACIN1 | SAP[1], RRM_5[1] | 2.51 |
| ACO1 | Aconitase_C[1], Aconitase[1] | 2.51 |
| ADAD1 | A_deamin[1], dsrm[1] | 3.13 |
| ADAD2 | A_deamin[1], dsrm[1] | 2.51 |
| ADAR | A_deamin[1], z-alpha[2], dsrm[3] | 4.18 |
| ADARB1 | A_deamin[1], dsrm[2] | 2.51 |
| ADARB2 | A_deamin[1], dsrm[2] | 4.39 |
| AFF1 | AF-4[1] | 3.55 |
| AFF2 | AF-4[1] | 5.64 |
| AFF3 | AF-4[1] | 5.23 |
| AFF4 | AF-4[1] | 5.02 |
| AGFG1 | ArfGap[1] | 1.67 |
| AKAP1 | TUDOR[1], KH_1[1], DUF3552[1] | 5.23 |
| AKAP17A | . | 1.04 |
| AKAP8 | AKAP95[1] | 1.67 |
| AKAP8L | AKAP95[1] | 1.25 |
| ALKBH1 | 2OG-FeII_Oxy_2[1] | 1.46 |
| ALKBH5 | 2OG-FeII_Oxy_2[1] | 1.04 |
| ALYREF | FoP_duplication[1], RRM_1[1], FYTT[1] | 4.18 |
| ANGEL1 | Exo_endo_phos[1] | 2.71 |
| ANGEL2 | Exo_endo_phos[1] | 3.13 |
| APEX1 | Exo_endo_phos[1] | 0.62 |
| API5 | API5[1] | 0.83 |
| APOBEC1 | APOBEC_N[1] | 1.88 |
| APOBEC2 | APOBEC_N[1] | 3.97 |
| APOBEC3F | APOBEC_N[2] | 1.88 |
| APOBEC3G | APOBEC_N[2] | 2.3 |
| APOBEC4 | APOBEC_N[1] | 2.92 |
| AQR | AAA_11[1], AAA_12[1] | 4.6 |
| ARHGEF28 | PH[1], RhoGEF[1], C1_1[1] | 3.97 |
| ARL6IP4 | SR-25[1] | 1.04 |
| ATXN1 | ATXN-1_C[1], AXH[1] | 8.78 |
| ATXN1L | AXH[1] | 0.41 |
| ATXN2 | PAM2[1], LsmAD[1], SM-ATX[1] | 2.09 |
| ATXN2L | PAM2[1], LsmAD[1], SM-ATX[1] | 2.3 |
| AUH | ECH[1] | 0.2 |
| BCLAF1 | THRAP3_BCLAF1[1] | 12.55 |
| BICC1 | SAM_1[1], KH_1[3] | 3.97 |
| BOLL | RRM_1[1] | 0.83 |
| BUD13 | Bud13[1] | 4.18 |
| BZW1 | W2[1] | 0.41 |
| BZW2 | W2[1] | 2.92 |
| C12orf65 | RF-1[1] | 0.62 |
| C1QBP | MAM33[1] | 1.04 |
| CACTIN | CactinC_cactus[1], Cactin_mid[1] | 1.46 |
| CALR | Calreticulin[1] | 0.83 |
| CAPRIN1 | Caprin-1_C[1] | 1.67 |
| CAPRIN2 | C1q[1], Caprin-1_C[1] | 3.13 |
| CARHSP1 | CSD[1] | 0.41 |
| CASC3 | Btz[1] | 2.51 |
| CCAR1 | SAP[1], DBC1[1], MIP-T3[1], S1-like[1] | 1.25 |
| CCAR2 | DBC1[1], S1-like[1] | 3.13 |
| CDC40 | WD40[5] | 3.55 |
| CDC5L | Myb_Cef[1], Myb_DNA-bind_6[1] | 3.97 |
| CELF1 | RRM_1[3] | 1.04 |
| CELF2 | RRM_1[3] | 0.62 |
| CELF3 | RRM_1[3] | 3.34 |
| CELF4 | RRM_5[1], RRM_1[2] | 3.13 |
| CELF5 | RRM_1[3] | 1.67 |
| CELF6 | RRM_1[3] | 1.67 |
| CHERP | G-patch[1], Surp[1], CTD_bind[1] | 2.71 |
| CHTOP | FoP_duplication[1] | 3.55 |
| CIRBP | RRM_1[1] | 0.62 |
| CLASRP | DRY_EERY[1] | 1.25 |
| CLK1 | Pkinase[1] | 1.04 |
| CLK2 | Pkinase[1] | 4.39 |
| CLK3 | Pkinase[1] | 1.88 |
| CLK4 | Pkinase[1] | 1.25 |
| CLP1 | MobB[1], Clp1[1] | 1.04 |
| CMTR1 | G-patch[1], FtsJ[1] | 5.02 |
| CMTR2 | FtsJ[1] | 2.51 |
| CNBP | zf-CCHC[7] | 0.62 |
| CNOT1 | DUF3819[1], Not1[1] | 4.81 |
| CNOT10 | TPR_1[2], BLOC1_2[1] | 2.51 |
| CNOT11 | DUF2363[1] | 0.2 |
| CNOT2 | NOT2_3_5[1] | 3.76 |
| CNOT3 | Not3[1], NOT2_3_5[1] | 1.25 |
| CNOT4 | RRM_5[1], zf-RING_4[1] | 6.06 |
| CNOT6 | LRR_8[1], Exo_endo_phos[1] | 2.3 |
| CNOT6L | LRR_8[1], Exo_endo_phos[1] | 1.67 |
| CNOT7 | CAF1[1] | 2.3 |
| CNOT8 | CAF1[1] | 1.04 |
| CNP | CNPase[1], AAA_33[1] | 0.2 |
| CPEB1 | RRM_1[1], RRM_6[1] | 2.71 |
| CPEB2 | RRM_1[1] | 1.88 |
| CPEB3 | RRM_1[1] | 1.04 |
| CPEB4 | RRM_1[1] | 2.3 |
| CPSF1 | MMS1_N[1], CPSF_A[1] | 7.53 |
| CPSF2 | RMMBL[1], Beta-Casp[1], Lactamase_B[1], CPSF100_C[1] | 0.83 |
| CPSF3 | RMMBL[1], Beta-Casp[1], Lactamase_B[1], CPSF73-100_C[1] | 1.04 |
| CPSF3L | RMMBL[1], Beta-Casp[1], Lactamase_B[1] | 2.71 |
| CPSF4 | zf-CCHC[1], zf-CCCH[3] | 1.67 |
| CPSF4L | zf-CCCH[3] | 3.55 |
| CPSF6 | RRM_6[1] | 4.81 |
| CPSF7 | RRM_1[1] | 0.83 |
| CRNKL1 | HAT[4] | 3.97 |
| CRYZ | ADH_N[1], ADH_zinc_N[1] | 1.67 |
| CSDC2 | CSD[1] | 2.92 |
| CSDE1 | SUZ-C[1], CSD[5] | 4.18 |
| CSTF1 | WD40[4] | 2.71 |
| CSTF2 | RRM_1[1], CSTF2_hinge[1], CSTF_C[1] | 1.25 |
| CSTF2T | RRM_1[1], CSTF2_hinge[1], CSTF_C[1] | 2.09 |
| CSTF3 | Suf[1], TPR_16[1] | 1.46 |
| CTIF | MIF4G[1] | 3.13 |
| CWC15 | Cwf_Cwc_15[1] | 2.92 |
| CWC22 | MIF4G[1], MA3[1], CAF-1_p150[1] | 1.04 |
| CWC25 | Cir_N[1], CWC25[1] | 1.46 |
| CWC27 | Pro_isomerase[1] | 1.88 |
| CWF19L1 | Metallophos_3[1], CwfJ_C_1[1], CwfJ_C_2[1] | 0.62 |
| CWF19L2 | CwfJ_C_1[1], CwfJ_C_2[1] | 4.18 |
| CXorf23 | THRAP3_BCLAF1[1] | 2.09 |
| DAZ1 | RRM_1[3] | 0 |
| DAZ2 | RRM_1[1] | 0.2 |
| DAZ3 | RRM_1[1] | 0 |
| DAZ4 | RRM_1[2] | 0 |
| DAZAP1 | RRM_1[2] | 1.67 |
| DAZL | RRM_1[1] | 2.3 |
| DBR1 | Metallophos[1], DBR1[1] | 1.67 |
| DCP1A | DCP1[1] | 0.83 |
| DCP1B | DCP1[1] | 2.51 |
| DCP2 | DCP2[1], NUDIX[1] | 2.51 |
| DCPS | DcpS[1], DcpS_C[1] | 2.92 |
| DDX1 | Helicase_C[1], DEAD[1] | 0.62 |
| DDX17 | Helicase_C[1], DEAD[1] | 4.39 |
| DDX19A | Helicase_C[1], DEAD[1] | 0.41 |
| DDX19B | DEAD[1] | 0.62 |
| DDX25 | Helicase_C[1], DEAD[1] | 3.76 |
| DDX26B | VWA_2[1], INT_SG_DDX_CT_C[1] | 1.46 |
| DDX39A | Helicase_C[1], DEAD[1] | 1.25 |
| DDX39B | Helicase_C[1], DEAD[1] | 4.81 |
| DDX3X | Helicase_C[1], DEAD[1] | 4.81 |
| DDX3Y | Helicase_C[1], DEAD[1] | 0.62 |
| DDX4 | Helicase_C[1], DEAD[1] | 2.92 |
| DDX41 | Helicase_C[1], DEAD[1] | 2.92 |
| DDX42 | Helicase_C[1], DEAD[1] | 5.43 |
| DDX43 | Helicase_C[1], DEAD[1], KH_1[1] | 4.81 |
| DDX5 | P68HR[2], Helicase_C[1], DEAD[1] | 4.81 |
| DDX53 | Helicase_C[1], DEAD[1], KH_1[1] | 3.34 |
| DDX59 | zf-HIT[1], Helicase_C[1], DEAD[1] | 5.64 |
| DDX6 | Helicase_C[1], DEAD[1] | 2.92 |
| DEK | SAP[1], DEK_C[1] | 5.23 |
| DENR | SUI1[1] | 0.41 |
| DGCR14 | Es2[1] | 3.97 |
| DHX15 | OB_NTP_bind[1], DUF1777[1], HA2[1], Helicase_C[1], DEAD[1] | 2.71 |
| DHX29 | OB_NTP_bind[1], HA2[1], Helicase_C[1], DEAD[1] | 3.13 |
| DHX30 | OB_NTP_bind[1], HA2[1], Helicase_C[1], dsrm[1], DEAD[1] | 1.67 |
| DHX32 | OB_NTP_bind[1], HA2[1] | 1.88 |
| DHX33 | OB_NTP_bind[1], HA2[1], Helicase_C[1], DEAD[1] | 1.67 |
| DHX34 | OB_NTP_bind[1], HA2[1], Helicase_C[1], DEAD[1] | 2.92 |
| DHX35 | OB_NTP_bind[1], HA2[1], Helicase_C[1], DEAD[1] | 2.3 |
| DHX36 | OB_NTP_bind[1], HA2[1], Helicase_C[1], DEAD[1] | 2.51 |
| DHX38 | OB_NTP_bind[1], HA2[1], Helicase_C[1], DEAD[1] | 2.51 |
| DHX40 | OB_NTP_bind[1], HA2[1], Helicase_C[1], DEAD[1] | 4.81 |
| DHX57 | OB_NTP_bind[1], HA2[1], Helicase_C[1], DEAD[1], RWD[1] | 1.88 |
| DHX8 | OB_NTP_bind[1], S1[1], Helicase_C[1], DEAD[1] | 2.92 |
| DND1 | RRM_1[1], DND1_DSRM[1] | 1.04 |
| DQX1 | OB_NTP_bind[1], HA2[1], AAA_22[1] | 2.51 |
| DRG1 | MMR_HSR1[1], TGS[1] | 0.41 |
| DRG2 | MMR_HSR1[1], TGS[1] | 1.25 |
| DUSP11 | DSPc[1] | 0.41 |
| DYNC1H1 | Dynein_heavy[1], AAA_9[1], DHC_N1[1], DHC_N2[1], MT[1], AAA_6[1], AAA_5[1], AAA_7[1], AAA_8[1] | 5.02 |
| DYNLL1 | Dynein_light[1] | 0.41 |
| DZIP1 | Dzip-like_N[1], zf-C2H2[1] | 5.43 |
| DZIP1L | Dzip-like_N[1] | 1.67 |
| DZIP3 | zf-RING_2[1] | 2.51 |
| EDC3 | LSM14[1], YjeF_N[1] | 2.3 |
| EDC4 | WD40[1] | 3.55 |
| EEF2K | Alpha_kinase[1] | 2.09 |
| EIF1 | SUI1[1] | 0.2 |
| EIF1AD | eIF-1a[1] | 1.67 |
| EIF1AX | eIF-1a[1] | 1.25 |
| EIF1AY | eIF-1a[1] | 0 |
| EIF1B | SUI1[1] | 1.04 |
| EIF2AK1 | Pkinase[2] | 3.76 |
| EIF2AK3 | PQQ_2[1], Pkinase[2] | 4.6 |
| EIF2B1 | IF-2B[1] | 0.83 |
| EIF2B2 | IF-2B[1] | 0.83 |
| EIF2B3 | NTP_transferase[1] | 1.46 |
| EIF2B4 | IF-2B[1] | 0.41 |
| EIF2B5 | Hexapep[1], NTP_transferase[1], W2[1] | 1.46 |
| EIF3A | PCI[1] | 2.3 |
| EIF3B | eIF2A[2], RRM_1[1] | 4.18 |
| EIF3C | eIF-3c_N[1], PCI[1] | 0.2 |
| EIF3CL | eIF-3c_N[1], PCI[1] | 0 |
| EIF3D | eIF-3_zeta[1] | 1.88 |
| EIF3E | eIF3_N[1], PCI[1] | 4.18 |
| EIF3G | RRM_1[1], eIF3g[1] | 0.62 |
| EIF3H | XkdN[1], JAB[1] | 4.81 |
| EIF3I | WD40[4] | 1.46 |
| EIF3J | eIF3_subunit[1] | 1.88 |
| EIF3K | PCI_Csn8[1] | 0.83 |
| EIF3L | Paf67[1] | 2.92 |
| EIF3M | PCI[1] | 0.2 |
| EIF4A1 | Helicase_C[1], DEAD[1] | 1.25 |
| EIF4A2 | Helicase_C[1], DEAD[1] | 1.46 |
| EIF4A3 | Helicase_C[1], DEAD[1] | 5.02 |
| EIF4B | RRM_1[1] | 1.25 |
| EIF4E | IF4E[2] | 1.04 |
| EIF4E1B | IF4E[1] | 1.88 |
| EIF4E2 | IF4E[1] | 0.62 |
| EIF4E3 | IF4E[1] | 3.13 |
| EIF4ENIF1 | EIF4E-T[1] | 0.83 |
| EIF4G1 | MIF4G[1], MA3[1], W2[1] | 4.18 |
| EIF4G2 | MIF4G[1], MA3[1], W2[1] | 1.25 |
| EIF4G3 | MIF4G[1], MA3[1], W2[1] | 4.81 |
| EIF4H | RRM_1[1] | 1.88 |
| EIF5 | eIF-5_eIF-2B[1], W2[1] | 1.46 |
| EIF5A | KOW[1], eIF-5a[1] | 0.41 |
| EIF5A2 | KOW[1], eIF-5a[1] | 0.83 |
| EIF5AL1 | eIF-5a[1] | 0.83 |
| EIF6 | eIF-6[1] | 1.46 |
| ELAVL1 | RRM_1[3] | 0.83 |
| ELAVL2 | RRM_1[3] | 9.41 |
| ELAVL3 | RRM_1[3] | 1.88 |
| ELAVL4 | RRM_1[3] | 3.34 |
| ENOX1 | RRM_1[1] | 1.88 |
| ENOX2 | RRM_1[1] | 1.67 |
| ERN1 | PQQ_2[1], Pkinase[1], Ribonuc_2-5A[1] | 6.27 |
| ERN2 | PQQ_2[1], Pkinase[1], Ribonuc_2-5A[1] | 5.02 |
| ESRP1 | RRM_6[3] | 7.74 |
| ESRP2 | RRM_6[3] | 1.88 |
| ETF1 | eRF1_1[1], eRF1_2[1], eRF1_3[1] | 0.83 |
| EWSR1 | zf-RanBP[1], RRM_6[1] | 2.71 |
| FAM103A1 | RAM[1] | 1.04 |
| FAM120A | . | 1.46 |
| FAM98A | DUF2465[1] | 0.62 |
| FASTK | FAST_1[1], FAST_2[1], RAP[1] | 4.6 |
| FASTKD1 | FAST_1[1], FAST_2[1], RAP[1] | 1.04 |
| FASTKD2 | FAST_1[1], FAST_2[1], RAP[1] | 0.83 |
| FASTKD3 | FAST_1[1], FAST_2[1], RAP[1] | 3.13 |
| FASTKD5 | FAST_1[1], FAST_2[1], RAP[1] | 3.13 |
| FIP1L1 | Fip1[1], Pkinase_Tyr[1] | 3.55 |
| FMR1 | Agenet[1], FXR1P_C[1], KH_1[2] | 1.46 |
| FRG1 | FRG1[1] | 3.55 |
| FRG1BP | FRG1[1] | 11.29 |
| FTO | FTO_CTD[1], FTO_NTD[1] | 1.04 |
| FUBP1 | DUF1897[2], KH_1[4] | 1.88 |
| FUBP3 | KH_1[4] | 1.46 |
| FUS | zf-RanBP[1], RRM_1[1] | 0.62 |
| FXR1 | Agenet[1], FXR1P_C[1], KH_1[2] | 1.88 |
| FXR2 | Agenet[1], FXR1P_C[1], KH_1[2] | 0.83 |
| FYTTD1 | FYTT[1] | 1.88 |
| G3BP1 | RRM_1[1], NTF2[1] | 1.46 |
| G3BP2 | RRM_1[1], NTF2[1] | 1.04 |
| GAPDH | Gp_dh_C[1], Gp_dh_N[1] | 1.67 |
| GCFC2 | GCFC[1] | 1.46 |
| GFM1 | EFG_C[1], GTP_EFTU_D2[1], EFG_IV[1], EFG_II[1], GTP_EFTU[1] | 1.46 |
| GFM2 | EFG_C[1], GTP_EFTU_D2[1], EFG_IV[1], EFG_II[1], GTP_EFTU[1] | 1.67 |
| GLE1 | GLE1[1] | 0.62 |
| GPATCH1 | G-patch[1], Cwf_Cwc_15[1], DUF1604[1] | 3.34 |
| GRSF1 | RRM_6[3] | 0.83 |
| GSPT1 | PAM2[1], GTP_EFTU_D3[1], GTP_EFTU[1] | 1.46 |
| GSPT2 | PAM2[1], GTP_EFTU_D3[1], GTP_EFTU[1] | 2.92 |
| GTF2F1 | TFIIF_alpha[1] | 1.25 |
| GTPBP1 | GTP_EFTU_D2[1], GTP_EFTU_D3[1], GTP_EFTU[1] | 4.18 |
| GTPBP2 | GTP_EFTU_D2[1], GTP_EFTU_D3[1], GTP_EFTU[1] | 3.34 |
| GUF1 | EFG_C[1], GTP_EFTU_D2[1], LepA_C[1], EFG_II[1], GTP_EFTU[1] | 1.88 |
| HABP4 | HABP4_PAI-RBP1[1] | 0.41 |
| HBS1L | HBS1_N[1], GTP_EFTU_D2[1], GTP_EFTU_D3[1], GTP_EFTU[1] | 2.71 |
| HDLBP | KH_1[14] | 3.34 |
| HELZ | PAM2[1], AAA_19[1], AAA_11[1], zf-CCCH[1], AAA_12[1] | 5.64 |
| HELZ2 | AAA_11[2], RNB[1], AAA_12[2] | 6.9 |
| HNRNPA0 | RRM_1[1], RRM_6[1] | 0.62 |
| HNRNPA1 | HnRNPA1[1], RRM_1[2] | 0.83 |
| HNRNPA1L2 | HnRNPA1[1], RRM_1[2] | 1.04 |
| HNRNPA2B1 | RRM_1[1], RRM_6[1] | 2.09 |
| HNRNPA3 | RRM_1[1], RRM_6[1] | 0.41 |
| HNRNPAB | RRM_1[2], CBFNT[1] | 1.67 |
| HNRNPC | RRM_1[1] | 0.62 |
| HNRNPCL1 | RRM_1[1] | 8.15 |
| HNRNPD | RRM_1[2], CBFNT[1] | 0.62 |
| HNRNPDL | RRM_1[2], CBFNT[1] | 0.2 |
| HNRNPF | zf-RNPHF[1], RRM_1[1], RRM_6[2] | 1.67 |
| HNRNPH1 | zf-RNPHF[1], RRM_6[3] | 2.09 |
| HNRNPH2 | zf-RNPHF[1], RRM_6[3] | 0.62 |
| HNRNPH3 | RRM_6[2] | 0.2 |
| HNRNPK | ROKNT[1], KH_1[3] | 1.04 |
| HNRNPL | RRM_5[3] | 2.51 |
| HNRNPLL | RRM_5[2], RRM_6[1] | 0.62 |
| HNRNPM | HnRNP_M[1], RRM_1[3] | 2.71 |
| HNRNPR | RRM_1[3] | 3.55 |
| HNRNPU | SAP[1], SPRY[1], AAA_33[1] | 4.6 |
| HNRNPUL1 | SAP[1], SPRY[1], AAA_33[1] | 1.67 |
| HNRNPUL2 | SAP[1], SPRY[1], AAA_33[1] | 0.83 |
| HTATSF1 | RRM_5[1], RRM_1[1] | 1.88 |
| IGF2BP1 | RRM_1[1], KH_1[4], RRM_6[1] | 4.6 |
| IGF2BP2 | RRM_1[2], KH_1[4] | 1.46 |
| IGF2BP3 | RRM_1[1], KH_1[4], RRM_6[1] | 3.76 |
| ILF2 | DZF[1] | 3.76 |
| ILF3 | dsrm[2], DZF[1] | 2.09 |
| IPO11 | IBN_N[1], Xpo1[1] | 2.3 |
| IPO13 | IBN_N[1], Xpo1[1] | 1.88 |
| IPO4 | IBN_N[1], HEAT_2[1], HEAT_EZ[1] | 2.51 |
| IPO5 | IBN_N[1], HEAT_2[1], HEAT[1] | 4.39 |
| IPO7 | Cse1[1], IBN_N[1] | 2.09 |
| IPO9 | IBN_N[1], Xpo1[1] | 5.64 |
| IREB2 | Aconitase_C[1], Aconitase[2] | 3.34 |
| ISY1 | Isy1[1] | 0.41 |
| JAKMIP1 | . | 6.27 |
| KHDC1 | . | 4.6 |
| KHDC1L | . | 3.34 |
| KHDRBS1 | KH_1[1] | 2.51 |
| KHDRBS2 | KH_1[1] | 8.57 |
| KHDRBS3 | KH_1[1] | 5.85 |
| KHNYN | RNase_Zc3h12a[1] | 1.25 |
| KHSRP | DUF1897[2], KH_1[4] | 0.62 |
| KPNB1 | IBN_N[1], HEAT_EZ[1] | 1.25 |
| L1TD1 | Transposase_22[2] | 5.43 |
| LARP1 | La[1] | 3.55 |
| LARP1B | La[1] | 1.88 |
| LARP4 | La[1], RRM_6[1] | 2.71 |
| LARP4B | La[1], RRM_5[1] | 3.76 |
| LARP6 | SUZ-C[1], La[1] | 2.09 |
| LIN28A | zf-CCHC[1], CSD[1] | 1.46 |
| LIN28B | zf-CCHC[1], CSD[1] | 4.18 |
| LRPPRC | PPR_2[2], PPR[1] | 3.97 |
| LSM12 | AD[1] | 0.41 |
| LSM14A | LSM14[1], FDF[1] | 2.09 |
| LSM14B | LSM14[1], FDF[1] | 2.09 |
| LUC7L | LUC7[1] | 1.25 |
| LUC7L2 | LUC7[1] | 4.81 |
| LUC7L3 | LUC7[1] | 2.92 |
| LUZP4 | FYTT[1] | 1.46 |
| MAGOH | Mago_nashi[1] | 0.62 |
| MAGOHB | Mago_nashi[1] | 1.46 |
| MATR3 | RRM_5[2] | 2.09 |
| MBNL1 | zf-CCCH[1] | 1.46 |
| MBNL2 | zf-CCCH[2] | 1.25 |
| MBNL3 | zf-CCCH[2] | 0.62 |
| MECP2 | MBD[1] | 2.09 |
| METTL14 | MT-A70[1] | 0.83 |
| METTL3 | MT-A70[1] | 0.83 |
| MEX3A | KH_1[2], zf-C3HC4_3[1] | 2.71 |
| MEX3B | KH_1[2], zf-C3HC4_3[1] | 1.04 |
| MEX3C | KH_1[2], zf-C3HC4_3[1] | 0.83 |
| MEX3D | KH_1[2], zf-C3HC4_3[1] | 0.41 |
| MIF4GD | MIF4G[1] | 3.97 |
| MKRN1 | zf-C3HC4[1], zf-CCCH[3] | 5.02 |
| MKRN2 | zf-RING_2[1], zf-CCCH[4] | 1.88 |
| MKRN3 | zf-C3HC4[1], zf-CCCH[1] | 4.39 |
| MOV10 | AAA_19[1], AAA_11[1], AAA_12[1] | 3.55 |
| MOV10L1 | AAA_11[2], AAA_12[1], S1-like[1] | 7.11 |
| MRTO4 | Ribosomal_L10[1] | 1.25 |
| MSI1 | RRM_1[2] | 1.04 |
| MSI2 | RRM_1[2] | 3.76 |
| MTHFSD | RRM_1[1], 5-FTHF_cyc-lig[1] | 1.46 |
| MTIF3 | IF3_C[1], IF3_N[1] | 0.62 |
| MTPAP | GOLGA2L5[1], PAP_assoc[1] | 1.04 |
| MTRF1 | PCRF[1], RF-1[1] | 0.83 |
| MTRF1L | PCRF[1], RF-1[1] | 1.46 |
| MYEF2 | RRM_1[3] | 1.88 |
| NAA38 | LSM[1] | 3.34 |
| NANOS1 | zf-nanos[1] | 0.83 |
| NANOS2 | zf-nanos[1] | 0.83 |
| NANOS3 | zf-nanos[1] | 1.25 |
| NCBP1 | MIF4G_like_2[1], MIF4G[1], MIF4G_like[1] | 0.2 |
| NCBP2 | RRM_5[1] | 1.25 |
| NCBP2L | RRM_1[1] | 0.41 |
| NELFE | RRM_1[1] | 5.02 |
| NFX1 | R3H[1], zf-NF-X1[7] | 2.51 |
| NGDN | Sas10_Utp3[1] | 1.46 |
| NMD3 | NMD3[1] | 1.46 |
| NOCT | Exo_endo_phos[1] | 1.04 |
| NOL3 | CARD[1] | 1.25 |
| NOVA1 | KH_1[3] | 4.6 |
| NOVA2 | KH_1[3] | 0.62 |
| NR0B1 | Hormone_recep[1], NR_Repeat[4] | 1.04 |
| NSRP1 | DUF2040[1] | 1.46 |
| NUDT21 | NUDIX_2[1] | 1.25 |
| NUFIP2 | NUFIP2[1] | 1.67 |
| NUPL2 | zf-CCCH[1] | 2.51 |
| NUTF2 | NTF2[1] | 0.2 |
| NXF1 | LRR_4[1], Tap-RNA_bind[1], NTF2[1], TAP_C[1] | 1.25 |
| NXF2 | LRR_4[1], Tap-RNA_bind[1], NTF2[1], TAP_C[1] | 1.25 |
| NXF2B | LRR_4[1], Tap-RNA_bind[1], NTF2[1], TAP_C[1] | 1.04 |
| NXF3 | Tap-RNA_bind[1], NTF2[1] | 3.13 |
| NXF5 | LRR_4[1], Tap-RNA_bind[1] | 2.09 |
| NXT1 | NTF2[1] | 0.83 |
| NXT2 | NTF2[1] | 0.41 |
| PABPC1 | RRM_1[4], PABP[1] | 4.81 |
| PABPC1L | RRM_1[4], PABP[1] | 1.88 |
| PABPC1L2A | RRM_1[2] | 1.04 |
| PABPC1L2B | RRM_1[2] | 1.04 |
| PABPC3 | RRM_1[4], PABP[1] | 2.3 |
| PABPC4 | RRM_1[4], PABP[1] | 2.09 |
| PABPC4L | RRM_1[4] | 1.25 |
| PABPC5 | RRM_1[4] | 1.67 |
| PABPN1 | RRM_1[1] | 0.83 |
| PABPN1L | RRM_1[1] | 0.83 |
| PAIP1 | PAM2[1], MIF4G[1] | 3.34 |
| PAIP2 | PAM2[1] | 1.04 |
| PAIP2B | PAM2[1] | 0.2 |
| PAN2 | RNase_T[1], UCH_1[1] | 3.13 |
| PAN3 | Pkinase[1], zf-CCCH[1] | 1.88 |
| PAPD4 | PAP_assoc[1], NTP_transf_2[1] | 2.09 |
| PAPOLA | PAP_RNA-bind[1], PAP_central[1] | 1.25 |
| PAPOLB | PAP_RNA-bind[1], PAP_central[1] | 4.18 |
| PAPOLG | PAP_RNA-bind[1], PAP_central[1] | 1.46 |
| PARK7 | DJ-1_PfpI[1] | 1.67 |
| PARN | CAF1[2], RNA_bind[1] | 2.3 |
| PARP1 | BRCT[1], WGR[1], PARP_reg[1], zf-PARP[2], PARP[1], PADR1[1] | 4.39 |
| PATL1 | PAT1[1] | 2.51 |
| PATL2 | PAT1[1] | 2.09 |
| PCBP1 | KH_1[3] | 0.2 |
| PCBP2 | KH_1[3] | 1.04 |
| PCBP3 | KH_1[3] | 1.67 |
| PCBP4 | KH_1[3] | 0.62 |
| PCF11 | CTD_bind[1] | 4.6 |
| PDCD4 | MA3[2] | 1.88 |
| PDE12 | Exo_endo_phos[1] | 1.46 |
| PELO | eRF1_1[1], eRF1_2[1], eRF1_3[1] | 1.04 |
| PHF5A | PHF5[1] | 3.34 |
| PHRF1 | PHD[1], zf-RING_2[1] | 2.92 |
| PLRG1 | WD40[6] | 2.3 |
| PNLDC1 | . | 2.71 |
| PNN | Pinin_SDK_N[1], Pinin_SDK_memA[1] | 1.25 |
| PNRC2 | PNRC[1] | 1.46 |
| POLDIP3 | RRM_1[1] | 3.97 |
| POLR2A | RNA_pol_Rpb1_3[1], RNA_pol_Rpb1_R[1], RNA_pol_Rpb1_2[1], RNA_pol_Rpb1_1[1], RNA_pol_Rpb1_5[1], RNA_pol_Rpb1_4[1] | 2.71 |
| POLR2B | RNA_pol_Rpb2_7[1], RNA_pol_Rpb2_4[1], RNA_pol_Rpb2_1[1], RNA_pol_Rpb2_3[1], RNA_pol_Rpb2_5[1], RNA_pol_Rpb2_6[1] | 3.97 |
| POLR2D | RNA_pol_Rpb4[1] | 0.41 |
| POLR2E | RNA_pol_Rpb5_N[1], RNA_pol_Rpb5_C[1] | 0.62 |
| POLR2F | RNA_pol_Rpb6[1] | 2.51 |
| POLR2G | S1[1], SHS2_Rpb7-N[1] | 0 |
| POLR2H | RNA_pol_Rpb8[1] | 1.04 |
| POLR2I | RNA_POL_M_15KD[1], TFIIS_C[1] | 1.25 |
| POLR2J | RNA_pol_L_2[1] | 1.88 |
| POLR2J2 | RNA_pol_L_2[1] | 1.67 |
| POLR2J3 | . | 1.67 |
| POLR2K | DNA_RNApol_7kD[1] | 3.13 |
| POLR2L | RNA_pol_N[1] | 0.2 |
| POLRMT | RPOL_N[1], RNA_pol[1] | 1.67 |
| PPAN | 7tm_1[1], Brix[1] | 0.2 |
| PPIE | RRM_1[1], Pro_isomerase[1] | 0.62 |
| PPIH | Pro_isomerase[1] | 1.04 |
| PPIL3 | Pro_isomerase[1] | 0.41 |
| PPIL4 | RRM_1[1], Pro_isomerase[1] | 1.46 |
| PPRC1 | RRM_1[1] | 3.34 |
| PPWD1 | Pro_isomerase[1], WD40[2] | 1.25 |
| PQBP1 | WW[1] | 0.83 |
| PRDX1 | AhpC-TSA[1], 1-cysPrx_C[1] | 1.67 |
| PRKDC | PI3_PI4_kinase[1] | 7.74 |
| PRPF18 | PRP4[1], Prp18[1] | 1.25 |
| PRPF19 | Prp19[1], U-box[1], WD40[4] | 0.83 |
| PSIP1 | LEDGF[1], PWWP[1] | 3.13 |
| PTBP1 | RRM_5[3], RRM_6[1] | 1.25 |
| PTBP2 | RRM_5[3], RRM_6[1] | 1.67 |
| PTBP3 | RRM_5[2], RRM_1[1], RRM_6[1] | 0.83 |
| PTCD2 | MRP-S27[1] | 1.88 |
| PTCD3 | PPR_2[1], PPR_3[1] | 1.25 |
| PUF60 | RRM_5[1], RRM_1[2] | 5.85 |
| PUM1 | Shadoo[1], PUF[8] | 3.55 |
| PUM2 | PUF[8] | 1.67 |
| PUM3 | CPL[1] | 2.09 |
| PURA | PurA[1] | 1.25 |
| PURB | PurA[1] | 0.41 |
| PURG | PurA[1] | 2.51 |
| PYM1 | Mago-bind[1] | 1.25 |
| QKI | KH_1[1] | 2.71 |
| RAE1 | WD40[3] | 1.88 |
| RALY | RRM_1[1] | 2.3 |
| RALYL | RRM_1[1] | 5.43 |
| RAN | Ras[1] | 0.62 |
| RANBP17 | IBN_N[1], CRM1_C[1] | 5.85 |
| RANBP2 | TPR_1[1], IR1-M[2], zf-RanBP[8], Ran_BP1[4], Pro_isomerase[1] | 6.69 |
| RANBP6 | HEAT_2[2] | 3.13 |
| RAVER1 | RRM_1[1], RRM_6[2] | 2.09 |
| RAVER2 | RRM_1[2], RRM_6[1] | 1.88 |
| RBBP6 | DWNN[1], zf-CCHC[1], zf-C3HC4_2[1] | 4.6 |
| RBFOX1 | Fox-1_C[1], RRM_1[1] | 7.32 |
| RBFOX2 | Fox-1_C[1], RRM_1[1] | 2.09 |
| RBFOX3 | Fox-1_C[1], RRM_1[1] | 4.6 |
| RBM10 | G-patch[1], zf-RanBP[1], RRM_6[2] | 1.88 |
| RBM11 | RRM_1[1] | 4.39 |
| RBM12 | RRM_1[1], RRM_6[4] | 3.76 |
| RBM12B | RRM_6[5] | 5.02 |
| RBM14 | RRM_1[2] | 3.13 |
| RBM15 | RRM_5[2], RRM_1[1], SPOC[1] | 2.3 |
| RBM15B | RRM_5[1], RRM_1[2], SPOC[1] | 1.46 |
| RBM17 | G-patch[1], RRM_5[1] | 0.83 |
| RBM18 | RRM_1[1] | 0.41 |
| RBM20 | RRM_5[1] | 0.62 |
| RBM22 | FYVE[1], RRM_1[1], zf-CCCH[1] | 3.13 |
| RBM23 | RRM_1[2], RBM39linker[1] | 1.67 |
| RBM24 | RRM_6[1] | 5.02 |
| RBM25 | RRM_1[1], PWI[1] | 2.51 |
| RBM26 | RRM_5[1], PWI[1], zf-CCCH[1], RRM_6[1] | 2.71 |
| RBM27 | RRM_5[1], PWI[1], zf-CCCH[1] | 2.92 |
| RBM3 | RRM_1[1] | 0.62 |
| RBM33 | RRM_6[1] | 4.81 |
| RBM34 | RRM_1[2] | 3.76 |
| RBM38 | RRM_1[1] | 2.09 |
| RBM39 | RRM_5[1], RRM_1[2], RBM39linker[1] | 1.88 |
| RBM4 | RRM_1[2], zf-CCHC[1] | 2.09 |
| RBM41 | RRM_1[1] | 0.62 |
| RBM42 | RRM_1[1] | 1.46 |
| RBM43 | RRM_6[1] | 0.62 |
| RBM44 | RRM_1[1] | 2.3 |
| RBM45 | RRM_5[1], RRM_1[3] | 1.04 |
| RBM46 | RRM_1[3], DND1_DSRM[1] | 5.02 |
| RBM47 | RRM_1[3] | 3.76 |
| RBM48 | RRM_5[1] | 1.67 |
| RBM4B | RRM_1[2], zf-CCHC[1] | 2.09 |
| RBM5 | G-patch[1], zf-RanBP[1], RRM_6[2] | 2.71 |
| RBM6 | G-patch[1], RRM_6[2] | 2.51 |
| RBM7 | RRM_1[1] | 3.55 |
| RBM8A | RRM_1[1] | 4.39 |
| RBMS1 | RRM_1[2] | 1.25 |
| RBMS2 | RRM_1[2] | 2.3 |
| RBMS3 | RRM_1[2] | 2.51 |
| RBMX | RBM1CTR[1], RRM_1[1] | 1.88 |
| RBMX2 | RRM_1[1] | 0.62 |
| RBMXL1 | RBM1CTR[1], RRM_1[1] | 2.3 |
| RBMXL2 | RBM1CTR[1], RRM_1[1] | 3.13 |
| RBMXL3 | RRM_1[1] | 0.41 |
| RBMY1A1 | RBM1CTR[1], RRM_1[1] | 0 |
| RBMY1B | RBM1CTR[1], RRM_1[1] | 0 |
| RBMY1D | RBM1CTR[1], RRM_1[1] | 0 |
| RBMY1E | RBM1CTR[1], RRM_1[1] | 0.2 |
| RBMY1F | RBM1CTR[1], RRM_1[1] | 0 |
| RBMY1J | RBM1CTR[1], RRM_1[1] | 0 |
| RBPMS | RRM_1[1] | 1.67 |
| RBPMS2 | RRM_1[1] | 0.62 |
| RC3H1 | zf-CCCH[1], zf-RING_UBOX[1] | 4.81 |
| RC3H2 | zf-RING_5[1], zf-CCCH[1] | 2.51 |
| RNF17 | TUDOR[5] | 9.62 |
| RNGTT | mRNA_cap_C[1], mRNA_cap_enzyme[1], DSPc[1] | 3.13 |
| RNMT | Pox_MCEL[1] | 1.46 |
| RNPS1 | RRM_1[1] | 0.41 |
| RPUSD3 | PseudoU_synth_2[1] | 1.04 |
| RPUSD4 | PseudoU_synth_2[1] | 3.97 |
| RQCD1 | Rcd1[1] | 2.3 |
| RRBP1 | Rib_recp_KP_reg[1], IncA[1] | 1.88 |
| RSRC1 | THRAP3_BCLAF1[1] | 1.88 |
| RTF1 | Plus-3[1], SYF2[1] | 2.09 |
| RUVBL1 | TIP49[1] | 0.62 |
| RUVBL2 | TIP49[1] | 1.25 |
| SAFB | SAP[1], RRM_6[1] | 2.09 |
| SAFB2 | SAP[1], RRM_1[1] | 1.25 |
| SAMD4A | SAM_1[1] | 1.67 |
| SAMD4B | PHAT[1], SAM_1[1] | 1.25 |
| SAP18 | SAP18[1] | 0.83 |
| SARNP | SAP[1] | 0.83 |
| SCAF1 | . | 4.39 |
| SCAF11 | zf-RING_2[1] | 2.71 |
| SCAF4 | RRM_1[1], CTD_bind[1] | 2.09 |
| SCAF8 | RRM_1[1], CTD_bind[1] | 3.76 |
| SECISBP2 | Ribosomal_L7Ae[1] | 1.46 |
| SECISBP2L | Ribosomal_L7Ae[1] | 2.51 |
| SERBP1 | HABP4_PAI-RBP1[1] | 1.04 |
| SETX | AAA_11[1], AAA_12[1] | 3.76 |
| SF1 | zf-CCHC[1], KH_1[1] | 2.3 |
| SF3B1 | HEAT_2[1], SF3b1[1] | 3.34 |
| SF3B14 | RRM_1[1] | 0.62 |
| SF3B2 | SAP[1], DUF382[1], PSP[1] | 3.76 |
| SF3B3 | MMS1_N[1], CPSF_A[1] | 2.09 |
| SF3B4 | RRM_1[2] | 3.34 |
| SF3B5 | SF3b10[1] | 1.67 |
| SFPQ | RRM_1[2], NOPS[1] | 1.46 |
| SFSWAP | Surp[2], DRY_EERY[1] | 2.51 |
| SKIV2L | Helicase_C[1], rRNA_proc-arch[2], DSHCT[1], DEAD[1] | 7.32 |
| SLBP | SLBP_RNA_bind[1] | 1.46 |
| SLTM | SAP[1], RRM_1[1] | 2.71 |
| SLU7 | Slu7[1] | 2.09 |
| SMG1 | PI3_PI4_kinase[1], FATC[1] | 3.55 |
| SMG5 | PIN_4[1], EST1_DNA_bind[2], EST1[1] | 3.55 |
| SMG6 | PIN_4[1], EST1[1], EST1_DNA_bind[1] | 2.51 |
| SMG7 | EST1_DNA_bind[1] | 5.43 |
| SMG8 | DUF2146[2] | 4.6 |
| SMG9 | DUF2146[1] | 0.83 |
| SMNDC1 | SMN[1] | 0.62 |
| SND1 | SNase[5], TUDOR[1] | 6.48 |
| SNW1 | SKIP_SNW[1] | 0.83 |
| SON | G-patch[1], DND1_DSRM[1] | 2.71 |
| SPATS2 | DUF1387[1] | 1.88 |
| SPATS2L | GVQW[1], DUF1387[1] | 1.04 |
| SPEN | RRM_5[1], RRM_1[3], SPOC[1] | 8.78 |
| SREK1 | RRM_1[1], RRM_6[1] | 1.67 |
| SRPK2 | Pkinase[2] | 3.13 |
| SRRM1 | PWI[1] | 3.76 |
| SRRM2 | cwf21[1] | 5.85 |
| SRRM3 | cwf21[1], SRRM_C[1] | 1.67 |
| SRRM4 | SRRM_C[1] | 2.51 |
| SRSF1 | RRM_1[2] | 3.55 |
| SRSF10 | RRM_1[1] | 1.67 |
| SRSF11 | RRM_1[1] | 1.46 |
| SRSF12 | RRM_1[1] | 2.71 |
| SRSF2 | RRM_1[1] | 5.23 |
| SRSF3 | RRM_1[1] | 3.97 |
| SRSF4 | RRM_1[2] | 1.67 |
| SRSF5 | RRM_1[2] | 1.25 |
| SRSF6 | RRM_1[2] | 2.09 |
| SRSF7 | RRM_1[1], zf-CCHC[1] | 1.25 |
| SRSF8 | RRM_1[1] | 2.71 |
| SRSF9 | RRM_1[2] | 0.83 |
| SSU72 | Ssu72[1] | 2.51 |
| STAU1 | dsrm[3] | 3.97 |
| STAU2 | dsrm[4] | 4.18 |
| STRAP | WD40[5] | 1.25 |
| STRBP | dsrm[2], DZF[1] | 1.04 |
| SUB1 | PC4[1] | 3.34 |
| SUGP1 | G-patch[1], Surp[2] | 2.51 |
| SUGP2 | G-patch[1], Surp[2] | 3.97 |
| SUPT4H1 | Spt4[1] | 3.34 |
| SUPT5H | KOW[3], CTD[1], Spt5-NGN[1], Spt5_N[1] | 2.09 |
| SUPT6H | S1[1], DLD[1], SH2_2[1], YqgF[1], HTH_44[1], Tex_N[1], HHH_7[1], SPT6_acidic[1] | 4.39 |
| SUPV3L1 | Helicase_C[1], SUV3_C[1] | 1.25 |
| SWT1 | PIN_4[1] | 4.6 |
| SYF2 | SYF2[1] | 1.46 |
| SYMPK | DUF3453[1], Cohesin_HEAT[1], Symplekin_C[1] | 2.09 |
| SYNCRIP | RRM_1[3] | 3.34 |
| TAF15 | zf-RanBP[1], RRM_1[1] | 1.67 |
| TARBP1 | SpoU_methylase[1] | 6.9 |
| TARBP2 | dsrm[2] | 0 |
| TARDBP | RRM_1[2] | 2.3 |
| TBRG4 | FAST_1[1], FAST_2[1], RAP[1] | 1.46 |
| TCERG1 | FF[6], WW[3] | 3.55 |
| TDRD3 | TUDOR[1], DUF1767[1], UBA[1] | 2.09 |
| TDRD7 | TUDOR[3], OST-HTH[1] | 2.09 |
| TEFM | HHH_3[1] | 0.62 |
| TEX13A | zf-RanBP[1], TEX13[1] | 3.76 |
| TFAM | HMG_box[1], HMG_box_2[1] | 1.88 |
| TFIP11 | G-patch[1], TIP_N[1], GCFC[1] | 2.71 |
| THOC1 | efThoc1[1], Death[1] | 1.25 |
| THOC2 | Tho2[1], Thoc2[1] | 5.02 |
| THOC3 | WD40[4] | 1.67 |
| THOC5 | FimP[1] | 1.67 |
| THOC6 | WD40[1] | 0.62 |
| THOC7 | THOC7[1] | 2.09 |
| THRAP3 | THRAP3_BCLAF1[1] | 3.97 |
| TIA1 | RRM_1[3] | 0.2 |
| TIAL1 | RRM_1[3] | 1.04 |
| TNPO1 | IBN_N[1], HEAT_2[1], HEAT_EZ[1], HEAT[1] | 2.3 |
| TNPO2 | IBN_N[1], HEAT_2[1], Arm[1], HEAT_EZ[1], HEAT[1] | 0.83 |
| TNPO3 | Xpo1[1] | 6.06 |
| TNRC6A | RRM_5[1], Ago_hook[1] | 2.92 |
| TNRC6B | RRM_5[1], Ago_hook[1] | 7.32 |
| TNRC6C | RRM_5[1], M_domain[1], Ago_hook[1] | 8.15 |
| TOP3B | Topoisom_bac[1], Toprim[1] | 2.92 |
| TPR | TPR_MLP1_2[2] | 4.39 |
| TRA2A | RRM_1[1] | 1.67 |
| TRA2B | RRM_1[1] | 1.67 |
| TRIM25 | SPRY[1], PRY[1], zf-C3HC4_4[1], zf-B_box[1] | 3.55 |
| TSFM | EF_TS[1], UBA[1] | 3.13 |
| TTF2 | zf-GRF[1], Helicase_C[1], SNF2_N[1] | 3.97 |
| TUFM | GTP_EFTU_D2[1], GTP_EFTU_D3[1], GTP_EFTU[1] | 1.67 |
| U2AF1 | RRM_5[1], zf-CCCH[2] | 0.41 |
| U2AF1L4 | RRM_5[1], zf-CCCH[2] | 1.25 |
| U2AF2 | RRM_1[2], Transformer[1], RRM_6[1] | 1.25 |
| UBA1 | UBACT[2], UBA_e1_C[1], UBA_e1_thiolCys[1], ThiF[2] | 0.83 |
| UBAP2 | DUF3697[1], UBA[1] | 2.51 |
| UBAP2L | DUF3697[1], UBA[1] | 5.23 |
| UHMK1 | RRM_5[1], Pkinase[1] | 3.34 |
| UPF1 | AAA_11[1], AAA_12[1], UPF1_Zn_bind[1] | 3.13 |
| UPF2 | MIF4G[3], Upf2[1] | 4.6 |
| UPF3A | Smg4_UPF3[1] | 1.88 |
| UPF3B | Smg4_UPF3[1] | 2.3 |
| USP10 | PAM2[1], UCH[1] | 2.71 |
| WBP4 | WW[2], zf-U1[1] | 1.04 |
| WDR61 | WD40[6] | 1.04 |
| WDR83 | WD40[3] | 0.62 |
| XAB2 | TPR_2[1], TPR_12[1] | 1.67 |
| XPO1 | IBN_N[1], Xpo1[1], CRM1_C[1] | 1.04 |
| XPO4 | CRM1_C[1] | 2.3 |
| XPO6 | IBN_N[1], Xpo1[1] | 1.88 |
| XPO7 | IBN_N[1], CRM1_C[1] | 3.34 |
| XRCC6 | SAP[1], Ku[1], Ku_C[1], Ku_N[1] | 3.13 |
| YBX1 | CSD[1] | 1.67 |
| YBX2 | CSD[1] | 2.3 |
| YBX3 | CSD[1] | 2.09 |
| YTHDC1 | YTH[1] | 2.71 |
| YTHDC2 | OB_NTP_bind[1], Ank_2[1], HA2[1], Helicase_C[1], YTH[1], R3H[1], DEAD[1] | 3.97 |
| YTHDF1 | YTH[1] | 5.23 |
| YTHDF2 | YTH[1] | 2.3 |
| YTHDF3 | YTH[1] | 2.51 |
| ZC3H14 | zf-CCCH_2[3] | 1.88 |
| ZC3H3 | zf-CCCH[3] | 9.2 |
| ZCCHC13 | zf-CCHC[4] | 2.09 |
| ZCCHC8 | zf-CCHC[1], PSP[1] | 1.04 |
| ZFP36 | zf-CCCH[2] | 0.83 |
| ZFP36L1 | Tis11B_N[1], zf-CCCH[2] | 0.83 |
| ZFP36L2 | Tis11B_N[1], zf-CCCH[2] | 1.67 |
| ZNF326 | AKAP95[1] | 1.67 |
| ZNF385A | GVQW[1], zf-met[2] | 1.04 |
| ZNF598 | . | 2.3 |
| ZNF638 | RRM_5[2], zf-C2H2_jaz[1], RRM_6[1] | 4.6 |
| ZRANB2 | zf-RanBP[2] | 0.83 |
| ZRSR1 | RRM_5[1], zf-CCCH[2] | 2.71 |
| ZRSR2 | RRM_5[1], zf-CCCH[2] | 0.83 |
