# Supplementary material for: Systems analysis identifies melanoma-enriched pro-oncogenic networks controlled by the RNA binding protein CELF1
Source: Nat Commun. 2017 Dec 21;8:2249. doi: 10.1038/s41467-017-02353-y (PMC5740069; doi:10.1038/s41467-017-02353-y)
Supplement: Supplementary file 4 — Supplementary Data 2 [file 41467_2017_2353_MOESM4_ESM.docx]

| **Node** | **GO Term** | **GO ID** | **#DEG** | **p-value** | **Regulated Genes** |
| --- | --- | --- | --- | --- | --- |
| 1 | Spliceosomal Complex Assembly | GO:0000245 | 11 | 3.89E-14 | **CELF1**, CELF2, DDX39B, MBNL1, PSIP1, SF3A3, SNRNP200, SNRPD1, SNRPD2, SNRPE, SRSF9 |
| 2 | Ribonucleoprotein Complex Biogenesis | GO:0022613 | 19 | 2.37E-12 | AATF, C1QBP, CDKN2A, **CELF1,** CELF2, DDX39B, EIF6, MBNL1, NHP2L1, NOP56, PSIP1, PTEN, SF3A3, SNRNP200, SNRPB, SNRPD1, SNRPD2, SNRPE, SRSF9 |
| 3 | mRNA Splice Site Selection | GO:0006376 | 6 | 2.69E-08 | **CELF1,** CELF2, MBNL1, PSIP1, SF3A3, SRSF9 |
| 4 | Ribonucleoprotein Complex Assembly | GO:0022618 | 14 | 3.14E-11 | C1QBP, **CELF1,** CELF2, DDX39B, EIF6, MBNL1, PSIP1, SF3A3, SNRNP200, SNRPB, SNRPD1, SNRPD2, SNRPE, SRSF9 |
| 5 | mRNA Splicing, via Spliceosome | GO:0000398 | 33 | 2.76E-34 | C1QBP, **CELF1,** CELF2, DDX39B, DDX46, DYRK1A, EFTUD2, HNRNPA1, HNRNPK, LSM7, MAGOH, MBNL1, NHP2L1, NUDT21, PRPF4B, PSIP1, PTBP1, RBM17, SF3A3, SF3B6, SNRNP200, SNRPA1, SNRPB, SNRPD1, SNRPD2, SNRPE, SRRM1, SRSF2, SRSF3, SRSF7, SRSF9, TRA2B, U2AF1 |
| 6 | RNA Splicing | GO:0008380 | 35 | 1.31E-31 | C1QBP, **CELF1,** CELF2, DDX39B, DDX46, DHX15, DYRK1A, EFTUD2, HNRNPA1, HNRNPK, LSM1, LSM7, MAGOH, MBNL1, NHP2L1, NUDT21, PRPF4B, PSIP1, PTBP1, RBM17, SF3A3, SF3B6, SNRNP200, SNRPA1, SNRPB, SNRPD1, SNRPD2, SNRPE, SRRM1, SRSF2, SRSF3, SRSF7, SRSF9, TRA2B, U2AF1 |
| 7 | RNA Processing | GO:0006396 | 42 | 2.23E-28 | C1QBP, CDK9, CDKN2A, **CELF1,** CELF2, DDX39B, DDX46, DHX15, DYRK1A, EFTUD2, HNRNPA1, HNRNPDL, HNRNPK, KHDRBS1, LSM1, LSM7, MAGOH, MBNL1, NHP2L1, NOP56, NUDT21, PABPC4, PRPF4B, PSIP1, PTBP1, RBM17, SF3A3, SF3B6, SMN2, SNRNP200, SNRPA1, SNRPB, SNRPD1, SNRPD2, SNRPE, SRRM1, SRSF2, SRSF3, SRSF7, SRSF9, TRA2B, U2AF1 |
| 8 | mRNA Metabolic Process | GO:0016071 | 39 | 8.12E-29 | ATM, C1QBP, CDK9, **CELF1,** CELF2, DDX39B, DDX46, DHX15, DYRK1A, EFTUD2, HNRNPA1, HNRNPK, KHDRBS1, LSM1, LSM7, MAGOH, MBNL1, NHP2L1, NUDT21, PRPF4B, PSIP1, PTBP1, RBM17, SF3A3, SF3B6, SMN2, SNRNP200, SNRPA1, SNRPB, SNRPD1, SNRPD2, SNRPE, SRRM1, SRSF2, SRSF3, SRSF7, SRSF9, TRA2B, U2AF1 |
| 9 | Spliceosomal snRNP Assembly | GO:0000387 | 4 | 1.34E-04 | SNRPB, SNRPD1, SNRPD2, SNRPE |
| 10 | Histone mRNA Metabolic Process | GO:0008334 | 4 | 2.61E-05 | ATM, LSM1, SNRPB, SNRPE |
| 11 | Alternative mRNA Splicing, via Spliceosome | GO:0000380 | 8 | 7.38E-11 | DYRK1A, HNRNPA1, MAGOH, MBNL1, PTBP1, RBM17, SRSF2, TRA2B |
| 12 | Negative Regulation of mRNA Processing | GO:0050686 | 6 | 3.32E-08 | C1QBP, CDK9, DYRK1A, PTBP1, SRSF7, SRSF9 |
| 13 | Regulation of RNA Splicing | GO:0043484 | 10 | 3.79E-10 | C1QBP, **CELF1,** DYRK1A, MAGOH, MBNL1, PTBP1, SRSF2, SRSF7, SRSF9, TRA2B |
| 14 | Regulation of Alternative mRNA Splicing, via Spliceosome | GO:0000381 | 6 | 2.17E-08 | DYRK1A, MAGOH, MBNL1, PTBP1, SRSF2, TRA2B |
| 15 | Regulation of mRNA Processing | GO:0050684 | 10 | 3.79E-10 | C1QBP, CDK9, DYRK1A, MAGOH, MBNL1, PTBP1, SRSF2, SRSF7, SRSF9, TRA2B |
| 16 | mRNA 3'-End Processing | GO:0031124 | 11 | 4.20E-12 | CDK9, MAGOH, NUDT21, SNRPB, SNRPE, SRRM1, SRSF2, SRSF3, SRSF7, SRSF9, U2AF1 |
| 17 | RNA Export from Nucleus | GO:0006405 | 10 | 2.75E-10 | DDX39B, HNRNPA1, KHDRBS1, MAGOH, SRRM1, SRSF2, SRSF3, SRSF7, SRSF9, U2AF1 |
| 18 | Nucleocytoplasmic Transport | GO:0006913 | 18 | 2.02E-10 | C21orf33, CDH1, CDKN2A, DDX39B, EIF6, FAF1, HNRNPA1, KHDRBS1, LMNA, MAGOH, MAPK3, PTGS2, SRRM1, SRSF2, SRSF3, SRSF7, SRSF9, U2AF1 |
| 19 | Termination of RNA Polymerase II Transcription | GO:0006369 | 10 | 3.91E-13 | MAGOH, NUDT21, SNRPB, SNRPE, SRRM1, SRSF2, SRSF3, SRSF7, SRSF9, U2AF1 |
| 20 | Nuclear Export | GO:0051168 | 12 | 2.13E-10 | CDKN2A, DDX39B, EIF6, HNRNPA1, KHDRBS1, MAGOH, SRRM1, SRSF2, SRSF3, SRSF7, SRSF9, U2AF1 |
| 21 | RNA Localization | GO:0006403 | 11 | 1.72E-08 | DDX39B, HNRNPA1, KHDRBS1, MAGOH, SRRM1, SRSF2, SRSF3, SRSF7, SRSF9, STAU1, U2AF1 |
| 22 | Ribonucleoprotein Complex Export from Nucleus | GO:0071426 | 9 | 5.22E-09 | DDX39B, EIF6, MAGOH, SRRM1, SRSF2, SRSF3, SRSF7, SRSF9, U2AF1 |
| 23 | Negative Regulation of Cell Cycle | GO:0045786 | 21 | 5.77E-12 | ABL1, APBB1, ATM, BUB1B, BUB3, CCNA2, CDK1, CDK9, CDKN2A, CTNNB1, DDX39B, ETS1, FAP, GADD45A, KHDRBS1, MSH2, MYC, PLK1, PLK2, PTEN, PTGS2 |
| 24 | Anaphase-Promoting Complex-Dependent Proteasomal Ubiquitin-Dependent Protein Catabolic Process | GO:0031145 | 7 | 1.01E-05 | ATM, BUB1B, BUB3, CDK1, PLK1, PTEN, SKP2 |
| 25 | Negative Regulation of Mitotic Cell Cycle Phase Transition | GO:1901991 | 7 | 1.13E-04 | ATM, BUB1B, BUB3, CCNA2, CDK1, PLK1, PTEN |
| 26 | Regulation of Cyclin-Dependent Protein Serine/Threonine Kinase Activity | GO:0000079 | 7 | 2.81E-06 | CCNA2, CCNC, CCND3, CDKN2A, GADD45A, PLK1, PTEN |
| 27 | G2/M Transition of Mitotic Cell Cycle | GO:0000086 | 9 | 4.47E-06 | ATM, CCNA2, CDK1, CDKN2A, GADD45A, KHDRBS1, MYBL2, PLK1, SKP2 |
| 28 | Mitochondrial Depolarization | GO:0051882 | 3 | 3.34E-04 | ABL1, CASP1, CDKN2A |
| 29 | Regulation of DNA Damage Response, Signal Transduction By P53 Class Mediator | GO:0043516 | 3 | 1.34E-03 | ATM, CDKN2A, DYRK1A |
| 30 | Regulation of Response to DNA Damage Stimulus | GO:2001020 | 9 | 4.82E-07 | ABL1, ATM, CDK9, CDKN2A, DDX39B, DEK, DYRK1A, MCL1, MYC |
| 31 | Regulation of G2/M Transition of Mitotic Cell Cycle | GO:0010389 | 4 | 4.10E-04 | ATM, CCNA2, CDK1, CDKN2A |
| 32 | Regulation of Ubiquitin-Protein Transferase Activity | GO:0051438 | 8 | 2.03E-06 | ABL1, ATM, BUB1B, BUB3, CDK1, CDKN2A, PLK1, PTEN |
| 33 | Negative Regulation of Mitotic Cell Cycle | GO:0045930 | 11 | 3.75E-07 | ABL1, ATM, BUB1B, BUB3, CCNA2, CDK1, CTNNB1, MSH2, PLK1, PLK2, PTEN |
| 34 | Centrosome Organization | GO:0051297 | 5 | 4.24E-04 | CDK1, CTNNB1, GADD45A, PLK1, PLK2 |
| 35 | Mitotic Nuclear Envelope Disassembly | GO:0007077 | 3 | 2.43E-03 | CDK1, LMNA, PLK1 |
| 36 | Regulation of Ubiquitin-Protein Ligase Activity Involved in Mitotic Cell Cycle | GO:0051439 | 6 | 4.25E-05 | ATM, BUB1B, BUB3, CDK1, PLK1, PTEN |
| 37 | Negative Regulation of Protein Modification By Small Protein Conjugation or Removal | GO:1903321 | 7 | 4.46E-05 | ABL1, ATM, BUB1B, BUB3, CDKN2A, CTNNB1, PLK1 |
| 38 | Regulation of Protein Modification By Small Protein Conjugation or Removal | GO:1903320 | 12 | 2.07E-07 | ABL1, ATM, BUB1B, BUB3, CDK1, CDK9, CDKN2A, CTNNB1, PLK1, PTEN, PTK2, SKP2 |
| 39 | Negative Regulation of Ubiquitin-Protein Transferase Activity | GO:0051444 | 6 | 3.80E-05 | ABL1, ATM, BUB1B, BUB3, CDKN2A, PLK1 |
| 40 | Regulation of Fibroblast Proliferation | GO:0048145 | 4 | 2.06E-03 | CCNA2, CTNNB1, FN1, MYC |
| 41 | Ureteric Bud Development | GO:0001657 | 5 | 5.77E-04 | C21orf33, CTNNB1, FGFR1, MYC, VEGFA |
| 42 | Positive Regulation of Stem Cell Proliferation | GO:2000648 | 6 | 6.22E-06 | CTNNB1, FGFR1, HIF1A, MYC, PDCD2, VEGFA |
| 43 | Neuroblast Proliferation | GO:0007405 | 4 | 4.69E-04 | CTNNB1, FGFR1, HIF1A, VEGFA |
| 44 | Organ Formation | GO:0048645 | 4 | 8.59E-04 | C21orf33, CTNNB1, FGFR1, MAPK3 |
| 45 | Positive Regulation of Mesenchymal Cell Proliferation | GO:0002053 | 4 | 6.47E-05 | CTNNB1, FGFR1, MYC, VEGFA |
| 46 | Telomere Maintenance | GO:0000723 | 4 | 2.06E-03 | ATM, CTNNB1, MYC, PTEN |
| 47 | Adherens Junction Assembly | GO:0034333 | 4 | 1.73E-03 | CTNNB1, PTEN, PTK2, VEGFA |
| 48 | Cell-Substrate Junction Assembly | GO:0007044 | 4 | 2.43E-03 | FN1, PTEN, PTK2, VEGFA |
| 49 | Regulation of Protein Ubiquitination Involved in Ubiquitin-Dependent Protein Catabolic Process | GO:2000058 | 5 | 3.52E-04 | CDK1, CDKN2A, PLK1, PTEN, PTK2 |
| 50 | Positive Regulation of Chromosome Organization | GO:2001252 | 6 | 3.20E-05 | CDK9, CTNNB1, MAPK3, PLK1, PTEN, VEGFA |
| 51 | Regulation of Chromosome Segregation | GO:0051983 | 6 | 1.72E-05 | ATM, BUB1B, BUB3, CTNNB1, PLK1, PTEN |
| 52 | Negative Regulation of Cyclin-Dependent Protein Serine/Threonine Kinase Activity | GO:0045736 | 3 | 1.03E-03 | CDKN2A, PLK1, PTEN |
| 53 | Cell Aging | GO:0007569 | 5 | 4.44E-04 | ABL1, ATM, CDKN2A, LMNA, PTEN |
| 54 | Regulation of Cell-Matrix Adhesion | GO:0001952 | 4 | 3.07E-03 | CDKN2A, PTEN, PTK2, VEGFA |
| 55 | Regulation of Chromosome Organization | GO:0033044 | 10 | 2.29E-06 | ATM, BUB1B, BUB3, CDK9, CTNNB1, MAPK3, MYC, PLK1, PTEN, VEGFA |
| 56 | Positive Regulation of Histone Modification | GO:0031058 | 4 | 1.01E-03 | CDK9, CTNNB1, MAPK3, VEGFA |
| 57 | Positive Regulation of Response to DNA Damage Stimulus | GO:2001022 | 3 | 8.13E-03 | ATM, CDKN2A, MYC |
| 58 | Cellular Component Disassembly Involved in Execution Phase of Apoptosis | GO:0006921 | 8 | 1.89E-08 | AIFM1, CDH1, CDKN2A, CTNNB1, LMNA, LMNB1, PTK2, SPTAN1 |
| 59 | Execution Phase of Apoptosis | GO:0097194 | 11 | 9.31E-11 | AIFM1, CASP1, CASP4, CDH1, CDKN2A, CTNNB1, FAP, LMNA, LMNB1, PTK2, SPTAN1 |
| 60 | Regulation of Mitotic Metaphase/Anaphase Transition | GO:0030071 | 5 | 2.79E-05 | ATM, BUB1B, BUB3, PLK1, PTEN |
| 61 | Multicellular Organismal Aging | GO:0010259 | 4 | 5.10E-05 | ATM, CTSV, MSH2, MSH6 |
| 62 | Somatic Diversification of Immune Receptors via Germline Recombination within a Single Locus | GO:0002562 | 4 | 6.05E-04 | ATM, MSH2, MSH6, POLB |
| 63 | Somatic Hypermutation of Immunoglobulin Genes | GO:0016446 | 3 | 1.02E-04 | MSH2, MSH6, POLB |
| 64 | Mitotic Spindle Assembly Checkpoint | GO:0007094 | 4 | 1.75E-04 | ATM, BUB1B, BUB3, PLK1 |
| 65 | Mitotic Cell Cycle Checkpoint | GO:0007093 | 8 | 1.91E-05 | ATM, BUB1B, BUB3, CCNA2, CDK1, MSH2, PLK1, PLK2 |
| 66 | Negative Regulation of Proteolysis Involved in Cellular Protein Catabolic Process | GO:1903051 | 5 | 1.70E-04 | ATM, BUB1B, BUB3, CDKN2A, PLK1 |
| 67 | Negative Regulation of Cell Division | GO:0051782 | 6 | 2.09E-05 | ATM, BUB1B, BUB3, MSH2, MYC, PLK1 |
| 68 | Meiosis I | GO:0007127 | 4 | 2.63E-03 | ATM, MSH2, MSH6, PLK1 |
| 69 | G2 DNA Damage Checkpoint | GO:0031572 | 4 | 9.96E-05 | ATM, CCNA2, CDK1, PLK1 |
| 70 | Regulation of Execution Phase of Apoptosis | GO:1900117 | 3 | 6.91E-04 | AIFM1, CDKN2A, FAP |
| 71 | Cardiac Muscle Cell Development | GO:0055013 | 3 | 5.41E-03 | LMNA, MYH10, VEGFA |
| 72 | Regulation of Oxidative Stress-induced Intrinsic Apoptotic Signaling Pathway | GO:1902175 | 3 | 1.03E-03 | HIF1A, MCL1, NME5 |
| 73 | TRIF-Dependent Toll-Like Receptor Signaling Pathway | GO:0035666 | 4 | 1.65E-03 | CDK1, FOS, MAP3K7, MAPK3 |
| 74 | Positive Regulation of Viral Life Cycle | GO:1903902 | 4 | 2.06E-03 | CDK9, SSRP1, STAU1, SUPT16H |
| 75 | Regulation of Smooth Muscle Cell Proliferation | GO:0048660 | 4 | 2.73E-03 | CTNNB1, MYD88, PTGS2, SKP2 |
