# Supplementary material for: Systems analysis identifies melanoma-enriched pro-oncogenic networks controlled by the RNA binding protein CELF1
Source: Nat Commun. 2017 Dec 21;8:2249. doi: 10.1038/s41467-017-02353-y (PMC5740069; doi:10.1038/s41467-017-02353-y)
Supplement: Supplementary file 5 — Supplementary Data 3 [file 41467_2017_2353_MOESM5_ESM.docx]

| Cell Line | **Melanoma** | **HeLa** | **HeLa (2)** | **T-Cells** | **K562** | **GM12878** |
| --- | --- | --- | --- | --- | --- | --- |
| Author | **Cifdaloz** | **Rattenbacher** | **LeTonquèze** | **Beisang** | **ENCODE** | **ENCODE** |
| Technique | **RIP-Seq** | **RIP+Microarray** | **CLIP-Seq** | **RIP+Microarray** | **RIP+Microarray** | **RIP+Microarray** |
| n (total) | 2024 | 322 | 1576 | 1131 | 2359 | 147 |
| n (unique) | 952 | 249 | 773 | 494 | 1671 | 77 |

| **Gene Name** | **Melanoma** | **HeLa** | **HeLa (2)** | **T-Cells** | **K562** | **GM12878** |
| --- | --- | --- | --- | --- | --- | --- |
| 7SK | 0 | 0 | 0 | 0 | 1 | 1 |
| AAAS | 0 | 0 | 0 | 0 | 1 | 0 |
| AACS | 0 | 0 | 0 | 0 | 1 | 0 |
| AAGAB | 0 | 0 | 0 | 0 | 1 | 0 |
| AAK1 | 1 | 0 | 0 | 0 | 1 | 0 |
| AAMDC | 0 | 0 | 1 | 0 | 0 | 0 |
| AAMP | 0 | 0 | 0 | 0 | 1 | 0 |
| AARS | 0 | 0 | 1 | 0 | 0 | 0 |
| AASDHPPT | 1 | 0 | 0 | 1 | 0 | 0 |
| ABAT | 0 | 1 | 0 | 0 | 0 | 0 |
| ABCA13 | 0 | 0 | 0 | 0 | 0 | 1 |
| ABCB10 | 0 | 0 | 0 | 0 | 1 | 0 |
| ABCC3 | 0 | 0 | 1 | 0 | 0 | 0 |
| ABCC4 | 0 | 0 | 1 | 0 | 1 | 0 |
| ABCC5 | 0 | 0 | 1 | 0 | 0 | 0 |
| ABCC9 | 1 | 0 | 0 | 0 | 0 | 0 |
| ABCD3 | 0 | 0 | 0 | 1 | 1 | 0 |
| ABCD4 | 0 | 0 | 1 | 0 | 0 | 0 |
| ABCE1 | 0 | 0 | 0 | 0 | 1 | 0 |
| ABCG2 | 0 | 0 | 1 | 0 | 0 | 0 |
| ABHD12 | 1 | 0 | 0 | 0 | 0 | 0 |
| ABHD13 | 0 | 0 | 0 | 1 | 1 | 0 |
| ABHD14B | 0 | 0 | 1 | 1 | 1 | 0 |
| ABHD17B | 0 | 0 | 1 | 0 | 0 | 0 |
| ABHD17C | 0 | 0 | 1 | 0 | 0 | 0 |
| ABHD2 | 1 | 0 | 0 | 0 | 1 | 0 |
| ABI1 | 0 | 0 | 0 | 1 | 1 | 0 |
| ABL2 | 1 | 0 | 0 | 0 | 0 | 0 |
| ABLIM1 | 0 | 0 | 1 | 0 | 0 | 0 |
| ABO | 0 | 1 | 0 | 0 | 0 | 0 |
| ABR | 1 | 0 | 0 | 0 | 0 | 0 |
| ABT1 | 0 | 0 | 0 | 0 | 1 | 0 |
| AC002310.13 | 0 | 0 | 0 | 0 | 1 | 0 |
| AC003029.1 | 0 | 0 | 0 | 0 | 1 | 0 |
| AC004076.9 | 1 | 0 | 0 | 0 | 0 | 1 |
| AC004837.3 | 0 | 0 | 0 | 0 | 1 | 0 |
| AC004917.1 | 0 | 0 | 0 | 0 | 1 | 0 |
| AC004967.7 | 0 | 0 | 0 | 0 | 1 | 0 |
| AC004967.8 | 0 | 0 | 0 | 0 | 1 | 0 |
| AC005082.1 | 0 | 0 | 0 | 0 | 1 | 0 |
| AC005255.3 | 0 | 0 | 0 | 0 | 1 | 0 |
| AC005329.1 | 0 | 0 | 0 | 0 | 0 | 1 |
| AC005375.1 | 0 | 0 | 0 | 0 | 1 | 0 |
| AC005518.2 | 0 | 0 | 0 | 0 | 1 | 0 |
| AC005618.1 | 0 | 0 | 0 | 0 | 1 | 0 |
| AC006026.11 | 0 | 0 | 0 | 0 | 1 | 0 |
| AC006042.8 | 0 | 0 | 0 | 0 | 1 | 0 |
| AC006277.2 | 0 | 0 | 0 | 0 | 1 | 0 |
| AC006465.3 | 1 | 0 | 0 | 0 | 1 | 0 |
| AC006538.4 | 0 | 0 | 0 | 0 | 1 | 0 |
| AC007036.4 | 0 | 0 | 0 | 0 | 1 | 0 |
| AC007036.5 | 0 | 0 | 0 | 0 | 1 | 0 |
| AC007040.11 | 0 | 0 | 0 | 0 | 1 | 0 |
| AC007401.1 | 0 | 0 | 0 | 0 | 1 | 0 |
| AC007401.2 | 1 | 0 | 0 | 0 | 1 | 0 |
| AC007919.1 | 0 | 0 | 0 | 0 | 1 | 0 |
| AC008065.1 | 0 | 0 | 0 | 0 | 1 | 0 |
| AC008536.1 | 0 | 0 | 0 | 0 | 1 | 0 |
| AC009121.1 | 0 | 0 | 0 | 0 | 1 | 0 |
| AC010323.1 | 0 | 0 | 0 | 0 | 1 | 0 |
| AC011933.1 | 0 | 0 | 0 | 0 | 1 | 0 |
| AC011997.1 | 0 | 0 | 0 | 0 | 1 | 0 |
| AC012370.2 | 0 | 0 | 0 | 0 | 1 | 0 |
| AC012615.1 | 0 | 0 | 0 | 0 | 1 | 0 |
| AC013449.1 | 0 | 0 | 0 | 0 | 1 | 0 |
| AC015987.2 | 1 | 0 | 0 | 0 | 0 | 0 |
| AC015989.1 | 0 | 0 | 0 | 0 | 1 | 0 |
| AC017076.5 | 0 | 0 | 0 | 0 | 1 | 0 |
| AC018755.15 | 0 | 0 | 0 | 0 | 1 | 0 |
| AC021205.1 | 0 | 0 | 0 | 0 | 1 | 0 |
| AC021593.1 | 0 | 0 | 0 | 0 | 1 | 0 |
| AC022819.1 | 0 | 0 | 0 | 0 | 1 | 0 |
| AC023818.1 | 0 | 0 | 0 | 0 | 1 | 0 |
| AC024592.12 | 1 | 0 | 0 | 0 | 1 | 0 |
| AC025287.1 | 0 | 0 | 0 | 0 | 0 | 1 |
| AC055876.1 | 0 | 0 | 0 | 0 | 1 | 0 |
| AC063976.6 | 0 | 0 | 0 | 0 | 1 | 0 |
| AC068538.2 | 0 | 0 | 0 | 0 | 1 | 0 |
| AC068580.5 | 0 | 0 | 0 | 0 | 1 | 0 |
| AC068946.1 | 0 | 0 | 0 | 0 | 1 | 0 |
| AC069287.1 | 0 | 0 | 0 | 0 | 1 | 0 |
| AC073063.1 | 0 | 0 | 0 | 0 | 1 | 0 |
| AC073346.2 | 1 | 0 | 0 | 0 | 0 | 0 |
| AC073610.1 | 0 | 0 | 0 | 0 | 1 | 0 |
| AC073610.5 | 1 | 0 | 0 | 0 | 1 | 0 |
| AC073958.2 | 0 | 0 | 0 | 0 | 1 | 0 |
| AC083829.1 | 0 | 0 | 0 | 0 | 1 | 0 |
| AC083899.3 | 0 | 0 | 0 | 0 | 1 | 0 |
| AC087645.1 | 1 | 0 | 0 | 0 | 1 | 0 |
| AC087738.1 | 0 | 0 | 0 | 0 | 1 | 0 |
| AC090286.2 | 0 | 0 | 0 | 0 | 1 | 0 |
| AC090427.1 | 0 | 0 | 0 | 0 | 1 | 0 |
| AC090587.2 | 0 | 0 | 0 | 0 | 1 | 0 |
| AC092620.2 | 0 | 0 | 0 | 0 | 1 | 0 |
| AC093323.3 | 0 | 0 | 0 | 0 | 1 | 0 |
| AC093510.1 | 0 | 0 | 0 | 0 | 1 | 0 |
| AC096633.1 | 0 | 0 | 0 | 0 | 1 | 0 |
| AC096772.6 | 0 | 0 | 0 | 0 | 1 | 0 |
| AC104698.1 | 0 | 0 | 0 | 0 | 1 | 1 |
| AC104841.2 | 1 | 0 | 0 | 0 | 0 | 0 |
| AC105036.1 | 0 | 0 | 0 | 0 | 1 | 0 |
| AC107016.2 | 0 | 0 | 0 | 0 | 0 | 1 |
| AC108456.1 | 0 | 0 | 0 | 0 | 1 | 0 |
| AC114546.1 | 1 | 0 | 0 | 0 | 0 | 0 |
| AC114772.1 | 0 | 0 | 0 | 0 | 1 | 0 |
| AC115115.2 | 0 | 0 | 0 | 0 | 1 | 0 |
| AC119403.1 | 0 | 0 | 0 | 0 | 1 | 0 |
| AC119673.1 | 1 | 0 | 0 | 0 | 1 | 0 |
| AC120194.1 | 1 | 0 | 0 | 0 | 0 | 0 |
| AC138649.1 | 0 | 0 | 0 | 0 | 1 | 0 |
| ACAA2 | 0 | 0 | 0 | 0 | 1 | 0 |
| ACACA | 0 | 0 | 1 | 0 | 0 | 0 |
| ACADM | 0 | 0 | 0 | 0 | 1 | 0 |
| ACAP2 | 0 | 0 | 0 | 1 | 0 | 0 |
| ACAT2 | 1 | 0 | 0 | 0 | 0 | 0 |
| ACBD3 | 1 | 0 | 0 | 0 | 0 | 0 |
| ACBD7 | 1 | 0 | 0 | 0 | 0 | 0 |
| ACER2 | 0 | 0 | 0 | 0 | 1 | 0 |
| ACIN1 | 0 | 0 | 1 | 0 | 0 | 0 |
| ACLY | 1 | 0 | 1 | 0 | 1 | 0 |
| ACOT13 | 1 | 0 | 0 | 0 | 0 | 0 |
| ACOT8 | 0 | 0 | 0 | 0 | 1 | 0 |
| ACOT9 | 0 | 0 | 0 | 0 | 1 | 0 |
| ACOX1 | 0 | 0 | 0 | 0 | 1 | 0 |
| ACR | 0 | 0 | 0 | 0 | 1 | 0 |
| ACSL1 | 0 | 0 | 0 | 1 | 0 | 0 |
| ACSL3 | 1 | 0 | 0 | 0 | 0 | 0 |
| ACSL4 | 1 | 0 | 1 | 0 | 0 | 0 |
| ACSL6 | 0 | 1 | 0 | 0 | 0 | 0 |
| ACSM3 | 1 | 0 | 0 | 0 | 0 | 0 |
| ACSS2 | 0 | 0 | 1 | 0 | 0 | 0 |
| ACTB | 1 | 0 | 1 | 1 | 0 | 0 |
| ACTG1 | 1 | 0 | 0 | 1 | 0 | 0 |
| ACTG2 | 0 | 1 | 0 | 0 | 0 | 0 |
| ACTN1 | 0 | 0 | 1 | 0 | 0 | 0 |
| ACTN4 | 1 | 0 | 0 | 0 | 0 | 0 |
| ACTR1A | 0 | 0 | 0 | 0 | 1 | 0 |
| ACTR2 | 1 | 0 | 0 | 1 | 0 | 0 |
| ACTR3 | 1 | 0 | 0 | 1 | 0 | 0 |
| ACTR8 | 0 | 0 | 0 | 0 | 1 | 0 |
| ACVR1B | 0 | 0 | 0 | 0 | 1 | 0 |
| ACVR2A | 0 | 0 | 0 | 0 | 1 | 0 |
| ACVR2B | 0 | 0 | 0 | 0 | 1 | 0 |
| ACVRL1 | 0 | 0 | 0 | 0 | 1 | 0 |
| ADAM10 | 1 | 0 | 0 | 0 | 0 | 0 |
| ADAM28 | 0 | 1 | 0 | 0 | 0 | 0 |
| ADAM9 | 1 | 0 | 0 | 0 | 1 | 0 |
| ADAMDEC1 | 0 | 1 | 0 | 0 | 0 | 0 |
| ADAMTS10 | 0 | 0 | 1 | 0 | 0 | 0 |
| ADAR | 1 | 0 | 1 | 0 | 0 | 0 |
| ADARB2 | 0 | 0 | 1 | 0 | 0 | 0 |
| ADAT2 | 0 | 0 | 1 | 0 | 0 | 1 |
| ADAT3 | 0 | 0 | 0 | 0 | 1 | 0 |
| ADCK2 | 0 | 0 | 0 | 0 | 1 | 0 |
| ADCK4 | 0 | 1 | 0 | 0 | 0 | 0 |
| ADCY9 | 0 | 0 | 1 | 0 | 0 | 0 |
| ADD3 | 0 | 0 | 0 | 1 | 0 | 0 |
| ADH5 | 1 | 0 | 0 | 0 | 1 | 0 |
| ADH5P4 | 0 | 0 | 0 | 0 | 1 | 0 |
| ADIPOR2 | 0 | 0 | 0 | 0 | 1 | 0 |
| ADK | 0 | 0 | 1 | 0 | 0 | 0 |
| ADNP | 0 | 0 | 1 | 0 | 0 | 0 |
| ADNP2 | 0 | 0 | 0 | 0 | 1 | 0 |
| ADO | 0 | 0 | 0 | 0 | 1 | 0 |
| ADORA2B | 0 | 0 | 0 | 0 | 1 | 0 |
| ADPGK | 1 | 0 | 0 | 0 | 1 | 0 |
| ADPRHL1 | 1 | 0 | 0 | 0 | 0 | 0 |
| ADPRM | 0 | 0 | 0 | 0 | 1 | 0 |
| ADRA2A | 0 | 1 | 0 | 0 | 0 | 0 |
| ADSS | 1 | 0 | 1 | 1 | 1 | 0 |
| ADSSL1 | 0 | 1 | 0 | 0 | 0 | 0 |
| AEBP2 | 0 | 0 | 1 | 1 | 1 | 0 |
| AF222686.1 | 0 | 0 | 0 | 0 | 1 | 0 |
| AFAP1 | 0 | 0 | 1 | 0 | 0 | 0 |
| AFF1 | 1 | 0 | 0 | 1 | 1 | 0 |
| AFF2 | 0 | 1 | 0 | 0 | 0 | 0 |
| AFF4 | 1 | 1 | 0 | 1 | 0 | 0 |
| AFG3L2 | 0 | 0 | 1 | 0 | 0 | 0 |
| AFTPH | 0 | 0 | 0 | 1 | 0 | 0 |
| AGBL5 | 1 | 0 | 0 | 0 | 0 | 0 |
| AGFG1 | 1 | 0 | 0 | 1 | 0 | 0 |
| AGPAT1 | 0 | 0 | 0 | 0 | 1 | 0 |
| AGPAT4 | 0 | 0 | 0 | 0 | 1 | 0 |
| AGPAT5 | 1 | 0 | 0 | 1 | 1 | 0 |
| AGPAT9 | 1 | 0 | 0 | 0 | 1 | 0 |
| AGRN | 0 | 0 | 1 | 0 | 0 | 0 |
| AGXT2L2 | 1 | 0 | 0 | 0 | 0 | 0 |
| AHCTF1 | 0 | 0 | 1 | 0 | 0 | 0 |
| AHCY | 1 | 0 | 0 | 0 | 0 | 0 |
| AHNAK | 1 | 0 | 0 | 0 | 0 | 0 |
| AHR | 0 | 0 | 1 | 0 | 0 | 0 |
| AHRR | 1 | 0 | 0 | 0 | 0 | 0 |
| AIDA | 0 | 0 | 0 | 1 | 1 | 0 |
| AIG1 | 0 | 0 | 0 | 1 | 1 | 0 |
| AJUBA | 0 | 0 | 0 | 0 | 1 | 0 |
| AK2 | 1 | 0 | 0 | 1 | 0 | 0 |
| AK3 | 0 | 0 | 1 | 1 | 0 | 0 |
| AK3P3 | 0 | 0 | 0 | 0 | 1 | 0 |
| AK4 | 1 | 0 | 0 | 0 | 0 | 0 |
| AK4P2 | 0 | 0 | 0 | 0 | 0 | 1 |
| AK7 | 0 | 1 | 0 | 0 | 0 | 0 |
| AKAP11 | 1 | 0 | 1 | 0 | 1 | 0 |
| AKAP11-IT1 | 0 | 0 | 0 | 0 | 1 | 0 |
| AKAP12 | 1 | 0 | 0 | 0 | 0 | 0 |
| AKAP2 | 1 | 0 | 0 | 1 | 0 | 0 |
| AKAP7 | 0 | 0 | 0 | 1 | 0 | 0 |
| AKAP8 | 0 | 0 | 0 | 1 | 0 | 0 |
| AKIP1 | 0 | 0 | 0 | 0 | 1 | 0 |
| AKIRIN1 | 1 | 0 | 0 | 1 | 0 | 0 |
| AKIRIN2 | 0 | 0 | 0 | 1 | 1 | 0 |
| AKNAD1 | 0 | 0 | 1 | 0 | 0 | 0 |
| AKR1B1 | 1 | 0 | 0 | 0 | 0 | 0 |
| AKT2 | 1 | 0 | 0 | 0 | 0 | 0 |
| AKT3 | 0 | 1 | 0 | 0 | 0 | 0 |
| AL009178.1 | 0 | 0 | 0 | 0 | 1 | 0 |
| AL033381.1 | 0 | 0 | 0 | 0 | 0 | 1 |
| AL034548.1 | 0 | 0 | 0 | 0 | 1 | 0 |
| AL109805.1 | 0 | 0 | 0 | 0 | 1 | 0 |
| AL121987.1 | 0 | 0 | 0 | 0 | 1 | 0 |
| AL136303.1 | 0 | 0 | 0 | 0 | 1 | 0 |
| AL138815.1 | 0 | 0 | 0 | 0 | 1 | 0 |
| AL138930.2 | 0 | 0 | 0 | 0 | 1 | 0 |
| AL139385.1 | 0 | 0 | 0 | 0 | 1 | 0 |
| AL139812.1 | 0 | 0 | 0 | 0 | 1 | 0 |
| AL162381.1 | 0 | 0 | 0 | 0 | 1 | 0 |
| AL162407.1 | 0 | 0 | 0 | 0 | 1 | 0 |
| AL162431.1 | 1 | 0 | 0 | 0 | 0 | 0 |
| AL163636.6 | 0 | 0 | 0 | 0 | 1 | 0 |
| AL356475.1 | 0 | 0 | 0 | 0 | 1 | 0 |
| AL590489.1 | 0 | 0 | 0 | 0 | 1 | 0 |
| AL590489.2 | 0 | 0 | 0 | 0 | 1 | 0 |
| AL590822.1 | 0 | 0 | 0 | 0 | 1 | 0 |
| AL591516.3 | 0 | 0 | 0 | 0 | 1 | 0 |
| AL591516.5 | 0 | 0 | 0 | 0 | 1 | 0 |
| AL691523.1 | 0 | 0 | 0 | 0 | 1 | 0 |
| ALDH18A1 | 0 | 1 | 0 | 1 | 0 | 0 |
| ALDH1A3 | 0 | 0 | 1 | 0 | 0 | 0 |
| ALDH3B1 | 0 | 0 | 0 | 0 | 0 | 1 |
| ALDH5A1 | 0 | 0 | 0 | 1 | 0 | 0 |
| ALDH9A1 | 1 | 0 | 0 | 0 | 0 | 0 |
| ALDOA | 1 | 0 | 0 | 1 | 0 | 0 |
| ALG1 | 0 | 0 | 0 | 0 | 1 | 0 |
| ALG11 | 0 | 0 | 0 | 0 | 1 | 1 |
| ALG13 | 0 | 0 | 0 | 1 | 0 | 0 |
| ALG14 | 0 | 0 | 0 | 0 | 1 | 0 |
| ALG2 | 0 | 0 | 0 | 1 | 1 | 0 |
| ALG6 | 0 | 0 | 0 | 0 | 1 | 0 |
| ALG9 | 0 | 0 | 0 | 0 | 1 | 0 |
| ALG9-IT1 | 0 | 0 | 0 | 0 | 1 | 0 |
| ALKBH1 | 0 | 0 | 0 | 0 | 1 | 0 |
| ALKBH5 | 1 | 0 | 0 | 0 | 0 | 0 |
| ALS2CR8 | 1 | 0 | 0 | 0 | 0 | 0 |
| AMD1 | 1 | 0 | 0 | 1 | 1 | 0 |
| AMDHD1 | 0 | 0 | 0 | 0 | 1 | 0 |
| AMFR | 0 | 0 | 0 | 0 | 1 | 0 |
| AMMECR1 | 0 | 0 | 0 | 0 | 1 | 0 |
| AMMECR1-IT1 | 0 | 0 | 0 | 0 | 1 | 0 |
| AMMECR1L | 0 | 0 | 0 | 0 | 1 | 0 |
| AMN1 | 0 | 0 | 0 | 0 | 1 | 0 |
| AMOTL1 | 1 | 0 | 1 | 0 | 0 | 0 |
| AMY2B | 1 | 0 | 0 | 0 | 0 | 0 |
| ANAPC13 | 1 | 0 | 0 | 1 | 1 | 0 |
| ANAPC16 | 0 | 0 | 1 | 0 | 0 | 0 |
| ANAPC2 | 0 | 0 | 1 | 0 | 0 | 0 |
| ANG | 0 | 0 | 0 | 0 | 1 | 0 |
| ANGEL1 | 0 | 0 | 1 | 0 | 1 | 0 |
| ANGEL2 | 0 | 0 | 0 | 1 | 0 | 0 |
| ANKFY1 | 1 | 0 | 1 | 0 | 0 | 0 |
| ANKMY1 | 0 | 1 | 0 | 0 | 0 | 0 |
| ANKRA2 | 0 | 0 | 0 | 1 | 0 | 0 |
| ANKRD10 | 0 | 0 | 1 | 0 | 1 | 0 |
| ANKRD10-IT1 | 0 | 0 | 0 | 0 | 1 | 0 |
| ANKRD11 | 0 | 0 | 1 | 0 | 0 | 1 |
| ANKRD12 | 0 | 0 | 0 | 1 | 0 | 0 |
| ANKRD13C | 0 | 0 | 1 | 1 | 0 | 0 |
| ANKRD17 | 0 | 0 | 1 | 0 | 0 | 0 |
| ANKRD28 | 0 | 0 | 1 | 0 | 0 | 0 |
| ANKRD30BL | 0 | 0 | 1 | 0 | 0 | 0 |
| ANKRD32 | 0 | 0 | 1 | 0 | 0 | 0 |
| ANKRD33 | 0 | 0 | 0 | 0 | 1 | 0 |
| ANKRD37 | 0 | 0 | 0 | 0 | 1 | 0 |
| ANKRD42 | 0 | 0 | 0 | 0 | 1 | 0 |
| ANKRD46 | 0 | 0 | 0 | 0 | 1 | 0 |
| ANKRD52 | 1 | 0 | 0 | 0 | 0 | 0 |
| ANKUB1 | 0 | 0 | 0 | 0 | 0 | 1 |
| ANLN | 1 | 0 | 1 | 0 | 0 | 0 |
| ANO8 | 0 | 0 | 1 | 0 | 0 | 0 |
| ANP32A | 1 | 0 | 1 | 1 | 0 | 0 |
| ANP32E | 0 | 0 | 0 | 1 | 1 | 0 |
| ANTXR1 | 0 | 0 | 1 | 0 | 0 | 0 |
| ANXA1 | 1 | 0 | 0 | 0 | 0 | 0 |
| ANXA2 | 1 | 0 | 0 | 0 | 0 | 0 |
| ANXA2R | 0 | 0 | 0 | 0 | 1 | 0 |
| ANXA5 | 1 | 0 | 0 | 0 | 0 | 0 |
| ANXA7 | 0 | 0 | 0 | 0 | 1 | 0 |
| AP000233.2 | 0 | 0 | 0 | 0 | 1 | 0 |
| AP000350.10 | 1 | 0 | 0 | 0 | 0 | 0 |
| AP000350.4 | 1 | 0 | 0 | 0 | 0 | 0 |
| AP000769.1 | 0 | 0 | 0 | 0 | 1 | 0 |
| AP001052.9 | 0 | 0 | 0 | 0 | 1 | 0 |
| AP003064.2 | 0 | 0 | 0 | 0 | 1 | 0 |
| AP005482.1 | 0 | 0 | 0 | 0 | 1 | 0 |
| AP1AR | 0 | 0 | 0 | 0 | 1 | 0 |
| AP1G1 | 1 | 0 | 0 | 0 | 0 | 0 |
| AP1M1 | 1 | 0 | 1 | 0 | 1 | 0 |
| AP1S1 | 1 | 0 | 0 | 0 | 0 | 0 |
| AP1S2 | 1 | 0 | 0 | 0 | 0 | 0 |
| AP2A2 | 0 | 0 | 0 | 0 | 1 | 0 |
| AP2M1 | 1 | 0 | 0 | 0 | 0 | 0 |
| AP2S1 | 1 | 0 | 0 | 0 | 0 | 0 |
| AP3B1 | 1 | 0 | 0 | 0 | 0 | 0 |
| AP3M1 | 1 | 0 | 0 | 0 | 0 | 0 |
| AP3M2 | 0 | 0 | 0 | 0 | 1 | 0 |
| AP3S1 | 1 | 0 | 0 | 0 | 0 | 0 |
| AP3S2 | 0 | 0 | 1 | 0 | 1 | 0 |
| AP4B1 | 0 | 0 | 0 | 0 | 1 | 0 |
| AP4E1 | 0 | 0 | 0 | 0 | 1 | 0 |
| AP4S1 | 0 | 0 | 0 | 0 | 1 | 0 |
| APAF1 | 0 | 0 | 0 | 0 | 1 | 0 |
| APC | 0 | 0 | 0 | 1 | 0 | 0 |
| APEH | 0 | 0 | 0 | 0 | 1 | 0 |
| APEX2 | 0 | 0 | 0 | 0 | 1 | 0 |
| APH1A | 1 | 0 | 1 | 0 | 1 | 0 |
| APH1B | 0 | 0 | 0 | 0 | 1 | 0 |
| API5 | 1 | 0 | 0 | 1 | 0 | 0 |
| APLNR | 0 | 0 | 0 | 0 | 1 | 0 |
| APLP2 | 1 | 0 | 1 | 1 | 0 | 0 |
| APMAP | 1 | 0 | 0 | 0 | 1 | 0 |
| APOBEC3G | 0 | 0 | 0 | 1 | 0 | 0 |
| APOL6 | 0 | 0 | 1 | 0 | 1 | 0 |
| APP | 1 | 0 | 0 | 0 | 0 | 0 |
| APTX | 1 | 0 | 1 | 0 | 0 | 0 |
| AQP3 | 0 | 0 | 1 | 0 | 1 | 0 |
| ARAF | 0 | 0 | 0 | 0 | 1 | 0 |
| ARAP2 | 0 | 0 | 0 | 1 | 0 | 0 |
| ARCN1 | 1 | 0 | 0 | 1 | 0 | 0 |
| ARF1 | 1 | 0 | 1 | 1 | 0 | 0 |
| ARF1P2 | 0 | 0 | 0 | 0 | 1 | 0 |
| ARF3 | 1 | 0 | 1 | 0 | 1 | 0 |
| ARF4 | 1 | 0 | 0 | 1 | 0 | 0 |
| ARF5 | 0 | 0 | 0 | 0 | 1 | 0 |
| ARF6 | 1 | 0 | 0 | 1 | 0 | 0 |
| ARFGEF2 | 1 | 0 | 0 | 0 | 0 | 0 |
| ARGLU1 | 0 | 0 | 1 | 0 | 0 | 0 |
| ARHGAP11B | 0 | 0 | 0 | 0 | 1 | 0 |
| ARHGAP12 | 1 | 0 | 1 | 0 | 0 | 0 |
| ARHGAP21 | 0 | 0 | 1 | 0 | 0 | 0 |
| ARHGAP22 | 0 | 1 | 0 | 0 | 0 | 0 |
| ARHGAP33 | 0 | 0 | 0 | 0 | 1 | 0 |
| ARHGAP9 | 0 | 0 | 0 | 1 | 0 | 0 |
| ARHGDIA | 1 | 0 | 1 | 0 | 0 | 0 |
| ARHGEF7 | 0 | 0 | 1 | 0 | 0 | 0 |
| ARID4B | 0 | 0 | 0 | 1 | 0 | 0 |
| ARID5B | 1 | 0 | 1 | 1 | 0 | 0 |
| ARIH1 | 1 | 0 | 1 | 1 | 0 | 0 |
| ARIH2 | 1 | 0 | 1 | 1 | 0 | 0 |
| ARL1 | 1 | 0 | 0 | 0 | 0 | 0 |
| ARL10 | 1 | 0 | 0 | 0 | 1 | 0 |
| ARL14 | 0 | 0 | 0 | 0 | 1 | 0 |
| ARL2BP | 1 | 0 | 0 | 0 | 1 | 0 |
| ARL4A | 0 | 0 | 0 | 0 | 1 | 0 |
| ARL4D | 0 | 0 | 1 | 0 | 0 | 0 |
| ARL4P | 0 | 0 | 0 | 0 | 1 | 0 |
| ARL5A | 1 | 0 | 0 | 1 | 0 | 0 |
| ARL5AP3 | 0 | 0 | 0 | 0 | 1 | 0 |
| ARL5B | 0 | 0 | 0 | 0 | 1 | 0 |
| ARL6 | 0 | 0 | 0 | 1 | 0 | 0 |
| ARL6IP1 | 1 | 0 | 1 | 1 | 0 | 0 |
| ARL6IP4 | 0 | 0 | 0 | 1 | 0 | 0 |
| ARL6IP5 | 0 | 0 | 0 | 1 | 1 | 0 |
| ARL6IP6 | 0 | 0 | 0 | 0 | 1 | 0 |
| ARL8A | 0 | 0 | 0 | 0 | 1 | 0 |
| ARL8B | 1 | 0 | 0 | 1 | 0 | 0 |
| ARMC1 | 0 | 0 | 0 | 0 | 1 | 0 |
| ARMC10 | 0 | 0 | 0 | 1 | 0 | 0 |
| ARMC2 | 1 | 0 | 0 | 0 | 0 | 0 |
| ARMC6 | 0 | 0 | 0 | 0 | 1 | 0 |
| ARMCX1 | 0 | 1 | 0 | 0 | 0 | 0 |
| ARMCX5 | 0 | 0 | 0 | 0 | 1 | 0 |
| ARMCX6 | 1 | 0 | 0 | 0 | 0 | 0 |
| ARPC2 | 1 | 0 | 0 | 1 | 0 | 0 |
| ARPC4 | 1 | 0 | 0 | 0 | 0 | 0 |
| ARPC4-TTLL3 | 1 | 0 | 1 | 0 | 0 | 0 |
| ARPC5 | 1 | 0 | 0 | 1 | 0 | 0 |
| ARPC5L | 0 | 0 | 0 | 1 | 1 | 0 |
| ARPP19 | 1 | 0 | 1 | 1 | 1 | 0 |
| ARRB2 | 0 | 0 | 0 | 0 | 1 | 0 |
| ARRDC2 | 0 | 0 | 0 | 1 | 0 | 0 |
| ARRDC3 | 1 | 0 | 0 | 0 | 0 | 0 |
| ARSI | 1 | 0 | 0 | 0 | 0 | 0 |
| ASAH1 | 0 | 0 | 0 | 1 | 0 | 0 |
| ASAH2B | 0 | 0 | 0 | 0 | 1 | 0 |
| ASAP1 | 1 | 0 | 0 | 0 | 0 | 0 |
| ASB1 | 0 | 0 | 0 | 0 | 1 | 1 |
| ASB13 | 0 | 0 | 0 | 0 | 1 | 0 |
| ASB3 | 1 | 0 | 0 | 0 | 0 | 0 |
| ASCC1 | 0 | 0 | 0 | 0 | 1 | 0 |
| ASF1B | 0 | 0 | 0 | 0 | 1 | 0 |
| ASH1L | 1 | 0 | 0 | 0 | 1 | 0 |
| ASH1L-IT1 | 0 | 0 | 0 | 0 | 1 | 0 |
| ASNSD1 | 0 | 0 | 0 | 0 | 1 | 0 |
| ASPH | 1 | 0 | 1 | 0 | 0 | 0 |
| ASPHD2 | 0 | 1 | 0 | 0 | 0 | 0 |
| ASPM | 0 | 0 | 1 | 0 | 0 | 0 |
| ASRGL1 | 0 | 0 | 0 | 0 | 1 | 0 |
| ASS1 | 0 | 0 | 1 | 0 | 0 | 0 |
| ASTN2 | 1 | 0 | 0 | 0 | 0 | 0 |
| ASXL1 | 0 | 0 | 1 | 0 | 0 | 0 |
| ATAD1 | 1 | 0 | 1 | 1 | 1 | 0 |
| ATAD2B | 0 | 0 | 0 | 1 | 0 | 0 |
| ATE1 | 0 | 0 | 1 | 1 | 1 | 0 |
| ATF1 | 1 | 0 | 0 | 1 | 1 | 0 |
| ATF2 | 1 | 0 | 0 | 0 | 0 | 0 |
| ATF4 | 1 | 0 | 1 | 1 | 0 | 0 |
| ATF7 | 0 | 0 | 0 | 0 | 1 | 0 |
| ATF7IP | 1 | 0 | 0 | 0 | 0 | 0 |
| ATG16L1 | 1 | 0 | 0 | 0 | 0 | 0 |
| ATG3 | 0 | 0 | 0 | 1 | 0 | 0 |
| ATG4B | 0 | 0 | 0 | 0 | 1 | 0 |
| ATG7 | 1 | 0 | 1 | 0 | 0 | 0 |
| ATG9A | 0 | 0 | 0 | 0 | 1 | 0 |
| ATL2 | 0 | 0 | 0 | 1 | 0 | 0 |
| ATL3 | 1 | 0 | 0 | 0 | 0 | 0 |
| ATM | 1 | 0 | 0 | 1 | 0 | 0 |
| ATMIN | 1 | 0 | 0 | 1 | 0 | 0 |
| ATN1 | 0 | 0 | 1 | 0 | 0 | 0 |
| ATOX1 | 1 | 0 | 0 | 0 | 0 | 0 |
| ATP11A | 0 | 0 | 1 | 0 | 0 | 0 |
| ATP13A3 | 1 | 0 | 1 | 0 | 0 | 0 |
| ATP13A5 | 0 | 0 | 0 | 0 | 0 | 1 |
| ATP1A1 | 0 | 0 | 1 | 0 | 0 | 0 |
| ATP1B1 | 1 | 0 | 1 | 0 | 1 | 0 |
| ATP1B3 | 1 | 0 | 1 | 0 | 1 | 0 |
| ATP2A2 | 1 | 0 | 1 | 1 | 0 | 0 |
| ATP2C1 | 0 | 0 | 1 | 0 | 0 | 0 |
| ATP5A1 | 1 | 0 | 0 | 0 | 0 | 0 |
| ATP5B | 1 | 0 | 0 | 0 | 0 | 0 |
| ATP5C1 | 1 | 0 | 0 | 1 | 0 | 0 |
| ATP5E | 1 | 0 | 0 | 0 | 0 | 0 |
| ATP5EP2 | 1 | 0 | 0 | 0 | 0 | 0 |
| ATP5F1 | 1 | 0 | 0 | 0 | 0 | 0 |
| ATP5G2 | 1 | 0 | 0 | 1 | 0 | 0 |
| ATP5G3 | 1 | 0 | 0 | 0 | 0 | 0 |
| ATP5I | 1 | 0 | 0 | 1 | 0 | 0 |
| ATP5L | 1 | 0 | 0 | 1 | 0 | 0 |
| ATP5L2 | 0 | 0 | 0 | 0 | 1 | 0 |
| ATP5S | 0 | 0 | 1 | 0 | 1 | 0 |
| ATP5SL | 0 | 0 | 0 | 0 | 1 | 0 |
| ATP6AP1 | 1 | 0 | 1 | 0 | 0 | 0 |
| ATP6AP2 | 1 | 0 | 0 | 1 | 0 | 0 |
| ATP6V0A1 | 0 | 0 | 1 | 0 | 0 | 0 |
| ATP6V0B | 1 | 0 | 1 | 0 | 1 | 0 |
| ATP6V0C | 0 | 0 | 1 | 0 | 0 | 0 |
| ATP6V0D1 | 1 | 0 | 0 | 0 | 1 | 0 |
| ATP6V0E1 | 1 | 0 | 0 | 0 | 0 | 0 |
| ATP6V0E2 | 0 | 0 | 0 | 1 | 1 | 0 |
| ATP6V1A | 1 | 0 | 1 | 0 | 1 | 0 |
| ATP6V1B2 | 1 | 0 | 0 | 0 | 0 | 0 |
| ATP6V1C1 | 1 | 1 | 1 | 1 | 1 | 0 |
| ATP6V1C2 | 1 | 0 | 0 | 0 | 0 | 0 |
| ATP6V1E1 | 1 | 0 | 0 | 0 | 0 | 0 |
| ATP6V1F | 1 | 0 | 0 | 0 | 0 | 0 |
| ATP6V1G1 | 1 | 0 | 0 | 1 | 0 | 0 |
| ATP6V1G1P4 | 0 | 0 | 0 | 0 | 1 | 0 |
| ATP7A | 1 | 0 | 0 | 0 | 1 | 0 |
| ATP8A2 | 0 | 0 | 0 | 0 | 1 | 0 |
| ATP8B2 | 1 | 0 | 0 | 0 | 0 | 0 |
| ATP9A | 1 | 0 | 0 | 0 | 1 | 0 |
| ATPAF1 | 0 | 0 | 0 | 0 | 1 | 0 |
| ATPIF1 | 1 | 0 | 1 | 1 | 0 | 0 |
| ATR | 0 | 0 | 1 | 0 | 0 | 0 |
| ATRAID | 0 | 0 | 0 | 0 | 1 | 0 |
| ATRN | 0 | 0 | 0 | 0 | 1 | 0 |
| ATRX | 1 | 0 | 0 | 0 | 0 | 0 |
| ATXN1 | 0 | 0 | 0 | 1 | 0 | 0 |
| ATXN10 | 0 | 0 | 0 | 0 | 1 | 0 |
| ATXN7 | 1 | 0 | 0 | 0 | 0 | 0 |
| ATXN7L3B | 1 | 0 | 0 | 0 | 0 | 0 |
| AUP1 | 1 | 0 | 0 | 0 | 0 | 0 |
| AURKB | 1 | 0 | 1 | 0 | 0 | 0 |
| AUTS2 | 0 | 0 | 1 | 0 | 0 | 0 |
| AVL9 | 1 | 0 | 0 | 0 | 0 | 0 |
| AXL | 0 | 0 | 1 | 0 | 0 | 0 |
| AZI2 | 0 | 0 | 0 | 1 | 0 | 0 |
| AZIN1 | 1 | 0 | 0 | 0 | 1 | 0 |
| B2M | 1 | 0 | 0 | 0 | 0 | 0 |
| B3GALT6 | 0 | 0 | 0 | 0 | 1 | 0 |
| B3GNT2 | 0 | 0 | 0 | 1 | 0 | 0 |
| B3GNT4 | 1 | 0 | 0 | 0 | 0 | 0 |
| B3GNTL1 | 0 | 1 | 1 | 0 | 0 | 0 |
| B4GALNT4 | 0 | 1 | 0 | 0 | 0 | 0 |
| B4GALT1 | 1 | 0 | 1 | 0 | 1 | 0 |
| B4GALT3 | 0 | 0 | 0 | 0 | 1 | 0 |
| B4GALT5 | 1 | 0 | 1 | 1 | 0 | 0 |
| B4GALT6 | 1 | 0 | 0 | 0 | 0 | 0 |
| B4GALT7 | 0 | 0 | 1 | 0 | 0 | 0 |
| B9D1 | 0 | 0 | 0 | 0 | 1 | 0 |
| BABAM1 | 0 | 0 | 0 | 0 | 1 | 0 |
| BACE2 | 0 | 0 | 1 | 0 | 0 | 0 |
| BACH1 | 1 | 0 | 0 | 0 | 0 | 0 |
| BACH2 | 0 | 0 | 0 | 1 | 0 | 0 |
| BAG1 | 0 | 0 | 1 | 0 | 0 | 0 |
| BAG4 | 1 | 0 | 0 | 0 | 0 | 0 |
| BAG5 | 1 | 0 | 0 | 0 | 1 | 0 |
| BAG6 | 1 | 0 | 0 | 0 | 1 | 0 |
| BAIAP2L1 | 0 | 0 | 1 | 0 | 0 | 0 |
| BANF1 | 0 | 0 | 0 | 1 | 0 | 0 |
| BANP | 0 | 0 | 1 | 0 | 0 | 0 |
| BARD1 | 0 | 0 | 0 | 0 | 1 | 0 |
| BASP1 | 0 | 0 | 1 | 0 | 0 | 0 |
| BAX | 1 | 0 | 0 | 0 | 0 | 0 |
| BAZ1A | 0 | 0 | 0 | 1 | 0 | 0 |
| BAZ1B | 0 | 0 | 1 | 0 | 0 | 0 |
| BAZ2A | 1 | 0 | 0 | 0 | 1 | 1 |
| BBS1 | 0 | 1 | 0 | 0 | 0 | 0 |
| BBS5 | 0 | 0 | 0 | 0 | 1 | 0 |
| BBX | 0 | 0 | 1 | 0 | 0 | 0 |
| BCAP31 | 1 | 0 | 0 | 0 | 1 | 0 |
| BCDIN3D | 0 | 0 | 0 | 1 | 0 | 0 |
| BCL2 | 0 | 0 | 1 | 0 | 0 | 0 |
| BCL2A1 | 0 | 0 | 0 | 1 | 0 | 0 |
| BCL2L11 | 0 | 0 | 0 | 0 | 1 | 0 |
| BCL2L13 | 0 | 0 | 1 | 0 | 0 | 0 |
| BCL2L14 | 1 | 0 | 0 | 0 | 0 | 0 |
| BCL2L2 | 0 | 0 | 0 | 0 | 1 | 0 |
| BCL2L2-PABPN1 | 1 | 0 | 0 | 0 | 1 | 0 |
| BCL9L | 0 | 0 | 1 | 0 | 0 | 0 |
| BCLAF1 | 1 | 0 | 0 | 1 | 0 | 0 |
| BCORL1 | 0 | 0 | 0 | 0 | 1 | 0 |
| BEND3 | 1 | 0 | 0 | 0 | 0 | 0 |
| BEST1 | 1 | 0 | 0 | 0 | 0 | 0 |
| BET1L | 0 | 0 | 0 | 0 | 1 | 0 |
| BEX1 | 0 | 0 | 0 | 0 | 1 | 0 |
| BEX2 | 0 | 0 | 0 | 1 | 0 | 0 |
| BEX4 | 0 | 0 | 0 | 1 | 0 | 0 |
| BFAR | 0 | 0 | 0 | 0 | 1 | 0 |
| BHLHE40 | 0 | 0 | 1 | 1 | 0 | 0 |
| BIN2 | 0 | 0 | 0 | 1 | 0 | 0 |
| BIRC2 | 1 | 0 | 0 | 0 | 1 | 0 |
| BIRC3 | 0 | 0 | 0 | 1 | 0 | 0 |
| BIRC5 | 1 | 0 | 0 | 0 | 1 | 0 |
| BIVM-ERCC5 | 0 | 0 | 1 | 0 | 0 | 0 |
| BLCAP | 1 | 0 | 0 | 1 | 0 | 0 |
| BLMH | 1 | 0 | 0 | 0 | 0 | 0 |
| BLOC1S2 | 0 | 0 | 0 | 0 | 1 | 0 |
| BLOC1S5-TXNDC5 | 1 | 0 | 1 | 0 | 0 | 0 |
| BLOC1S6 | 1 | 0 | 0 | 0 | 0 | 0 |
| BMI1 | 1 | 0 | 0 | 1 | 1 | 0 |
| BMP1 | 0 | 1 | 0 | 0 | 0 | 0 |
| BMP2K | 0 | 0 | 0 | 1 | 0 | 0 |
| BMP6 | 0 | 0 | 1 | 0 | 0 | 0 |
| BMPR1A | 0 | 0 | 1 | 0 | 1 | 0 |
| BMPR1APS2 | 0 | 0 | 0 | 0 | 1 | 0 |
| BMPR2 | 1 | 0 | 0 | 0 | 0 | 0 |
| BNIP2 | 1 | 0 | 0 | 0 | 0 | 0 |
| BNIP3 | 1 | 0 | 0 | 1 | 0 | 0 |
| BOC | 0 | 1 | 0 | 0 | 0 | 0 |
| BOLA2 | 0 | 0 | 0 | 1 | 0 | 0 |
| BOLA2B | 1 | 0 | 0 | 0 | 0 | 0 |
| BRAF | 0 | 0 | 1 | 0 | 0 | 0 |
| BRCA2 | 0 | 0 | 1 | 0 | 0 | 0 |
| BRD4 | 1 | 0 | 0 | 0 | 0 | 0 |
| BRD9 | 0 | 0 | 1 | 0 | 0 | 0 |
| BRE | 0 | 1 | 0 | 0 | 0 | 0 |
| BRF2 | 0 | 0 | 0 | 0 | 1 | 0 |
| BRI3 | 1 | 0 | 0 | 0 | 1 | 0 |
| BRI3BP | 1 | 0 | 0 | 0 | 1 | 0 |
| BRIP1 | 0 | 0 | 1 | 0 | 0 | 0 |
| BRK1 | 1 | 0 | 0 | 0 | 0 | 0 |
| BRWD1 | 1 | 0 | 0 | 0 | 0 | 0 |
| BSG | 1 | 0 | 1 | 0 | 0 | 0 |
| BTBD1 | 1 | 0 | 0 | 0 | 0 | 0 |
| BTBD3 | 0 | 0 | 1 | 0 | 1 | 0 |
| BTBD7 | 1 | 0 | 0 | 0 | 1 | 0 |
| BTF3 | 1 | 0 | 0 | 0 | 0 | 0 |
| BTF3L4 | 1 | 0 | 0 | 1 | 1 | 0 |
| BTF3L4P1 | 0 | 0 | 0 | 0 | 1 | 0 |
| BTG1 | 1 | 0 | 0 | 0 | 0 | 0 |
| BTN3A3 | 0 | 0 | 0 | 1 | 0 | 0 |
| BTRC | 1 | 0 | 0 | 0 | 0 | 0 |
| BUB1 | 0 | 0 | 1 | 0 | 0 | 0 |
| BUB3 | 1 | 0 | 1 | 1 | 0 | 0 |
| BZW1 | 1 | 0 | 0 | 1 | 0 | 0 |
| BZW2 | 1 | 0 | 1 | 1 | 0 | 0 |
| C10orf104 | 0 | 0 | 0 | 1 | 0 | 0 |
| C10orf112 | 0 | 0 | 0 | 0 | 1 | 0 |
| C10orf137 | 0 | 0 | 0 | 1 | 0 | 0 |
| C10orf32 | 0 | 0 | 0 | 0 | 1 | 0 |
| C10orf38 | 0 | 1 | 0 | 0 | 0 | 0 |
| C10orf84 | 0 | 0 | 0 | 1 | 0 | 0 |
| C11orf1 | 0 | 0 | 0 | 1 | 0 | 0 |
| C11orf17 | 0 | 0 | 0 | 1 | 0 | 0 |
| C11orf31 | 1 | 0 | 0 | 1 | 0 | 0 |
| C11orf48 | 0 | 0 | 0 | 1 | 0 | 0 |
| C11orf57 | 0 | 0 | 0 | 1 | 0 | 0 |
| C11orf58 | 1 | 0 | 0 | 0 | 0 | 0 |
| C11orf59 | 0 | 0 | 0 | 1 | 0 | 0 |
| C11orf65 | 1 | 0 | 0 | 0 | 0 | 0 |
| C11orf73 | 0 | 0 | 0 | 1 | 0 | 0 |
| C11orf75 | 0 | 0 | 0 | 0 | 1 | 1 |
| C11orf96 | 0 | 0 | 0 | 0 | 1 | 0 |
| C12orf23 | 1 | 0 | 0 | 1 | 0 | 0 |
| C12orf30 | 0 | 0 | 0 | 1 | 0 | 0 |
| C12orf32 | 0 | 0 | 0 | 1 | 0 | 0 |
| C12orf45 | 0 | 0 | 0 | 1 | 0 | 0 |
| C12orf49 | 0 | 0 | 0 | 0 | 1 | 0 |
| C12orf55 | 0 | 1 | 0 | 0 | 0 | 0 |
| C12orf66 | 0 | 0 | 0 | 0 | 1 | 0 |
| C12orf76 | 0 | 0 | 0 | 0 | 1 | 0 |
| C14orf1 | 0 | 0 | 0 | 1 | 0 | 0 |
| C14orf100 | 0 | 0 | 0 | 1 | 0 | 0 |
| C14orf126 | 0 | 0 | 0 | 1 | 0 | 0 |
| C14orf129 | 0 | 0 | 0 | 1 | 0 | 0 |
| C14orf138 | 0 | 0 | 0 | 1 | 0 | 0 |
| C14orf166 | 1 | 0 | 0 | 1 | 0 | 0 |
| C14orf2 | 1 | 0 | 0 | 1 | 0 | 0 |
| C14orf37 | 0 | 0 | 1 | 0 | 0 | 0 |
| C15orf26 | 0 | 0 | 0 | 0 | 1 | 0 |
| C15orf38-AP3S2 | 0 | 0 | 1 | 0 | 1 | 0 |
| C15orf57 | 0 | 0 | 0 | 0 | 1 | 0 |
| C16orf46 | 0 | 0 | 0 | 0 | 1 | 0 |
| C16orf52 | 1 | 0 | 0 | 0 | 1 | 0 |
| C16orf63 | 0 | 0 | 0 | 1 | 0 | 0 |
| C16orf72 | 1 | 0 | 1 | 0 | 0 | 0 |
| C16orf87 | 0 | 0 | 0 | 0 | 1 | 0 |
| C16orf91 | 0 | 0 | 0 | 0 | 1 | 0 |
| C16orf95 | 0 | 0 | 0 | 0 | 1 | 0 |
| C17orf103 | 1 | 0 | 0 | 0 | 0 | 0 |
| C17orf49 | 0 | 0 | 1 | 1 | 0 | 0 |
| C17orf58 | 0 | 0 | 0 | 0 | 1 | 0 |
| C17orf61-PLSCR3 | 0 | 0 | 0 | 0 | 1 | 0 |
| C17orf62 | 0 | 0 | 1 | 0 | 0 | 0 |
| C17orf79 | 0 | 0 | 0 | 0 | 1 | 0 |
| C17orf80 | 0 | 0 | 0 | 1 | 0 | 0 |
| C17orf81 | 0 | 0 | 0 | 1 | 0 | 0 |
| C18orf1 | 0 | 1 | 0 | 0 | 0 | 0 |
| C18orf10 | 0 | 0 | 0 | 1 | 0 | 0 |
| C18orf25 | 1 | 0 | 1 | 0 | 1 | 0 |
| C18orf30 | 0 | 1 | 0 | 0 | 0 | 0 |
| C18orf32 | 0 | 0 | 0 | 1 | 1 | 0 |
| C18orf54 | 0 | 0 | 0 | 0 | 1 | 0 |
| C18orf56 | 0 | 0 | 0 | 0 | 1 | 0 |
| C19ORF42 | 0 | 0 | 0 | 0 | 1 | 0 |
| C19orf44 | 0 | 1 | 0 | 0 | 0 | 0 |
| C19orf47 | 0 | 0 | 0 | 0 | 1 | 0 |
| C19orf48 | 0 | 0 | 1 | 0 | 0 | 0 |
| C19orf55 | 0 | 0 | 0 | 0 | 1 | 0 |
| C19orf59 | 0 | 0 | 0 | 0 | 1 | 0 |
| C1GALT1 | 0 | 0 | 1 | 0 | 1 | 0 |
| C1orf198 | 0 | 0 | 0 | 0 | 1 | 0 |
| C1orf204 | 0 | 0 | 0 | 0 | 0 | 1 |
| C1orf212 | 0 | 0 | 0 | 0 | 1 | 0 |
| C1orf216 | 1 | 0 | 0 | 0 | 1 | 0 |
| C1orf228 | 1 | 0 | 0 | 0 | 0 | 0 |
| C1orf43 | 1 | 0 | 0 | 1 | 0 | 0 |
| C1orf52 | 0 | 0 | 0 | 1 | 0 | 0 |
| C1orf53 | 0 | 0 | 0 | 0 | 1 | 0 |
| C1orf56 | 1 | 0 | 0 | 0 | 0 | 0 |
| C1orf74 | 0 | 0 | 0 | 0 | 1 | 0 |
| C1orf85 | 0 | 0 | 0 | 0 | 1 | 0 |
| C1orf86 | 0 | 0 | 0 | 0 | 1 | 0 |
| C1orf9 | 0 | 0 | 0 | 1 | 0 | 0 |
| C1QBP | 1 | 0 | 0 | 0 | 0 | 0 |
| C2 | 0 | 0 | 1 | 0 | 0 | 0 |
| C20orf160 | 0 | 1 | 0 | 0 | 0 | 0 |
| C20orf177 | 0 | 0 | 0 | 1 | 0 | 0 |
| C20orf20 | 0 | 0 | 0 | 1 | 0 | 0 |
| C20orf24 | 1 | 0 | 0 | 0 | 0 | 0 |
| C20orf56 | 0 | 1 | 0 | 0 | 0 | 0 |
| C21orf62 | 0 | 1 | 0 | 0 | 0 | 0 |
| C21orf86 | 0 | 1 | 0 | 0 | 0 | 0 |
| C21orf91 | 1 | 0 | 0 | 1 | 0 | 0 |
| C22orf29 | 0 | 0 | 1 | 0 | 0 | 0 |
| C22orf39 | 0 | 0 | 0 | 0 | 1 | 0 |
| C2CD3 | 0 | 0 | 0 | 1 | 0 | 0 |
| C2orf15 | 1 | 0 | 0 | 0 | 0 | 0 |
| C2orf18 | 0 | 1 | 0 | 0 | 1 | 0 |
| C2orf44 | 0 | 0 | 0 | 0 | 1 | 0 |
| C2orf55 | 0 | 1 | 0 | 0 | 0 | 0 |
| C2orf56 | 0 | 0 | 0 | 1 | 0 | 0 |
| C2orf69 | 0 | 0 | 0 | 1 | 0 | 0 |
| C2orf88 | 0 | 0 | 0 | 0 | 1 | 0 |
| C3orf10 | 0 | 0 | 0 | 1 | 0 | 0 |
| C3orf17 | 0 | 0 | 0 | 0 | 1 | 0 |
| C3orf37 | 0 | 0 | 0 | 0 | 1 | 0 |
| C3orf67 | 0 | 0 | 0 | 0 | 1 | 0 |
| C4A | 0 | 0 | 0 | 0 | 1 | 0 |
| C4BPA | 0 | 0 | 1 | 0 | 0 | 0 |
| C4orf3 | 0 | 0 | 0 | 1 | 1 | 0 |
| C4orf30 | 0 | 0 | 0 | 1 | 0 | 0 |
| C4orf32 | 0 | 0 | 0 | 1 | 0 | 0 |
| C4orf34 | 0 | 0 | 0 | 0 | 1 | 0 |
| C4orf46 | 1 | 0 | 0 | 0 | 1 | 0 |
| C5orf15 | 1 | 0 | 0 | 1 | 0 | 0 |
| C5orf22 | 1 | 0 | 0 | 0 | 1 | 0 |
| C5orf24 | 1 | 0 | 1 | 1 | 1 | 0 |
| C5orf28 | 0 | 0 | 0 | 0 | 1 | 0 |
| C5orf30 | 0 | 0 | 0 | 0 | 1 | 0 |
| C5orf45 | 1 | 0 | 0 | 0 | 0 | 0 |
| C5orf51 | 1 | 0 | 1 | 0 | 0 | 0 |
| C5orf55 | 0 | 0 | 0 | 0 | 1 | 0 |
| C6orf1 | 0 | 0 | 0 | 0 | 1 | 0 |
| C6orf106 | 0 | 1 | 0 | 0 | 1 | 0 |
| C6orf130 | 0 | 0 | 0 | 1 | 0 | 0 |
| C6orf162 | 0 | 0 | 0 | 0 | 1 | 0 |
| C6orf165 | 0 | 0 | 0 | 0 | 1 | 0 |
| C6orf192 | 0 | 0 | 0 | 1 | 0 | 0 |
| C6orf204 | 0 | 0 | 0 | 1 | 0 | 0 |
| C6orf57 | 0 | 0 | 0 | 0 | 1 | 0 |
| C6orf62 | 1 | 0 | 0 | 1 | 0 | 0 |
| C6orf70 | 0 | 0 | 0 | 0 | 1 | 0 |
| C7orf41 | 0 | 1 | 0 | 0 | 1 | 0 |
| C7orf43 | 0 | 0 | 0 | 0 | 1 | 0 |
| C7orf49 | 0 | 0 | 0 | 0 | 1 | 0 |
| C7orf55-LUC7L2 | 0 | 0 | 1 | 0 | 0 | 0 |
| C7orf60 | 0 | 0 | 0 | 1 | 1 | 0 |
| C7orf73 | 1 | 0 | 0 | 0 | 0 | 0 |
| C8orf33 | 0 | 0 | 0 | 1 | 0 | 0 |
| C8orf46 | 0 | 1 | 0 | 0 | 0 | 0 |
| C8orf49 | 0 | 0 | 0 | 0 | 1 | 0 |
| C8orf76 | 0 | 0 | 0 | 0 | 1 | 0 |
| C9orf123 | 0 | 0 | 0 | 0 | 1 | 0 |
| C9orf35 | 0 | 0 | 0 | 0 | 1 | 0 |
| C9orf41 | 0 | 0 | 1 | 0 | 1 | 0 |
| C9orf64 | 0 | 0 | 0 | 0 | 1 | 0 |
| C9orf7 | 0 | 1 | 0 | 0 | 0 | 0 |
| C9orf85 | 0 | 0 | 0 | 1 | 1 | 0 |
| CABIN1 | 0 | 0 | 1 | 0 | 0 | 0 |
| CACNB2 | 0 | 1 | 1 | 0 | 0 | 0 |
| CADM1 | 1 | 0 | 0 | 0 | 0 | 0 |
| CADPS | 0 | 0 | 0 | 0 | 1 | 0 |
| CALCRL | 0 | 0 | 0 | 0 | 0 | 1 |
| CALD1 | 1 | 0 | 0 | 0 | 0 | 0 |
| CALM1 | 1 | 0 | 0 | 1 | 0 | 0 |
| CALM2 | 1 | 0 | 1 | 0 | 0 | 0 |
| CALR | 1 | 0 | 0 | 1 | 0 | 0 |
| CALU | 1 | 0 | 1 | 0 | 0 | 0 |
| CAMK2D | 1 | 0 | 0 | 0 | 0 | 0 |
| CAMK2N1 | 0 | 0 | 0 | 0 | 1 | 0 |
| CAMKMT | 0 | 0 | 0 | 0 | 1 | 0 |
| CAMLG | 0 | 0 | 0 | 0 | 1 | 0 |
| CAMSAP1 | 0 | 1 | 0 | 0 | 0 | 0 |
| CAMSAP2 | 1 | 0 | 0 | 0 | 1 | 0 |
| CAMSAP3 | 0 | 0 | 0 | 0 | 1 | 0 |
| CAMTA2 | 0 | 0 | 1 | 0 | 0 | 0 |
| CAND1 | 1 | 0 | 0 | 0 | 0 | 0 |
| CAND2 | 1 | 0 | 0 | 0 | 0 | 0 |
| CANT1 | 0 | 0 | 0 | 0 | 1 | 0 |
| CANX | 1 | 0 | 1 | 1 | 0 | 0 |
| CAP1 | 1 | 0 | 0 | 0 | 0 | 0 |
| CAPN15 | 0 | 0 | 1 | 0 | 0 | 0 |
| CAPN2 | 1 | 0 | 0 | 0 | 0 | 0 |
| CAPNS1 | 0 | 0 | 0 | 1 | 0 | 0 |
| CAPRIN1 | 1 | 0 | 1 | 0 | 0 | 0 |
| CAPZA1 | 1 | 0 | 1 | 1 | 0 | 0 |
| CAPZA2 | 1 | 0 | 0 | 1 | 0 | 0 |
| CARD14 | 0 | 0 | 1 | 0 | 0 | 0 |
| CARD8 | 1 | 0 | 0 | 1 | 0 | 0 |
| CARKD | 0 | 0 | 1 | 0 | 1 | 0 |
| CARS2 | 0 | 0 | 1 | 0 | 0 | 0 |
| CASC3 | 1 | 0 | 0 | 0 | 0 | 0 |
| CASD1 | 1 | 0 | 0 | 1 | 1 | 0 |
| CASP2 | 1 | 0 | 1 | 0 | 0 | 0 |
| CASP3 | 0 | 0 | 0 | 0 | 1 | 0 |
| CASP6 | 0 | 0 | 0 | 1 | 0 | 0 |
| CASP8AP2 | 0 | 0 | 0 | 1 | 0 | 0 |
| CAV1 | 1 | 0 | 0 | 0 | 0 | 0 |
| CAV2 | 1 | 0 | 0 | 0 | 1 | 0 |
| CBFA2T2 | 0 | 1 | 0 | 0 | 0 | 0 |
| CBFB | 1 | 0 | 0 | 1 | 1 | 0 |
| CBL | 1 | 0 | 0 | 0 | 1 | 0 |
| CBLL1 | 0 | 0 | 0 | 1 | 0 | 0 |
| CBR3 | 0 | 0 | 0 | 0 | 1 | 0 |
| CBX1 | 1 | 0 | 1 | 0 | 0 | 0 |
| CBX2 | 0 | 0 | 0 | 0 | 1 | 0 |
| CBX3 | 1 | 0 | 1 | 1 | 0 | 0 |
| CBX5 | 1 | 0 | 0 | 0 | 0 | 0 |
| CBX6 | 1 | 0 | 1 | 0 | 0 | 0 |
| CCAT1 | 0 | 0 | 1 | 0 | 0 | 0 |
| CCBL1 | 0 | 0 | 1 | 0 | 0 | 0 |
| CCBL2 | 1 | 0 | 0 | 1 | 0 | 0 |
| CCDC102B | 0 | 0 | 1 | 0 | 0 | 0 |
| CCDC111 | 0 | 0 | 0 | 0 | 1 | 0 |
| CCDC115 | 0 | 0 | 0 | 0 | 1 | 0 |
| CCDC117 | 0 | 0 | 0 | 1 | 0 | 0 |
| CCDC120 | 0 | 0 | 0 | 0 | 1 | 0 |
| CCDC126 | 0 | 0 | 0 | 0 | 1 | 0 |
| CCDC127 | 0 | 0 | 0 | 0 | 1 | 0 |
| CCDC15 | 0 | 1 | 0 | 0 | 0 | 0 |
| CCDC169 | 0 | 0 | 0 | 0 | 1 | 0 |
| CCDC169-SOHLH2 | 0 | 0 | 1 | 0 | 1 | 0 |
| CCDC18 | 0 | 0 | 1 | 0 | 0 | 0 |
| CCDC25 | 0 | 0 | 0 | 0 | 1 | 0 |
| CCDC50 | 1 | 0 | 1 | 0 | 0 | 0 |
| CCDC51 | 0 | 0 | 0 | 0 | 1 | 0 |
| CCDC57 | 0 | 0 | 1 | 0 | 0 | 0 |
| CCDC58 | 0 | 0 | 1 | 0 | 0 | 0 |
| CCDC69 | 0 | 0 | 0 | 1 | 0 | 0 |
| CCDC71L | 0 | 0 | 0 | 0 | 1 | 0 |
| CCDC73 | 1 | 0 | 0 | 0 | 0 | 0 |
| CCDC90B | 1 | 0 | 0 | 0 | 1 | 0 |
| CCL20 | 0 | 0 | 0 | 0 | 0 | 1 |
| CCL4 | 0 | 0 | 0 | 1 | 0 | 0 |
| CCL5 | 0 | 0 | 0 | 1 | 0 | 0 |
| CCNB1 | 1 | 0 | 0 | 0 | 0 | 0 |
| CCNB1IP1 | 1 | 0 | 0 | 0 | 0 | 0 |
| CCND1 | 1 | 0 | 0 | 0 | 1 | 0 |
| CCNDBP1 | 0 | 0 | 0 | 0 | 1 | 0 |
| CCNG2 | 0 | 0 | 0 | 1 | 1 | 0 |
| CCNI | 1 | 0 | 1 | 0 | 0 | 0 |
| CCNT1 | 1 | 0 | 0 | 0 | 0 | 0 |
| CCNT2 | 0 | 0 | 0 | 1 | 1 | 0 |
| CCNY | 0 | 0 | 0 | 1 | 0 | 0 |
| CCR1 | 0 | 1 | 0 | 0 | 0 | 0 |
| CCR7 | 0 | 0 | 0 | 1 | 0 | 0 |
| CCT2 | 1 | 0 | 0 | 0 | 0 | 0 |
| CCT3 | 1 | 0 | 1 | 1 | 0 | 0 |
| CCT4 | 1 | 0 | 0 | 0 | 0 | 0 |
| CCT5 | 1 | 0 | 0 | 0 | 0 | 0 |
| CCT6A | 1 | 0 | 0 | 1 | 0 | 0 |
| CCT7 | 1 | 0 | 0 | 0 | 0 | 0 |
| CCT8 | 1 | 0 | 0 | 0 | 0 | 0 |
| CD151 | 1 | 0 | 0 | 0 | 0 | 0 |
| CD164 | 1 | 0 | 0 | 1 | 0 | 0 |
| CD164L2 | 0 | 0 | 0 | 0 | 1 | 0 |
| CD1D | 0 | 0 | 0 | 0 | 1 | 0 |
| CD2 | 0 | 0 | 0 | 1 | 0 | 0 |
| CD200 | 0 | 0 | 0 | 1 | 0 | 0 |
| CD247 | 0 | 0 | 0 | 1 | 0 | 0 |
| CD28 | 0 | 0 | 0 | 1 | 0 | 0 |
| CD2AP | 1 | 0 | 0 | 0 | 0 | 0 |
| CD320 | 0 | 0 | 0 | 0 | 1 | 0 |
| CD3E | 0 | 0 | 0 | 1 | 0 | 0 |
| CD3G | 0 | 0 | 0 | 0 | 1 | 0 |
| CD44 | 1 | 0 | 1 | 1 | 0 | 0 |
| CD46 | 1 | 0 | 1 | 0 | 0 | 0 |
| CD47 | 1 | 0 | 0 | 1 | 1 | 1 |
| CD55 | 0 | 0 | 1 | 0 | 0 | 0 |
| CD59 | 1 | 0 | 0 | 0 | 0 | 0 |
| CD6 | 0 | 0 | 0 | 1 | 0 | 0 |
| CD63 | 1 | 0 | 0 | 0 | 0 | 0 |
| CD69 | 0 | 0 | 0 | 1 | 1 | 0 |
| CD81 | 0 | 0 | 0 | 1 | 0 | 0 |
| CD83 | 0 | 0 | 0 | 1 | 0 | 0 |
| CD84 | 0 | 0 | 0 | 0 | 1 | 0 |
| CD9 | 1 | 0 | 1 | 0 | 1 | 0 |
| CD93 | 0 | 1 | 0 | 0 | 0 | 0 |
| CD96 | 0 | 0 | 0 | 1 | 0 | 0 |
| CD97 | 0 | 0 | 0 | 0 | 1 | 0 |
| CD99 | 0 | 0 | 0 | 1 | 0 | 0 |
| CD99L2 | 0 | 0 | 0 | 0 | 1 | 0 |
| CDC123 | 0 | 0 | 0 | 0 | 1 | 0 |
| CDC14A | 0 | 0 | 0 | 1 | 0 | 0 |
| CDC14B | 0 | 0 | 1 | 0 | 0 | 0 |
| CDC16 | 0 | 0 | 1 | 0 | 0 | 0 |
| CDC25A | 0 | 0 | 0 | 0 | 1 | 0 |
| CDC25B | 1 | 0 | 1 | 0 | 1 | 0 |
| CDC27 | 1 | 0 | 1 | 0 | 0 | 0 |
| CDC2L6 | 0 | 0 | 0 | 1 | 0 | 0 |
| CDC34 | 1 | 0 | 0 | 0 | 0 | 0 |
| CDC37L1 | 0 | 0 | 0 | 1 | 0 | 0 |
| CDC42 | 1 | 0 | 0 | 1 | 0 | 0 |
| CDC42BPA | 0 | 0 | 1 | 0 | 1 | 0 |
| CDC42EP3 | 0 | 0 | 0 | 1 | 0 | 0 |
| CDC42EP5 | 1 | 0 | 0 | 0 | 0 | 0 |
| CDC42SE1 | 1 | 0 | 0 | 1 | 0 | 0 |
| CDC42SE2 | 1 | 0 | 0 | 1 | 0 | 0 |
| CDC6 | 0 | 0 | 1 | 0 | 0 | 0 |
| CDC73 | 1 | 0 | 0 | 0 | 0 | 0 |
| CDCA3 | 1 | 0 | 0 | 0 | 0 | 0 |
| CDCA4 | 1 | 0 | 1 | 0 | 1 | 0 |
| CDH24 | 0 | 0 | 1 | 0 | 1 | 0 |
| CDH8 | 1 | 0 | 0 | 0 | 0 | 0 |
| CDHR4 | 0 | 0 | 0 | 0 | 1 | 0 |
| CDIPT | 0 | 0 | 0 | 0 | 1 | 0 |
| CDK1 | 1 | 0 | 1 | 0 | 0 | 0 |
| CDK13 | 0 | 0 | 1 | 0 | 0 | 0 |
| CDK14 | 1 | 0 | 0 | 0 | 0 | 0 |
| CDK17 | 1 | 0 | 1 | 0 | 0 | 0 |
| CDK2 | 1 | 0 | 0 | 0 | 0 | 0 |
| CDK2AP1 | 1 | 0 | 0 | 1 | 0 | 0 |
| CDK4 | 1 | 0 | 0 | 0 | 0 | 0 |
| CDK5R1 | 1 | 0 | 0 | 0 | 0 | 0 |
| CDK5RAP2 | 0 | 0 | 1 | 0 | 0 | 0 |
| CDK6 | 1 | 0 | 1 | 0 | 0 | 0 |
| CDKAL1 | 0 | 0 | 1 | 0 | 0 | 0 |
| CDKN1A | 1 | 0 | 0 | 0 | 0 | 0 |
| CDKN1B | 0 | 0 | 0 | 0 | 1 | 0 |
| CDKN2A | 0 | 0 | 1 | 0 | 0 | 0 |
| CDKN2AIP | 0 | 0 | 0 | 1 | 0 | 0 |
| CDKN2B-AS1 | 0 | 0 | 1 | 0 | 0 | 0 |
| CDKN2C | 0 | 0 | 0 | 0 | 1 | 0 |
| CDKN2D | 0 | 0 | 0 | 0 | 1 | 0 |
| CDPF1 | 0 | 0 | 0 | 0 | 1 | 0 |
| CDS2 | 1 | 0 | 0 | 0 | 1 | 0 |
| CDV3 | 1 | 0 | 0 | 1 | 0 | 0 |
| CDYL | 0 | 0 | 1 | 0 | 0 | 0 |
| CDYL2 | 0 | 0 | 1 | 0 | 0 | 0 |
| CEACAM6 | 0 | 1 | 0 | 0 | 0 | 0 |
| CEACAM8 | 1 | 0 | 0 | 0 | 0 | 0 |
| CEBPB | 1 | 0 | 1 | 1 | 1 | 0 |
| CEBPE | 0 | 1 | 0 | 0 | 0 | 0 |
| CEBPG | 1 | 0 | 0 | 0 | 0 | 0 |
| CELF1 | 1 | 0 | 1 | 0 | 0 | 0 |
| CELF2 | 1 | 0 | 0 | 0 | 0 | 0 |
| CELSR1 | 0 | 0 | 1 | 0 | 0 | 0 |
| CELSR3 | 0 | 0 | 1 | 0 | 0 | 0 |
| CENPA | 0 | 0 | 0 | 0 | 1 | 0 |
| CENPH | 0 | 0 | 1 | 0 | 0 | 0 |
| CENPI | 1 | 0 | 1 | 0 | 0 | 0 |
| CENPK | 0 | 0 | 1 | 0 | 0 | 0 |
| CENPM | 0 | 0 | 1 | 0 | 0 | 0 |
| CENPP | 0 | 0 | 1 | 0 | 0 | 0 |
| CEP120 | 0 | 0 | 1 | 0 | 0 | 0 |
| CEP128 | 0 | 0 | 1 | 0 | 0 | 0 |
| CEP135 | 0 | 0 | 0 | 1 | 0 | 0 |
| CEP192 | 0 | 0 | 1 | 0 | 0 | 0 |
| CEP72 | 0 | 0 | 1 | 0 | 0 | 0 |
| CEP76 | 0 | 0 | 0 | 0 | 1 | 0 |
| CEPT1 | 0 | 0 | 0 | 0 | 1 | 0 |
| CER1 | 0 | 1 | 0 | 0 | 0 | 0 |
| CERK | 0 | 0 | 1 | 1 | 1 | 0 |
| CERS2 | 1 | 0 | 0 | 0 | 0 | 0 |
| CERS5 | 1 | 0 | 0 | 0 | 1 | 0 |
| CERS6 | 1 | 0 | 0 | 0 | 1 | 0 |
| CES2 | 0 | 0 | 0 | 0 | 1 | 0 |
| CETN3 | 0 | 0 | 0 | 0 | 1 | 0 |
| CFDP1 | 0 | 0 | 0 | 0 | 1 | 0 |
| CFL1 | 1 | 0 | 1 | 0 | 0 | 0 |
| CFL1P3 | 0 | 0 | 0 | 0 | 1 | 0 |
| CFL2 | 0 | 0 | 0 | 0 | 1 | 0 |
| CFTR | 1 | 0 | 0 | 0 | 0 | 0 |
| CGGBP1 | 1 | 0 | 0 | 1 | 1 | 0 |
| CHAC1 | 0 | 0 | 1 | 0 | 1 | 0 |
| CHAD | 0 | 1 | 0 | 0 | 0 | 0 |
| CHAMP1 | 0 | 0 | 0 | 0 | 1 | 0 |
| CHCHD2 | 1 | 0 | 1 | 0 | 0 | 0 |
| CHCHD2P6 | 0 | 0 | 0 | 0 | 1 | 0 |
| CHCHD2P8 | 0 | 0 | 0 | 0 | 1 | 0 |
| CHCHD2P9 | 0 | 0 | 0 | 0 | 1 | 0 |
| CHCHD4 | 0 | 0 | 0 | 0 | 1 | 0 |
| CHD1 | 0 | 0 | 0 | 1 | 0 | 0 |
| CHD2 | 0 | 0 | 1 | 0 | 0 | 0 |
| CHD3 | 1 | 0 | 0 | 0 | 0 | 0 |
| CHD8 | 0 | 0 | 1 | 0 | 0 | 0 |
| CHD9 | 1 | 0 | 1 | 0 | 0 | 0 |
| CHERP | 0 | 0 | 0 | 1 | 0 | 0 |
| CHIC2 | 0 | 0 | 0 | 0 | 1 | 0 |
| CHML | 0 | 0 | 0 | 0 | 1 | 0 |
| CHMP1A | 0 | 0 | 0 | 0 | 1 | 0 |
| CHMP2B | 0 | 0 | 0 | 0 | 1 | 0 |
| CHMP3 | 0 | 0 | 0 | 0 | 1 | 0 |
| CHMP4B | 0 | 1 | 0 | 0 | 0 | 0 |
| CHMP4BP1 | 0 | 0 | 0 | 0 | 1 | 0 |
| CHMP5 | 0 | 0 | 0 | 1 | 0 | 0 |
| CHORDC1 | 1 | 0 | 1 | 1 | 0 | 0 |
| CHP1 | 1 | 0 | 1 | 0 | 0 | 0 |
| CHPT1 | 0 | 0 | 0 | 1 | 0 | 0 |
| CHRM1 | 0 | 1 | 0 | 0 | 0 | 0 |
| CHRNA2 | 0 | 1 | 0 | 0 | 0 | 0 |
| CHRNA5 | 0 | 0 | 0 | 0 | 1 | 0 |
| CHST10 | 0 | 0 | 0 | 0 | 1 | 0 |
| CHST11 | 0 | 0 | 0 | 1 | 0 | 0 |
| CHSY1 | 1 | 0 | 1 | 1 | 1 | 0 |
| CHTOP | 1 | 0 | 1 | 0 | 0 | 0 |
| CHUK | 0 | 0 | 0 | 1 | 0 | 0 |
| CHURC1 | 0 | 0 | 0 | 0 | 1 | 0 |
| CHURC1-FNTB | 0 | 0 | 0 | 0 | 1 | 0 |
| CIAO1 | 0 | 0 | 0 | 0 | 1 | 0 |
| CIAPIN1 | 0 | 0 | 0 | 0 | 1 | 0 |
| CIB1 | 0 | 0 | 0 | 0 | 1 | 0 |
| CIRBP | 0 | 0 | 0 | 1 | 0 | 0 |
| CISD1 | 0 | 0 | 0 | 0 | 1 | 0 |
| CISD2 | 1 | 0 | 0 | 1 | 1 | 0 |
| CISH | 0 | 0 | 0 | 0 | 1 | 0 |
| CIT | 1 | 0 | 0 | 0 | 0 | 0 |
| CITED2 | 0 | 0 | 1 | 0 | 1 | 0 |
| CKAP2 | 0 | 0 | 1 | 0 | 0 | 0 |
| CKAP4 | 1 | 0 | 1 | 0 | 0 | 0 |
| CKAP5 | 0 | 0 | 0 | 0 | 1 | 0 |
| CKLF | 0 | 0 | 0 | 0 | 1 | 0 |
| CKLF-CMTM1 | 0 | 0 | 0 | 0 | 1 | 0 |
| CKS1B | 1 | 0 | 0 | 0 | 0 | 0 |
| CKS2 | 1 | 0 | 0 | 0 | 0 | 0 |
| CLASP1 | 1 | 0 | 0 | 1 | 0 | 0 |
| CLASP2 | 1 | 0 | 0 | 0 | 0 | 0 |
| CLCC1 | 0 | 0 | 1 | 0 | 0 | 0 |
| CLCN5 | 0 | 0 | 0 | 0 | 1 | 0 |
| CLCN7 | 0 | 0 | 1 | 0 | 0 | 0 |
| CLDN6 | 0 | 1 | 0 | 0 | 0 | 0 |
| CLDN7 | 0 | 0 | 0 | 0 | 1 | 0 |
| CLDND1 | 0 | 0 | 0 | 1 | 1 | 0 |
| CLEC1B | 0 | 0 | 0 | 0 | 1 | 0 |
| CLEC2B | 0 | 0 | 0 | 0 | 1 | 0 |
| CLEC3B | 0 | 0 | 1 | 0 | 0 | 0 |
| CLEC7A | 0 | 1 | 0 | 1 | 0 | 0 |
| CLIC1 | 1 | 0 | 0 | 1 | 0 | 0 |
| CLIC4 | 1 | 0 | 1 | 0 | 0 | 0 |
| CLINT1 | 1 | 0 | 0 | 0 | 0 | 0 |
| CLIP4 | 1 | 0 | 0 | 0 | 0 | 0 |
| CLN6 | 1 | 0 | 0 | 0 | 1 | 0 |
| CLN8 | 0 | 0 | 0 | 0 | 1 | 0 |
| CLNS1A | 1 | 0 | 0 | 0 | 0 | 0 |
| CLP1 | 0 | 0 | 0 | 0 | 1 | 0 |
| CLPTM1 | 0 | 0 | 0 | 0 | 1 | 0 |
| CLPTM1L | 1 | 0 | 1 | 0 | 1 | 0 |
| CLPX | 0 | 0 | 0 | 0 | 1 | 0 |
| CLTA | 1 | 0 | 1 | 0 | 1 | 0 |
| CLTC | 1 | 0 | 1 | 0 | 0 | 0 |
| CMAH | 0 | 0 | 0 | 1 | 0 | 0 |
| CMAS | 1 | 0 | 0 | 0 | 0 | 0 |
| CMBL | 0 | 0 | 1 | 0 | 0 | 0 |
| CMC2 | 0 | 0 | 1 | 0 | 0 | 0 |
| CMIP | 1 | 0 | 1 | 0 | 1 | 0 |
| CMPK1 | 1 | 0 | 0 | 0 | 0 | 0 |
| CMPK2 | 0 | 0 | 0 | 0 | 1 | 0 |
| CMSS1 | 0 | 0 | 1 | 0 | 0 | 0 |
| CMTM1 | 0 | 0 | 0 | 0 | 1 | 0 |
| CMTM4 | 1 | 0 | 0 | 0 | 1 | 0 |
| CMTM6 | 1 | 0 | 0 | 1 | 1 | 0 |
| CMTM7 | 1 | 0 | 0 | 0 | 0 | 0 |
| CNBP | 1 | 0 | 1 | 1 | 0 | 0 |
| CNGA4 | 1 | 0 | 0 | 0 | 0 | 0 |
| CNGB3 | 1 | 0 | 0 | 0 | 0 | 0 |
| CNIH | 1 | 0 | 0 | 0 | 0 | 0 |
| CNIH4 | 0 | 1 | 0 | 0 | 1 | 0 |
| CNN3 | 1 | 0 | 0 | 0 | 0 | 0 |
| CNNM2 | 0 | 0 | 1 | 0 | 0 | 0 |
| CNNM3 | 0 | 0 | 0 | 0 | 1 | 0 |
| CNO | 0 | 0 | 0 | 1 | 0 | 0 |
| CNOT1 | 0 | 0 | 0 | 0 | 1 | 0 |
| CNOT4 | 0 | 0 | 1 | 0 | 0 | 0 |
| CNOT6 | 1 | 0 | 0 | 1 | 1 | 0 |
| CNOT6L | 1 | 0 | 0 | 0 | 1 | 0 |
| CNOT6LP1 | 0 | 0 | 0 | 0 | 1 | 0 |
| CNOT7 | 0 | 0 | 0 | 1 | 0 | 0 |
| CNOT8 | 0 | 0 | 0 | 1 | 0 | 0 |
| CNPY2 | 1 | 0 | 0 | 0 | 0 | 0 |
| CNR1 | 0 | 0 | 0 | 0 | 1 | 0 |
| CNRIP1 | 1 | 0 | 0 | 0 | 0 | 0 |
| CNTN5 | 0 | 0 | 0 | 0 | 0 | 1 |
| CNTNAP1 | 0 | 1 | 0 | 0 | 0 | 0 |
| CNTNAP2 | 0 | 0 | 0 | 0 | 1 | 0 |
| CNTNAP5 | 0 | 1 | 0 | 0 | 0 | 0 |
| COA1 | 0 | 0 | 1 | 0 | 1 | 0 |
| COA3 | 0 | 0 | 0 | 0 | 1 | 0 |
| COBL | 0 | 0 | 1 | 0 | 0 | 0 |
| COG5 | 0 | 0 | 1 | 0 | 0 | 0 |
| COL12A1 | 0 | 0 | 1 | 0 | 0 | 0 |
| COL4A5 | 0 | 0 | 1 | 0 | 0 | 0 |
| COL5A1 | 0 | 0 | 1 | 0 | 0 | 0 |
| COL6A1 | 0 | 1 | 0 | 0 | 0 | 0 |
| COL6A2 | 1 | 0 | 0 | 0 | 0 | 0 |
| COLGALT1 | 1 | 0 | 0 | 0 | 0 | 0 |
| COMMD10 | 1 | 0 | 0 | 0 | 0 | 0 |
| COMMD5 | 0 | 0 | 0 | 0 | 1 | 0 |
| COPA | 1 | 0 | 0 | 0 | 0 | 0 |
| COPB1 | 1 | 0 | 0 | 1 | 0 | 0 |
| COPB2 | 1 | 0 | 0 | 0 | 0 | 0 |
| COPRS | 1 | 0 | 0 | 0 | 0 | 0 |
| COPS7A | 1 | 0 | 0 | 1 | 1 | 0 |
| COPS8 | 1 | 0 | 0 | 0 | 0 | 0 |
| COPZ1 | 1 | 0 | 1 | 0 | 0 | 0 |
| COQ10A | 0 | 0 | 0 | 0 | 1 | 0 |
| COQ2 | 0 | 0 | 0 | 0 | 1 | 0 |
| CORO1C | 1 | 0 | 0 | 0 | 0 | 0 |
| CORO7-PAM16 | 0 | 0 | 1 | 0 | 0 | 0 |
| COTL1 | 1 | 0 | 0 | 0 | 0 | 0 |
| COX11 | 0 | 0 | 1 | 0 | 0 | 0 |
| COX16 | 0 | 0 | 0 | 0 | 1 | 0 |
| COX19 | 0 | 0 | 0 | 0 | 1 | 0 |
| COX4I1 | 1 | 0 | 0 | 0 | 0 | 0 |
| COX5B | 1 | 0 | 0 | 0 | 0 | 0 |
| COX5BP6 | 0 | 0 | 0 | 0 | 1 | 0 |
| COX6A1 | 1 | 0 | 0 | 1 | 0 | 0 |
| COX6B1 | 1 | 0 | 0 | 0 | 0 | 0 |
| COX6C | 1 | 0 | 0 | 0 | 0 | 0 |
| COX7A2 | 1 | 0 | 0 | 0 | 0 | 0 |
| COX7A2L | 1 | 0 | 0 | 1 | 0 | 0 |
| COX7A2P2 | 0 | 0 | 0 | 0 | 1 | 0 |
| COX7B | 1 | 0 | 0 | 0 | 0 | 0 |
| COX7C | 1 | 0 | 1 | 0 | 0 | 0 |
| COX8A | 1 | 0 | 0 | 0 | 0 | 0 |
| CPA4 | 0 | 0 | 1 | 0 | 0 | 0 |
| CPAMD8 | 0 | 1 | 0 | 0 | 0 | 0 |
| CPEB2 | 0 | 0 | 1 | 0 | 1 | 0 |
| CPEB3 | 0 | 0 | 0 | 0 | 1 | 1 |
| CPEB3_ribozyme | 0 | 0 | 0 | 0 | 1 | 1 |
| CPEB4 | 0 | 0 | 0 | 0 | 1 | 0 |
| CPM | 1 | 0 | 0 | 0 | 0 | 0 |
| CPNE3 | 1 | 0 | 0 | 0 | 1 | 0 |
| CPNE7 | 0 | 0 | 1 | 0 | 0 | 0 |
| CPNE8 | 1 | 0 | 0 | 0 | 0 | 0 |
| CPOX | 1 | 0 | 0 | 0 | 1 | 0 |
| CPS1 | 0 | 0 | 1 | 0 | 0 | 0 |
| CPSF3L | 0 | 0 | 1 | 0 | 0 | 0 |
| CPSF4 | 0 | 0 | 0 | 0 | 1 | 0 |
| CPSF6 | 1 | 0 | 0 | 0 | 0 | 0 |
| CPT1A | 0 | 0 | 0 | 0 | 1 | 0 |
| CRADD | 0 | 0 | 0 | 0 | 1 | 0 |
| CRAMP1L | 0 | 0 | 1 | 0 | 0 | 0 |
| CRBN | 0 | 0 | 0 | 1 | 1 | 0 |
| CREB1 | 0 | 0 | 0 | 1 | 0 | 0 |
| CREB3L2 | 1 | 0 | 1 | 0 | 0 | 0 |
| CREB5 | 1 | 0 | 0 | 0 | 0 | 0 |
| CREBBP | 0 | 0 | 1 | 0 | 0 | 0 |
| CREBL2 | 1 | 0 | 0 | 0 | 0 | 0 |
| CREM | 0 | 0 | 0 | 1 | 1 | 0 |
| CRIM1 | 1 | 0 | 0 | 0 | 0 | 0 |
| CRIP2 | 1 | 0 | 1 | 0 | 0 | 0 |
| CRIPAK | 1 | 0 | 0 | 0 | 0 | 0 |
| CRIPT | 0 | 0 | 0 | 0 | 1 | 0 |
| CRK | 1 | 0 | 0 | 0 | 1 | 0 |
| CRKL | 1 | 0 | 1 | 0 | 0 | 0 |
| CRKRS | 0 | 1 | 0 | 0 | 0 | 0 |
| CRLF3 | 0 | 0 | 0 | 1 | 1 | 0 |
| CRLS1 | 0 | 0 | 1 | 0 | 1 | 0 |
| CROCCL1 | 0 | 1 | 0 | 0 | 0 | 0 |
| CRTAM | 0 | 0 | 0 | 1 | 0 | 0 |
| CRTAP | 1 | 0 | 1 | 0 | 0 | 0 |
| CRTC3 | 0 | 0 | 1 | 0 | 0 | 0 |
| CRY2 | 0 | 0 | 0 | 0 | 1 | 0 |
| CRYM | 0 | 0 | 0 | 0 | 1 | 0 |
| CSDA | 0 | 0 | 0 | 1 | 0 | 0 |
| CSDE1 | 1 | 0 | 0 | 1 | 0 | 0 |
| CSE1L | 1 | 0 | 0 | 0 | 0 | 0 |
| CSF1R | 0 | 1 | 0 | 0 | 0 | 0 |
| CSF2RB | 0 | 0 | 0 | 1 | 0 | 0 |
| CSGALNACT1 | 1 | 0 | 0 | 0 | 0 | 0 |
| CSH1 | 0 | 1 | 0 | 0 | 0 | 0 |
| CSK | 1 | 0 | 0 | 0 | 0 | 0 |
| CSNK1A1 | 1 | 0 | 0 | 1 | 0 | 0 |
| CSNK1D | 0 | 0 | 1 | 0 | 0 | 0 |
| CSNK1E | 1 | 1 | 1 | 0 | 1 | 0 |
| CSNK1G3 | 0 | 0 | 0 | 1 | 0 | 0 |
| CSNK2A2 | 1 | 0 | 0 | 1 | 0 | 0 |
| CSPG5 | 0 | 0 | 0 | 0 | 1 | 0 |
| CSRNP1 | 0 | 0 | 0 | 0 | 1 | 0 |
| CSRP2BP | 0 | 0 | 0 | 0 | 1 | 0 |
| CSTF1 | 0 | 0 | 0 | 0 | 1 | 0 |
| CSTF3 | 0 | 1 | 0 | 0 | 0 | 0 |
| CTB-111H14.1 | 0 | 0 | 0 | 0 | 1 | 0 |
| CTB-131B5.2 | 0 | 0 | 0 | 0 | 1 | 0 |
| CTB-96E2.3 | 0 | 0 | 0 | 0 | 1 | 0 |
| CTBP1 | 1 | 0 | 0 | 1 | 1 | 0 |
| CTBP1-AS1 | 0 | 0 | 0 | 0 | 1 | 0 |
| CTBP2 | 1 | 0 | 1 | 0 | 0 | 0 |
| CTC-203F4.1 | 1 | 0 | 0 | 0 | 0 | 0 |
| CTC-325L16.1 | 0 | 0 | 0 | 0 | 1 | 0 |
| CTC-444N24.8 | 0 | 0 | 0 | 0 | 1 | 0 |
| CTC-454I21.4 | 0 | 0 | 0 | 0 | 1 | 0 |
| CTD-2054N24.2 | 0 | 0 | 0 | 0 | 1 | 0 |
| CTD-2083E4.4 | 0 | 0 | 0 | 0 | 1 | 0 |
| CTD-2192J16.20 | 0 | 0 | 0 | 0 | 1 | 0 |
| CTD-2192J16.24 | 0 | 0 | 0 | 0 | 1 | 0 |
| CTD-2201E18.3 | 0 | 0 | 0 | 0 | 1 | 0 |
| CTD-2210P24.6 | 0 | 0 | 0 | 0 | 1 | 0 |
| CTD-2270L9.5 | 0 | 0 | 0 | 0 | 1 | 0 |
| CTD-2278I10.6 | 0 | 0 | 0 | 0 | 1 | 0 |
| CTD-2323K18.1 | 0 | 0 | 0 | 0 | 1 | 0 |
| CTD-2323K18.2 | 0 | 0 | 0 | 0 | 1 | 0 |
| CTD-2510F5.6 | 1 | 0 | 0 | 0 | 0 | 0 |
| CTD-2540M10.1 | 0 | 0 | 0 | 0 | 1 | 0 |
| CTD-2545G14.7 | 0 | 0 | 0 | 0 | 1 | 0 |
| CTD-2574D22.6 | 0 | 0 | 0 | 0 | 1 | 0 |
| CTD-2593A12.4 | 0 | 0 | 0 | 0 | 1 | 0 |
| CTD-3064H18.6 | 0 | 0 | 0 | 0 | 1 | 0 |
| CTD-3083F21.5 | 0 | 0 | 0 | 0 | 1 | 0 |
| CTD-3214H19.16 | 0 | 0 | 0 | 0 | 1 | 0 |
| CTD-3222D19.2 | 0 | 0 | 0 | 0 | 1 | 0 |
| CTDSP1 | 0 | 0 | 0 | 0 | 1 | 0 |
| CTDSP2 | 1 | 0 | 0 | 0 | 0 | 0 |
| CTDSPL | 0 | 0 | 0 | 0 | 1 | 0 |
| CTDSPL2 | 1 | 0 | 1 | 0 | 0 | 0 |
| CTGF | 1 | 0 | 0 | 0 | 0 | 0 |
| CTH | 0 | 0 | 0 | 0 | 1 | 0 |
| CTNNA1 | 1 | 0 | 0 | 0 | 0 | 1 |
| CTNNA3 | 1 | 0 | 0 | 0 | 0 | 0 |
| CTNNB1 | 1 | 0 | 1 | 0 | 0 | 0 |
| CTPS2 | 0 | 0 | 1 | 0 | 0 | 0 |
| CTSB | 1 | 0 | 0 | 0 | 0 | 0 |
| CTSC | 1 | 0 | 0 | 0 | 0 | 0 |
| CTSD | 0 | 0 | 0 | 0 | 1 | 0 |
| CTSS | 0 | 0 | 0 | 0 | 1 | 0 |
| CTXN1 | 0 | 0 | 0 | 0 | 1 | 0 |
| CELF1 | 0 | 0 | 0 | 1 | 0 | 0 |
| CELF2 | 0 | 1 | 0 | 1 | 0 | 0 |
| CUL1 | 0 | 0 | 1 | 0 | 0 | 0 |
| CUL4A | 0 | 0 | 0 | 1 | 0 | 0 |
| CUL4B | 1 | 0 | 0 | 1 | 0 | 0 |
| CUTC | 0 | 0 | 0 | 0 | 1 | 0 |
| CUX1 | 0 | 0 | 1 | 0 | 0 | 0 |
| CUZD1 | 0 | 0 | 0 | 0 | 1 | 0 |
| CXCL10 | 0 | 0 | 0 | 1 | 0 | 0 |
| CXCL11 | 0 | 0 | 0 | 0 | 1 | 0 |
| CXCL2 | 0 | 0 | 0 | 0 | 1 | 0 |
| CXCR4 | 0 | 0 | 1 | 1 | 0 | 0 |
| CXCR7 | 0 | 0 | 0 | 0 | 0 | 1 |
| CXorf1 | 0 | 0 | 0 | 0 | 1 | 0 |
| CXorf24 | 0 | 0 | 0 | 0 | 1 | 0 |
| CXorf48 | 0 | 0 | 0 | 0 | 1 | 0 |
| CXorf56 | 0 | 1 | 0 | 0 | 1 | 0 |
| CXorf57 | 1 | 0 | 0 | 0 | 0 | 0 |
| CYB561 | 0 | 0 | 0 | 0 | 1 | 0 |
| CYB5B | 0 | 0 | 1 | 1 | 0 | 0 |
| CYB5D2 | 1 | 0 | 0 | 0 | 0 | 0 |
| CYB5R3 | 0 | 0 | 0 | 0 | 1 | 0 |
| CYBA | 0 | 0 | 0 | 0 | 1 | 0 |
| CYBASC3 | 0 | 0 | 0 | 0 | 1 | 0 |
| CYCS | 1 | 0 | 0 | 1 | 0 | 0 |
| CYFIP1 | 0 | 0 | 1 | 0 | 0 | 0 |
| CYHR1 | 0 | 0 | 1 | 0 | 0 | 0 |
| CYP1B1 | 0 | 0 | 1 | 0 | 0 | 0 |
| CYP20A1 | 0 | 0 | 0 | 1 | 0 | 0 |
| CYP21A1P | 0 | 0 | 0 | 0 | 1 | 0 |
| CYP2D6 | 0 | 1 | 0 | 0 | 0 | 0 |
| CYP51A1 | 0 | 0 | 1 | 0 | 0 | 0 |
| CYSTM1 | 0 | 0 | 0 | 0 | 1 | 0 |
| CYTH3 | 0 | 0 | 1 | 0 | 0 | 0 |
| D2HGDH | 0 | 0 | 1 | 0 | 0 | 0 |
| DAAM1 | 0 | 0 | 0 | 0 | 1 | 0 |
| DAD1 | 1 | 0 | 0 | 0 | 0 | 0 |
| DAG1 | 0 | 0 | 1 | 0 | 0 | 0 |
| DAGLB | 1 | 0 | 1 | 0 | 0 | 0 |
| DAP | 0 | 0 | 1 | 0 | 0 | 0 |
| DAP3 | 1 | 0 | 0 | 0 | 0 | 0 |
| DAPP1 | 0 | 0 | 0 | 1 | 0 | 0 |
| DARS | 0 | 0 | 0 | 1 | 0 | 0 |
| DAZAP1 | 0 | 0 | 0 | 1 | 0 | 0 |
| DAZAP2 | 1 | 0 | 0 | 1 | 0 | 0 |
| DAZAP2P1 | 0 | 0 | 0 | 0 | 1 | 0 |
| DBC1 | 0 | 1 | 0 | 0 | 0 | 0 |
| DBI | 1 | 0 | 0 | 0 | 0 | 0 |
| DBT | 0 | 0 | 0 | 0 | 1 | 0 |
| DBX2 | 0 | 0 | 0 | 0 | 1 | 0 |
| DCAF12 | 1 | 0 | 0 | 0 | 0 | 0 |
| DCAF13P3 | 0 | 0 | 0 | 0 | 1 | 0 |
| DCAF16 | 1 | 0 | 0 | 0 | 0 | 0 |
| DCAF17 | 1 | 0 | 0 | 0 | 0 | 0 |
| DCAF7 | 1 | 0 | 1 | 0 | 0 | 0 |
| DCAF8 | 0 | 0 | 1 | 0 | 1 | 0 |
| DCBLD2 | 1 | 0 | 0 | 0 | 1 | 0 |
| DCK | 1 | 0 | 0 | 1 | 1 | 0 |
| DCLK2 | 0 | 0 | 1 | 0 | 0 | 0 |
| DCLRE1B | 0 | 0 | 0 | 0 | 1 | 0 |
| DCLRE1C | 0 | 0 | 0 | 1 | 0 | 0 |
| DCP2 | 1 | 0 | 0 | 1 | 0 | 0 |
| DCT | 0 | 1 | 0 | 0 | 0 | 0 |
| DCTD | 0 | 0 | 0 | 1 | 1 | 0 |
| DCTN3 | 0 | 0 | 0 | 1 | 0 | 0 |
| DCTN4 | 1 | 0 | 0 | 1 | 0 | 0 |
| DCTN6 | 0 | 0 | 0 | 0 | 1 | 0 |
| DCUN1D4 | 1 | 0 | 1 | 0 | 1 | 0 |
| DDB1 | 1 | 0 | 0 | 0 | 0 | 0 |
| DDB2 | 0 | 0 | 1 | 0 | 0 | 0 |
| DDIT4 | 0 | 0 | 1 | 1 | 0 | 0 |
| DDOST | 1 | 0 | 0 | 0 | 0 | 0 |
| DDX1 | 1 | 0 | 0 | 0 | 0 | 0 |
| DDX17 | 1 | 0 | 1 | 1 | 0 | 0 |
| DDX21 | 1 | 0 | 1 | 1 | 0 | 0 |
| DDX24 | 1 | 0 | 1 | 0 | 0 | 0 |
| DDX27 | 0 | 0 | 0 | 1 | 0 | 0 |
| DDX39 | 0 | 0 | 0 | 1 | 0 | 0 |
| DDX3X | 1 | 0 | 1 | 1 | 0 | 0 |
| DDX3Y | 1 | 1 | 0 | 0 | 0 | 0 |
| DDX46 | 0 | 0 | 0 | 0 | 1 | 0 |
| DDX5 | 1 | 0 | 0 | 1 | 0 | 0 |
| DDX50P1 | 0 | 0 | 0 | 0 | 1 | 0 |
| DDX54 | 0 | 1 | 0 | 0 | 0 | 0 |
| DDX56 | 1 | 0 | 0 | 0 | 0 | 0 |
| DDX58 | 0 | 0 | 0 | 1 | 0 | 0 |
| DDX6 | 0 | 0 | 0 | 1 | 0 | 0 |
| DDX60 | 0 | 0 | 0 | 1 | 0 | 0 |
| DECR1 | 0 | 0 | 0 | 1 | 0 | 0 |
| DEFB110 | 0 | 0 | 0 | 0 | 0 | 1 |
| DEK | 1 | 0 | 0 | 1 | 0 | 0 |
| DENND1A | 0 | 0 | 1 | 0 | 0 | 0 |
| DENND6A | 1 | 0 | 0 | 0 | 0 | 0 |
| DENR | 1 | 0 | 0 | 1 | 1 | 0 |
| DEPDC1 | 1 | 0 | 1 | 0 | 0 | 0 |
| DEPDC5 | 0 | 0 | 0 | 0 | 1 | 0 |
| DERL1 | 0 | 0 | 0 | 1 | 0 | 0 |
| DERL2 | 0 | 0 | 1 | 0 | 1 | 0 |
| DESI2 | 1 | 0 | 0 | 0 | 1 | 0 |
| DEXI | 0 | 0 | 0 | 0 | 1 | 0 |
| DFFB | 0 | 0 | 0 | 0 | 1 | 0 |
| DGCR2 | 1 | 0 | 1 | 0 | 1 | 0 |
| DGCR8 | 0 | 0 | 1 | 0 | 0 | 0 |
| DGKG | 0 | 0 | 0 | 0 | 1 | 0 |
| DGKH | 0 | 0 | 1 | 0 | 0 | 0 |
| DGKQ | 0 | 0 | 0 | 0 | 1 | 0 |
| DGUOK | 1 | 0 | 0 | 0 | 1 | 0 |
| DHFR | 1 | 0 | 1 | 0 | 0 | 0 |
| DHRS1 | 0 | 0 | 0 | 0 | 1 | 0 |
| DHRS11 | 0 | 0 | 0 | 0 | 1 | 0 |
| DHX15 | 1 | 0 | 0 | 0 | 0 | 0 |
| DHX30 | 0 | 0 | 1 | 0 | 0 | 0 |
| DHX34 | 0 | 0 | 1 | 0 | 0 | 0 |
| DHX36 | 1 | 0 | 1 | 0 | 0 | 0 |
| DHX40 | 1 | 0 | 1 | 1 | 0 | 0 |
| DHX9 | 1 | 0 | 0 | 0 | 0 | 0 |
| DIABLO | 1 | 0 | 0 | 0 | 1 | 0 |
| DIAPH3 | 0 | 0 | 1 | 0 | 0 | 0 |
| DICER1 | 1 | 0 | 0 | 0 | 0 | 0 |
| DIDO1 | 1 | 0 | 0 | 0 | 0 | 0 |
| DIMT1L | 0 | 0 | 0 | 1 | 0 | 0 |
| DIP2B | 1 | 0 | 0 | 0 | 1 | 0 |
| DIRAS1 | 0 | 0 | 0 | 0 | 1 | 0 |
| DIRAS3 | 0 | 0 | 0 | 0 | 1 | 0 |
| DIRC2 | 0 | 0 | 1 | 0 | 0 | 0 |
| DKC1 | 1 | 0 | 0 | 0 | 0 | 0 |
| DKFZp564N2472 | 0 | 1 | 0 | 0 | 0 | 0 |
| DKFZp667E0512 | 0 | 1 | 0 | 0 | 0 | 0 |
| DLEU2 | 0 | 0 | 1 | 0 | 0 | 0 |
| DLG1 | 1 | 0 | 1 | 0 | 0 | 0 |
| DLG2 | 1 | 0 | 0 | 0 | 0 | 0 |
| DLGAP1-AS2 | 0 | 0 | 1 | 0 | 0 | 0 |
| DLGAP4 | 0 | 0 | 1 | 0 | 0 | 0 |
| DLX1 | 1 | 0 | 0 | 0 | 1 | 0 |
| DMC1 | 0 | 1 | 0 | 0 | 1 | 0 |
| DMD | 1 | 0 | 1 | 0 | 0 | 0 |
| DMTF1 | 0 | 0 | 1 | 0 | 0 | 0 |
| DMXL1 | 1 | 0 | 0 | 0 | 0 | 0 |
| DNAAF2 | 0 | 0 | 0 | 0 | 1 | 0 |
| DNAI1 | 0 | 1 | 0 | 0 | 0 | 0 |
| DNAJA1 | 1 | 0 | 1 | 1 | 0 | 0 |
| DNAJA2 | 1 | 0 | 0 | 0 | 1 | 0 |
| DNAJB1 | 1 | 0 | 0 | 1 | 0 | 0 |
| DNAJB11 | 1 | 0 | 0 | 0 | 0 | 0 |
| DNAJB9 | 0 | 0 | 1 | 1 | 0 | 0 |
| DNAJC10 | 0 | 0 | 0 | 1 | 0 | 0 |
| DNAJC19 | 0 | 0 | 0 | 0 | 1 | 0 |
| DNAJC3 | 0 | 0 | 1 | 0 | 1 | 0 |
| DNAJC5 | 1 | 0 | 1 | 0 | 1 | 0 |
| DNAJC8 | 0 | 0 | 0 | 1 | 0 | 0 |
| DNAL4 | 0 | 0 | 0 | 0 | 1 | 0 |
| DNER | 1 | 0 | 0 | 0 | 0 | 0 |
| DNM3OS | 0 | 0 | 0 | 0 | 1 | 0 |
| DNMT1 | 1 | 0 | 1 | 0 | 0 | 0 |
| DNMT3B | 0 | 0 | 1 | 0 | 0 | 0 |
| DOCK10 | 0 | 0 | 0 | 1 | 0 | 0 |
| DOCK9 | 0 | 1 | 1 | 0 | 0 | 0 |
| DOLPP1 | 1 | 0 | 0 | 0 | 1 | 0 |
| DOT1L | 0 | 0 | 1 | 0 | 0 | 0 |
| DPH2 | 0 | 0 | 0 | 0 | 1 | 0 |
| DPH3 | 0 | 0 | 0 | 1 | 1 | 0 |
| DPH3P1 | 0 | 0 | 0 | 0 | 1 | 0 |
| DPP6 | 1 | 0 | 0 | 0 | 0 | 0 |
| DPP8 | 0 | 0 | 1 | 0 | 0 | 0 |
| DPY19L1 | 1 | 0 | 1 | 0 | 1 | 0 |
| DPY19L3 | 1 | 0 | 0 | 1 | 1 | 0 |
| DPY19L4 | 1 | 0 | 0 | 0 | 0 | 0 |
| DPYSL2 | 1 | 0 | 0 | 0 | 0 | 1 |
| DR1 | 1 | 0 | 0 | 1 | 1 | 0 |
| DRAM1 | 0 | 0 | 0 | 0 | 1 | 0 |
| DRAM2 | 0 | 0 | 0 | 0 | 1 | 0 |
| DRG1 | 0 | 0 | 1 | 0 | 1 | 0 |
| DROSHA | 0 | 0 | 1 | 0 | 0 | 0 |
| DSCR10 | 0 | 0 | 0 | 0 | 1 | 1 |
| DSEL | 0 | 0 | 1 | 0 | 0 | 0 |
| DSN1 | 0 | 0 | 0 | 1 | 0 | 0 |
| DSTN | 1 | 0 | 0 | 0 | 0 | 0 |
| DTD1 | 0 | 0 | 0 | 0 | 1 | 0 |
| DTD2 | 0 | 0 | 0 | 0 | 1 | 0 |
| DTWD1 | 0 | 0 | 1 | 0 | 0 | 0 |
| DTWD2 | 1 | 0 | 0 | 0 | 0 | 0 |
| DTX3L | 1 | 0 | 0 | 1 | 0 | 0 |
| DTYMK | 1 | 0 | 0 | 0 | 0 | 0 |
| DUOX1 | 0 | 1 | 0 | 0 | 0 | 0 |
| DUS2L | 0 | 1 | 0 | 0 | 0 | 0 |
| DUSP11 | 1 | 0 | 0 | 0 | 0 | 0 |
| DUSP16 | 0 | 0 | 0 | 0 | 1 | 0 |
| DUSP19 | 0 | 0 | 0 | 0 | 1 | 0 |
| DUSP22 | 0 | 0 | 0 | 0 | 1 | 0 |
| DUSP3 | 0 | 0 | 0 | 0 | 1 | 0 |
| DUSP5 | 1 | 0 | 0 | 1 | 0 | 0 |
| DUSP6 | 1 | 0 | 0 | 0 | 0 | 0 |
| DUSP7 | 1 | 0 | 0 | 0 | 0 | 0 |
| DUT | 1 | 0 | 1 | 0 | 1 | 0 |
| DUTP3 | 0 | 0 | 0 | 0 | 1 | 0 |
| DYNC1H1 | 1 | 0 | 0 | 0 | 0 | 0 |
| DYNC1I2 | 1 | 0 | 0 | 0 | 0 | 0 |
| DYNC1LI2 | 1 | 0 | 1 | 0 | 0 | 0 |
| DYNLL1 | 1 | 0 | 0 | 0 | 0 | 0 |
| DYNLL1P1 | 0 | 0 | 0 | 0 | 1 | 0 |
| DYNLL2 | 1 | 0 | 1 | 0 | 1 | 0 |
| DYNLRB1 | 0 | 0 | 0 | 1 | 0 | 0 |
| DYNLT3 | 0 | 0 | 0 | 0 | 1 | 0 |
| DYRK2 | 1 | 0 | 0 | 1 | 0 | 0 |
| E2F3 | 1 | 0 | 1 | 0 | 1 | 0 |
| E2F3-IT1 | 0 | 0 | 0 | 0 | 1 | 0 |
| EAF1 | 0 | 0 | 0 | 1 | 1 | 0 |
| EARS2 | 0 | 0 | 1 | 0 | 0 | 0 |
| EBPL | 0 | 0 | 0 | 1 | 0 | 0 |
| ECE2 | 0 | 0 | 0 | 0 | 1 | 0 |
| ECH1 | 1 | 0 | 0 | 0 | 0 | 0 |
| ECHDC1 | 0 | 0 | 0 | 1 | 0 | 0 |
| ECHS1 | 0 | 0 | 0 | 0 | 1 | 0 |
| ECT2 | 0 | 0 | 1 | 0 | 0 | 0 |
| EDARADD | 1 | 0 | 0 | 0 | 0 | 0 |
| EDC3 | 0 | 0 | 0 | 0 | 1 | 0 |
| EED | 0 | 0 | 0 | 0 | 1 | 1 |
| EEF1A1 | 1 | 0 | 0 | 1 | 0 | 0 |
| EEF1B2 | 1 | 0 | 0 | 0 | 0 | 0 |
| EEF1E1 | 1 | 0 | 1 | 0 | 0 | 0 |
| EEF1E1-BLOC1S5 | 0 | 0 | 1 | 0 | 0 | 0 |
| EEF1G | 1 | 0 | 0 | 0 | 0 | 0 |
| EEF2 | 1 | 0 | 0 | 0 | 0 | 0 |
| EEF2K | 1 | 0 | 0 | 0 | 0 | 0 |
| EEFSEC | 0 | 0 | 1 | 0 | 0 | 0 |
| EFCAB11 | 1 | 0 | 1 | 0 | 0 | 0 |
| EFCAB14 | 1 | 0 | 1 | 0 | 0 | 0 |
| EFCAB4B | 0 | 0 | 0 | 0 | 1 | 0 |
| EFHA1 | 0 | 0 | 0 | 1 | 0 | 0 |
| EFHC2 | 0 | 0 | 0 | 0 | 1 | 0 |
| EFHD1 | 0 | 0 | 0 | 0 | 1 | 0 |
| EFHD2 | 1 | 0 | 1 | 0 | 0 | 0 |
| EFNA1 | 0 | 0 | 1 | 0 | 0 | 0 |
| EFNA5 | 1 | 0 | 0 | 0 | 0 | 0 |
| EFR3A | 1 | 0 | 0 | 1 | 0 | 0 |
| EFTUD2 | 0 | 0 | 1 | 0 | 0 | 0 |
| EGFEM1P | 0 | 0 | 0 | 0 | 0 | 1 |
| EGFR | 0 | 0 | 1 | 0 | 0 | 0 |
| EGLN1P1 | 0 | 0 | 0 | 0 | 1 | 0 |
| EGLN3 | 0 | 0 | 1 | 0 | 1 | 0 |
| EGR3 | 0 | 0 | 0 | 0 | 0 | 1 |
| EHMT1 | 0 | 0 | 1 | 0 | 0 | 0 |
| EI24 | 1 | 0 | 0 | 1 | 0 | 0 |
| EID1 | 1 | 0 | 0 | 0 | 0 | 0 |
| EIF1 | 1 | 0 | 0 | 1 | 0 | 0 |
| EIF1AD | 1 | 0 | 1 | 0 | 1 | 0 |
| EIF1AX | 1 | 0 | 0 | 0 | 1 | 1 |
| EIF1AXP1 | 0 | 0 | 0 | 0 | 1 | 0 |
| EIF1B | 1 | 0 | 0 | 1 | 1 | 0 |
| EIF2AK1 | 0 | 0 | 1 | 0 | 0 | 0 |
| EIF2AK2 | 1 | 0 | 0 | 1 | 0 | 0 |
| EIF2B2 | 0 | 0 | 0 | 0 | 1 | 0 |
| EIF2B5 | 1 | 0 | 0 | 0 | 1 | 0 |
| EIF2C2 | 0 | 0 | 0 | 1 | 0 | 0 |
| EIF2S1 | 1 | 0 | 0 | 0 | 1 | 0 |
| EIF2S2 | 1 | 0 | 0 | 0 | 0 | 0 |
| EIF2S3 | 1 | 0 | 1 | 0 | 0 | 0 |
| EIF3A | 1 | 0 | 1 | 0 | 0 | 0 |
| EIF3B | 1 | 0 | 0 | 0 | 0 | 0 |
| EIF3C | 0 | 0 | 0 | 1 | 0 | 0 |
| EIF3D | 1 | 0 | 1 | 0 | 0 | 0 |
| EIF3E | 1 | 0 | 0 | 0 | 0 | 0 |
| EIF3F | 1 | 0 | 0 | 0 | 0 | 0 |
| EIF3G | 1 | 0 | 1 | 0 | 0 | 0 |
| EIF3I | 1 | 0 | 0 | 0 | 0 | 0 |
| EIF3K | 1 | 0 | 0 | 0 | 0 | 0 |
| EIF3L | 1 | 0 | 0 | 0 | 0 | 0 |
| EIF3M | 1 | 0 | 0 | 0 | 0 | 0 |
| EIF4A1 | 1 | 0 | 0 | 0 | 0 | 0 |
| EIF4A2 | 1 | 0 | 1 | 1 | 1 | 0 |
| EIF4A3 | 1 | 0 | 0 | 0 | 0 | 0 |
| EIF4B | 1 | 0 | 0 | 0 | 0 | 0 |
| EIF4E | 1 | 0 | 0 | 1 | 0 | 0 |
| EIF4E2 | 1 | 0 | 0 | 0 | 1 | 0 |
| EIF4EBP2 | 1 | 0 | 0 | 1 | 0 | 0 |
| EIF4G1 | 1 | 0 | 0 | 0 | 0 | 0 |
| EIF4G2 | 1 | 0 | 0 | 0 | 0 | 0 |
| EIF4H | 1 | 0 | 0 | 0 | 0 | 0 |
| EIF5 | 1 | 0 | 0 | 1 | 0 | 0 |
| EIF5A | 1 | 0 | 1 | 0 | 0 | 0 |
| EIF5AL1 | 1 | 0 | 0 | 0 | 0 | 0 |
| EIF6 | 1 | 0 | 0 | 0 | 0 | 0 |
| ELAVL1 | 1 | 0 | 0 | 1 | 1 | 0 |
| ELF4 | 0 | 0 | 0 | 1 | 0 | 0 |
| ELK3 | 1 | 0 | 0 | 0 | 0 | 0 |
| ELK4 | 1 | 0 | 0 | 0 | 0 | 0 |
| ELL2 | 1 | 0 | 0 | 1 | 0 | 0 |
| ELMOD2 | 0 | 0 | 0 | 0 | 1 | 0 |
| ELOF1 | 1 | 0 | 0 | 0 | 0 | 1 |
| ELOVL1 | 0 | 0 | 0 | 0 | 1 | 0 |
| ELOVL4 | 0 | 0 | 0 | 0 | 1 | 0 |
| ELOVL5 | 1 | 0 | 0 | 0 | 0 | 0 |
| ELOVL6 | 0 | 0 | 1 | 0 | 0 | 0 |
| ELP2 | 0 | 0 | 1 | 0 | 0 | 0 |
| ELP5 | 0 | 0 | 0 | 0 | 1 | 0 |
| ELP6 | 0 | 0 | 0 | 0 | 1 | 0 |
| EMC1 | 1 | 0 | 0 | 0 | 0 | 0 |
| EMC10 | 0 | 0 | 1 | 0 | 1 | 0 |
| EMC9 | 0 | 0 | 0 | 0 | 1 | 0 |
| EMG1 | 0 | 0 | 0 | 0 | 1 | 0 |
| EML2 | 0 | 0 | 1 | 0 | 0 | 0 |
| EMP2 | 0 | 0 | 0 | 0 | 1 | 0 |
| ENAH | 1 | 0 | 0 | 0 | 0 | 0 |
| ENC1 | 1 | 0 | 0 | 0 | 0 | 0 |
| ENDOV | 0 | 0 | 1 | 0 | 0 | 0 |
| ENO1 | 1 | 0 | 0 | 0 | 0 | 0 |
| ENO1P4 | 0 | 0 | 0 | 0 | 1 | 0 |
| ENO2 | 1 | 0 | 0 | 0 | 0 | 0 |
| ENOPH1 | 0 | 0 | 0 | 1 | 0 | 0 |
| ENOSF1 | 1 | 0 | 1 | 0 | 0 | 0 |
| ENOX1 | 0 | 0 | 1 | 0 | 0 | 0 |
| ENOX2 | 0 | 0 | 0 | 0 | 1 | 0 |
| ENPP1 | 0 | 1 | 0 | 0 | 0 | 0 |
| ENPP6 | 0 | 1 | 0 | 0 | 0 | 0 |
| ENSA | 1 | 0 | 0 | 1 | 0 | 0 |
| ENTPD3 | 0 | 0 | 0 | 0 | 1 | 0 |
| ENTPD6 | 0 | 0 | 0 | 0 | 1 | 0 |
| ENTPD7 | 0 | 0 | 0 | 0 | 1 | 0 |
| EOGT | 1 | 0 | 0 | 0 | 1 | 0 |
| EOMES | 0 | 0 | 0 | 1 | 0 | 0 |
| EP400NL | 0 | 1 | 0 | 0 | 0 | 0 |
| EPB41L3 | 1 | 0 | 0 | 0 | 0 | 0 |
| EPC1 | 0 | 0 | 0 | 1 | 0 | 0 |
| EPC2 | 0 | 0 | 0 | 1 | 0 | 0 |
| EPCAM | 0 | 0 | 0 | 0 | 1 | 0 |
| EPDR1 | 1 | 0 | 0 | 0 | 0 | 0 |
| EPHA4 | 0 | 1 | 0 | 0 | 0 | 0 |
| EPHB4 | 0 | 0 | 1 | 0 | 0 | 0 |
| EPHX3 | 0 | 0 | 0 | 0 | 1 | 0 |
| EPPIN | 1 | 0 | 0 | 0 | 0 | 0 |
| EPPIN-WFDC6 | 1 | 0 | 0 | 0 | 0 | 0 |
| EPS15 | 0 | 0 | 0 | 1 | 0 | 0 |
| EPT1 | 1 | 0 | 0 | 0 | 1 | 0 |
| ERAP1 | 0 | 0 | 0 | 1 | 0 | 0 |
| ERAP2 | 0 | 0 | 0 | 1 | 0 | 0 |
| ERCC1 | 1 | 0 | 0 | 0 | 0 | 0 |
| ERCC5 | 0 | 0 | 1 | 0 | 0 | 0 |
| ERCC6L2 | 0 | 0 | 1 | 0 | 0 | 0 |
| ERGIC3 | 1 | 0 | 0 | 0 | 0 | 0 |
| ERH | 1 | 0 | 1 | 1 | 0 | 0 |
| ERLEC1 | 1 | 0 | 0 | 0 | 0 | 0 |
| ERMP1 | 0 | 0 | 1 | 0 | 1 | 0 |
| ERN1 | 0 | 1 | 0 | 0 | 0 | 0 |
| ERO1L | 1 | 0 | 1 | 0 | 0 | 0 |
| ERO1LB | 1 | 0 | 0 | 0 | 0 | 0 |
| ERP29 | 0 | 0 | 0 | 1 | 0 | 0 |
| ESYT2 | 0 | 0 | 1 | 0 | 0 | 0 |
| ETF1 | 1 | 0 | 0 | 1 | 0 | 0 |
| ETFA | 1 | 0 | 0 | 0 | 0 | 0 |
| ETFB | 0 | 0 | 1 | 0 | 0 | 0 |
| ETFDH | 0 | 0 | 0 | 0 | 1 | 0 |
| ETNK1 | 1 | 0 | 0 | 1 | 0 | 0 |
| ETS1 | 1 | 0 | 0 | 0 | 0 | 0 |
| ETV4 | 0 | 0 | 0 | 0 | 1 | 0 |
| ETV5 | 1 | 0 | 0 | 0 | 1 | 0 |
| EVI2B | 0 | 0 | 0 | 1 | 0 | 0 |
| EVI5 | 1 | 0 | 1 | 0 | 0 | 0 |
| EWSR1 | 0 | 0 | 1 | 0 | 0 | 0 |
| EXO1 | 0 | 0 | 1 | 0 | 0 | 0 |
| EXOC4 | 0 | 0 | 1 | 0 | 0 | 0 |
| EXOC5 | 0 | 0 | 0 | 1 | 0 | 0 |
| EXOC8 | 0 | 0 | 0 | 0 | 1 | 0 |
| EXOSC7 | 0 | 0 | 1 | 0 | 0 | 0 |
| EXT2 | 0 | 0 | 0 | 0 | 1 | 0 |
| EXTL2 | 0 | 0 | 1 | 0 | 0 | 0 |
| EXTL3 | 0 | 0 | 0 | 0 | 1 | 0 |
| EYA4 | 1 | 0 | 0 | 0 | 0 | 0 |
| EYS | 1 | 0 | 0 | 0 | 0 | 0 |
| EZR | 1 | 0 | 0 | 1 | 0 | 0 |
| F11R | 0 | 0 | 0 | 0 | 0 | 1 |
| F2R | 0 | 0 | 0 | 0 | 1 | 0 |
| F7 | 0 | 1 | 0 | 0 | 0 | 0 |
| FABP5 | 1 | 0 | 0 | 1 | 0 | 0 |
| FADS2 | 0 | 0 | 0 | 0 | 1 | 0 |
| FAF1 | 0 | 0 | 1 | 0 | 0 | 0 |
| FAM100A | 0 | 0 | 0 | 0 | 1 | 0 |
| FAM102B | 0 | 0 | 0 | 1 | 0 | 0 |
| FAM104A | 0 | 0 | 1 | 0 | 0 | 0 |
| FAM104B | 0 | 0 | 0 | 0 | 1 | 0 |
| FAM105B | 1 | 0 | 0 | 0 | 0 | 0 |
| FAM107B | 0 | 0 | 0 | 1 | 0 | 0 |
| FAM108B1 | 0 | 0 | 0 | 0 | 1 | 0 |
| FAM114A2 | 0 | 0 | 0 | 1 | 0 | 0 |
| FAM116A | 0 | 0 | 0 | 1 | 0 | 0 |
| FAM117B | 0 | 0 | 0 | 1 | 0 | 0 |
| FAM120A | 1 | 0 | 0 | 1 | 0 | 0 |
| FAM122B | 1 | 0 | 1 | 0 | 1 | 0 |
| FAM126A | 1 | 0 | 0 | 1 | 1 | 0 |
| FAM127A | 0 | 0 | 0 | 0 | 1 | 0 |
| FAM129B | 1 | 0 | 0 | 0 | 0 | 0 |
| FAM12A | 0 | 1 | 0 | 0 | 0 | 0 |
| FAM133B | 0 | 0 | 1 | 0 | 0 | 0 |
| FAM134A | 1 | 0 | 0 | 0 | 1 | 0 |
| FAM134B | 0 | 0 | 0 | 1 | 0 | 0 |
| FAM134C | 0 | 0 | 0 | 1 | 1 | 0 |
| FAM135B | 0 | 1 | 0 | 0 | 0 | 1 |
| FAM149B1 | 0 | 0 | 0 | 0 | 1 | 0 |
| FAM160A1 | 0 | 0 | 1 | 0 | 0 | 0 |
| FAM160A2 | 0 | 0 | 1 | 0 | 0 | 0 |
| FAM160B1 | 1 | 0 | 0 | 0 | 0 | 0 |
| FAM162A | 0 | 0 | 0 | 1 | 0 | 0 |
| FAM164A | 0 | 0 | 0 | 1 | 0 | 0 |
| FAM165A | 0 | 0 | 0 | 0 | 0 | 1 |
| FAM165B | 0 | 0 | 0 | 0 | 1 | 0 |
| FAM166A | 1 | 0 | 0 | 0 | 0 | 0 |
| FAM171A1 | 0 | 0 | 0 | 0 | 1 | 0 |
| FAM172A | 0 | 0 | 1 | 0 | 0 | 0 |
| FAM173B | 0 | 0 | 0 | 0 | 1 | 0 |
| FAM174A | 0 | 0 | 0 | 0 | 1 | 0 |
| FAM178A | 1 | 0 | 0 | 0 | 1 | 0 |
| FAM189A1 | 1 | 0 | 0 | 0 | 1 | 0 |
| FAM189B | 0 | 0 | 0 | 0 | 1 | 0 |
| FAM18B | 0 | 0 | 0 | 1 | 0 | 0 |
| FAM18B1 | 0 | 0 | 0 | 0 | 1 | 0 |
| FAM195A | 0 | 0 | 0 | 0 | 1 | 0 |
| FAM199X | 1 | 0 | 0 | 0 | 0 | 0 |
| FAM208A | 0 | 0 | 1 | 0 | 0 | 0 |
| FAM20B | 1 | 0 | 0 | 1 | 0 | 0 |
| FAM212B | 0 | 0 | 0 | 0 | 1 | 0 |
| FAM214A | 0 | 0 | 0 | 0 | 1 | 0 |
| FAM214B | 0 | 0 | 0 | 0 | 1 | 0 |
| FAM219A | 1 | 0 | 0 | 0 | 1 | 0 |
| FAM24B | 0 | 0 | 0 | 0 | 1 | 0 |
| FAM26F | 0 | 0 | 0 | 1 | 0 | 0 |
| FAM27A | 0 | 0 | 0 | 0 | 1 | 0 |
| FAM27B | 0 | 0 | 0 | 0 | 1 | 0 |
| FAM27C | 0 | 0 | 0 | 0 | 1 | 0 |
| FAM32A | 0 | 0 | 0 | 1 | 1 | 0 |
| FAM3C | 1 | 0 | 0 | 0 | 0 | 0 |
| FAM40A | 0 | 1 | 0 | 0 | 0 | 0 |
| FAM46A | 0 | 0 | 0 | 0 | 1 | 0 |
| FAM46C | 0 | 0 | 0 | 1 | 1 | 0 |
| FAM49B | 1 | 0 | 0 | 1 | 1 | 0 |
| FAM53C | 0 | 0 | 0 | 0 | 1 | 0 |
| FAM54A | 0 | 0 | 0 | 0 | 1 | 0 |
| FAM54B | 0 | 0 | 0 | 0 | 1 | 0 |
| FAM57A | 0 | 0 | 0 | 0 | 1 | 0 |
| FAM60A | 1 | 0 | 0 | 0 | 0 | 0 |
| FAM62B | 0 | 0 | 0 | 1 | 0 | 0 |
| FAM73A | 0 | 0 | 0 | 1 | 1 | 0 |
| FAM83D | 1 | 0 | 1 | 0 | 0 | 0 |
| FAM83H | 0 | 1 | 0 | 0 | 0 | 0 |
| FAM91A1 | 1 | 0 | 0 | 1 | 0 | 0 |
| FAM91A2 | 0 | 0 | 0 | 1 | 0 | 0 |
| FAM96A | 1 | 0 | 0 | 0 | 0 | 0 |
| FANCA | 0 | 0 | 1 | 0 | 0 | 0 |
| FANCC | 0 | 0 | 0 | 0 | 0 | 1 |
| FANCF | 0 | 0 | 0 | 0 | 1 | 0 |
| FAR1 | 1 | 0 | 0 | 1 | 0 | 0 |
| FAR2 | 0 | 0 | 0 | 0 | 1 | 0 |
| FARP1 | 0 | 0 | 1 | 0 | 0 | 0 |
| FARSA | 0 | 0 | 1 | 0 | 0 | 0 |
| FASN | 1 | 0 | 1 | 0 | 0 | 0 |
| FASTKD1 | 0 | 0 | 0 | 0 | 1 | 0 |
| FASTKD2 | 1 | 0 | 0 | 0 | 0 | 0 |
| FASTKD5 | 0 | 0 | 0 | 0 | 1 | 0 |
| FAT1 | 1 | 0 | 0 | 0 | 1 | 0 |
| FAT2 | 0 | 0 | 0 | 0 | 0 | 1 |
| FAU | 1 | 0 | 0 | 0 | 0 | 0 |
| FBL | 0 | 0 | 0 | 0 | 1 | 0 |
| FBLL1 | 0 | 0 | 0 | 0 | 1 | 0 |
| FBLN2 | 1 | 0 | 0 | 0 | 0 | 0 |
| FBLN5 | 0 | 0 | 1 | 0 | 0 | 0 |
| FBN3 | 0 | 0 | 0 | 0 | 1 | 0 |
| FBP1 | 0 | 1 | 0 | 0 | 0 | 0 |
| FBRSL1 | 1 | 0 | 0 | 0 | 0 | 0 |
| FBXL12 | 0 | 0 | 0 | 0 | 1 | 0 |
| FBXL14 | 0 | 0 | 0 | 1 | 0 | 0 |
| FBXL17 | 1 | 0 | 1 | 0 | 1 | 0 |
| FBXL18 | 0 | 1 | 0 | 0 | 0 | 0 |
| FBXL3 | 0 | 0 | 0 | 0 | 1 | 0 |
| FBXL5 | 0 | 0 | 0 | 0 | 1 | 0 |
| FBXO10 | 1 | 0 | 0 | 0 | 0 | 0 |
| FBXO18 | 0 | 0 | 1 | 0 | 0 | 0 |
| FBXO21 | 1 | 0 | 0 | 0 | 0 | 0 |
| FBXO27 | 0 | 0 | 0 | 0 | 1 | 0 |
| FBXO3 | 0 | 0 | 0 | 0 | 1 | 0 |
| FBXO33 | 0 | 0 | 0 | 1 | 1 | 0 |
| FBXO34 | 0 | 0 | 1 | 0 | 1 | 0 |
| FBXO38 | 0 | 0 | 0 | 1 | 0 | 0 |
| FBXO45 | 0 | 0 | 0 | 0 | 1 | 0 |
| FBXO5 | 1 | 0 | 0 | 0 | 0 | 0 |
| FBXO7 | 0 | 0 | 0 | 0 | 1 | 0 |
| FBXO9 | 0 | 0 | 0 | 0 | 1 | 0 |
| FBXW11 | 1 | 0 | 0 | 1 | 1 | 0 |
| FBXW7 | 0 | 0 | 1 | 1 | 0 | 0 |
| FCF1 | 1 | 0 | 0 | 1 | 1 | 0 |
| FCHSD2 | 0 | 0 | 0 | 1 | 0 | 0 |
| FDPS | 0 | 0 | 1 | 0 | 0 | 0 |
| FDXACB1 | 0 | 0 | 0 | 0 | 1 | 0 |
| FEM1B | 0 | 0 | 0 | 1 | 0 | 0 |
| FEM1C | 1 | 0 | 0 | 0 | 0 | 0 |
| FERMT2 | 1 | 0 | 1 | 0 | 0 | 0 |
| FEZ1 | 0 | 0 | 0 | 0 | 1 | 0 |
| FEZ2 | 0 | 0 | 0 | 0 | 1 | 0 |
| FGD5-AS1 | 0 | 0 | 1 | 0 | 0 | 0 |
| FGF13 | 1 | 0 | 0 | 0 | 0 | 0 |
| FGF18 | 0 | 1 | 0 | 0 | 0 | 0 |
| FGF2 | 0 | 0 | 0 | 0 | 1 | 0 |
| FGFR1OP2 | 0 | 0 | 0 | 1 | 0 | 0 |
| FGL2 | 0 | 1 | 0 | 0 | 0 | 0 |
| FHAD1 | 0 | 1 | 0 | 0 | 0 | 0 |
| FHL2 | 1 | 0 | 1 | 0 | 0 | 0 |
| FICD | 1 | 0 | 0 | 0 | 0 | 0 |
| FIGN | 0 | 1 | 0 | 0 | 0 | 0 |
| FKBP10 | 0 | 0 | 1 | 0 | 0 | 0 |
| FKBP1A | 1 | 0 | 1 | 1 | 0 | 0 |
| FKBP4 | 1 | 0 | 0 | 0 | 0 | 0 |
| FLCN | 0 | 0 | 0 | 0 | 1 | 0 |
| FLJ12825 | 0 | 1 | 0 | 0 | 0 | 0 |
| FLJ27365 | 1 | 0 | 0 | 0 | 0 | 0 |
| FLJ32255 | 0 | 0 | 0 | 1 | 0 | 0 |
| FLJ33996 | 0 | 1 | 0 | 0 | 0 | 0 |
| FLJ37201 | 0 | 1 | 0 | 0 | 0 | 0 |
| FLNB | 0 | 0 | 1 | 0 | 0 | 0 |
| FLOT2 | 0 | 0 | 1 | 0 | 0 | 0 |
| FLVCR1 | 0 | 0 | 1 | 0 | 1 | 0 |
| FMNL2 | 1 | 0 | 0 | 0 | 0 | 0 |
| FMR1 | 0 | 0 | 0 | 1 | 0 | 0 |
| FMR1NB | 0 | 0 | 0 | 0 | 1 | 0 |
| FN3K | 0 | 0 | 0 | 0 | 1 | 0 |
| FNBP1L | 1 | 0 | 1 | 0 | 0 | 0 |
| FNBP4 | 1 | 0 | 1 | 0 | 1 | 0 |
| FNDC3A | 0 | 0 | 1 | 0 | 0 | 0 |
| FNDC3B | 1 | 1 | 1 | 0 | 0 | 0 |
| FNTA | 1 | 0 | 0 | 0 | 1 | 0 |
| FOXA2 | 0 | 0 | 0 | 0 | 1 | 0 |
| FOXG1 | 0 | 0 | 0 | 0 | 1 | 0 |
| FOXI2 | 0 | 0 | 0 | 0 | 1 | 0 |
| FOXJ3 | 1 | 0 | 0 | 1 | 0 | 0 |
| FOXK1 | 1 | 0 | 0 | 0 | 1 | 0 |
| FOXK2 | 1 | 0 | 1 | 0 | 0 | 0 |
| FOXL2 | 0 | 0 | 0 | 0 | 1 | 0 |
| FOXO3B | 0 | 0 | 0 | 0 | 1 | 0 |
| FOXRED2 | 0 | 0 | 1 | 0 | 0 | 0 |
| FPR2 | 0 | 1 | 0 | 0 | 0 | 0 |
| FPR3 | 0 | 0 | 0 | 0 | 1 | 0 |
| FRAT2 | 0 | 0 | 0 | 0 | 1 | 0 |
| FRK | 1 | 0 | 0 | 0 | 0 | 0 |
| FRMD6 | 1 | 0 | 0 | 0 | 0 | 0 |
| FRS2 | 1 | 0 | 0 | 0 | 0 | 0 |
| FRS3 | 0 | 1 | 0 | 0 | 1 | 0 |
| FRYL | 0 | 1 | 0 | 0 | 0 | 0 |
| FSCN1 | 0 | 0 | 1 | 0 | 0 | 0 |
| FSCN3 | 0 | 0 | 0 | 0 | 1 | 0 |
| FTH1 | 1 | 0 | 0 | 0 | 0 | 0 |
| FTL | 1 | 0 | 0 | 0 | 0 | 0 |
| FTO | 0 | 0 | 1 | 0 | 0 | 0 |
| FTX | 0 | 0 | 1 | 0 | 0 | 0 |
| FUBP1 | 1 | 0 | 1 | 0 | 0 | 0 |
| FUBP3 | 0 | 0 | 1 | 1 | 0 | 0 |
| FUS | 1 | 0 | 1 | 0 | 0 | 0 |
| FUSIP1 | 0 | 0 | 0 | 1 | 0 | 0 |
| FUT5 | 1 | 0 | 0 | 0 | 1 | 0 |
| FXN | 0 | 0 | 1 | 0 | 1 | 0 |
| FXR1 | 1 | 0 | 0 | 0 | 0 | 0 |
| FYB | 0 | 0 | 0 | 0 | 1 | 0 |
| FYN | 0 | 0 | 1 | 1 | 1 | 0 |
| FYTTD1 | 1 | 0 | 0 | 0 | 0 | 0 |
| FZD10 | 0 | 0 | 0 | 0 | 1 | 0 |
| FZD4 | 0 | 0 | 0 | 0 | 1 | 0 |
| FZD5 | 0 | 0 | 0 | 0 | 1 | 0 |
| FZR1 | 0 | 0 | 0 | 0 | 1 | 0 |
| G2E3 | 1 | 0 | 1 | 1 | 1 | 0 |
| G3BP1 | 1 | 0 | 1 | 0 | 0 | 0 |
| G3BP2 | 1 | 0 | 0 | 1 | 1 | 0 |
| GABARAP | 1 | 0 | 0 | 0 | 1 | 0 |
| GABARAPL2 | 1 | 0 | 0 | 0 | 1 | 0 |
| GABPB1-AS1 | 0 | 0 | 1 | 0 | 0 | 0 |
| GABRA3 | 0 | 0 | 0 | 0 | 1 | 0 |
| GABRE | 0 | 0 | 1 | 0 | 0 | 0 |
| GABRQ | 0 | 0 | 0 | 0 | 1 | 0 |
| GAK | 0 | 0 | 1 | 0 | 0 | 0 |
| GALC | 0 | 1 | 0 | 0 | 0 | 0 |
| GALNT2 | 0 | 0 | 1 | 0 | 1 | 0 |
| GALNT7 | 0 | 0 | 0 | 0 | 1 | 0 |
| GALR3 | 0 | 1 | 0 | 0 | 0 | 0 |
| GALT | 0 | 0 | 0 | 0 | 1 | 0 |
| GAN | 0 | 0 | 0 | 0 | 1 | 0 |
| GANAB | 0 | 0 | 1 | 0 | 0 | 0 |
| GAPDH | 1 | 0 | 0 | 0 | 0 | 0 |
| GAPDHP62 | 0 | 0 | 0 | 0 | 1 | 0 |
| GARS | 0 | 0 | 1 | 0 | 0 | 0 |
| GAS5 | 0 | 0 | 0 | 0 | 1 | 0 |
| GATAD2B | 0 | 0 | 1 | 0 | 0 | 0 |
| GATC | 1 | 0 | 0 | 0 | 0 | 0 |
| GATSL3 | 0 | 0 | 0 | 0 | 1 | 0 |
| GBAS | 0 | 0 | 1 | 0 | 1 | 0 |
| GBE1 | 1 | 0 | 1 | 0 | 1 | 0 |
| GBP1 | 0 | 0 | 0 | 1 | 0 | 0 |
| GBP2 | 0 | 0 | 0 | 1 | 0 | 0 |
| GBP4 | 0 | 0 | 0 | 1 | 0 | 1 |
| GBP5 | 0 | 0 | 0 | 1 | 0 | 0 |
| GCA | 0 | 0 | 0 | 1 | 0 | 0 |
| GCH1 | 0 | 0 | 1 | 1 | 1 | 0 |
| GCN1L1 | 1 | 0 | 0 | 0 | 0 | 0 |
| GCOM1 | 1 | 0 | 0 | 1 | 0 | 0 |
| GDA | 0 | 0 | 1 | 0 | 0 | 0 |
| GDI1 | 1 | 0 | 1 | 0 | 0 | 0 |
| GDI2 | 0 | 0 | 0 | 1 | 0 | 0 |
| GEMIN2 | 0 | 0 | 0 | 0 | 1 | 0 |
| GEMIN4 | 0 | 0 | 1 | 0 | 0 | 0 |
| GEMIN6 | 1 | 0 | 0 | 0 | 1 | 0 |
| GEMIN7 | 0 | 0 | 0 | 0 | 1 | 0 |
| GFM1 | 1 | 0 | 0 | 1 | 0 | 0 |
| GFOD1 | 0 | 0 | 1 | 0 | 0 | 0 |
| GFPT1 | 1 | 0 | 0 | 0 | 0 | 0 |
| GGA2 | 0 | 0 | 0 | 0 | 1 | 0 |
| GGCX | 0 | 0 | 0 | 0 | 1 | 0 |
| GGPS1 | 1 | 0 | 0 | 1 | 0 | 0 |
| GGT7 | 0 | 1 | 0 | 0 | 0 | 0 |
| GHc-210E9.2 | 0 | 0 | 0 | 0 | 1 | 0 |
| GHITM | 1 | 0 | 0 | 1 | 0 | 0 |
| GID4 | 0 | 0 | 0 | 0 | 1 | 1 |
| GID8 | 1 | 0 | 0 | 0 | 0 | 0 |
| GIGYF1 | 0 | 0 | 1 | 0 | 0 | 0 |
| GIMAP1 | 0 | 0 | 0 | 1 | 0 | 0 |
| GIMAP8 | 0 | 0 | 0 | 1 | 0 | 0 |
| GINS1 | 1 | 0 | 1 | 0 | 1 | 0 |
| GIT2 | 1 | 0 | 0 | 0 | 0 | 0 |
| GJA9 | 0 | 0 | 0 | 0 | 1 | 0 |
| GK5 | 0 | 0 | 1 | 0 | 0 | 0 |
| GLB1 | 1 | 0 | 1 | 0 | 1 | 0 |
| GLCCI1 | 0 | 1 | 1 | 1 | 1 | 0 |
| GLG1 | 0 | 0 | 1 | 0 | 0 | 0 |
| GLIPR2 | 0 | 0 | 0 | 1 | 1 | 0 |
| GLO1 | 1 | 0 | 0 | 0 | 0 | 0 |
| GLRX2 | 0 | 0 | 0 | 0 | 1 | 0 |
| GLRX3 | 1 | 0 | 0 | 0 | 0 | 0 |
| GLRX5 | 0 | 0 | 0 | 0 | 1 | 0 |
| GLS | 1 | 0 | 0 | 1 | 1 | 0 |
| GLTP | 1 | 0 | 0 | 1 | 0 | 0 |
| GLUD1 | 0 | 0 | 0 | 1 | 0 | 0 |
| GLUD2 | 0 | 0 | 0 | 0 | 1 | 0 |
| GM2A | 0 | 1 | 0 | 0 | 0 | 0 |
| GMDS | 0 | 0 | 1 | 0 | 1 | 0 |
| GMEB2 | 0 | 0 | 0 | 0 | 1 | 0 |
| GMFB | 1 | 0 | 0 | 0 | 1 | 0 |
| GMFG | 0 | 0 | 0 | 0 | 1 | 0 |
| GMPR2 | 0 | 0 | 0 | 0 | 1 | 0 |
| GNA12 | 0 | 0 | 1 | 0 | 1 | 0 |
| GNA13 | 1 | 0 | 0 | 0 | 0 | 1 |
| GNAI1 | 0 | 0 | 1 | 0 | 0 | 0 |
| GNAI2 | 0 | 0 | 1 | 0 | 0 | 0 |
| GNAI3 | 1 | 0 | 0 | 1 | 0 | 0 |
| GNAL | 0 | 0 | 1 | 0 | 0 | 0 |
| GNAQ | 1 | 0 | 0 | 1 | 0 | 0 |
| GNAS | 1 | 0 | 1 | 0 | 0 | 0 |
| GNB1 | 1 | 0 | 0 | 1 | 0 | 0 |
| GNB1L | 0 | 0 | 1 | 0 | 0 | 0 |
| GNB2L1 | 1 | 0 | 0 | 0 | 0 | 0 |
| GNB4 | 1 | 0 | 1 | 0 | 1 | 0 |
| GNG10 | 0 | 0 | 0 | 1 | 0 | 0 |
| GNG12 | 1 | 0 | 0 | 0 | 0 | 0 |
| GNG2 | 1 | 0 | 0 | 1 | 0 | 0 |
| GNG5P2 | 0 | 0 | 0 | 0 | 1 | 0 |
| GNL3 | 0 | 0 | 1 | 1 | 0 | 0 |
| GNPDA1 | 1 | 0 | 0 | 1 | 1 | 0 |
| GNPDA2 | 0 | 0 | 0 | 0 | 1 | 0 |
| GNPNAT1 | 1 | 0 | 0 | 0 | 1 | 0 |
| GNPTAB | 0 | 0 | 0 | 1 | 0 | 0 |
| GNS | 0 | 0 | 0 | 0 | 1 | 0 |
| GOLGA3 | 0 | 1 | 0 | 0 | 0 | 0 |
| GOLGA4 | 0 | 0 | 1 | 1 | 0 | 0 |
| GOLGA5P1 | 0 | 0 | 0 | 0 | 1 | 0 |
| GOLGA7 | 0 | 0 | 0 | 1 | 0 | 0 |
| GOLIM4 | 1 | 0 | 0 | 0 | 0 | 0 |
| GOLPH3 | 0 | 0 | 1 | 0 | 0 | 0 |
| GOLT1B | 1 | 0 | 0 | 1 | 1 | 0 |
| GON4L | 0 | 0 | 1 | 0 | 0 | 0 |
| GORASP2 | 0 | 0 | 0 | 1 | 0 | 0 |
| GOSR1 | 0 | 0 | 1 | 0 | 0 | 0 |
| GOSR2 | 1 | 0 | 1 | 0 | 1 | 0 |
| GPATCH2L | 1 | 0 | 0 | 0 | 0 | 0 |
| GPATCH8 | 0 | 0 | 1 | 0 | 0 | 0 |
| GPC5 | 0 | 0 | 1 | 0 | 0 | 0 |
| GPCPD1 | 1 | 0 | 1 | 0 | 0 | 0 |
| GPD1L | 0 | 0 | 0 | 0 | 1 | 0 |
| GPD2 | 1 | 0 | 0 | 0 | 0 | 0 |
| GPI | 1 | 0 | 1 | 0 | 0 | 0 |
| GPM6A | 1 | 0 | 0 | 0 | 0 | 0 |
| GPR12 | 0 | 0 | 0 | 0 | 1 | 1 |
| GPR133 | 0 | 0 | 0 | 0 | 1 | 0 |
| GPR137 | 0 | 0 | 0 | 0 | 1 | 0 |
| GPR137B | 0 | 0 | 0 | 0 | 1 | 0 |
| GPR144 | 0 | 1 | 0 | 0 | 0 | 0 |
| GPR160 | 0 | 0 | 0 | 0 | 1 | 0 |
| GPR171 | 0 | 0 | 0 | 1 | 0 | 0 |
| GPR180 | 0 | 0 | 1 | 0 | 0 | 0 |
| GPR183 | 0 | 0 | 0 | 1 | 0 | 0 |
| GPR56 | 1 | 0 | 0 | 0 | 0 | 0 |
| GPR63 | 0 | 0 | 0 | 0 | 1 | 0 |
| GPR75-ASB3 | 1 | 0 | 0 | 0 | 0 | 0 |
| GPRC5B | 0 | 1 | 0 | 0 | 0 | 0 |
| GPRC5C | 0 | 0 | 0 | 0 | 1 | 0 |
| GPRIN1 | 0 | 1 | 0 | 0 | 0 | 0 |
| GPRIN2 | 0 | 0 | 0 | 0 | 0 | 1 |
| GPS2 | 1 | 0 | 0 | 0 | 0 | 0 |
| GPT2 | 0 | 0 | 0 | 0 | 1 | 0 |
| GPX1 | 1 | 0 | 0 | 0 | 0 | 0 |
| GRB2 | 0 | 0 | 0 | 0 | 1 | 0 |
| GREB1L | 0 | 0 | 1 | 0 | 0 | 0 |
| GRIK4 | 0 | 1 | 0 | 0 | 0 | 0 |
| GRK6 | 0 | 0 | 0 | 1 | 0 | 0 |
| GRN | 0 | 1 | 1 | 0 | 0 | 0 |
| GRP | 0 | 0 | 0 | 0 | 1 | 0 |
| GRPEL1 | 0 | 0 | 0 | 0 | 1 | 0 |
| GRPEL2 | 0 | 0 | 0 | 0 | 1 | 0 |
| GRSF1 | 1 | 0 | 0 | 0 | 0 | 0 |
| GRWD1 | 1 | 0 | 0 | 0 | 1 | 0 |
| GS1-124K5.2 | 0 | 0 | 0 | 0 | 1 | 0 |
| GS1-257G1.1 | 0 | 0 | 0 | 0 | 1 | 0 |
| GSK3B | 1 | 0 | 1 | 0 | 1 | 0 |
| GSN | 0 | 0 | 0 | 0 | 1 | 0 |
| GSTM3 | 0 | 0 | 0 | 0 | 1 | 0 |
| GSTP1 | 1 | 0 | 0 | 0 | 0 | 0 |
| GSTTP1 | 0 | 0 | 0 | 0 | 1 | 0 |
| GTF2A1 | 1 | 0 | 1 | 1 | 1 | 0 |
| GTF2A1L | 0 | 0 | 0 | 0 | 1 | 0 |
| GTF2E2 | 1 | 0 | 0 | 0 | 0 | 0 |
| GTF2F2 | 0 | 0 | 1 | 0 | 0 | 0 |
| GTF2I | 0 | 0 | 1 | 0 | 0 | 0 |
| GTF2IP1 | 0 | 0 | 1 | 0 | 0 | 0 |
| GTF3A | 0 | 0 | 0 | 1 | 0 | 0 |
| GTF3C4 | 0 | 0 | 0 | 0 | 1 | 0 |
| GTPBP1 | 0 | 1 | 1 | 0 | 0 | 0 |
| GTPBP3 | 1 | 0 | 1 | 0 | 1 | 0 |
| GTPBP4 | 0 | 0 | 1 | 0 | 1 | 0 |
| GTPBP8 | 0 | 0 | 0 | 0 | 1 | 0 |
| GTSE1 | 0 | 0 | 1 | 0 | 1 | 0 |
| GULP1 | 1 | 0 | 0 | 0 | 0 | 0 |
| GYG2 | 1 | 0 | 0 | 0 | 0 | 0 |
| GYPB | 0 | 0 | 0 | 0 | 1 | 0 |
| GYPE | 0 | 0 | 0 | 0 | 1 | 0 |
| H1F0 | 0 | 0 | 1 | 0 | 0 | 0 |
| H1FX | 0 | 0 | 0 | 0 | 1 | 0 |
| H2AFV | 1 | 0 | 1 | 0 | 0 | 0 |
| H2AFY | 1 | 0 | 0 | 1 | 0 | 0 |
| H2AFZ | 1 | 0 | 0 | 0 | 0 | 0 |
| H3F3A | 1 | 0 | 0 | 1 | 0 | 0 |
| H3F3AP5 | 0 | 0 | 0 | 0 | 1 | 0 |
| H3F3B | 1 | 0 | 0 | 1 | 0 | 0 |
| H3F3C | 0 | 0 | 0 | 0 | 1 | 0 |
| H6PD | 0 | 0 | 1 | 0 | 1 | 0 |
| HADH | 0 | 0 | 0 | 0 | 1 | 0 |
| HAND2 | 0 | 0 | 0 | 0 | 1 | 0 |
| HAUS2 | 1 | 0 | 0 | 0 | 1 | 0 |
| HAUS6 | 0 | 0 | 1 | 0 | 0 | 0 |
| HBE1 | 0 | 0 | 0 | 0 | 1 | 0 |
| HBG2 | 0 | 0 | 0 | 0 | 1 | 0 |
| HBP1 | 0 | 0 | 0 | 0 | 1 | 0 |
| HCFC1 | 1 | 0 | 1 | 0 | 0 | 0 |
| hCG_1757335 | 0 | 0 | 0 | 1 | 0 | 0 |
| HCG17 | 0 | 0 | 1 | 0 | 0 | 0 |
| HCG18 | 0 | 0 | 1 | 1 | 0 | 0 |
| HCLS1 | 0 | 0 | 0 | 1 | 0 | 0 |
| HCP5 | 0 | 0 | 0 | 0 | 1 | 0 |
| HDAC1 | 1 | 0 | 0 | 0 | 0 | 0 |
| HDAC2 | 1 | 0 | 0 | 0 | 0 | 0 |
| HDAC5 | 0 | 0 | 0 | 0 | 1 | 0 |
| HDC | 0 | 1 | 0 | 0 | 0 | 0 |
| HDDC2 | 1 | 0 | 1 | 0 | 0 | 0 |
| HDGF | 1 | 0 | 1 | 0 | 0 | 0 |
| HDHD1 | 0 | 0 | 0 | 0 | 1 | 0 |
| HDLBP | 1 | 0 | 0 | 0 | 0 | 0 |
| HEATR2 | 0 | 0 | 0 | 0 | 1 | 0 |
| HEATR3 | 1 | 0 | 0 | 0 | 1 | 0 |
| HEATR5A | 0 | 0 | 1 | 0 | 1 | 0 |
| HECA | 0 | 0 | 0 | 0 | 1 | 0 |
| HELLS | 1 | 0 | 1 | 0 | 0 | 0 |
| HERC4 | 0 | 0 | 1 | 1 | 0 | 0 |
| HERC5 | 0 | 0 | 0 | 0 | 1 | 0 |
| HERPUD1 | 1 | 0 | 1 | 1 | 1 | 0 |
| HES6 | 1 | 0 | 0 | 0 | 0 | 0 |
| HFE | 0 | 0 | 0 | 1 | 0 | 0 |
| HFM1 | 1 | 0 | 1 | 0 | 0 | 0 |
| HGSNAT | 0 | 1 | 1 | 0 | 1 | 0 |
| HHAT | 1 | 0 | 0 | 0 | 0 | 0 |
| HIATL1 | 1 | 0 | 0 | 0 | 1 | 0 |
| HIBADH | 1 | 0 | 0 | 0 | 0 | 0 |
| HIF1A | 1 | 0 | 1 | 1 | 0 | 0 |
| HIF1AN | 1 | 1 | 1 | 0 | 1 | 1 |
| HIGD1A | 1 | 0 | 0 | 1 | 1 | 0 |
| HIGD2A | 1 | 0 | 0 | 0 | 1 | 0 |
| HIN1L | 0 | 0 | 0 | 0 | 1 | 0 |
| HINT1 | 1 | 0 | 0 | 0 | 0 | 0 |
| HINT1P1 | 0 | 0 | 0 | 0 | 1 | 0 |
| HINT3 | 1 | 0 | 0 | 0 | 1 | 0 |
| HIP1 | 0 | 0 | 1 | 0 | 0 | 0 |
| HIPK1 | 1 | 0 | 1 | 0 | 1 | 0 |
| HIPK2 | 1 | 0 | 0 | 1 | 0 | 0 |
| HIRA | 0 | 0 | 0 | 0 | 1 | 0 |
| HIST1H1B | 1 | 0 | 0 | 0 | 0 | 0 |
| HIST1H1C | 1 | 0 | 0 | 0 | 0 | 0 |
| HIST1H2AB | 1 | 0 | 0 | 0 | 0 | 0 |
| HIST1H2AG | 1 | 0 | 0 | 0 | 0 | 0 |
| HIST1H2AH | 1 | 0 | 0 | 0 | 0 | 0 |
| HIST1H2AI | 1 | 0 | 0 | 0 | 0 | 0 |
| HIST1H2AM | 1 | 0 | 0 | 0 | 0 | 0 |
| HIST1H2BC | 1 | 1 | 0 | 0 | 0 | 0 |
| HIST1H2BJ | 1 | 0 | 0 | 0 | 0 | 0 |
| HIST1H2BK | 1 | 0 | 0 | 0 | 0 | 0 |
| HIST1H2BL | 1 | 0 | 0 | 0 | 0 | 0 |
| HIST1H2BO | 1 | 0 | 0 | 0 | 0 | 0 |
| HIST1H3A | 0 | 0 | 0 | 0 | 1 | 0 |
| HIST1H3B | 1 | 0 | 0 | 0 | 0 | 0 |
| HIST1H3C | 0 | 0 | 0 | 0 | 1 | 0 |
| HIST1H3D | 0 | 0 | 1 | 0 | 0 | 0 |
| HIST1H3F | 1 | 0 | 0 | 0 | 0 | 0 |
| HIST1H3H | 1 | 0 | 0 | 0 | 0 | 0 |
| HIST1H3J | 1 | 0 | 0 | 0 | 0 | 0 |
| HIST1H4A | 0 | 0 | 0 | 0 | 1 | 0 |
| HIST1H4B | 1 | 0 | 0 | 0 | 0 | 0 |
| HIST1H4C | 1 | 0 | 0 | 0 | 0 | 0 |
| HIST1H4D | 1 | 0 | 0 | 0 | 0 | 0 |
| HIST1H4E | 1 | 0 | 0 | 0 | 0 | 0 |
| HIST1H4F | 0 | 0 | 0 | 0 | 1 | 0 |
| HIST1H4H | 1 | 0 | 0 | 0 | 0 | 0 |
| HIST1H4I | 1 | 0 | 0 | 0 | 0 | 0 |
| HIST1H4J | 1 | 0 | 0 | 0 | 0 | 0 |
| HIST1H4K | 1 | 0 | 0 | 0 | 0 | 0 |
| HIST2H2AA3 | 1 | 0 | 0 | 0 | 0 | 0 |
| HIST2H2AA4 | 1 | 0 | 0 | 0 | 0 | 0 |
| HIST2H2AB | 1 | 0 | 0 | 0 | 0 | 0 |
| HIST2H2AC | 1 | 0 | 0 | 0 | 0 | 0 |
| HIST2H2BE | 0 | 0 | 0 | 0 | 1 | 0 |
| HIST2H3A | 1 | 0 | 0 | 0 | 0 | 0 |
| HIST2H3C | 1 | 0 | 0 | 0 | 0 | 0 |
| HIST2H3D | 1 | 0 | 0 | 0 | 0 | 0 |
| HIST2H4A | 1 | 0 | 0 | 0 | 0 | 0 |
| HIST2H4B | 1 | 0 | 0 | 0 | 0 | 0 |
| HIST4H4 | 1 | 0 | 0 | 0 | 0 | 0 |
| HIVEP2 | 0 | 0 | 0 | 1 | 0 | 0 |
| HK2 | 1 | 0 | 0 | 1 | 0 | 0 |
| HLA-A | 0 | 0 | 1 | 0 | 0 | 0 |
| HLA-B | 1 | 0 | 1 | 1 | 0 | 0 |
| HLA-C | 0 | 0 | 1 | 0 | 0 | 0 |
| HLTF | 1 | 0 | 1 | 0 | 0 | 0 |
| HMG20A | 1 | 0 | 1 | 0 | 0 | 0 |
| HMGA1 | 1 | 0 | 1 | 0 | 0 | 0 |
| HMGA2 | 1 | 0 | 0 | 0 | 0 | 0 |
| HMGB1 | 1 | 0 | 0 | 1 | 0 | 0 |
| HMGB2 | 1 | 0 | 0 | 0 | 0 | 0 |
| HMGB3P4 | 0 | 0 | 0 | 0 | 1 | 0 |
| HMGCR | 1 | 0 | 1 | 0 | 0 | 0 |
| HMGCS1 | 0 | 0 | 1 | 0 | 0 | 0 |
| HMGN1 | 1 | 0 | 0 | 1 | 0 | 0 |
| HMGN1P26 | 0 | 0 | 0 | 0 | 1 | 0 |
| HMGN2 | 1 | 0 | 0 | 1 | 0 | 0 |
| HMGN3 | 1 | 0 | 0 | 0 | 0 | 0 |
| HMGN4 | 1 | 0 | 0 | 1 | 0 | 0 |
| HMGXB4 | 0 | 0 | 1 | 0 | 0 | 0 |
| HMOX1 | 1 | 1 | 0 | 0 | 0 | 0 |
| HN1 | 1 | 0 | 0 | 1 | 0 | 0 |
| HN1L | 1 | 0 | 0 | 0 | 0 | 0 |
| HNF4G | 0 | 0 | 0 | 0 | 0 | 1 |
| HNRNPA0 | 1 | 0 | 1 | 0 | 0 | 0 |
| HNRNPA1 | 1 | 0 | 0 | 1 | 0 | 0 |
| HNRNPA1L2 | 1 | 0 | 0 | 0 | 0 | 0 |
| HNRNPA2B1 | 1 | 0 | 1 | 0 | 0 | 0 |
| HNRNPA3 | 1 | 0 | 0 | 1 | 0 | 0 |
| HNRNPAB | 1 | 0 | 0 | 1 | 0 | 0 |
| HNRNPC | 1 | 0 | 1 | 1 | 0 | 0 |
| HNRNPD | 1 | 1 | 1 | 1 | 0 | 0 |
| HNRNPF | 1 | 0 | 1 | 0 | 0 | 0 |
| HNRNPH1 | 1 | 0 | 0 | 1 | 0 | 0 |
| HNRNPH2 | 1 | 0 | 0 | 1 | 0 | 0 |
| HNRNPK | 1 | 0 | 1 | 1 | 0 | 0 |
| HNRNPL | 1 | 0 | 0 | 0 | 0 | 0 |
| HNRNPM | 1 | 0 | 0 | 0 | 0 | 0 |
| HNRNPR | 1 | 0 | 1 | 1 | 0 | 0 |
| HNRNPU | 1 | 0 | 0 | 1 | 0 | 0 |
| HNRNPU-AS1 | 0 | 0 | 1 | 0 | 0 | 0 |
| HNRPDL | 1 | 0 | 0 | 1 | 0 | 0 |
| HNRPLL | 1 | 0 | 0 | 0 | 0 | 0 |
| HOOK2 | 0 | 0 | 1 | 0 | 0 | 0 |
| HOOK3 | 1 | 0 | 0 | 0 | 1 | 0 |
| HOXA3 | 1 | 0 | 0 | 0 | 0 | 0 |
| HOXA4 | 1 | 0 | 0 | 0 | 0 | 0 |
| HOXB3 | 0 | 0 | 0 | 0 | 1 | 0 |
| HOXB4 | 0 | 0 | 0 | 0 | 1 | 0 |
| HOXB7 | 1 | 0 | 0 | 0 | 1 | 0 |
| HOXB8 | 0 | 0 | 0 | 0 | 1 | 0 |
| HOXC10 | 0 | 0 | 1 | 0 | 0 | 0 |
| HP1BP3 | 1 | 0 | 1 | 1 | 0 | 0 |
| HPCA | 0 | 0 | 0 | 0 | 1 | 0 |
| HPCAL1 | 1 | 0 | 0 | 0 | 1 | 0 |
| HPDL | 0 | 0 | 0 | 0 | 1 | 0 |
| HPRT1 | 1 | 0 | 0 | 1 | 0 | 0 |
| HPS3 | 0 | 0 | 0 | 1 | 1 | 0 |
| HPS5 | 0 | 0 | 0 | 1 | 1 | 0 |
| HRBL | 0 | 1 | 0 | 0 | 0 | 0 |
| HS2ST1 | 0 | 0 | 0 | 1 | 0 | 0 |
| HS3ST3A1 | 0 | 0 | 0 | 0 | 1 | 0 |
| HS3ST3B1 | 0 | 0 | 0 | 0 | 1 | 0 |
| hsa-mir-744 | 0 | 0 | 0 | 0 | 1 | 0 |
| HSBP1 | 1 | 0 | 0 | 0 | 0 | 0 |
| HSD17B11 | 0 | 0 | 0 | 0 | 1 | 0 |
| HSD17B12 | 1 | 0 | 0 | 0 | 1 | 0 |
| HSD3BP4 | 0 | 0 | 0 | 0 | 0 | 1 |
| HSDL1 | 0 | 0 | 0 | 1 | 1 | 0 |
| HSDL2 | 0 | 0 | 1 | 0 | 1 | 0 |
| HSH2D | 0 | 0 | 0 | 1 | 0 | 0 |
| HSP90AA1 | 1 | 0 | 0 | 1 | 0 | 0 |
| HSP90AB1 | 1 | 0 | 1 | 0 | 0 | 0 |
| HSP90B1 | 1 | 0 | 0 | 0 | 0 | 0 |
| HSP90B2P | 0 | 0 | 0 | 0 | 1 | 0 |
| HSPA12A | 0 | 0 | 1 | 0 | 0 | 0 |
| HSPA13 | 1 | 0 | 0 | 1 | 0 | 0 |
| HSPA1A | 1 | 0 | 1 | 0 | 0 | 0 |
| HSPA1B | 1 | 0 | 1 | 0 | 0 | 0 |
| HSPA1L | 0 | 0 | 0 | 0 | 1 | 0 |
| HSPA4 | 1 | 0 | 0 | 0 | 0 | 0 |
| HSPA5 | 1 | 0 | 1 | 0 | 0 | 0 |
| HSPA8 | 1 | 0 | 0 | 1 | 0 | 0 |
| HSPA9 | 1 | 0 | 1 | 0 | 0 | 0 |
| HSPB1 | 0 | 0 | 1 | 0 | 0 | 0 |
| HSPBAP1 | 0 | 0 | 1 | 0 | 0 | 0 |
| HSPD1 | 1 | 0 | 0 | 1 | 0 | 0 |
| HSPE1 | 1 | 0 | 0 | 0 | 0 | 0 |
| HSPH1 | 1 | 0 | 0 | 0 | 0 | 0 |
| HTATIP2 | 0 | 0 | 0 | 0 | 1 | 0 |
| HTATSF1 | 0 | 0 | 0 | 1 | 0 | 0 |
| HTN1 | 0 | 1 | 0 | 0 | 0 | 0 |
| HTR1F | 0 | 0 | 0 | 0 | 1 | 0 |
| HUWE1 | 0 | 1 | 1 | 0 | 0 | 0 |
| HYAL1 | 0 | 0 | 0 | 0 | 1 | 0 |
| HYDIN2 | 0 | 1 | 0 | 0 | 0 | 0 |
| HYLS1 | 0 | 0 | 0 | 0 | 1 | 0 |
| HYOU1 | 0 | 0 | 1 | 0 | 0 | 0 |
| IARS | 0 | 0 | 1 | 0 | 0 | 0 |
| IARS2 | 0 | 0 | 0 | 0 | 1 | 0 |
| IBA57 | 0 | 0 | 0 | 0 | 1 | 0 |
| IBTK | 0 | 0 | 1 | 0 | 0 | 0 |
| ICMT | 0 | 0 | 0 | 0 | 1 | 0 |
| ID2 | 0 | 0 | 0 | 1 | 0 | 0 |
| IDE | 0 | 0 | 1 | 0 | 0 | 0 |
| IDH3A | 0 | 0 | 0 | 0 | 1 | 0 |
| IDS | 1 | 0 | 0 | 1 | 0 | 0 |
| IER2 | 1 | 0 | 1 | 1 | 0 | 0 |
| IER3 | 0 | 0 | 0 | 0 | 1 | 0 |
| IER3IP1 | 1 | 0 | 0 | 1 | 0 | 0 |
| IER5 | 1 | 0 | 0 | 0 | 1 | 0 |
| IFI16 | 1 | 0 | 0 | 1 | 0 | 0 |
| IFITM1 | 0 | 0 | 0 | 1 | 0 | 0 |
| IFNAR1 | 0 | 0 | 0 | 0 | 1 | 0 |
| IFNG | 0 | 0 | 0 | 1 | 0 | 0 |
| IFRG15 | 1 | 0 | 0 | 0 | 0 | 0 |
| IFT122 | 0 | 1 | 0 | 0 | 0 | 0 |
| IGF1 | 0 | 0 | 0 | 0 | 1 | 0 |
| IGF1R | 0 | 0 | 1 | 0 | 1 | 0 |
| IGF2BP2 | 0 | 0 | 1 | 0 | 0 | 0 |
| IGF2BP3 | 0 | 0 | 1 | 0 | 0 | 0 |
| IGF2R | 1 | 0 | 0 | 0 | 0 | 0 |
| IGFBP4 | 0 | 0 | 0 | 0 | 1 | 0 |
| IGIP | 0 | 0 | 0 | 0 | 1 | 0 |
| IGJ | 0 | 0 | 0 | 1 | 0 | 0 |
| IGKV2-18 | 0 | 0 | 0 | 0 | 0 | 1 |
| IGKV2D-18 | 0 | 0 | 0 | 0 | 0 | 1 |
| IGSF11 | 0 | 0 | 0 | 0 | 1 | 0 |
| IKZF1 | 0 | 1 | 0 | 0 | 0 | 0 |
| IKZF4 | 0 | 1 | 0 | 0 | 0 | 0 |
| IKZF5 | 0 | 0 | 0 | 1 | 0 | 0 |
| IL11 | 0 | 1 | 0 | 0 | 0 | 0 |
| IL11RA | 0 | 0 | 0 | 0 | 1 | 0 |
| IL15 | 0 | 0 | 1 | 1 | 0 | 0 |
| IL1RAP | 1 | 0 | 1 | 0 | 0 | 0 |
| IL20RB | 0 | 0 | 1 | 0 | 1 | 0 |
| IL2RB | 0 | 0 | 0 | 1 | 0 | 0 |
| IL4I1 | 1 | 0 | 1 | 0 | 1 | 0 |
| IL6ST | 1 | 0 | 0 | 1 | 0 | 0 |
| IL8 | 0 | 0 | 0 | 0 | 1 | 0 |
| ILF2 | 1 | 0 | 0 | 0 | 0 | 0 |
| ILF3 | 1 | 0 | 1 | 0 | 0 | 0 |
| ILVBL | 0 | 0 | 0 | 0 | 1 | 0 |
| IMMP1L | 0 | 0 | 0 | 0 | 1 | 0 |
| IMMP2L | 0 | 0 | 1 | 0 | 1 | 0 |
| IMMP2L-IT1 | 0 | 0 | 0 | 0 | 1 | 0 |
| IMMT | 1 | 0 | 0 | 0 | 0 | 0 |
| IMP3 | 1 | 0 | 0 | 1 | 0 | 0 |
| IMP4 | 1 | 0 | 0 | 0 | 0 | 0 |
| IMPA1 | 0 | 0 | 0 | 0 | 1 | 0 |
| IMPA2 | 0 | 0 | 1 | 0 | 0 | 0 |
| IMPACT | 0 | 0 | 0 | 0 | 1 | 0 |
| IMPAD1 | 1 | 0 | 1 | 0 | 1 | 0 |
| INADL | 0 | 0 | 0 | 0 | 0 | 1 |
| INCENP | 1 | 0 | 1 | 0 | 0 | 0 |
| INF2 | 0 | 1 | 1 | 0 | 0 | 0 |
| ING1 | 0 | 0 | 0 | 0 | 1 | 0 |
| ING5 | 0 | 0 | 0 | 0 | 1 | 0 |
| INHBA | 1 | 0 | 0 | 0 | 0 | 0 |
| INIP | 1 | 0 | 0 | 0 | 1 | 0 |
| INO80 | 0 | 0 | 1 | 0 | 0 | 0 |
| INO80D | 1 | 0 | 0 | 1 | 0 | 0 |
| INPP5A | 1 | 0 | 0 | 0 | 1 | 0 |
| INSIG1 | 0 | 0 | 1 | 1 | 0 | 0 |
| INSIG2 | 0 | 0 | 0 | 0 | 1 | 0 |
| INTS1 | 0 | 0 | 1 | 0 | 0 | 0 |
| INTS5 | 0 | 0 | 0 | 0 | 1 | 0 |
| INTS6 | 0 | 0 | 1 | 0 | 0 | 0 |
| INVS | 0 | 0 | 1 | 0 | 0 | 0 |
| IP6K1 | 0 | 0 | 0 | 0 | 1 | 0 |
| IP6K2 | 0 | 0 | 1 | 0 | 0 | 0 |
| IPO5 | 0 | 0 | 0 | 1 | 0 | 0 |
| IPO7 | 1 | 0 | 1 | 1 | 0 | 0 |
| IPO8 | 0 | 0 | 0 | 1 | 0 | 0 |
| IPP | 0 | 0 | 0 | 0 | 1 | 0 |
| IPPK | 0 | 0 | 0 | 0 | 1 | 0 |
| IPW | 0 | 0 | 0 | 1 | 0 | 0 |
| IQCG | 1 | 0 | 0 | 0 | 0 | 0 |
| IQGAP1 | 1 | 0 | 1 | 0 | 0 | 0 |
| IRAK1 | 1 | 0 | 0 | 0 | 0 | 0 |
| IRAK2 | 0 | 0 | 0 | 0 | 1 | 0 |
| IREB2 | 0 | 0 | 0 | 1 | 1 | 0 |
| IRF2BP2 | 1 | 0 | 0 | 1 | 0 | 0 |
| IRF2BPL | 1 | 0 | 1 | 0 | 1 | 0 |
| ISCA1 | 1 | 0 | 0 | 1 | 0 | 0 |
| ISCU | 0 | 0 | 0 | 0 | 1 | 0 |
| ISG20L2 | 0 | 0 | 0 | 0 | 1 | 0 |
| ISL2 | 0 | 0 | 0 | 0 | 1 | 0 |
| ISOC1 | 0 | 0 | 0 | 0 | 1 | 0 |
| IST1 | 1 | 0 | 0 | 0 | 0 | 0 |
| ITGA4 | 0 | 0 | 0 | 1 | 0 | 0 |
| ITGA6 | 1 | 0 | 0 | 1 | 0 | 0 |
| ITGA7 | 0 | 1 | 0 | 0 | 0 | 0 |
| ITGAV | 1 | 0 | 0 | 0 | 0 | 0 |
| ITGB1 | 1 | 0 | 1 | 0 | 0 | 0 |
| ITGB3 | 0 | 0 | 0 | 0 | 1 | 1 |
| ITGB3BP | 0 | 0 | 1 | 0 | 0 | 0 |
| ITGB8 | 1 | 0 | 1 | 0 | 0 | 0 |
| ITGBL1 | 0 | 0 | 0 | 0 | 1 | 0 |
| ITK | 0 | 0 | 0 | 1 | 0 | 0 |
| ITM2A | 0 | 0 | 0 | 1 | 1 | 0 |
| ITM2B | 1 | 0 | 0 | 0 | 0 | 0 |
| ITPK1 | 0 | 0 | 1 | 0 | 1 | 0 |
| ITPR1 | 0 | 0 | 1 | 0 | 0 | 0 |
| ITPRIPL2 | 0 | 0 | 1 | 0 | 0 | 0 |
| JAK2 | 0 | 0 | 1 | 0 | 0 | 0 |
| JAZF1 | 0 | 0 | 1 | 0 | 0 | 0 |
| JKAMP | 1 | 0 | 0 | 0 | 1 | 0 |
| JMJD1C | 0 | 0 | 1 | 1 | 0 | 0 |
| JPX | 0 | 0 | 1 | 0 | 0 | 0 |
| JTB | 0 | 0 | 0 | 1 | 0 | 0 |
| JUNB | 0 | 0 | 1 | 0 | 0 | 0 |
| JUND | 1 | 0 | 1 | 1 | 0 | 0 |
| JUP | 1 | 0 | 0 | 0 | 0 | 0 |
| KANK1 | 0 | 0 | 1 | 0 | 0 | 0 |
| KANK3 | 0 | 0 | 0 | 0 | 1 | 0 |
| KANSL2 | 0 | 0 | 0 | 0 | 1 | 1 |
| KAT8 | 1 | 0 | 0 | 0 | 0 | 0 |
| KATNBL1 | 0 | 0 | 1 | 0 | 1 | 0 |
| KAZALD1 | 0 | 0 | 1 | 0 | 0 | 0 |
| KAZN | 0 | 0 | 0 | 0 | 1 | 1 |
| KBTBD2 | 1 | 0 | 0 | 0 | 1 | 0 |
| KBTBD7 | 0 | 1 | 0 | 0 | 0 | 0 |
| KCMF1 | 1 | 0 | 0 | 1 | 0 | 0 |
| KCNC2 | 0 | 1 | 0 | 0 | 0 | 0 |
| KCNC4 | 0 | 0 | 1 | 0 | 0 | 0 |
| KCNE3 | 0 | 0 | 0 | 0 | 1 | 0 |
| KCNG2 | 0 | 0 | 0 | 0 | 1 | 0 |
| KCNH8 | 1 | 0 | 0 | 0 | 0 | 0 |
| KCNJ15 | 0 | 0 | 0 | 0 | 1 | 1 |
| KCNJ5 | 1 | 0 | 0 | 0 | 0 | 0 |
| KCNJ9 | 0 | 0 | 0 | 0 | 0 | 1 |
| KCNK1 | 0 | 0 | 1 | 0 | 1 | 0 |
| KCNK15 | 1 | 0 | 0 | 0 | 1 | 0 |
| KCNK3 | 0 | 0 | 0 | 0 | 1 | 0 |
| KCNMA1 | 1 | 0 | 0 | 0 | 0 | 0 |
| KCNQ4 | 0 | 0 | 0 | 0 | 0 | 1 |
| KCNQ5 | 0 | 0 | 0 | 0 | 1 | 0 |
| KCNQ5-IT1 | 0 | 0 | 0 | 0 | 1 | 0 |
| KCTD10 | 1 | 0 | 0 | 0 | 1 | 0 |
| KCTD2 | 1 | 0 | 0 | 0 | 1 | 0 |
| KCTD20 | 1 | 0 | 0 | 1 | 0 | 0 |
| KCTD7 | 0 | 0 | 1 | 0 | 1 | 0 |
| KDELC1P1 | 0 | 0 | 0 | 0 | 1 | 0 |
| KDELC2 | 0 | 0 | 0 | 1 | 0 | 0 |
| KDELR1 | 0 | 0 | 1 | 0 | 0 | 0 |
| KDELR2 | 1 | 0 | 1 | 1 | 0 | 0 |
| KDM2A | 0 | 0 | 0 | 1 | 0 | 0 |
| KDM4C | 0 | 0 | 0 | 1 | 0 | 0 |
| KDM5A | 1 | 0 | 0 | 0 | 0 | 0 |
| KDM5B | 0 | 0 | 0 | 0 | 1 | 0 |
| KDM6B | 1 | 0 | 0 | 0 | 0 | 0 |
| KDSR | 0 | 0 | 1 | 0 | 1 | 0 |
| KHDRBS1 | 1 | 0 | 1 | 1 | 0 | 0 |
| KHSRP | 0 | 0 | 1 | 0 | 0 | 0 |
| KIAA0020 | 0 | 0 | 1 | 0 | 0 | 0 |
| KIAA0101 | 1 | 0 | 0 | 0 | 0 | 0 |
| KIAA0174 | 0 | 0 | 0 | 1 | 0 | 0 |
| KIAA0226L | 0 | 0 | 1 | 0 | 0 | 0 |
| KIAA0232 | 0 | 0 | 0 | 0 | 1 | 0 |
| KIAA0240 | 0 | 0 | 0 | 1 | 0 | 0 |
| KIAA0247 | 0 | 0 | 0 | 0 | 1 | 0 |
| KIAA0355 | 1 | 0 | 0 | 0 | 0 | 0 |
| KIAA0368 | 0 | 0 | 1 | 0 | 0 | 0 |
| KIAA0391 | 0 | 0 | 0 | 0 | 1 | 0 |
| KIAA0430 | 0 | 0 | 0 | 1 | 0 | 0 |
| KIAA0494 | 0 | 0 | 0 | 0 | 1 | 0 |
| KIAA0746 | 0 | 0 | 0 | 1 | 0 | 0 |
| KIAA0753 | 0 | 0 | 1 | 0 | 0 | 0 |
| KIAA0831 | 0 | 0 | 0 | 1 | 0 | 0 |
| KIAA0922 | 0 | 0 | 0 | 1 | 0 | 0 |
| KIAA0999 | 0 | 0 | 0 | 1 | 0 | 0 |
| KIAA1191 | 0 | 0 | 0 | 1 | 0 | 0 |
| KIAA1217 | 0 | 1 | 0 | 0 | 0 | 0 |
| KIAA1267 | 0 | 0 | 0 | 1 | 0 | 0 |
| KIAA1310 | 0 | 1 | 0 | 0 | 0 | 0 |
| KIAA1429 | 0 | 1 | 0 | 0 | 0 | 0 |
| KIAA1430 | 0 | 0 | 0 | 0 | 1 | 0 |
| KIAA1456 | 1 | 0 | 0 | 0 | 0 | 0 |
| KIAA1467 | 0 | 0 | 0 | 0 | 1 | 0 |
| KIAA1549 | 0 | 0 | 1 | 0 | 0 | 0 |
| KIAA1671 | 0 | 0 | 1 | 0 | 0 | 0 |
| KIAA1715 | 1 | 0 | 0 | 0 | 0 | 0 |
| KIAA1731 | 0 | 0 | 0 | 0 | 1 | 1 |
| KIAA1881 | 0 | 1 | 0 | 0 | 0 | 0 |
| KIAA1919 | 0 | 0 | 0 | 0 | 1 | 0 |
| KIAA1949 | 0 | 0 | 0 | 1 | 0 | 0 |
| KIAA1958 | 0 | 0 | 1 | 0 | 0 | 0 |
| KIAA2018 | 0 | 0 | 0 | 0 | 1 | 0 |
| KIAA2026 | 0 | 0 | 0 | 1 | 1 | 0 |
| KIF13A | 1 | 0 | 0 | 0 | 0 | 0 |
| KIF17 | 0 | 1 | 0 | 0 | 0 | 0 |
| KIF18B | 1 | 0 | 0 | 0 | 0 | 0 |
| KIF1C | 1 | 0 | 0 | 0 | 0 | 0 |
| KIF24 | 0 | 0 | 1 | 0 | 0 | 0 |
| KIF2A | 0 | 0 | 1 | 0 | 0 | 0 |
| KIF2C | 1 | 0 | 0 | 0 | 0 | 0 |
| KIF5B | 1 | 0 | 1 | 1 | 0 | 0 |
| KIF9 | 0 | 1 | 0 | 0 | 0 | 0 |
| KIFAP3 | 1 | 0 | 0 | 0 | 1 | 0 |
| KIFC3 | 0 | 0 | 1 | 0 | 0 | 0 |
| KIR2DS4 | 0 | 1 | 0 | 0 | 0 | 0 |
| KISS1R | 0 | 0 | 0 | 0 | 1 | 0 |
| KIT | 0 | 0 | 0 | 0 | 1 | 0 |
| KLC1 | 0 | 0 | 1 | 0 | 0 | 0 |
| KLF11 | 0 | 0 | 0 | 0 | 1 | 0 |
| KLF12 | 0 | 0 | 1 | 0 | 0 | 0 |
| KLF16 | 1 | 0 | 0 | 0 | 0 | 0 |
| KLF6 | 0 | 0 | 0 | 1 | 0 | 0 |
| KLHDC10 | 1 | 0 | 1 | 0 | 0 | 0 |
| KLHDC4 | 0 | 0 | 1 | 0 | 0 | 0 |
| KLHDC7A | 1 | 0 | 0 | 0 | 0 | 0 |
| KLHL1 | 0 | 0 | 0 | 0 | 0 | 1 |
| KLHL11 | 0 | 0 | 0 | 0 | 1 | 0 |
| KLHL13 | 0 | 0 | 1 | 0 | 0 | 1 |
| KLHL15 | 0 | 0 | 1 | 0 | 0 | 0 |
| KLHL2 | 0 | 0 | 1 | 1 | 1 | 0 |
| KLHL23 | 0 | 0 | 0 | 0 | 1 | 0 |
| KLHL24 | 0 | 0 | 0 | 1 | 1 | 0 |
| KLHL34 | 0 | 0 | 0 | 0 | 1 | 0 |
| KLHL4 | 0 | 0 | 0 | 0 | 1 | 0 |
| KLHL5 | 0 | 0 | 0 | 0 | 1 | 0 |
| KLHL7 | 0 | 0 | 0 | 0 | 1 | 0 |
| KLHL8 | 1 | 0 | 0 | 0 | 1 | 0 |
| KLHL9 | 1 | 0 | 1 | 0 | 0 | 0 |
| KLK1 | 0 | 0 | 0 | 0 | 1 | 0 |
| KLK10 | 0 | 1 | 0 | 0 | 0 | 0 |
| KMT2C | 0 | 0 | 1 | 0 | 0 | 0 |
| KMT2E | 0 | 0 | 1 | 0 | 0 | 0 |
| KPNA1 | 0 | 0 | 0 | 0 | 1 | 0 |
| KPNA2 | 1 | 0 | 1 | 1 | 0 | 0 |
| KPNA3 | 0 | 0 | 1 | 1 | 0 | 0 |
| KPNA4 | 1 | 0 | 1 | 1 | 0 | 1 |
| KPNA5 | 0 | 0 | 0 | 0 | 1 | 0 |
| KPNB1 | 1 | 0 | 0 | 0 | 0 | 0 |
| KRCC1 | 0 | 0 | 0 | 1 | 0 | 0 |
| KRI1 | 0 | 0 | 1 | 0 | 0 | 0 |
| KRT12 | 0 | 0 | 0 | 0 | 1 | 0 |
| KRT18 | 0 | 0 | 0 | 0 | 0 | 1 |
| KRT222 | 0 | 0 | 0 | 0 | 1 | 0 |
| KRT72 | 0 | 1 | 0 | 0 | 0 | 0 |
| KRTAP5-2 | 0 | 0 | 0 | 0 | 0 | 1 |
| KRTAP5-4 | 0 | 0 | 0 | 0 | 0 | 1 |
| KRTAP5-5 | 0 | 0 | 0 | 0 | 0 | 1 |
| KTN1 | 1 | 0 | 1 | 0 | 0 | 0 |
| KYNU | 0 | 0 | 1 | 0 | 0 | 0 |
| L1CAM | 0 | 0 | 1 | 0 | 0 | 0 |
| L3HYPDH | 0 | 0 | 1 | 0 | 0 | 0 |
| L3MBTL4 | 0 | 1 | 0 | 0 | 0 | 0 |
| LALBA | 0 | 1 | 0 | 0 | 0 | 0 |
| LAMA1 | 0 | 0 | 1 | 0 | 0 | 0 |
| LAMA3 | 0 | 0 | 1 | 0 | 0 | 0 |
| LAMA5 | 0 | 0 | 1 | 0 | 0 | 0 |
| LAMP2 | 1 | 0 | 0 | 0 | 0 | 0 |
| LAMTOR1 | 1 | 0 | 0 | 0 | 1 | 0 |
| LAMTOR3 | 1 | 0 | 0 | 0 | 1 | 0 |
| LAMTOR5 | 1 | 0 | 0 | 0 | 0 | 0 |
| LANCL1 | 0 | 0 | 0 | 0 | 1 | 0 |
| LANCL2 | 0 | 0 | 0 | 0 | 1 | 0 |
| LANCL3 | 0 | 0 | 0 | 0 | 1 | 0 |
| LAPTM4A | 1 | 0 | 1 | 1 | 0 | 0 |
| LAPTM4B | 1 | 0 | 1 | 1 | 0 | 0 |
| LAPTM5 | 0 | 0 | 0 | 1 | 0 | 0 |
| LARP1 | 1 | 0 | 1 | 0 | 0 | 0 |
| LARP4 | 1 | 0 | 0 | 0 | 0 | 0 |
| LARS | 1 | 0 | 1 | 0 | 0 | 0 |
| LARS2 | 0 | 0 | 1 | 0 | 0 | 0 |
| LASP1 | 1 | 0 | 0 | 0 | 0 | 0 |
| LAT | 0 | 1 | 0 | 1 | 0 | 0 |
| LATS1 | 0 | 1 | 0 | 0 | 0 | 0 |
| LATS2 | 0 | 0 | 1 | 0 | 0 | 0 |
| LBH | 0 | 0 | 0 | 1 | 1 | 1 |
| LBR | 1 | 0 | 0 | 0 | 0 | 0 |
| LCA5L | 0 | 0 | 0 | 0 | 1 | 0 |
| LCK | 0 | 0 | 0 | 1 | 0 | 0 |
| LCLAT1 | 0 | 0 | 0 | 0 | 1 | 0 |
| LCORL | 0 | 0 | 1 | 0 | 1 | 0 |
| LCP2 | 0 | 0 | 0 | 1 | 0 | 0 |
| LDHA | 1 | 0 | 0 | 1 | 0 | 0 |
| LDHB | 1 | 0 | 0 | 0 | 0 | 0 |
| LDLR | 0 | 0 | 1 | 0 | 0 | 0 |
| LDLRAD3 | 1 | 0 | 0 | 0 | 1 | 0 |
| LDOC1L | 1 | 0 | 0 | 0 | 1 | 0 |
| LEF1 | 1 | 0 | 0 | 1 | 0 | 0 |
| LEMD3 | 0 | 0 | 0 | 0 | 1 | 0 |
| LENG8 | 0 | 0 | 1 | 0 | 0 | 0 |
| LEPR | 1 | 0 | 0 | 0 | 1 | 0 |
| LEPROT | 1 | 0 | 0 | 0 | 1 | 0 |
| LEPROTL1 | 0 | 0 | 0 | 0 | 1 | 0 |
| LETM1 | 1 | 0 | 1 | 0 | 0 | 0 |
| LETMD1 | 0 | 0 | 0 | 1 | 0 | 0 |
| LFNG | 0 | 0 | 0 | 1 | 0 | 0 |
| LGALS1 | 1 | 0 | 0 | 0 | 0 | 0 |
| LGALS8 | 0 | 0 | 0 | 1 | 0 | 0 |
| LGR4 | 0 | 0 | 0 | 0 | 1 | 0 |
| LHFPL2 | 1 | 0 | 0 | 0 | 0 | 0 |
| LHX6 | 0 | 0 | 0 | 0 | 1 | 0 |
| LIFR | 0 | 0 | 1 | 0 | 0 | 0 |
| LILRA1 | 0 | 1 | 0 | 0 | 0 | 0 |
| LIMA1 | 1 | 0 | 0 | 0 | 0 | 0 |
| LIMCH1 | 0 | 0 | 1 | 0 | 0 | 0 |
| LIMK1 | 0 | 1 | 0 | 0 | 0 | 0 |
| LIMS1 | 1 | 0 | 0 | 0 | 0 | 0 |
| LIN28B | 0 | 0 | 0 | 0 | 1 | 0 |
| LIN52 | 1 | 0 | 0 | 0 | 1 | 0 |
| LIN54 | 0 | 0 | 1 | 0 | 0 | 0 |
| LIN7C | 1 | 0 | 0 | 1 | 0 | 0 |
| LINC00200 | 0 | 0 | 0 | 0 | 1 | 1 |
| LINC00355 | 0 | 0 | 1 | 0 | 0 | 0 |
| LINC00470 | 0 | 0 | 1 | 0 | 0 | 0 |
| LINC00472 | 0 | 0 | 1 | 0 | 0 | 0 |
| LINC00473 | 0 | 0 | 1 | 0 | 0 | 0 |
| LINC00478 | 0 | 0 | 0 | 0 | 0 | 1 |
| LINC00526 | 0 | 0 | 0 | 0 | 1 | 0 |
| LINC00623 | 0 | 0 | 0 | 0 | 1 | 1 |
| LINC00657 | 0 | 0 | 1 | 0 | 0 | 0 |
| LINC00689 | 0 | 0 | 1 | 0 | 0 | 0 |
| LINC00887 | 0 | 0 | 1 | 0 | 0 | 0 |
| LINC00969 | 0 | 0 | 1 | 0 | 0 | 0 |
| LINC01004 | 0 | 0 | 1 | 0 | 0 | 0 |
| LINC01021 | 0 | 0 | 1 | 0 | 0 | 0 |
| LINS | 0 | 0 | 0 | 0 | 1 | 0 |
| LIX1 | 0 | 1 | 0 | 0 | 0 | 0 |
| LL0XNC01-37G1.1 | 0 | 0 | 0 | 0 | 1 | 0 |
| LL22NC03-5H6.5 | 0 | 1 | 0 | 0 | 0 | 0 |
| LMBR1 | 0 | 0 | 1 | 0 | 0 | 0 |
| LMBRD1 | 0 | 0 | 0 | 0 | 1 | 0 |
| LMF1 | 0 | 0 | 1 | 0 | 0 | 0 |
| LMNA | 1 | 0 | 0 | 0 | 0 | 0 |
| LMNB1 | 1 | 0 | 0 | 0 | 0 | 0 |
| LMO4 | 1 | 1 | 0 | 1 | 0 | 0 |
| LMO7 | 0 | 0 | 1 | 0 | 0 | 0 |
| LNPEP | 1 | 1 | 1 | 0 | 0 | 0 |
| LNX2 | 0 | 0 | 0 | 0 | 1 | 0 |
| LOC100127918 | 0 | 0 | 0 | 1 | 0 | 0 |
| LOC100128692 | 0 | 1 | 0 | 0 | 0 | 0 |
| LOC100128718 | 0 | 0 | 0 | 1 | 0 | 0 |
| LOC100129195 | 0 | 1 | 0 | 0 | 0 | 0 |
| LOC100129206 | 0 | 1 | 0 | 0 | 0 | 0 |
| LOC100129380 | 0 | 1 | 0 | 0 | 0 | 0 |
| LOC100129656 | 0 | 1 | 0 | 0 | 0 | 0 |
| LOC100133233 | 0 | 1 | 0 | 0 | 0 | 0 |
| LOC100134119 | 0 | 1 | 0 | 0 | 0 | 0 |
| LOC124685 | 0 | 0 | 0 | 0 | 1 | 0 |
| LOC144438 | 0 | 0 | 0 | 1 | 0 | 0 |
| LOC150166 | 0 | 0 | 0 | 1 | 0 | 0 |
| LOC198437 | 0 | 1 | 0 | 0 | 0 | 0 |
| LOC25845 | 0 | 0 | 0 | 1 | 0 | 0 |
| LOC26010 | 0 | 1 | 0 | 0 | 0 | 0 |
| LOC284014 | 0 | 1 | 0 | 0 | 0 | 0 |
| LOC286154 | 0 | 1 | 0 | 0 | 0 | 0 |
| LOC339047 | 0 | 0 | 0 | 1 | 0 | 0 |
| LOC339862 | 0 | 1 | 0 | 0 | 0 | 0 |
| LOC340107 | 0 | 1 | 0 | 0 | 0 | 0 |
| LOC400680 | 0 | 1 | 0 | 0 | 0 | 0 |
| LOC401312 | 0 | 1 | 0 | 0 | 0 | 0 |
| LOC401320 | 0 | 1 | 0 | 0 | 0 | 0 |
| LOC401442 | 0 | 1 | 0 | 0 | 0 | 0 |
| LOC552889 | 0 | 0 | 0 | 1 | 0 | 0 |
| LOC554174 | 0 | 1 | 0 | 0 | 0 | 0 |
| LOC572558 | 0 | 1 | 0 | 0 | 0 | 0 |
| LOC643287 | 0 | 0 | 0 | 1 | 0 | 0 |
| LOC643308 | 0 | 0 | 0 | 1 | 0 | 0 |
| LOC646934 | 0 | 1 | 0 | 0 | 0 | 0 |
| LOC653160 | 0 | 1 | 0 | 0 | 0 | 0 |
| LOC653566 | 0 | 0 | 0 | 1 | 0 | 0 |
| LOC728613 | 0 | 0 | 0 | 1 | 0 | 0 |
| LOC729839 | 0 | 0 | 0 | 1 | 0 | 0 |
| LOC729970 | 0 | 1 | 0 | 0 | 0 | 0 |
| LOC730101 | 0 | 1 | 0 | 0 | 0 | 0 |
| LONP2 | 1 | 0 | 0 | 0 | 0 | 0 |
| LONRF1 | 0 | 0 | 0 | 0 | 1 | 0 |
| LPCAT1 | 0 | 0 | 1 | 0 | 0 | 0 |
| LPCAT4 | 0 | 0 | 0 | 0 | 1 | 0 |
| LPGAT1 | 0 | 0 | 1 | 0 | 1 | 0 |
| LPIN2 | 1 | 0 | 1 | 1 | 0 | 0 |
| LPP | 1 | 0 | 1 | 0 | 0 | 0 |
| LRFN1 | 0 | 0 | 1 | 0 | 0 | 0 |
| LRP11 | 0 | 0 | 1 | 0 | 0 | 0 |
| LRP12 | 0 | 0 | 0 | 0 | 1 | 0 |
| LRP1B | 0 | 0 | 1 | 0 | 0 | 0 |
| LRP8 | 0 | 0 | 1 | 0 | 0 | 0 |
| LRPPRC | 1 | 0 | 0 | 1 | 0 | 0 |
| LRRC28 | 0 | 0 | 0 | 0 | 1 | 0 |
| LRRC47 | 0 | 0 | 0 | 1 | 0 | 0 |
| LRRC58 | 1 | 0 | 0 | 0 | 0 | 0 |
| LRRC59 | 1 | 0 | 0 | 0 | 0 | 0 |
| LRRC8A | 1 | 1 | 1 | 0 | 1 | 0 |
| LRRC8C | 0 | 0 | 0 | 0 | 1 | 0 |
| LRRC8D | 0 | 0 | 1 | 0 | 0 | 0 |
| LRRD1 | 0 | 0 | 1 | 0 | 0 | 0 |
| LRRFIP1 | 0 | 0 | 0 | 1 | 0 | 0 |
| LRRFIP2 | 0 | 0 | 1 | 0 | 0 | 0 |
| LRRN3 | 0 | 0 | 0 | 1 | 0 | 0 |
| LRRTM3 | 1 | 0 | 0 | 0 | 0 | 0 |
| LRTOMT | 1 | 0 | 0 | 0 | 0 | 0 |
| LSM1 | 0 | 0 | 0 | 1 | 0 | 0 |
| LSM11 | 0 | 0 | 0 | 0 | 1 | 0 |
| LSM14A | 1 | 0 | 0 | 1 | 0 | 0 |
| LSM14B | 1 | 0 | 0 | 0 | 1 | 0 |
| LSM4 | 1 | 0 | 0 | 0 | 0 | 0 |
| LSM5 | 1 | 0 | 0 | 1 | 0 | 0 |
| LSM6 | 0 | 0 | 0 | 1 | 0 | 0 |
| LTB | 0 | 1 | 0 | 0 | 0 | 0 |
| LUC7L | 0 | 0 | 1 | 0 | 0 | 0 |
| LUC7L2 | 0 | 0 | 1 | 0 | 0 | 0 |
| LUZP6 | 1 | 0 | 0 | 1 | 0 | 0 |
| LY75 | 0 | 0 | 0 | 1 | 0 | 0 |
| LYPD3 | 0 | 0 | 1 | 0 | 0 | 0 |
| LYPLA1 | 1 | 0 | 0 | 1 | 0 | 0 |
| LYPLA2 | 1 | 0 | 0 | 0 | 1 | 0 |
| LYPLA2P1 | 0 | 0 | 0 | 0 | 1 | 0 |
| LYPLAL1 | 0 | 0 | 0 | 0 | 1 | 0 |
| LYRM4 | 0 | 0 | 0 | 0 | 1 | 0 |
| LYRM7 | 0 | 0 | 0 | 0 | 1 | 0 |
| LYSMD2 | 0 | 0 | 0 | 1 | 0 | 0 |
| LYST | 0 | 0 | 0 | 1 | 0 | 0 |
| LYZ | 0 | 0 | 0 | 0 | 1 | 0 |
| LZIC | 1 | 0 | 0 | 0 | 1 | 0 |
| M6PR | 1 | 0 | 0 | 0 | 0 | 0 |
| MAB21L3 | 1 | 0 | 0 | 0 | 0 | 0 |
| MACC1 | 0 | 0 | 0 | 0 | 1 | 0 |
| MAD1L1 | 1 | 0 | 1 | 0 | 0 | 0 |
| MAD2L2 | 0 | 1 | 0 | 0 | 0 | 0 |
| MAFG | 1 | 0 | 1 | 0 | 1 | 0 |
| MAFK | 0 | 0 | 1 | 0 | 0 | 0 |
| MAGEA1 | 0 | 0 | 0 | 0 | 1 | 0 |
| MAGEA12 | 1 | 0 | 0 | 0 | 0 | 0 |
| MAGEA2 | 0 | 0 | 0 | 0 | 1 | 0 |
| MAGEA2B | 0 | 0 | 0 | 0 | 1 | 0 |
| MAGEA3 | 1 | 0 | 0 | 0 | 1 | 0 |
| MAGEA4 | 1 | 0 | 0 | 0 | 0 | 0 |
| MAGEA6 | 1 | 0 | 0 | 0 | 0 | 0 |
| MAGEB10 | 1 | 0 | 0 | 0 | 0 | 0 |
| MAGEF1 | 0 | 0 | 0 | 0 | 1 | 0 |
| MAGEH1 | 0 | 0 | 0 | 0 | 1 | 0 |
| MAGI1 | 0 | 0 | 1 | 0 | 0 | 0 |
| MAGOH | 0 | 0 | 0 | 1 | 0 | 0 |
| MAGOHB | 0 | 0 | 0 | 1 | 0 | 0 |
| MAGT1 | 1 | 0 | 1 | 0 | 0 | 0 |
| MAL | 0 | 0 | 0 | 1 | 0 | 1 |
| MALAT1 | 0 | 0 | 1 | 0 | 0 | 0 |
| MALSU1 | 0 | 0 | 0 | 0 | 1 | 0 |
| MALT1 | 0 | 0 | 0 | 1 | 0 | 0 |
| MAN2A1 | 0 | 0 | 0 | 1 | 0 | 0 |
| MANF | 1 | 0 | 0 | 0 | 0 | 0 |
| MAP1LC3B | 1 | 0 | 1 | 1 | 0 | 0 |
| MAP1LC3B2 | 0 | 0 | 0 | 0 | 1 | 0 |
| MAP2K1 | 0 | 0 | 0 | 0 | 1 | 0 |
| MAP2K4 | 0 | 0 | 0 | 0 | 1 | 0 |
| MAP2K5 | 1 | 0 | 0 | 0 | 0 | 0 |
| MAP2K6 | 0 | 0 | 1 | 0 | 0 | 0 |
| MAP3K1 | 0 | 0 | 0 | 1 | 0 | 0 |
| MAP3K13 | 1 | 0 | 0 | 0 | 0 | 0 |
| MAP3K2 | 0 | 0 | 0 | 1 | 0 | 0 |
| MAP4 | 1 | 0 | 1 | 0 | 0 | 0 |
| MAP4K5 | 0 | 0 | 1 | 0 | 0 | 0 |
| MAP7 | 0 | 0 | 1 | 0 | 0 | 0 |
| MAP7D1 | 0 | 0 | 1 | 0 | 0 | 0 |
| MAPK1 | 1 | 0 | 0 | 1 | 0 | 0 |
| MAPK14 | 1 | 0 | 1 | 0 | 1 | 0 |
| MAPK1IP1L | 0 | 0 | 0 | 1 | 0 | 0 |
| MAPK6 | 1 | 0 | 0 | 0 | 0 | 0 |
| MAPK8IP3 | 0 | 0 | 1 | 0 | 0 | 0 |
| MAPKAPK2 | 1 | 0 | 0 | 1 | 1 | 0 |
| MAPKAPK5-AS1 | 0 | 0 | 0 | 0 | 1 | 0 |
| MAPRE1 | 1 | 0 | 1 | 1 | 0 | 0 |
| MARCH1 | 0 | 1 | 0 | 0 | 0 | 1 |
| MARCH2 | 0 | 0 | 0 | 0 | 1 | 0 |
| MARCH5 | 1 | 0 | 1 | 0 | 1 | 0 |
| MARCH6 | 1 | 0 | 1 | 0 | 0 | 0 |
| MARCH7 | 1 | 0 | 0 | 0 | 0 | 0 |
| MARCH8 | 0 | 0 | 0 | 0 | 1 | 0 |
| MARCKS | 1 | 0 | 0 | 1 | 0 | 0 |
| MARCKSL1 | 1 | 0 | 1 | 1 | 0 | 0 |
| MARK3 | 0 | 0 | 1 | 0 | 0 | 0 |
| MARK4 | 0 | 0 | 0 | 0 | 1 | 0 |
| MARS | 0 | 1 | 0 | 1 | 0 | 0 |
| MARS2 | 0 | 0 | 0 | 0 | 1 | 0 |
| MAT2A | 1 | 0 | 1 | 1 | 0 | 0 |
| MAT2B | 1 | 0 | 0 | 1 | 0 | 0 |
| MATR3 | 1 | 0 | 1 | 1 | 0 | 0 |
| MAX | 1 | 0 | 0 | 1 | 1 | 0 |
| MAZ | 1 | 0 | 0 | 0 | 0 | 0 |
| MB21D1 | 0 | 0 | 1 | 0 | 0 | 0 |
| MB21D2 | 0 | 0 | 0 | 0 | 0 | 1 |
| MBD2 | 0 | 0 | 1 | 0 | 0 | 0 |
| MBD3 | 0 | 0 | 1 | 0 | 0 | 0 |
| MBLAC2 | 0 | 0 | 0 | 0 | 1 | 0 |
| MBNL1 | 1 | 1 | 1 | 1 | 0 | 0 |
| MBNL2 | 0 | 0 | 1 | 0 | 0 | 0 |
| MBOAT2 | 0 | 0 | 0 | 0 | 1 | 0 |
| MBOAT7 | 0 | 0 | 1 | 1 | 0 | 0 |
| MBP | 0 | 1 | 0 | 0 | 0 | 0 |
| MBTD1 | 0 | 0 | 0 | 1 | 0 | 0 |
| MC1R | 0 | 0 | 1 | 0 | 0 | 0 |
| MC5R | 0 | 0 | 0 | 0 | 1 | 0 |
| MCAM | 1 | 0 | 1 | 0 | 1 | 0 |
| MCAT | 0 | 0 | 0 | 0 | 1 | 0 |
| MCC | 0 | 1 | 0 | 0 | 0 | 0 |
| MCL1 | 1 | 1 | 1 | 0 | 0 | 0 |
| MCM2 | 1 | 0 | 1 | 0 | 0 | 0 |
| MCM3 | 1 | 0 | 1 | 0 | 0 | 0 |
| MCM5 | 0 | 0 | 1 | 0 | 0 | 0 |
| MCM7 | 1 | 0 | 0 | 0 | 0 | 0 |
| MCM9 | 0 | 0 | 0 | 0 | 1 | 0 |
| MCMBP | 0 | 0 | 0 | 0 | 1 | 0 |
| MCMDC2 | 1 | 0 | 0 | 0 | 0 | 0 |
| MCTP2 | 0 | 0 | 1 | 0 | 0 | 0 |
| MCTS1 | 0 | 0 | 0 | 0 | 1 | 0 |
| MDC1 | 0 | 0 | 0 | 1 | 0 | 0 |
| MDFIC | 0 | 0 | 0 | 1 | 0 | 0 |
| MDH1 | 1 | 0 | 0 | 0 | 1 | 0 |
| MDH2 | 1 | 0 | 0 | 0 | 0 | 0 |
| MDM2 | 1 | 0 | 0 | 0 | 0 | 0 |
| MDM4 | 0 | 0 | 0 | 1 | 0 | 0 |
| MEAF6 | 1 | 0 | 0 | 0 | 1 | 0 |
| MECOM | 1 | 0 | 0 | 0 | 0 | 0 |
| MECP2 | 0 | 0 | 0 | 0 | 1 | 0 |
| MED13 | 0 | 0 | 0 | 0 | 1 | 0 |
| MED13L | 0 | 0 | 0 | 1 | 0 | 0 |
| MED20 | 0 | 0 | 0 | 0 | 1 | 0 |
| MED25 | 0 | 0 | 1 | 0 | 0 | 0 |
| MED28 | 1 | 0 | 0 | 0 | 1 | 0 |
| MED29 | 1 | 0 | 1 | 0 | 1 | 0 |
| MED31 | 0 | 0 | 0 | 0 | 1 | 0 |
| MED4 | 0 | 0 | 1 | 1 | 1 | 0 |
| MED6 | 0 | 0 | 0 | 1 | 0 | 0 |
| MEGF9 | 0 | 0 | 0 | 0 | 1 | 0 |
| MELK | 1 | 0 | 0 | 0 | 0 | 0 |
| MEMO1 | 0 | 0 | 0 | 1 | 0 | 0 |
| MESDC2 | 1 | 0 | 0 | 0 | 1 | 0 |
| MET | 0 | 0 | 1 | 0 | 0 | 0 |
| METAP1 | 0 | 0 | 0 | 0 | 1 | 0 |
| METAP2 | 1 | 0 | 0 | 0 | 1 | 0 |
| Metazoa_SRP | 0 | 0 | 0 | 0 | 1 | 1 |
| METTL13 | 0 | 0 | 0 | 0 | 1 | 0 |
| METTL15 | 0 | 0 | 0 | 0 | 1 | 0 |
| METTL16 | 0 | 0 | 0 | 0 | 1 | 0 |
| METTL17 | 0 | 0 | 0 | 0 | 1 | 0 |
| METTL21A | 1 | 0 | 0 | 0 | 0 | 0 |
| METTL22 | 0 | 0 | 1 | 0 | 0 | 0 |
| METTL24 | 1 | 0 | 0 | 0 | 0 | 0 |
| METTL3 | 0 | 0 | 1 | 0 | 0 | 0 |
| METTL4 | 0 | 0 | 0 | 0 | 1 | 0 |
| METTL5 | 1 | 0 | 0 | 0 | 0 | 0 |
| METTL6 | 0 | 0 | 0 | 0 | 1 | 0 |
| METTL8 | 0 | 0 | 0 | 0 | 1 | 0 |
| METTL9 | 1 | 0 | 0 | 0 | 0 | 0 |
| MFAP3 | 0 | 0 | 0 | 0 | 1 | 0 |
| MFHAS1 | 0 | 0 | 0 | 1 | 0 | 0 |
| MFSD1 | 1 | 0 | 1 | 0 | 0 | 0 |
| MFSD12 | 0 | 0 | 1 | 0 | 0 | 0 |
| MFSD5 | 0 | 0 | 0 | 0 | 1 | 0 |
| MGA | 0 | 0 | 1 | 0 | 0 | 0 |
| MGAT2 | 0 | 0 | 0 | 1 | 0 | 0 |
| MGAT3 | 0 | 0 | 0 | 0 | 1 | 0 |
| MGAT5 | 1 | 0 | 0 | 0 | 0 | 0 |
| MGAT5B | 0 | 0 | 1 | 0 | 0 | 0 |
| MGC12982 | 0 | 1 | 0 | 0 | 0 | 0 |
| MGC2752 | 0 | 0 | 0 | 1 | 0 | 0 |
| MGC33846 | 0 | 1 | 0 | 0 | 0 | 0 |
| MGST2 | 0 | 0 | 0 | 0 | 1 | 0 |
| MIA | 1 | 0 | 0 | 0 | 0 | 0 |
| MIB1 | 1 | 0 | 1 | 1 | 0 | 0 |
| MIB2 | 1 | 1 | 0 | 0 | 0 | 0 |
| MICA | 0 | 0 | 0 | 0 | 1 | 0 |
| MICALL2 | 0 | 0 | 1 | 0 | 0 | 0 |
| MICB | 0 | 0 | 1 | 1 | 1 | 0 |
| MID1IP1 | 0 | 0 | 1 | 0 | 1 | 0 |
| MIDN | 1 | 0 | 1 | 0 | 0 | 0 |
| MIEF1 | 0 | 0 | 1 | 0 | 0 | 0 |
| MIER1 | 0 | 0 | 1 | 0 | 0 | 0 |
| MIER2 | 0 | 0 | 1 | 0 | 0 | 0 |
| MIF | 1 | 0 | 0 | 0 | 0 | 0 |
| MINA | 0 | 0 | 0 | 1 | 0 | 0 |
| MINOS1 | 1 | 0 | 0 | 0 | 0 | 0 |
| MIOS | 0 | 0 | 0 | 0 | 1 | 0 |
| MIPOL1 | 0 | 0 | 1 | 0 | 0 | 0 |
| MIR105-1 | 0 | 0 | 0 | 0 | 1 | 0 |
| MIR105-2 | 0 | 0 | 0 | 0 | 1 | 0 |
| MIR107 | 0 | 0 | 0 | 0 | 1 | 0 |
| MIR1180 | 0 | 0 | 0 | 0 | 1 | 0 |
| MIR122 | 0 | 0 | 0 | 0 | 0 | 1 |
| MIR1225 | 0 | 0 | 0 | 0 | 1 | 0 |
| MIR154 | 0 | 0 | 0 | 0 | 0 | 1 |
| MIR16-2 | 0 | 0 | 0 | 0 | 1 | 0 |
| MIR17HG | 0 | 0 | 1 | 0 | 0 | 0 |
| MIR188 | 0 | 0 | 0 | 0 | 1 | 0 |
| MIR199A2 | 0 | 0 | 0 | 0 | 1 | 0 |
| MIR19B2 | 0 | 0 | 0 | 0 | 1 | 0 |
| MIR21 | 0 | 0 | 0 | 1 | 1 | 0 |
| MIR26A1 | 0 | 0 | 0 | 0 | 1 | 0 |
| MIR26B | 0 | 0 | 0 | 0 | 1 | 0 |
| MIR29B1 | 0 | 0 | 1 | 0 | 0 | 0 |
| MIR29C | 0 | 0 | 0 | 0 | 0 | 1 |
| MIR31 | 0 | 0 | 1 | 0 | 0 | 0 |
| MIR3125 | 0 | 0 | 0 | 0 | 1 | 0 |
| MIR3146 | 0 | 0 | 0 | 0 | 1 | 0 |
| MIR3162 | 0 | 0 | 0 | 0 | 1 | 0 |
| MIR3164 | 0 | 0 | 0 | 0 | 1 | 0 |
| MIR31HG | 0 | 0 | 1 | 0 | 0 | 0 |
| MIR3609 | 0 | 0 | 1 | 0 | 0 | 0 |
| MIR362 | 0 | 0 | 0 | 0 | 1 | 0 |
| MIR3684 | 0 | 0 | 0 | 0 | 1 | 0 |
| MIR3691 | 0 | 0 | 0 | 0 | 1 | 0 |
| MIR3916 | 0 | 0 | 1 | 0 | 0 | 0 |
| MIR3926-2 | 0 | 0 | 0 | 0 | 1 | 0 |
| MIR3941 | 0 | 0 | 0 | 0 | 1 | 0 |
| MIR3944 | 0 | 0 | 0 | 0 | 1 | 0 |
| MIR3973 | 0 | 0 | 0 | 0 | 1 | 0 |
| MIR3978 | 0 | 0 | 0 | 0 | 1 | 0 |
| MIR4308 | 0 | 0 | 0 | 0 | 1 | 0 |
| MIR4427 | 0 | 0 | 0 | 0 | 1 | 0 |
| MIR4469 | 0 | 0 | 0 | 0 | 1 | 0 |
| MIR4500HG | 0 | 0 | 1 | 0 | 0 | 0 |
| MIR4525 | 0 | 0 | 0 | 0 | 1 | 0 |
| MIR4632 | 0 | 0 | 0 | 0 | 1 | 0 |
| MIR4633 | 0 | 0 | 0 | 0 | 1 | 0 |
| MIR4639 | 0 | 0 | 0 | 0 | 1 | 0 |
| MIR4658 | 0 | 0 | 0 | 0 | 1 | 0 |
| MIR4659B | 0 | 0 | 0 | 0 | 1 | 0 |
| MIR4668 | 0 | 0 | 0 | 0 | 1 | 0 |
| MIR4669 | 0 | 0 | 0 | 0 | 1 | 0 |
| MIR4687 | 0 | 0 | 0 | 0 | 1 | 0 |
| MIR4700 | 0 | 0 | 0 | 0 | 1 | 0 |
| MIR4709 | 0 | 0 | 1 | 0 | 1 | 0 |
| MIR4723 | 0 | 0 | 0 | 0 | 1 | 0 |
| MIR4729 | 0 | 0 | 0 | 0 | 0 | 1 |
| MIR4731 | 0 | 0 | 0 | 0 | 1 | 0 |
| MIR4762 | 0 | 0 | 0 | 0 | 1 | 0 |
| MIR4766 | 0 | 0 | 0 | 0 | 1 | 0 |
| MIR4771-1 | 0 | 0 | 0 | 0 | 1 | 0 |
| MIR4774 | 0 | 0 | 0 | 0 | 1 | 0 |
| MIR4782 | 0 | 0 | 0 | 0 | 1 | 0 |
| MIR4800 | 0 | 0 | 0 | 0 | 1 | 0 |
| MIR4999 | 0 | 0 | 0 | 0 | 1 | 0 |
| MIR5001 | 0 | 0 | 0 | 0 | 1 | 0 |
| MIR5009 | 0 | 0 | 0 | 0 | 1 | 0 |
| MIR500A | 0 | 0 | 0 | 0 | 1 | 0 |
| MIR500B | 0 | 0 | 0 | 0 | 1 | 0 |
| MIR501 | 0 | 0 | 0 | 0 | 1 | 0 |
| MIR502 | 0 | 0 | 0 | 0 | 1 | 0 |
| MIR5094 | 0 | 0 | 0 | 0 | 1 | 0 |
| MIR5194 | 0 | 0 | 0 | 0 | 1 | 0 |
| MIR532 | 0 | 0 | 0 | 0 | 1 | 0 |
| MIR544B | 0 | 0 | 0 | 0 | 1 | 0 |
| MIR548H3 | 0 | 0 | 0 | 0 | 1 | 0 |
| MIR548N | 0 | 0 | 0 | 0 | 1 | 0 |
| MIR548P | 0 | 0 | 0 | 0 | 1 | 0 |
| MIR548T | 0 | 0 | 0 | 0 | 1 | 0 |
| MIR555 | 0 | 0 | 0 | 0 | 1 | 0 |
| MIR5581 | 0 | 0 | 0 | 0 | 1 | 0 |
| MIR5584 | 0 | 0 | 0 | 0 | 1 | 0 |
| MIR5585 | 0 | 0 | 0 | 0 | 1 | 0 |
| MIR559 | 0 | 0 | 0 | 0 | 1 | 0 |
| MIR5687 | 0 | 0 | 0 | 0 | 0 | 1 |
| MIR5697 | 0 | 0 | 0 | 0 | 1 | 0 |
| MIR577 | 0 | 0 | 0 | 0 | 1 | 0 |
| MIR621 | 0 | 0 | 0 | 0 | 1 | 0 |
| MIR623 | 0 | 0 | 0 | 0 | 1 | 0 |
| MIR652 | 0 | 0 | 0 | 0 | 1 | 0 |
| MIR660 | 0 | 0 | 0 | 0 | 1 | 0 |
| MIR663A | 0 | 0 | 1 | 0 | 0 | 0 |
| MIR7-3 | 0 | 0 | 0 | 0 | 0 | 1 |
| MIR7-3HG | 0 | 0 | 0 | 0 | 0 | 1 |
| MIR744 | 0 | 0 | 0 | 0 | 1 | 0 |
| MIR767 | 0 | 0 | 0 | 0 | 1 | 0 |
| MIR92A2 | 0 | 0 | 0 | 0 | 1 | 0 |
| MIR941-1 | 0 | 0 | 0 | 0 | 1 | 0 |
| MIR941-2 | 0 | 0 | 0 | 0 | 1 | 0 |
| MIR941-4 | 0 | 0 | 0 | 0 | 1 | 0 |
| MIR99A | 0 | 0 | 0 | 0 | 0 | 1 |
| MIRHG2 | 0 | 0 | 0 | 1 | 0 | 0 |
| MIRLET7G | 0 | 0 | 0 | 0 | 1 | 0 |
| MIRLET7I | 0 | 0 | 0 | 0 | 1 | 0 |
| MIS12 | 0 | 0 | 0 | 1 | 0 | 0 |
| MKI67 | 0 | 0 | 1 | 0 | 0 | 0 |
| MKI67IP | 0 | 0 | 0 | 1 | 0 | 0 |
| MKL2 | 0 | 0 | 1 | 0 | 0 | 0 |
| MKLN1 | 0 | 0 | 1 | 0 | 0 | 0 |
| MKNK1 | 0 | 0 | 0 | 0 | 1 | 0 |
| MKRN1 | 1 | 0 | 0 | 0 | 0 | 0 |
| MKRN2 | 0 | 0 | 0 | 0 | 1 | 0 |
| MKRN3 | 0 | 0 | 0 | 0 | 1 | 0 |
| MKRN4P | 0 | 0 | 0 | 0 | 1 | 0 |
| MLEC | 1 | 0 | 0 | 0 | 1 | 0 |
| MLL5 | 0 | 0 | 0 | 1 | 0 | 0 |
| MLLT10 | 0 | 0 | 1 | 0 | 0 | 0 |
| MLLT11 | 0 | 0 | 0 | 0 | 1 | 0 |
| MLLT3 | 0 | 0 | 1 | 0 | 0 | 0 |
| MLLT6 | 1 | 0 | 0 | 0 | 0 | 0 |
| MLPH | 1 | 0 | 0 | 0 | 0 | 0 |
| MLXIP | 0 | 0 | 0 | 1 | 0 | 0 |
| MLXIPL | 0 | 0 | 1 | 0 | 0 | 0 |
| MMADHC | 1 | 0 | 0 | 0 | 0 | 0 |
| MMD | 0 | 0 | 0 | 1 | 1 | 0 |
| MME | 1 | 0 | 0 | 0 | 0 | 0 |
| MMGT1 | 1 | 0 | 1 | 0 | 1 | 0 |
| MMP14 | 1 | 0 | 0 | 0 | 0 | 0 |
| MMP15 | 0 | 0 | 1 | 0 | 0 | 0 |
| MMP16 | 0 | 1 | 0 | 0 | 0 | 0 |
| MMS22L | 1 | 0 | 0 | 0 | 1 | 0 |
| MNAT1 | 0 | 0 | 1 | 0 | 1 | 0 |
| MOB1A | 1 | 0 | 0 | 0 | 0 | 0 |
| MOB1B | 0 | 0 | 0 | 0 | 1 | 0 |
| MOB3B | 0 | 0 | 0 | 0 | 1 | 0 |
| MOB3C | 0 | 0 | 0 | 0 | 1 | 0 |
| MOB4 | 1 | 0 | 0 | 0 | 1 | 0 |
| MOCOS | 0 | 0 | 1 | 0 | 0 | 0 |
| MOCS2 | 0 | 0 | 0 | 1 | 0 | 0 |
| MOCS3 | 0 | 0 | 0 | 0 | 1 | 0 |
| MON1B | 0 | 0 | 0 | 0 | 1 | 0 |
| MORC3 | 0 | 0 | 0 | 1 | 0 | 0 |
| MORF4 | 0 | 0 | 0 | 0 | 1 | 0 |
| MORF4L1 | 1 | 0 | 0 | 0 | 0 | 0 |
| MORF4L2 | 1 | 0 | 1 | 1 | 0 | 0 |
| MORN1 | 0 | 0 | 0 | 0 | 1 | 0 |
| MOSPD1 | 0 | 0 | 0 | 1 | 1 | 0 |
| MPHOSPH8 | 0 | 0 | 0 | 0 | 1 | 0 |
| MPP6 | 0 | 0 | 1 | 0 | 0 | 0 |
| MPRIP | 1 | 0 | 0 | 0 | 0 | 0 |
| MPZL1 | 0 | 0 | 1 | 0 | 0 | 0 |
| MPZL3 | 0 | 0 | 0 | 1 | 1 | 0 |
| MR1 | 0 | 0 | 0 | 0 | 1 | 1 |
| MRAP2 | 0 | 0 | 0 | 0 | 1 | 0 |
| MRFAP1 | 1 | 0 | 0 | 0 | 1 | 0 |
| MRFAP1L1 | 1 | 0 | 1 | 1 | 0 | 0 |
| MRGPRX2 | 0 | 1 | 0 | 0 | 0 | 0 |
| M-RIP | 0 | 1 | 0 | 0 | 0 | 0 |
| MRPL10 | 0 | 0 | 0 | 0 | 1 | 0 |
| MRPL19 | 1 | 0 | 1 | 0 | 0 | 0 |
| MRPL3 | 1 | 0 | 0 | 1 | 0 | 0 |
| MRPL30 | 1 | 0 | 0 | 0 | 0 | 0 |
| MRPL35P1 | 0 | 0 | 0 | 0 | 1 | 0 |
| MRPL42 | 1 | 0 | 0 | 0 | 0 | 0 |
| MRPL51 | 1 | 0 | 0 | 0 | 1 | 0 |
| MRPL54 | 0 | 0 | 0 | 1 | 0 | 0 |
| MRPL9 | 0 | 0 | 0 | 0 | 1 | 0 |
| MRPS14 | 0 | 0 | 0 | 0 | 1 | 0 |
| MRPS18B | 0 | 0 | 0 | 0 | 1 | 0 |
| MRPS24 | 1 | 0 | 0 | 0 | 1 | 0 |
| MRPS27 | 0 | 0 | 0 | 1 | 0 | 0 |
| MRPS28 | 0 | 0 | 0 | 0 | 1 | 0 |
| MRPS33 | 0 | 0 | 0 | 1 | 0 | 0 |
| MRPS6 | 1 | 0 | 0 | 1 | 0 | 0 |
| MRRF | 0 | 0 | 1 | 0 | 0 | 0 |
| MSANTD2 | 0 | 0 | 1 | 0 | 0 | 1 |
| MSANTD3-TMEFF1 | 0 | 0 | 0 | 0 | 1 | 0 |
| MSH5 | 0 | 0 | 1 | 0 | 0 | 0 |
| MSH5-SAPCD1 | 0 | 0 | 1 | 0 | 0 | 0 |
| MSI2 | 1 | 1 | 1 | 0 | 0 | 0 |
| MSL1 | 0 | 0 | 0 | 0 | 1 | 0 |
| MSL2 | 1 | 0 | 0 | 1 | 1 | 0 |
| MSL3 | 0 | 0 | 0 | 0 | 1 | 0 |
| MSLN | 0 | 0 | 0 | 0 | 1 | 0 |
| MSMO1 | 0 | 0 | 1 | 0 | 0 | 0 |
| MSN | 1 | 0 | 1 | 1 | 0 | 0 |
| MSRA | 0 | 1 | 0 | 0 | 0 | 0 |
| MSRB1 | 0 | 0 | 0 | 0 | 1 | 0 |
| MSTO1 | 1 | 0 | 0 | 0 | 0 | 0 |
| MT1E | 0 | 1 | 0 | 0 | 1 | 0 |
| MT2A | 0 | 0 | 1 | 0 | 0 | 0 |
| MTA3 | 0 | 0 | 0 | 0 | 1 | 0 |
| MTAP | 1 | 0 | 1 | 0 | 0 | 0 |
| MTCH1 | 0 | 0 | 0 | 0 | 1 | 0 |
| MTCH2 | 1 | 0 | 0 | 0 | 0 | 0 |
| MTCYBP3 | 0 | 0 | 0 | 0 | 1 | 0 |
| MTDH | 1 | 0 | 0 | 0 | 0 | 0 |
| MTG1 | 0 | 0 | 1 | 0 | 0 | 0 |
| MTHFD1L | 0 | 0 | 1 | 0 | 0 | 0 |
| MTHFD2 | 1 | 0 | 0 | 1 | 0 | 0 |
| MTHFD2L | 0 | 0 | 1 | 0 | 0 | 0 |
| MTHFS | 0 | 0 | 0 | 0 | 1 | 0 |
| MTIF2 | 0 | 0 | 0 | 1 | 0 | 0 |
| MTL5 | 0 | 0 | 0 | 0 | 1 | 0 |
| MTMR12 | 0 | 0 | 0 | 1 | 0 | 0 |
| MTMR4 | 1 | 0 | 0 | 0 | 1 | 0 |
| MTMR6 | 1 | 0 | 0 | 0 | 1 | 0 |
| MTMR9 | 1 | 0 | 0 | 0 | 1 | 0 |
| MT-ND1 | 0 | 0 | 1 | 0 | 0 | 0 |
| MTND5P2 | 0 | 0 | 0 | 0 | 1 | 0 |
| MTPN | 1 | 0 | 0 | 0 | 0 | 0 |
| MTRF1 | 0 | 0 | 1 | 0 | 0 | 0 |
| MTRF1L | 0 | 0 | 0 | 1 | 0 | 0 |
| MT-RNR1 | 0 | 0 | 1 | 0 | 0 | 0 |
| MT-RNR2 | 0 | 0 | 1 | 0 | 0 | 0 |
| MTRNR2L1 | 1 | 0 | 0 | 0 | 0 | 0 |
| MTRNR2L10 | 1 | 0 | 0 | 0 | 0 | 0 |
| MTRNR2L2 | 1 | 0 | 0 | 0 | 0 | 0 |
| MTRNR2L8 | 1 | 0 | 0 | 0 | 0 | 0 |
| MT-TL1 | 0 | 0 | 1 | 0 | 0 | 0 |
| MT-TV | 0 | 0 | 1 | 0 | 0 | 0 |
| MTX2 | 0 | 0 | 0 | 0 | 1 | 0 |
| MUC4 | 0 | 1 | 0 | 0 | 0 | 0 |
| MUL1 | 0 | 0 | 0 | 0 | 1 | 0 |
| MUM1 | 1 | 0 | 0 | 0 | 1 | 0 |
| MUSTN1 | 0 | 0 | 0 | 0 | 1 | 0 |
| MXD4 | 0 | 0 | 1 | 0 | 1 | 0 |
| MXI1 | 0 | 0 | 1 | 1 | 0 | 0 |
| MXRA7 | 0 | 1 | 0 | 0 | 0 | 0 |
| MYADM | 0 | 0 | 1 | 0 | 0 | 0 |
| MYADML | 0 | 0 | 0 | 0 | 1 | 0 |
| MYB | 0 | 0 | 0 | 1 | 0 | 0 |
| MYCBP | 0 | 0 | 0 | 0 | 1 | 0 |
| MYD88 | 0 | 1 | 0 | 1 | 1 | 0 |
| MYEOV | 0 | 0 | 0 | 0 | 1 | 0 |
| MYEOV2 | 1 | 0 | 0 | 0 | 0 | 0 |
| MYL12A | 1 | 0 | 0 | 0 | 0 | 0 |
| MYL12B | 1 | 0 | 0 | 0 | 0 | 0 |
| MYL4 | 0 | 1 | 0 | 0 | 0 | 0 |
| MYL6 | 1 | 0 | 0 | 0 | 0 | 0 |
| MYL6P2 | 0 | 0 | 0 | 0 | 1 | 0 |
| MYLIP | 0 | 0 | 0 | 0 | 1 | 0 |
| MYLK | 1 | 0 | 0 | 0 | 0 | 0 |
| MYO18A | 0 | 0 | 1 | 0 | 0 | 0 |
| MYO1B | 1 | 0 | 0 | 0 | 0 | 0 |
| MYO1C | 0 | 0 | 1 | 0 | 0 | 0 |
| MYO1D | 1 | 0 | 0 | 0 | 0 | 0 |
| MYRIP | 0 | 0 | 0 | 0 | 0 | 1 |
| MYST3 | 0 | 0 | 0 | 1 | 0 | 0 |
| MZT2B | 1 | 0 | 0 | 0 | 0 | 0 |
| N4BP1 | 0 | 0 | 0 | 1 | 0 | 0 |
| N4BP2L1 | 0 | 0 | 0 | 1 | 0 | 0 |
| N4BP2L2 | 0 | 0 | 1 | 0 | 0 | 0 |
| N6AMT2 | 0 | 0 | 0 | 0 | 1 | 0 |
| NAA15 | 0 | 0 | 1 | 0 | 0 | 0 |
| NAA20 | 1 | 0 | 0 | 0 | 0 | 0 |
| NAA50 | 1 | 0 | 0 | 0 | 0 | 0 |
| NAAA | 0 | 0 | 0 | 0 | 1 | 0 |
| NAB1 | 1 | 0 | 0 | 0 | 0 | 0 |
| NACA | 1 | 0 | 0 | 0 | 0 | 0 |
| NACA2 | 1 | 0 | 0 | 0 | 0 | 0 |
| NACC1 | 1 | 0 | 1 | 0 | 0 | 0 |
| NACC2 | 1 | 0 | 1 | 0 | 1 | 0 |
| NADK2 | 0 | 0 | 1 | 0 | 0 | 0 |
| NADKD1 | 0 | 0 | 0 | 0 | 1 | 0 |
| NAGPA | 0 | 0 | 0 | 0 | 1 | 0 |
| NAMPT | 1 | 0 | 0 | 1 | 0 | 0 |
| NANP | 0 | 0 | 0 | 0 | 1 | 0 |
| NAP1L1 | 1 | 0 | 1 | 1 | 0 | 0 |
| NAP1L4 | 0 | 0 | 1 | 0 | 0 | 0 |
| NAP1L5 | 0 | 0 | 0 | 0 | 1 | 0 |
| NAPB | 1 | 0 | 0 | 0 | 1 | 0 |
| NAPEPLD | 1 | 1 | 0 | 0 | 0 | 0 |
| NAPG | 1 | 0 | 0 | 0 | 0 | 0 |
| NARS | 0 | 0 | 0 | 1 | 0 | 0 |
| NAT13 | 0 | 0 | 0 | 1 | 0 | 0 |
| NAT14 | 0 | 0 | 0 | 0 | 1 | 0 |
| NAT8L | 0 | 0 | 0 | 0 | 1 | 0 |
| NAV1 | 0 | 0 | 1 | 0 | 0 | 0 |
| NAV3 | 0 | 1 | 0 | 0 | 0 | 0 |
| NBEA | 0 | 1 | 0 | 0 | 0 | 0 |
| NBL1 | 1 | 0 | 0 | 0 | 0 | 0 |
| NBN | 1 | 0 | 0 | 1 | 1 | 0 |
| NBPF10 | 0 | 0 | 0 | 1 | 0 | 0 |
| NBPF16 | 1 | 0 | 0 | 0 | 0 | 0 |
| NCALD | 0 | 0 | 0 | 0 | 1 | 0 |
| NCAPD2 | 1 | 0 | 0 | 0 | 0 | 0 |
| NCAPG2 | 0 | 0 | 1 | 0 | 0 | 0 |
| NCBP1 | 0 | 0 | 1 | 0 | 0 | 0 |
| NCBP2 | 0 | 0 | 0 | 1 | 1 | 0 |
| NCEH1 | 0 | 0 | 0 | 0 | 1 | 0 |
| NCK1 | 0 | 0 | 1 | 0 | 1 | 0 |
| NCK2 | 0 | 0 | 0 | 1 | 0 | 0 |
| NCKAP1 | 1 | 0 | 0 | 0 | 0 | 0 |
| NCKAP1L | 0 | 0 | 0 | 0 | 1 | 0 |
| NCKAP5L | 0 | 0 | 0 | 0 | 1 | 0 |
| NCL | 1 | 0 | 0 | 1 | 0 | 0 |
| NCOA3 | 1 | 0 | 0 | 1 | 0 | 0 |
| NCR3LG1 | 0 | 0 | 0 | 0 | 1 | 0 |
| NCSTN | 1 | 0 | 0 | 0 | 1 | 0 |
| NDEL1 | 0 | 0 | 1 | 0 | 0 | 0 |
| NDFIP1 | 0 | 0 | 0 | 1 | 1 | 0 |
| NDFIP2 | 0 | 0 | 0 | 1 | 1 | 0 |
| NDN | 0 | 0 | 0 | 0 | 1 | 0 |
| NDNL2 | 1 | 0 | 0 | 0 | 1 | 0 |
| NDRG3 | 1 | 0 | 1 | 0 | 1 | 0 |
| NDUFA1 | 1 | 0 | 0 | 0 | 0 | 0 |
| NDUFA11 | 1 | 0 | 0 | 0 | 1 | 0 |
| NDUFA13 | 1 | 0 | 0 | 0 | 0 | 0 |
| NDUFA4 | 1 | 0 | 0 | 0 | 0 | 0 |
| NDUFA5 | 0 | 0 | 0 | 1 | 0 | 0 |
| NDUFA7 | 0 | 0 | 0 | 0 | 1 | 0 |
| NDUFA9 | 0 | 0 | 0 | 0 | 1 | 0 |
| NDUFAB1 | 0 | 0 | 0 | 0 | 1 | 0 |
| NDUFAF4 | 0 | 0 | 0 | 0 | 1 | 0 |
| NDUFB1 | 0 | 0 | 0 | 0 | 1 | 0 |
| NDUFB2 | 1 | 0 | 0 | 0 | 1 | 0 |
| NDUFB3 | 1 | 0 | 0 | 0 | 0 | 0 |
| NDUFB4 | 1 | 0 | 0 | 0 | 0 | 0 |
| NDUFB5 | 0 | 0 | 1 | 0 | 0 | 0 |
| NDUFB6 | 0 | 1 | 0 | 0 | 1 | 0 |
| NDUFB9 | 1 | 0 | 0 | 0 | 0 | 0 |
| NDUFC2 | 1 | 0 | 0 | 0 | 0 | 0 |
| NDUFC2-KCTD14 | 1 | 0 | 0 | 0 | 0 | 0 |
| NDUFS2 | 1 | 1 | 1 | 0 | 0 | 0 |
| NDUFS3 | 0 | 0 | 0 | 0 | 1 | 0 |
| NDUFS5 | 1 | 0 | 0 | 0 | 0 | 0 |
| NDUFV2 | 0 | 0 | 1 | 0 | 0 | 0 |
| NEAT1 | 0 | 0 | 1 | 0 | 0 | 0 |
| NECAP1 | 1 | 0 | 0 | 0 | 1 | 0 |
| NEDD8 | 1 | 0 | 1 | 0 | 0 | 0 |
| NEDD8-MDP1 | 1 | 0 | 1 | 0 | 0 | 0 |
| NEGR1 | 0 | 0 | 1 | 0 | 0 | 0 |
| NEK4 | 1 | 0 | 0 | 0 | 0 | 0 |
| NEK7 | 1 | 0 | 1 | 1 | 0 | 0 |
| NEK9 | 0 | 0 | 1 | 0 | 0 | 0 |
| NELFA | 0 | 0 | 1 | 0 | 0 | 0 |
| NELFB | 1 | 0 | 0 | 0 | 0 | 0 |
| NEU3 | 0 | 0 | 0 | 0 | 1 | 0 |
| NF1 | 0 | 0 | 1 | 0 | 0 | 0 |
| NF2 | 1 | 0 | 1 | 1 | 0 | 0 |
| NFASC | 0 | 1 | 0 | 0 | 0 | 0 |
| NFAT5 | 1 | 0 | 1 | 0 | 0 | 0 |
| NFIA | 0 | 0 | 1 | 0 | 0 | 0 |
| NFIB | 1 | 0 | 1 | 0 | 0 | 0 |
| NFIC | 0 | 0 | 1 | 0 | 0 | 0 |
| NFIL3 | 0 | 0 | 0 | 0 | 1 | 0 |
| NFKB1 | 0 | 0 | 1 | 0 | 0 | 0 |
| NFKBIZ | 0 | 0 | 0 | 1 | 0 | 0 |
| NFX1 | 0 | 0 | 1 | 0 | 1 | 0 |
| NFXL1 | 0 | 0 | 1 | 0 | 0 | 0 |
| NFYB | 0 | 0 | 0 | 0 | 1 | 0 |
| NFYC | 1 | 0 | 0 | 0 | 0 | 0 |
| NGFRAP1 | 1 | 0 | 1 | 0 | 0 | 0 |
| NGRN | 1 | 0 | 1 | 0 | 1 | 0 |
| NHLRC2 | 0 | 0 | 0 | 0 | 1 | 0 |
| NHLRC3 | 0 | 0 | 0 | 0 | 1 | 0 |
| NHP2L1 | 1 | 0 | 1 | 0 | 0 | 0 |
| NHS | 0 | 0 | 1 | 0 | 0 | 0 |
| NHSL2 | 1 | 0 | 0 | 0 | 0 | 0 |
| NIF3L1 | 0 | 0 | 0 | 0 | 1 | 0 |
| NINJ2 | 0 | 0 | 0 | 0 | 1 | 0 |
| NIPA2 | 0 | 0 | 0 | 0 | 1 | 0 |
| NIPAL3 | 0 | 0 | 0 | 0 | 1 | 0 |
| NIPBL | 0 | 0 | 1 | 0 | 0 | 0 |
| NIPSNAP3A | 0 | 0 | 0 | 0 | 1 | 0 |
| NKD2 | 1 | 0 | 0 | 0 | 0 | 0 |
| NKIRAS1 | 1 | 0 | 0 | 0 | 1 | 0 |
| NKTR | 0 | 0 | 1 | 0 | 0 | 0 |
| NLGN2 | 0 | 0 | 1 | 0 | 0 | 0 |
| NLGN4X | 0 | 1 | 0 | 0 | 0 | 0 |
| NLRP9 | 0 | 0 | 0 | 0 | 0 | 1 |
| NMD3 | 1 | 0 | 0 | 0 | 1 | 0 |
| NME1 | 1 | 0 | 0 | 0 | 0 | 0 |
| NME1-NME2 | 1 | 0 | 0 | 0 | 0 | 0 |
| NME2 | 1 | 0 | 0 | 0 | 0 | 0 |
| NME7 | 1 | 0 | 0 | 0 | 0 | 0 |
| NMNAT1 | 0 | 0 | 0 | 0 | 1 | 0 |
| NOC2L | 1 | 0 | 0 | 0 | 0 | 0 |
| NOL7 | 0 | 0 | 0 | 1 | 0 | 0 |
| NOL9 | 1 | 0 | 0 | 0 | 0 | 0 |
| NOLC1 | 1 | 0 | 1 | 0 | 0 | 0 |
| NONO | 1 | 0 | 0 | 0 | 0 | 0 |
| NOP56 | 1 | 0 | 0 | 0 | 0 | 0 |
| NOP58 | 1 | 0 | 0 | 1 | 0 | 0 |
| NOTCH2 | 1 | 0 | 1 | 1 | 0 | 0 |
| NOTCH2NL | 1 | 0 | 1 | 0 | 0 | 0 |
| NOVA1 | 0 | 1 | 0 | 0 | 0 | 0 |
| NPAS2 | 1 | 0 | 0 | 0 | 0 | 0 |
| NPC2 | 1 | 0 | 1 | 0 | 1 | 0 |
| NPEPPS | 0 | 0 | 1 | 0 | 0 | 0 |
| NPHP3 | 0 | 0 | 0 | 1 | 0 | 0 |
| NPLOC4 | 0 | 0 | 1 | 0 | 0 | 0 |
| NPM1 | 1 | 0 | 0 | 1 | 0 | 0 |
| NPNT | 0 | 1 | 0 | 0 | 0 | 0 |
| NPRL3 | 0 | 0 | 1 | 0 | 1 | 0 |
| NPTN | 1 | 0 | 0 | 0 | 0 | 0 |
| NPTX1 | 1 | 0 | 1 | 0 | 0 | 0 |
| NQO1 | 1 | 0 | 0 | 0 | 0 | 0 |
| NR1D2 | 0 | 0 | 0 | 1 | 0 | 0 |
| NR2F6 | 0 | 0 | 1 | 0 | 0 | 0 |
| NR3C1 | 1 | 0 | 0 | 1 | 1 | 0 |
| NR4A2 | 0 | 0 | 0 | 0 | 1 | 0 |
| NR5A2 | 0 | 0 | 0 | 0 | 1 | 0 |
| NR6A1 | 0 | 0 | 0 | 0 | 1 | 0 |
| NRARP | 0 | 0 | 0 | 0 | 1 | 0 |
| NRAS | 1 | 0 | 0 | 0 | 0 | 0 |
| NREP | 1 | 0 | 0 | 0 | 0 | 0 |
| NRM | 0 | 0 | 0 | 0 | 1 | 0 |
| NRXN2 | 0 | 0 | 0 | 0 | 1 | 0 |
| NSD1 | 0 | 1 | 0 | 0 | 0 | 0 |
| NSF | 1 | 0 | 0 | 0 | 0 | 0 |
| NSMCE1 | 0 | 0 | 0 | 1 | 0 | 0 |
| NSRP1 | 0 | 0 | 1 | 0 | 0 | 0 |
| NSRP1P1 | 0 | 0 | 0 | 0 | 1 | 0 |
| NSUN3 | 0 | 0 | 0 | 0 | 1 | 0 |
| NSUN4 | 0 | 0 | 0 | 0 | 1 | 0 |
| NT5C3 | 0 | 0 | 0 | 1 | 0 | 1 |
| NT5DC3 | 1 | 0 | 0 | 0 | 0 | 0 |
| NTPCR | 0 | 0 | 0 | 0 | 1 | 0 |
| NUBP2 | 0 | 0 | 0 | 0 | 1 | 0 |
| NUBPL | 0 | 0 | 0 | 0 | 1 | 0 |
| NUCKS1 | 1 | 0 | 1 | 0 | 0 | 0 |
| NUDC | 1 | 0 | 0 | 0 | 0 | 0 |
| NUDCD3 | 1 | 0 | 1 | 0 | 1 | 0 |
| NUDT15 | 0 | 0 | 0 | 0 | 1 | 0 |
| NUDT16L1 | 0 | 0 | 0 | 0 | 1 | 0 |
| NUDT19 | 1 | 0 | 0 | 0 | 0 | 0 |
| NUDT2 | 0 | 0 | 1 | 0 | 0 | 0 |
| NUDT21 | 1 | 0 | 0 | 1 | 0 | 0 |
| NUDT3 | 1 | 0 | 0 | 0 | 0 | 0 |
| NUDT4 | 0 | 0 | 0 | 1 | 0 | 0 |
| NUFIP1 | 0 | 0 | 0 | 1 | 0 | 0 |
| NUFIP2 | 1 | 0 | 1 | 0 | 0 | 0 |
| NUGGC | 0 | 0 | 0 | 0 | 1 | 0 |
| NUMA1 | 0 | 1 | 0 | 0 | 0 | 0 |
| NUP153 | 1 | 0 | 1 | 1 | 0 | 0 |
| NUP155 | 0 | 0 | 0 | 0 | 1 | 0 |
| NUP160 | 0 | 0 | 0 | 0 | 0 | 1 |
| NUP205 | 0 | 0 | 1 | 0 | 0 | 0 |
| NUP210 | 0 | 0 | 1 | 0 | 0 | 0 |
| NUP214 | 0 | 0 | 1 | 0 | 0 | 0 |
| NUP35 | 0 | 0 | 0 | 1 | 0 | 0 |
| NUP43 | 0 | 0 | 0 | 0 | 1 | 0 |
| NUP62 | 1 | 0 | 1 | 1 | 1 | 0 |
| NUP85 | 0 | 0 | 1 | 0 | 0 | 0 |
| NUP93 | 1 | 0 | 0 | 0 | 0 | 0 |
| NUP98 | 0 | 1 | 0 | 0 | 0 | 0 |
| NUPL1 | 1 | 0 | 0 | 0 | 0 | 0 |
| NUPL2 | 0 | 0 | 1 | 0 | 0 | 0 |
| NUS1 | 0 | 0 | 1 | 0 | 0 | 0 |
| NUTM2A-AS1 | 0 | 0 | 1 | 0 | 0 | 0 |
| NXPE3 | 1 | 0 | 0 | 0 | 0 | 0 |
| NXT1 | 0 | 0 | 0 | 0 | 1 | 0 |
| OARD1 | 0 | 0 | 0 | 0 | 1 | 0 |
| OAZ1 | 1 | 0 | 0 | 1 | 0 | 0 |
| OAZ2 | 1 | 0 | 0 | 0 | 1 | 0 |
| OBSCN | 0 | 0 | 1 | 0 | 0 | 0 |
| OCR1 | 0 | 1 | 0 | 0 | 0 | 0 |
| ODC1 | 1 | 0 | 0 | 0 | 0 | 0 |
| ODF2L | 1 | 0 | 0 | 0 | 0 | 0 |
| OGDH | 0 | 0 | 1 | 0 | 0 | 0 |
| OGFOD1 | 0 | 0 | 0 | 1 | 0 | 0 |
| OGFOD3 | 0 | 0 | 1 | 0 | 0 | 0 |
| OGT | 0 | 0 | 1 | 0 | 0 | 0 |
| OIP5 | 0 | 0 | 0 | 0 | 1 | 0 |
| OIP5-AS1 | 0 | 0 | 1 | 0 | 0 | 0 |
| OIT3 | 0 | 1 | 0 | 0 | 0 | 0 |
| ONECUT1 | 0 | 1 | 0 | 0 | 0 | 0 |
| OPA1 | 1 | 0 | 0 | 0 | 0 | 0 |
| OPHN1 | 1 | 0 | 0 | 0 | 0 | 0 |
| OPN3 | 0 | 0 | 1 | 1 | 1 | 0 |
| OPTN | 0 | 0 | 0 | 1 | 0 | 0 |
| OR10D1P | 0 | 1 | 0 | 0 | 0 | 0 |
| OR1C1 | 0 | 1 | 0 | 0 | 0 | 0 |
| OR1E2 | 0 | 0 | 0 | 0 | 1 | 0 |
| OR2AI1P | 0 | 0 | 0 | 0 | 1 | 0 |
| OR2T8 | 0 | 0 | 0 | 0 | 1 | 0 |
| OR2W3 | 1 | 0 | 0 | 0 | 1 | 0 |
| OR51B4 | 0 | 0 | 0 | 0 | 1 | 0 |
| OR52E6 | 0 | 0 | 0 | 0 | 1 | 0 |
| OR52E8 | 0 | 0 | 0 | 0 | 1 | 0 |
| OR52N1 | 0 | 0 | 0 | 0 | 1 | 0 |
| OR52N5 | 0 | 0 | 0 | 0 | 1 | 0 |
| OR5B3 | 0 | 0 | 0 | 0 | 1 | 0 |
| OR8G1 | 0 | 0 | 0 | 0 | 1 | 0 |
| OR8G3P | 0 | 0 | 0 | 0 | 1 | 0 |
| OR8G7P | 0 | 0 | 0 | 0 | 1 | 0 |
| ORAI1 | 0 | 0 | 0 | 0 | 1 | 0 |
| ORAOV1 | 1 | 0 | 0 | 0 | 0 | 0 |
| ORC5 | 0 | 0 | 0 | 0 | 1 | 0 |
| ORMDL1 | 0 | 0 | 0 | 1 | 0 | 0 |
| OSBP | 1 | 0 | 0 | 0 | 1 | 0 |
| OSBP2 | 0 | 0 | 1 | 0 | 0 | 0 |
| OSBPL10 | 1 | 0 | 1 | 0 | 0 | 0 |
| OSBPL11 | 0 | 0 | 0 | 0 | 1 | 0 |
| OSBPL3 | 1 | 0 | 1 | 0 | 0 | 0 |
| OSBPL8 | 1 | 0 | 0 | 1 | 0 | 0 |
| OSGEP | 0 | 0 | 0 | 0 | 1 | 0 |
| OSMR | 0 | 0 | 1 | 0 | 0 | 0 |
| OST4 | 1 | 0 | 0 | 0 | 0 | 0 |
| OSTC | 1 | 0 | 0 | 1 | 0 | 0 |
| OSTCP1 | 0 | 0 | 0 | 0 | 1 | 0 |
| OTOGL | 0 | 0 | 0 | 0 | 0 | 1 |
| OTUD4 | 1 | 0 | 0 | 1 | 0 | 0 |
| OTUD6B | 1 | 0 | 0 | 0 | 0 | 0 |
| OTUD7A | 1 | 0 | 0 | 0 | 0 | 0 |
| OVOL1 | 0 | 1 | 0 | 0 | 0 | 0 |
| OXSM | 0 | 0 | 0 | 0 | 1 | 0 |
| P2RY1 | 0 | 0 | 0 | 0 | 1 | 0 |
| P2RY11 | 1 | 0 | 0 | 0 | 0 | 0 |
| P4HA2 | 0 | 0 | 0 | 0 | 1 | 0 |
| P4HB | 1 | 0 | 1 | 0 | 0 | 0 |
| PABPC1 | 1 | 0 | 0 | 0 | 0 | 0 |
| PABPC1L | 1 | 0 | 0 | 0 | 0 | 0 |
| PABPC3 | 1 | 0 | 0 | 0 | 0 | 0 |
| PABPN1 | 1 | 0 | 1 | 1 | 0 | 0 |
| PACS1 | 0 | 0 | 1 | 0 | 0 | 0 |
| PACS2 | 1 | 0 | 0 | 0 | 0 | 0 |
| PADI1 | 0 | 1 | 0 | 0 | 0 | 0 |
| PAFAH1B1 | 1 | 0 | 1 | 0 | 0 | 1 |
| PAFAH1B2 | 1 | 0 | 0 | 1 | 0 | 0 |
| PAGE5 | 0 | 1 | 0 | 0 | 0 | 0 |
| PAGR1 | 1 | 0 | 0 | 0 | 1 | 0 |
| PAICS | 0 | 0 | 1 | 0 | 0 | 0 |
| PAIP2 | 0 | 0 | 0 | 1 | 0 | 0 |
| PAK2 | 1 | 0 | 0 | 1 | 0 | 0 |
| PAK3 | 0 | 1 | 0 | 0 | 0 | 0 |
| PAK4 | 0 | 0 | 0 | 0 | 1 | 0 |
| PALLD | 0 | 0 | 1 | 0 | 0 | 0 |
| PALM2-AKAP2 | 1 | 0 | 0 | 0 | 0 | 0 |
| PAM16 | 0 | 0 | 1 | 0 | 0 | 0 |
| PAN3 | 0 | 0 | 1 | 0 | 0 | 0 |
| PANK1 | 0 | 0 | 0 | 0 | 1 | 0 |
| PANK2 | 0 | 0 | 0 | 0 | 1 | 0 |
| PANK3 | 1 | 0 | 1 | 0 | 0 | 0 |
| PANK4 | 1 | 0 | 0 | 0 | 0 | 0 |
| PAPD5 | 1 | 0 | 0 | 1 | 0 | 0 |
| PAPOLA | 1 | 0 | 0 | 1 | 0 | 0 |
| PAPOLG | 1 | 0 | 0 | 1 | 1 | 0 |
| PAPSS1 | 0 | 0 | 0 | 0 | 1 | 0 |
| PAQR3 | 0 | 0 | 0 | 0 | 1 | 0 |
| PARD3 | 0 | 0 | 1 | 0 | 0 | 0 |
| PARK7 | 1 | 0 | 0 | 1 | 0 | 0 |
| PARL | 0 | 0 | 0 | 0 | 1 | 0 |
| PARN | 1 | 0 | 1 | 0 | 0 | 0 |
| PARP1 | 1 | 0 | 1 | 0 | 0 | 0 |
| PARP12 | 0 | 0 | 0 | 1 | 0 | 0 |
| PARP8 | 0 | 0 | 0 | 1 | 0 | 0 |
| PATL1 | 0 | 0 | 0 | 1 | 0 | 0 |
| PBK | 0 | 0 | 0 | 0 | 1 | 0 |
| PBRM1 | 0 | 0 | 0 | 1 | 0 | 0 |
| PBX1 | 0 | 0 | 1 | 0 | 0 | 0 |
| PBX3 | 0 | 0 | 1 | 0 | 0 | 0 |
| PCBP1 | 1 | 0 | 1 | 1 | 0 | 0 |
| PCBP2 | 1 | 0 | 1 | 0 | 0 | 0 |
| PCBP4 | 0 | 0 | 0 | 0 | 1 | 0 |
| PCCB | 0 | 0 | 0 | 0 | 1 | 0 |
| PCDH17 | 0 | 0 | 1 | 0 | 0 | 0 |
| PCDH7 | 0 | 0 | 1 | 0 | 0 | 0 |
| PCDHGB8P | 0 | 1 | 0 | 0 | 0 | 0 |
| PCDHGC5 | 0 | 1 | 0 | 0 | 0 | 0 |
| PCED1A | 0 | 0 | 1 | 0 | 0 | 0 |
| PCGF3 | 1 | 0 | 0 | 0 | 0 | 0 |
| PCGF5 | 0 | 0 | 0 | 1 | 1 | 0 |
| PCGF6 | 0 | 0 | 0 | 0 | 1 | 0 |
| PCGF7P | 0 | 0 | 0 | 0 | 1 | 0 |
| PCID2 | 0 | 0 | 1 | 0 | 0 | 0 |
| PCK1 | 0 | 0 | 0 | 0 | 1 | 0 |
| PCMTD2 | 0 | 0 | 0 | 1 | 0 | 0 |
| PCNA | 1 | 0 | 0 | 0 | 0 | 0 |
| PCNP | 1 | 0 | 1 | 0 | 0 | 0 |
| PCNX | 0 | 0 | 1 | 0 | 1 | 0 |
| PCNXL3 | 0 | 0 | 0 | 0 | 1 | 0 |
| PCNXL4 | 0 | 0 | 0 | 0 | 1 | 0 |
| PCYT1A | 1 | 0 | 0 | 0 | 0 | 0 |
| PDAP1 | 1 | 0 | 0 | 0 | 0 | 0 |
| PDCD10 | 1 | 0 | 0 | 0 | 0 | 0 |
| PDCD4 | 0 | 0 | 1 | 0 | 0 | 0 |
| PDCD5 | 1 | 0 | 0 | 0 | 0 | 0 |
| PDCD6 | 1 | 1 | 0 | 0 | 0 | 0 |
| PDCL | 0 | 0 | 0 | 0 | 1 | 0 |
| PDE10A | 0 | 0 | 1 | 0 | 0 | 0 |
| PDE12 | 1 | 0 | 0 | 0 | 1 | 0 |
| PDE1A | 0 | 0 | 1 | 0 | 0 | 0 |
| PDE3A | 1 | 0 | 1 | 0 | 0 | 0 |
| PDE3B | 0 | 0 | 0 | 1 | 0 | 0 |
| PDE4B | 0 | 0 | 1 | 1 | 0 | 0 |
| PDE4D | 0 | 0 | 1 | 0 | 0 | 0 |
| PDE7A | 0 | 0 | 0 | 1 | 1 | 0 |
| PDGFA | 1 | 0 | 1 | 0 | 1 | 0 |
| PDIA3 | 1 | 0 | 0 | 0 | 0 | 0 |
| PDIA5 | 0 | 0 | 1 | 0 | 0 | 0 |
| PDIA6 | 1 | 0 | 0 | 1 | 0 | 0 |
| PDIK1L | 0 | 0 | 0 | 0 | 1 | 0 |
| PDLIM5 | 1 | 0 | 1 | 0 | 0 | 0 |
| PDPN | 0 | 0 | 0 | 0 | 1 | 0 |
| PDS5A | 1 | 0 | 1 | 1 | 0 | 0 |
| PDS5B | 0 | 0 | 1 | 0 | 0 | 0 |
| PDSS2 | 0 | 0 | 0 | 0 | 1 | 0 |
| PDXDC2P | 0 | 0 | 1 | 0 | 0 | 0 |
| PDXK | 0 | 0 | 0 | 0 | 1 | 0 |
| PDXP | 0 | 0 | 0 | 0 | 1 | 0 |
| PDZD8 | 1 | 0 | 0 | 0 | 0 | 0 |
| PDZRN4 | 0 | 0 | 0 | 0 | 1 | 0 |
| PEA15 | 1 | 0 | 0 | 0 | 1 | 0 |
| PEAR1 | 0 | 0 | 0 | 0 | 1 | 0 |
| PEBP1 | 1 | 0 | 0 | 0 | 1 | 0 |
| PEG10 | 1 | 0 | 0 | 0 | 0 | 0 |
| PENK | 0 | 1 | 0 | 0 | 0 | 0 |
| PER3 | 0 | 0 | 0 | 0 | 1 | 0 |
| PERP | 1 | 0 | 1 | 0 | 1 | 0 |
| PET117 | 0 | 0 | 0 | 0 | 1 | 0 |
| PEX1 | 1 | 0 | 0 | 0 | 0 | 0 |
| PEX11B | 1 | 0 | 1 | 1 | 1 | 0 |
| PEX12 | 0 | 0 | 0 | 0 | 1 | 0 |
| PEX19 | 0 | 0 | 0 | 0 | 1 | 0 |
| PEX2 | 0 | 0 | 0 | 0 | 1 | 0 |
| PEX26 | 1 | 0 | 1 | 0 | 1 | 0 |
| PEX5 | 0 | 0 | 0 | 0 | 1 | 0 |
| PEX7 | 0 | 0 | 0 | 0 | 1 | 0 |
| PFDN2 | 1 | 0 | 0 | 1 | 0 | 0 |
| PFDN5 | 1 | 0 | 0 | 0 | 0 | 0 |
| PFKFB2 | 0 | 0 | 1 | 0 | 1 | 0 |
| PFKFB3 | 1 | 0 | 0 | 0 | 0 | 0 |
| PFKM | 0 | 0 | 0 | 0 | 1 | 0 |
| PFN1 | 1 | 0 | 0 | 0 | 0 | 0 |
| PFN2 | 1 | 0 | 0 | 0 | 0 | 1 |
| PGAM1 | 1 | 0 | 0 | 1 | 0 | 0 |
| PGAM1P10 | 0 | 0 | 0 | 0 | 1 | 0 |
| PGAM1P5 | 0 | 0 | 0 | 0 | 1 | 0 |
| PGAM4 | 1 | 0 | 0 | 0 | 0 | 0 |
| PGAP2 | 0 | 0 | 0 | 0 | 1 | 0 |
| PGGT1B | 0 | 0 | 0 | 0 | 1 | 0 |
| PGK1 | 1 | 0 | 0 | 1 | 0 | 0 |
| PGM2 | 1 | 0 | 0 | 0 | 0 | 0 |
| PGPEP1 | 0 | 0 | 0 | 0 | 1 | 0 |
| PGR | 0 | 0 | 0 | 0 | 1 | 0 |
| PGRMC1 | 1 | 0 | 0 | 0 | 0 | 0 |
| PHACTR2 | 0 | 0 | 0 | 1 | 0 | 0 |
| PHACTR4 | 1 | 0 | 0 | 0 | 0 | 0 |
| PHB | 1 | 0 | 0 | 0 | 0 | 0 |
| PHB2 | 1 | 0 | 0 | 1 | 0 | 0 |
| PHC1 | 1 | 0 | 0 | 0 | 0 | 0 |
| PHC3 | 1 | 0 | 0 | 0 | 0 | 0 |
| PHF11 | 0 | 0 | 1 | 0 | 0 | 0 |
| PHF14 | 0 | 0 | 1 | 0 | 0 | 0 |
| PHF16 | 0 | 0 | 0 | 0 | 1 | 0 |
| PHF17 | 0 | 0 | 0 | 1 | 1 | 0 |
| PHF2 | 0 | 0 | 0 | 1 | 0 | 0 |
| PHF20 | 0 | 0 | 1 | 0 | 0 | 0 |
| PHF21A | 0 | 0 | 1 | 0 | 0 | 0 |
| PHF3 | 0 | 0 | 1 | 0 | 0 | 0 |
| PHF5A | 0 | 0 | 0 | 1 | 0 | 0 |
| PHF6 | 1 | 0 | 0 | 1 | 0 | 0 |
| PHF8 | 1 | 0 | 0 | 1 | 0 | 0 |
| PHKB | 1 | 0 | 0 | 0 | 0 | 0 |
| PHLDA1 | 1 | 0 | 0 | 0 | 1 | 0 |
| PHLDA2 | 0 | 0 | 0 | 0 | 1 | 0 |
| PHLDB2 | 0 | 0 | 1 | 0 | 0 | 0 |
| PHLPP1 | 0 | 0 | 1 | 0 | 1 | 0 |
| PHOSPHO2 | 0 | 0 | 0 | 0 | 1 | 0 |
| PHOX2B | 0 | 0 | 0 | 0 | 1 | 0 |
| PHTF2 | 1 | 0 | 0 | 1 | 1 | 0 |
| PHYKPL | 0 | 0 | 1 | 0 | 0 | 0 |
| PIAS1 | 0 | 0 | 1 | 1 | 0 | 0 |
| PIBF1 | 0 | 0 | 1 | 0 | 0 | 0 |
| PID1 | 1 | 0 | 1 | 0 | 0 | 0 |
| PIEZO2 | 0 | 0 | 1 | 0 | 0 | 0 |
| PIGA | 0 | 0 | 0 | 0 | 1 | 0 |
| PIGB | 0 | 0 | 0 | 0 | 1 | 0 |
| PIGC | 0 | 0 | 0 | 0 | 1 | 0 |
| PIGG | 0 | 1 | 0 | 0 | 0 | 1 |
| PIGH | 0 | 0 | 0 | 0 | 1 | 0 |
| PIGK | 0 | 0 | 0 | 0 | 1 | 0 |
| PIGM | 0 | 0 | 0 | 0 | 1 | 0 |
| PIGN | 0 | 0 | 0 | 0 | 1 | 0 |
| PIGS | 1 | 0 | 1 | 0 | 1 | 0 |
| PIGU | 0 | 0 | 0 | 0 | 1 | 0 |
| PIGV | 0 | 0 | 0 | 0 | 1 | 0 |
| PIGW | 0 | 0 | 0 | 0 | 1 | 0 |
| PIGX | 0 | 0 | 0 | 0 | 1 | 0 |
| PIGY | 1 | 0 | 0 | 1 | 1 | 0 |
| PIH1D2 | 0 | 1 | 0 | 0 | 0 | 0 |
| PIK3C2B | 0 | 0 | 1 | 0 | 0 | 0 |
| PIK3R1 | 1 | 0 | 0 | 0 | 1 | 0 |
| PIKFYVE | 1 | 0 | 0 | 0 | 0 | 0 |
| PIM3 | 0 | 0 | 0 | 1 | 1 | 0 |
| PIN1 | 0 | 0 | 0 | 1 | 0 | 0 |
| PIP4K2A | 0 | 0 | 0 | 1 | 1 | 0 |
| PIP4K2B | 1 | 0 | 1 | 0 | 1 | 0 |
| PIP4K2C | 0 | 0 | 0 | 0 | 1 | 0 |
| PIP5K1A | 0 | 0 | 1 | 0 | 0 | 0 |
| PIP5K1C | 1 | 0 | 0 | 0 | 0 | 0 |
| PITHD1 | 1 | 0 | 0 | 0 | 1 | 0 |
| PITPNA | 0 | 0 | 1 | 0 | 0 | 0 |
| PITPNB | 0 | 0 | 1 | 0 | 0 | 0 |
| PITPNC1 | 0 | 0 | 1 | 0 | 0 | 0 |
| PJA2 | 1 | 0 | 0 | 1 | 0 | 0 |
| PKD1 | 0 | 0 | 0 | 0 | 1 | 0 |
| PKIA | 0 | 0 | 0 | 1 | 0 | 0 |
| PKM | 1 | 0 | 1 | 0 | 0 | 0 |
| PKN1 | 0 | 0 | 1 | 0 | 0 | 0 |
| PKN2 | 0 | 0 | 1 | 0 | 0 | 0 |
| PL-5283 | 0 | 0 | 0 | 1 | 0 | 0 |
| PLAGL1 | 0 | 0 | 0 | 0 | 1 | 0 |
| PLAGL2 | 0 | 0 | 0 | 0 | 1 | 0 |
| PLAU | 0 | 0 | 0 | 0 | 1 | 0 |
| PLCL1 | 0 | 0 | 0 | 0 | 1 | 0 |
| PLCL2 | 0 | 0 | 1 | 0 | 0 | 0 |
| PLD3 | 0 | 0 | 1 | 0 | 0 | 0 |
| PLD6 | 0 | 0 | 0 | 0 | 1 | 0 |
| PLEC | 0 | 0 | 1 | 0 | 0 | 0 |
| PLEC1 | 0 | 1 | 0 | 0 | 0 | 0 |
| PLEKHA1 | 0 | 0 | 0 | 0 | 1 | 0 |
| PLEKHA2 | 1 | 0 | 0 | 0 | 0 | 0 |
| PLEKHA3 | 1 | 0 | 0 | 0 | 0 | 0 |
| PLEKHA5 | 1 | 0 | 0 | 0 | 0 | 0 |
| PLEKHB1 | 0 | 0 | 0 | 1 | 0 | 0 |
| PLEKHB2 | 1 | 0 | 0 | 0 | 0 | 0 |
| PLEKHF2 | 0 | 0 | 0 | 0 | 1 | 0 |
| PLIN2 | 0 | 0 | 0 | 0 | 1 | 0 |
| PLOD2 | 0 | 0 | 1 | 0 | 0 | 0 |
| PLS3 | 1 | 0 | 0 | 0 | 0 | 0 |
| PLSCR1 | 0 | 0 | 0 | 0 | 1 | 0 |
| PLSCR2 | 0 | 0 | 0 | 0 | 1 | 0 |
| PLSCR3 | 0 | 0 | 0 | 0 | 1 | 0 |
| PLXDC1 | 1 | 0 | 0 | 0 | 0 | 0 |
| PLXNA2 | 0 | 1 | 0 | 0 | 0 | 0 |
| PLXNB2 | 0 | 0 | 1 | 0 | 0 | 0 |
| PM20D2 | 0 | 0 | 0 | 0 | 1 | 0 |
| PMAIP1 | 0 | 0 | 0 | 1 | 1 | 0 |
| PMEL | 1 | 0 | 0 | 0 | 0 | 0 |
| PMEPA1 | 1 | 0 | 0 | 0 | 0 | 0 |
| PMF1 | 1 | 0 | 0 | 0 | 0 | 0 |
| PMF1-BGLAP | 1 | 0 | 0 | 0 | 0 | 0 |
| PMP22 | 1 | 0 | 1 | 0 | 1 | 0 |
| PMS2L1 | 0 | 0 | 0 | 1 | 0 | 0 |
| PNKD | 0 | 0 | 0 | 0 | 1 | 0 |
| PNMA1 | 0 | 0 | 0 | 0 | 1 | 0 |
| PNMAL1 | 0 | 0 | 0 | 0 | 1 | 0 |
| PNN | 1 | 0 | 1 | 0 | 0 | 0 |
| PNPLA3 | 0 | 0 | 1 | 0 | 0 | 0 |
| PNPLA8 | 0 | 0 | 0 | 1 | 0 | 0 |
| PNPO | 1 | 0 | 0 | 0 | 1 | 0 |
| PODXL | 0 | 0 | 1 | 0 | 0 | 0 |
| POGZ | 0 | 0 | 1 | 0 | 0 | 0 |
| POLA1 | 0 | 0 | 1 | 0 | 1 | 0 |
| POLDIP3 | 1 | 0 | 1 | 0 | 1 | 0 |
| POLE2 | 0 | 0 | 0 | 0 | 1 | 0 |
| POLE3 | 1 | 0 | 0 | 1 | 1 | 0 |
| POLQ | 0 | 0 | 1 | 0 | 0 | 0 |
| POLR1A | 0 | 0 | 1 | 0 | 0 | 0 |
| POLR1B | 0 | 0 | 0 | 0 | 1 | 0 |
| POLR2A | 0 | 0 | 1 | 0 | 0 | 0 |
| POLR2B | 0 | 0 | 0 | 1 | 0 | 0 |
| POLR2F | 1 | 0 | 0 | 0 | 0 | 0 |
| POLR2G | 0 | 0 | 0 | 0 | 1 | 0 |
| POLR2H | 0 | 0 | 0 | 0 | 1 | 0 |
| POLR2K | 0 | 0 | 0 | 1 | 0 | 0 |
| POLR2M | 1 | 0 | 0 | 0 | 0 | 0 |
| POLR3G | 1 | 0 | 0 | 0 | 0 | 0 |
| POLR3H | 0 | 0 | 0 | 0 | 1 | 0 |
| POLR3K | 1 | 0 | 0 | 0 | 0 | 0 |
| POM121 | 0 | 0 | 0 | 1 | 0 | 0 |
| POM121C | 0 | 0 | 1 | 0 | 0 | 0 |
| POMP | 1 | 0 | 0 | 1 | 0 | 0 |
| POMT1 | 0 | 0 | 0 | 0 | 1 | 0 |
| POMT2 | 0 | 0 | 1 | 0 | 0 | 0 |
| POMZP3 | 0 | 0 | 0 | 1 | 0 | 0 |
| PON2 | 0 | 0 | 1 | 0 | 0 | 0 |
| POP7 | 0 | 0 | 0 | 0 | 1 | 0 |
| POT1 | 0 | 0 | 1 | 0 | 0 | 0 |
| POTEE | 1 | 0 | 0 | 0 | 0 | 0 |
| POU2F1 | 1 | 0 | 1 | 0 | 0 | 0 |
| POU4F1 | 0 | 0 | 0 | 0 | 1 | 0 |
| PPA1 | 0 | 0 | 0 | 1 | 0 | 0 |
| PPA2 | 0 | 1 | 1 | 0 | 0 | 0 |
| PPAP2A | 0 | 0 | 0 | 0 | 0 | 1 |
| PPAPDC2 | 0 | 0 | 0 | 1 | 1 | 0 |
| PPARA | 0 | 0 | 0 | 0 | 1 | 0 |
| PPARD | 0 | 0 | 0 | 0 | 1 | 0 |
| PPARG | 1 | 0 | 0 | 0 | 0 | 0 |
| PPARGC1B | 0 | 1 | 0 | 0 | 0 | 0 |
| PPAT | 1 | 0 | 0 | 0 | 1 | 0 |
| PPCS | 0 | 0 | 0 | 0 | 1 | 0 |
| PPEF2 | 1 | 0 | 0 | 0 | 0 | 0 |
| PPIA | 1 | 0 | 0 | 1 | 0 | 0 |
| PPIAL4G | 1 | 0 | 0 | 0 | 0 | 0 |
| PPIB | 1 | 0 | 0 | 0 | 0 | 0 |
| PPIC | 1 | 1 | 0 | 0 | 0 | 0 |
| PPID | 0 | 0 | 0 | 1 | 1 | 0 |
| PPIF | 0 | 0 | 0 | 0 | 1 | 0 |
| PPIL4 | 1 | 0 | 0 | 0 | 0 | 0 |
| PPIP5K2 | 1 | 0 | 0 | 0 | 1 | 0 |
| PPM1A | 1 | 0 | 0 | 0 | 1 | 1 |
| PPM1B | 0 | 0 | 0 | 1 | 1 | 0 |
| PPM1D | 0 | 0 | 0 | 0 | 1 | 0 |
| PPM1K | 0 | 0 | 0 | 1 | 1 | 0 |
| PPM1L | 0 | 0 | 1 | 0 | 0 | 0 |
| PPME1 | 0 | 0 | 0 | 0 | 1 | 0 |
| PPP1CB | 1 | 0 | 0 | 0 | 1 | 0 |
| PPP1CC | 1 | 0 | 0 | 1 | 0 | 0 |
| PPP1R11 | 0 | 0 | 0 | 1 | 0 | 0 |
| PPP1R12A | 1 | 0 | 0 | 0 | 0 | 0 |
| PPP1R14B | 1 | 0 | 0 | 0 | 0 | 0 |
| PPP1R15B | 1 | 0 | 0 | 0 | 0 | 0 |
| PPP1R2 | 1 | 0 | 0 | 0 | 0 | 0 |
| PPP1R2P4 | 0 | 0 | 1 | 0 | 0 | 0 |
| PPP1R35 | 0 | 0 | 1 | 0 | 1 | 0 |
| PPP1R37 | 0 | 0 | 0 | 0 | 1 | 0 |
| PPP1R8 | 1 | 0 | 0 | 0 | 0 | 0 |
| PPP1R9A | 0 | 0 | 1 | 0 | 0 | 0 |
| PPP2CA | 1 | 0 | 0 | 0 | 0 | 0 |
| PPP2CB | 1 | 0 | 0 | 0 | 1 | 0 |
| PPP2R1A | 1 | 0 | 0 | 0 | 1 | 0 |
| PPP2R2A | 0 | 0 | 0 | 1 | 0 | 0 |
| PPP2R4 | 1 | 0 | 0 | 0 | 0 | 0 |
| PPP2R5B | 0 | 0 | 0 | 0 | 1 | 0 |
| PPP2R5C | 0 | 0 | 0 | 1 | 0 | 0 |
| PPP2R5E | 0 | 0 | 1 | 1 | 0 | 0 |
| PPP3CA | 0 | 0 | 1 | 0 | 0 | 0 |
| PPP3CB | 1 | 0 | 1 | 0 | 1 | 0 |
| PPP3R1 | 1 | 0 | 0 | 0 | 1 | 0 |
| PPP4R1 | 0 | 0 | 1 | 0 | 0 | 0 |
| PPP4R2 | 1 | 0 | 1 | 1 | 0 | 0 |
| PPP6C | 1 | 0 | 0 | 1 | 0 | 0 |
| PPP6R2 | 0 | 0 | 1 | 0 | 0 | 0 |
| PPP6R3 | 1 | 0 | 0 | 0 | 0 | 0 |
| PPRC1 | 0 | 0 | 1 | 0 | 0 | 0 |
| PQBP1 | 0 | 0 | 0 | 1 | 0 | 0 |
| PQLC3 | 0 | 0 | 0 | 0 | 1 | 0 |
| PRDM16 | 0 | 1 | 0 | 0 | 0 | 0 |
| PRDM2 | 0 | 0 | 0 | 0 | 1 | 0 |
| PRDX1 | 1 | 0 | 0 | 0 | 0 | 0 |
| PRDX3 | 1 | 0 | 0 | 0 | 0 | 0 |
| PRDX4 | 1 | 0 | 0 | 0 | 0 | 0 |
| PRDX6 | 1 | 0 | 0 | 0 | 0 | 0 |
| PRELID1 | 0 | 0 | 0 | 1 | 0 | 0 |
| PREPL | 1 | 0 | 0 | 1 | 0 | 0 |
| PRIM2 | 0 | 1 | 0 | 0 | 0 | 0 |
| PRIMPOL | 0 | 0 | 1 | 0 | 0 | 0 |
| PRKAA1 | 1 | 0 | 0 | 0 | 0 | 0 |
| PRKACB | 0 | 0 | 0 | 1 | 0 | 0 |
| PRKAG2 | 0 | 0 | 0 | 1 | 1 | 0 |
| PRKAR1A | 1 | 0 | 1 | 1 | 0 | 0 |
| PRKAR2A | 0 | 0 | 0 | 1 | 0 | 0 |
| PRKCB | 0 | 0 | 0 | 1 | 0 | 0 |
| PRKCI | 0 | 0 | 1 | 0 | 1 | 0 |
| PRKCZ | 0 | 0 | 1 | 0 | 0 | 0 |
| PRKD3 | 1 | 0 | 0 | 0 | 1 | 0 |
| PRKDC | 1 | 0 | 1 | 0 | 0 | 0 |
| PRKRA | 0 | 0 | 0 | 0 | 1 | 0 |
| PRKRIP1 | 0 | 0 | 1 | 0 | 0 | 0 |
| PRKRIR | 0 | 0 | 0 | 0 | 1 | 0 |
| PRKRIRP1 | 0 | 0 | 0 | 0 | 1 | 0 |
| PRKX | 0 | 0 | 1 | 1 | 1 | 0 |
| PRMT1 | 1 | 0 | 0 | 0 | 0 | 0 |
| PRMT5 | 1 | 0 | 0 | 0 | 0 | 0 |
| PRNP | 1 | 0 | 0 | 1 | 0 | 0 |
| PRPF19 | 0 | 0 | 0 | 1 | 1 | 0 |
| PRPF3 | 0 | 0 | 1 | 0 | 0 | 0 |
| PRPF31 | 0 | 0 | 0 | 1 | 0 | 0 |
| PRPF38B | 0 | 1 | 1 | 0 | 1 | 0 |
| PRPF4 | 0 | 0 | 1 | 0 | 0 | 0 |
| PRPF4B | 1 | 0 | 0 | 0 | 0 | 0 |
| PRPF8 | 1 | 0 | 1 | 0 | 0 | 0 |
| PRPS1 | 1 | 0 | 0 | 1 | 0 | 0 |
| PRPSAP2 | 0 | 0 | 0 | 1 | 1 | 0 |
| PRR12 | 0 | 0 | 1 | 0 | 0 | 0 |
| PRR13 | 0 | 0 | 0 | 1 | 0 | 0 |
| PRR14L | 1 | 0 | 1 | 0 | 0 | 0 |
| PRR3 | 1 | 0 | 0 | 0 | 1 | 0 |
| PRRC1 | 0 | 0 | 0 | 1 | 1 | 0 |
| PRRC2A | 0 | 0 | 1 | 0 | 0 | 0 |
| PRRC2B | 1 | 0 | 1 | 0 | 0 | 0 |
| PRRG4 | 1 | 0 | 0 | 0 | 0 | 0 |
| PRRX2 | 1 | 0 | 0 | 0 | 0 | 0 |
| PRSS16 | 0 | 0 | 0 | 0 | 1 | 0 |
| PRSS23 | 1 | 0 | 1 | 0 | 0 | 0 |
| PRTFDC1 | 0 | 0 | 0 | 0 | 1 | 0 |
| PSAP | 1 | 0 | 1 | 0 | 0 | 0 |
| PSAT1 | 1 | 0 | 1 | 0 | 1 | 0 |
| PSCA | 0 | 0 | 0 | 0 | 1 | 0 |
| PSD3 | 1 | 0 | 0 | 0 | 0 | 0 |
| PSEN1 | 0 | 0 | 0 | 1 | 1 | 0 |
| PSMA1 | 1 | 0 | 0 | 0 | 0 | 0 |
| PSMA2 | 1 | 0 | 0 | 0 | 0 | 0 |
| PSMA3 | 1 | 0 | 0 | 0 | 0 | 0 |
| PSMA4 | 1 | 0 | 0 | 1 | 0 | 0 |
| PSMA5 | 1 | 0 | 1 | 0 | 1 | 0 |
| PSMA6 | 1 | 0 | 0 | 0 | 0 | 0 |
| PSMA7 | 1 | 0 | 0 | 0 | 0 | 0 |
| PSMB1 | 1 | 0 | 0 | 0 | 0 | 0 |
| PSMB3 | 1 | 0 | 0 | 0 | 0 | 0 |
| PSMB3P2 | 0 | 0 | 0 | 0 | 1 | 0 |
| PSMB4 | 1 | 0 | 0 | 0 | 0 | 0 |
| PSMB5 | 1 | 0 | 0 | 0 | 0 | 0 |
| PSMB9 | 0 | 0 | 0 | 1 | 0 | 0 |
| PSMC1 | 1 | 0 | 0 | 0 | 0 | 0 |
| PSMC1P13 | 0 | 0 | 0 | 0 | 0 | 1 |
| PSMC6 | 0 | 0 | 0 | 1 | 0 | 0 |
| PSMD1 | 1 | 0 | 0 | 0 | 0 | 0 |
| PSMD10 | 1 | 0 | 0 | 0 | 0 | 0 |
| PSMD11 | 1 | 0 | 0 | 0 | 0 | 0 |
| PSMD12 | 1 | 0 | 0 | 0 | 0 | 0 |
| PSMD13 | 1 | 0 | 0 | 0 | 0 | 0 |
| PSMD2 | 1 | 0 | 0 | 0 | 0 | 0 |
| PSMD3 | 1 | 0 | 1 | 0 | 0 | 0 |
| PSMD6 | 0 | 0 | 0 | 1 | 0 | 0 |
| PSMD8 | 1 | 0 | 0 | 0 | 0 | 0 |
| PSME1 | 0 | 0 | 0 | 1 | 0 | 0 |
| PSME3 | 1 | 0 | 1 | 0 | 1 | 0 |
| PSME4 | 0 | 0 | 0 | 1 | 0 | 0 |
| PSMG2 | 0 | 0 | 0 | 1 | 1 | 0 |
| PSMG3 | 0 | 0 | 0 | 1 | 0 | 0 |
| PSMG4 | 0 | 0 | 1 | 0 | 0 | 0 |
| PSPC1 | 0 | 0 | 1 | 0 | 0 | 0 |
| PTAR1 | 1 | 0 | 1 | 0 | 0 | 0 |
| PTBP1 | 1 | 0 | 0 | 1 | 0 | 0 |
| PTBP2 | 0 | 0 | 1 | 0 | 0 | 0 |
| PTBP3 | 1 | 0 | 1 | 0 | 0 | 0 |
| PTCH1 | 0 | 0 | 1 | 0 | 1 | 0 |
| PTCH2 | 1 | 0 | 0 | 0 | 0 | 0 |
| PTDSS1 | 0 | 0 | 0 | 0 | 1 | 0 |
| PTGER3 | 0 | 0 | 0 | 0 | 1 | 0 |
| PTGER4 | 0 | 0 | 0 | 1 | 0 | 0 |
| PTGES3 | 1 | 0 | 0 | 1 | 0 | 0 |
| PTGES3P1 | 0 | 0 | 0 | 0 | 1 | 0 |
| PTGFRN | 0 | 0 | 1 | 0 | 1 | 0 |
| PTGR1 | 1 | 0 | 0 | 0 | 0 | 0 |
| PTHLH | 0 | 0 | 0 | 0 | 1 | 0 |
| PTMA | 1 | 0 | 0 | 1 | 0 | 0 |
| PTP4A1 | 1 | 0 | 1 | 1 | 0 | 0 |
| PTP4A1P7 | 0 | 0 | 0 | 0 | 1 | 0 |
| PTP4A2 | 1 | 0 | 0 | 1 | 0 | 0 |
| PTPLAD1 | 0 | 0 | 0 | 0 | 1 | 0 |
| PTPLB | 0 | 0 | 0 | 0 | 1 | 0 |
| PTPN1 | 1 | 0 | 1 | 0 | 0 | 0 |
| PTPN11 | 1 | 0 | 0 | 1 | 0 | 0 |
| PTPN12 | 1 | 0 | 0 | 0 | 0 | 0 |
| PTPN2 | 0 | 0 | 1 | 1 | 0 | 0 |
| PTPN6 | 1 | 0 | 0 | 0 | 0 | 0 |
| PTPN9 | 0 | 0 | 0 | 0 | 1 | 0 |
| PTPRC | 0 | 0 | 0 | 1 | 1 | 0 |
| PTPRE | 0 | 1 | 0 | 0 | 0 | 0 |
| PTPRF | 0 | 0 | 1 | 0 | 0 | 0 |
| PTPRJ | 0 | 0 | 1 | 0 | 0 | 0 |
| PTPRM | 0 | 0 | 1 | 0 | 0 | 0 |
| PTPRN2 | 0 | 1 | 0 | 0 | 0 | 0 |
| PTPRR | 0 | 0 | 0 | 0 | 1 | 0 |
| PTPRS | 0 | 0 | 1 | 0 | 0 | 0 |
| PTRH2 | 0 | 0 | 1 | 0 | 0 | 0 |
| PTS | 0 | 0 | 0 | 0 | 1 | 0 |
| PTTG1 | 1 | 0 | 0 | 0 | 0 | 0 |
| PUM1 | 1 | 0 | 1 | 1 | 0 | 0 |
| PUM2 | 0 | 0 | 0 | 1 | 0 | 0 |
| PURA | 1 | 0 | 0 | 1 | 1 | 0 |
| PURB | 1 | 0 | 0 | 0 | 0 | 0 |
| PUS10 | 0 | 0 | 0 | 0 | 1 | 0 |
| PUS7 | 0 | 0 | 1 | 0 | 0 | 0 |
| PUS7L | 0 | 0 | 0 | 0 | 1 | 0 |
| PVRL3 | 1 | 0 | 0 | 0 | 0 | 0 |
| PVT1 | 0 | 0 | 1 | 0 | 0 | 0 |
| PXMP4 | 0 | 0 | 0 | 0 | 1 | 0 |
| PXN | 1 | 0 | 0 | 1 | 1 | 0 |
| PYGB | 1 | 0 | 0 | 0 | 0 | 0 |
| PYGO2 | 1 | 0 | 1 | 0 | 1 | 0 |
| PYURF | 1 | 0 | 0 | 0 | 1 | 0 |
| QDPR | 1 | 0 | 0 | 0 | 1 | 0 |
| QKI | 1 | 0 | 0 | 1 | 1 | 0 |
| QSER1 | 1 | 0 | 0 | 0 | 0 | 0 |
| QSOX2 | 0 | 0 | 1 | 0 | 1 | 0 |
| R3HDM4 | 0 | 0 | 1 | 0 | 1 | 0 |
| RAB10 | 1 | 0 | 0 | 1 | 0 | 0 |
| RAB11A | 1 | 0 | 1 | 1 | 0 | 0 |
| RAB11B | 0 | 0 | 1 | 0 | 1 | 0 |
| RAB11B-AS1 | 0 | 0 | 0 | 0 | 1 | 0 |
| RAB11FIP3 | 0 | 0 | 1 | 0 | 0 | 0 |
| RAB12 | 1 | 1 | 1 | 0 | 1 | 0 |
| RAB14 | 1 | 0 | 1 | 0 | 1 | 0 |
| RAB18 | 1 | 0 | 1 | 1 | 1 | 0 |
| RAB1A | 1 | 0 | 0 | 1 | 0 | 0 |
| RAB23 | 1 | 0 | 0 | 0 | 0 | 0 |
| RAB27A | 0 | 0 | 0 | 1 | 0 | 0 |
| RAB28 | 0 | 0 | 0 | 1 | 1 | 0 |
| RAB2A | 1 | 0 | 0 | 0 | 0 | 0 |
| RAB2B | 1 | 0 | 0 | 1 | 0 | 0 |
| RAB31 | 0 | 0 | 1 | 0 | 1 | 0 |
| RAB33B | 0 | 0 | 0 | 0 | 1 | 0 |
| RAB35 | 0 | 0 | 0 | 0 | 1 | 0 |
| RAB40B | 0 | 0 | 0 | 0 | 1 | 0 |
| RAB41 | 0 | 0 | 0 | 0 | 1 | 0 |
| RAB4A | 0 | 0 | 0 | 1 | 1 | 0 |
| RAB5A | 0 | 0 | 1 | 0 | 0 | 0 |
| RAB5B | 0 | 0 | 0 | 1 | 0 | 0 |
| RAB5C | 1 | 0 | 1 | 1 | 0 | 0 |
| RAB6A | 0 | 0 | 0 | 1 | 0 | 0 |
| RAB6B | 0 | 0 | 0 | 0 | 1 | 0 |
| RAB7A | 1 | 0 | 1 | 0 | 1 | 0 |
| RAB7L1 | 0 | 0 | 0 | 0 | 1 | 0 |
| RAB8B | 0 | 0 | 0 | 1 | 0 | 0 |
| RABAC1 | 0 | 1 | 0 | 0 | 0 | 0 |
| RABEP2 | 1 | 0 | 0 | 0 | 0 | 0 |
| RABEPK | 0 | 0 | 1 | 0 | 0 | 0 |
| RABGAP1 | 0 | 1 | 1 | 1 | 0 | 0 |
| RABGEF1 | 0 | 0 | 1 | 0 | 0 | 0 |
| RABGGTB | 0 | 0 | 1 | 0 | 1 | 0 |
| RABL2A | 0 | 0 | 0 | 1 | 1 | 0 |
| RABL2B | 1 | 0 | 1 | 0 | 0 | 0 |
| RABL3 | 0 | 0 | 0 | 0 | 1 | 0 |
| RABL5 | 0 | 0 | 0 | 0 | 1 | 0 |
| RAC1 | 1 | 0 | 1 | 1 | 1 | 0 |
| RAC1P2 | 0 | 0 | 1 | 0 | 0 | 0 |
| RAC1P4 | 0 | 0 | 0 | 0 | 1 | 0 |
| RAC2 | 0 | 0 | 0 | 1 | 0 | 0 |
| RACGAP1 | 1 | 0 | 0 | 0 | 0 | 0 |
| RAD21 | 1 | 0 | 0 | 0 | 0 | 0 |
| RAD23B | 1 | 0 | 0 | 0 | 0 | 0 |
| RAD51B | 0 | 0 | 1 | 0 | 0 | 0 |
| RAD54L | 0 | 0 | 1 | 0 | 0 | 0 |
| RALA | 0 | 0 | 0 | 1 | 0 | 0 |
| RAN | 1 | 0 | 0 | 1 | 0 | 0 |
| RANBP1 | 1 | 0 | 0 | 0 | 0 | 0 |
| RANBP6 | 1 | 0 | 0 | 0 | 1 | 0 |
| RANGAP1 | 0 | 0 | 1 | 0 | 0 | 0 |
| RANP2 | 0 | 0 | 0 | 0 | 1 | 0 |
| RANP8 | 0 | 0 | 0 | 0 | 1 | 0 |
| RAP1A | 0 | 0 | 0 | 1 | 0 | 0 |
| RAP1B | 1 | 0 | 0 | 0 | 0 | 0 |
| RAP2A | 1 | 0 | 1 | 1 | 1 | 0 |
| RAP2B | 1 | 0 | 1 | 0 | 0 | 0 |
| RAP2C | 0 | 0 | 0 | 0 | 1 | 0 |
| RAPGEF6 | 0 | 0 | 0 | 1 | 0 | 0 |
| RAPH1 | 1 | 0 | 0 | 0 | 0 | 0 |
| RASA2 | 1 | 0 | 1 | 0 | 0 | 0 |
| RASSF3 | 1 | 0 | 0 | 0 | 0 | 0 |
| RASSF5 | 0 | 0 | 0 | 1 | 0 | 0 |
| RAVER2 | 0 | 0 | 1 | 0 | 0 | 0 |
| RB1 | 0 | 0 | 1 | 0 | 0 | 0 |
| RBBP4 | 1 | 0 | 0 | 1 | 0 | 0 |
| RBBP6 | 0 | 0 | 0 | 0 | 1 | 0 |
| RBBP7 | 1 | 0 | 0 | 0 | 1 | 0 |
| RBBP8 | 0 | 0 | 1 | 0 | 0 | 0 |
| RBFA | 0 | 0 | 1 | 0 | 0 | 0 |
| RBFOX2 | 1 | 0 | 1 | 0 | 0 | 0 |
| RBM12 | 0 | 0 | 0 | 1 | 0 | 0 |
| RBM12B | 0 | 0 | 0 | 0 | 1 | 0 |
| RBM15 | 1 | 0 | 1 | 0 | 0 | 0 |
| RBM17 | 0 | 0 | 0 | 1 | 0 | 0 |
| RBM18 | 0 | 0 | 0 | 0 | 1 | 0 |
| RBM22 | 1 | 0 | 0 | 1 | 0 | 0 |
| RBM23 | 0 | 0 | 0 | 0 | 1 | 0 |
| RBM25 | 1 | 0 | 1 | 1 | 0 | 0 |
| RBM26 | 0 | 0 | 1 | 0 | 1 | 0 |
| RBM27 | 1 | 0 | 1 | 0 | 0 | 0 |
| RBM3 | 1 | 0 | 0 | 1 | 0 | 0 |
| RBM33 | 0 | 0 | 1 | 0 | 0 | 0 |
| RBM34 | 0 | 0 | 0 | 1 | 0 | 0 |
| RBM35A | 0 | 1 | 0 | 0 | 0 | 0 |
| RBM39 | 1 | 0 | 1 | 1 | 0 | 0 |
| RBM47 | 0 | 0 | 1 | 0 | 0 | 0 |
| RBM4B | 0 | 0 | 0 | 0 | 1 | 0 |
| RBM5 | 1 | 0 | 0 | 0 | 0 | 0 |
| RBM8A | 1 | 0 | 0 | 0 | 0 | 0 |
| RBMS1 | 1 | 0 | 0 | 1 | 0 | 0 |
| RBMX | 1 | 0 | 0 | 1 | 0 | 0 |
| RBMXL1 | 1 | 0 | 0 | 0 | 0 | 0 |
| RBP2 | 0 | 0 | 0 | 0 | 0 | 1 |
| RBPJ | 1 | 0 | 1 | 1 | 0 | 0 |
| RBX1 | 1 | 0 | 1 | 1 | 0 | 0 |
| RC3H1 | 1 | 0 | 0 | 0 | 0 | 0 |
| RC3H2 | 1 | 0 | 0 | 0 | 0 | 0 |
| RCAN1 | 1 | 0 | 0 | 0 | 1 | 0 |
| RCBTB1 | 0 | 0 | 0 | 0 | 1 | 0 |
| RCC1 | 1 | 0 | 1 | 0 | 0 | 0 |
| RCC2 | 1 | 0 | 0 | 0 | 0 | 0 |
| RCC2P6 | 0 | 0 | 0 | 0 | 1 | 0 |
| RCCD1 | 1 | 0 | 1 | 0 | 1 | 0 |
| RCHY1 | 0 | 0 | 0 | 0 | 1 | 0 |
| RCN1 | 1 | 0 | 0 | 0 | 0 | 0 |
| RCN2 | 1 | 0 | 0 | 0 | 0 | 0 |
| RCOR1 | 1 | 0 | 1 | 0 | 0 | 0 |
| RDH10 | 0 | 0 | 0 | 0 | 1 | 0 |
| RDH11 | 0 | 0 | 1 | 0 | 1 | 0 |
| RDX | 1 | 0 | 0 | 0 | 0 | 0 |
| RECQL | 0 | 0 | 0 | 1 | 0 | 0 |
| REEP3 | 1 | 0 | 0 | 1 | 1 | 0 |
| REEP4 | 0 | 0 | 0 | 0 | 1 | 0 |
| REEP5 | 0 | 0 | 0 | 0 | 1 | 0 |
| RER1 | 0 | 0 | 0 | 1 | 0 | 0 |
| REST | 1 | 0 | 0 | 0 | 0 | 0 |
| REXO1L1 | 1 | 0 | 0 | 0 | 0 | 0 |
| REXO2 | 1 | 0 | 0 | 0 | 0 | 0 |
| RFC3 | 0 | 0 | 1 | 0 | 0 | 0 |
| RFESD | 0 | 1 | 0 | 0 | 0 | 0 |
| RFFL | 0 | 1 | 0 | 0 | 0 | 0 |
| RFK | 1 | 0 | 0 | 1 | 0 | 0 |
| RFPL4B | 0 | 0 | 0 | 0 | 1 | 0 |
| RFT1 | 0 | 0 | 0 | 0 | 1 | 0 |
| RFWD3 | 1 | 0 | 0 | 0 | 0 | 0 |
| RFX1 | 0 | 1 | 0 | 0 | 0 | 0 |
| RFX7 | 1 | 0 | 0 | 1 | 0 | 0 |
| RFXAP | 0 | 0 | 1 | 0 | 1 | 0 |
| RG9MTD1 | 0 | 0 | 0 | 1 | 0 | 0 |
| RGL3 | 0 | 1 | 0 | 0 | 0 | 0 |
| RGP1 | 0 | 0 | 0 | 0 | 1 | 0 |
| RGS1 | 0 | 0 | 0 | 1 | 0 | 0 |
| RGS3 | 0 | 1 | 0 | 0 | 0 | 0 |
| RHBDD2 | 0 | 0 | 0 | 0 | 1 | 0 |
| RHEB | 1 | 0 | 0 | 0 | 0 | 0 |
| RHEBL1 | 0 | 0 | 0 | 0 | 1 | 0 |
| RHOA | 1 | 0 | 1 | 1 | 0 | 0 |
| RHOC | 0 | 1 | 0 | 0 | 0 | 0 |
| RHPN1-AS1 | 0 | 0 | 0 | 0 | 1 | 0 |
| RICTOR | 0 | 0 | 1 | 0 | 0 | 0 |
| RIF1 | 1 | 0 | 0 | 1 | 0 | 0 |
| RILP | 0 | 1 | 0 | 0 | 0 | 0 |
| RIMS1 | 0 | 0 | 1 | 0 | 0 | 0 |
| RINT1 | 0 | 0 | 1 | 0 | 0 | 0 |
| RIOK3 | 0 | 0 | 1 | 1 | 0 | 0 |
| RIT1 | 0 | 0 | 0 | 1 | 0 | 0 |
| RLIM | 1 | 0 | 0 | 0 | 1 | 0 |
| RMI1 | 0 | 0 | 1 | 0 | 0 | 0 |
| RMI2 | 0 | 0 | 0 | 0 | 1 | 0 |
| RMND5A | 1 | 0 | 0 | 0 | 1 | 0 |
| RMRP | 0 | 0 | 1 | 0 | 0 | 0 |
| RN7SK | 0 | 0 | 1 | 0 | 0 | 0 |
| RN7SKP7 | 0 | 0 | 0 | 0 | 1 | 0 |
| RN7SKP9 | 0 | 0 | 0 | 0 | 1 | 0 |
| RN7SL1 | 0 | 0 | 1 | 0 | 0 | 0 |
| RNA5SP141 | 0 | 0 | 1 | 0 | 0 | 0 |
| RNA5SP150 | 0 | 0 | 1 | 0 | 0 | 0 |
| RNA5SP202 | 0 | 0 | 1 | 0 | 0 | 0 |
| RNA5SP21 | 0 | 0 | 0 | 0 | 1 | 0 |
| RNA5SP216 | 0 | 0 | 1 | 0 | 0 | 0 |
| RNA5SP243 | 0 | 0 | 1 | 0 | 0 | 0 |
| RNA5SP249 | 0 | 0 | 0 | 0 | 1 | 0 |
| RNA5SP25 | 0 | 0 | 0 | 0 | 1 | 0 |
| RNA5SP319 | 0 | 0 | 0 | 0 | 1 | 0 |
| RNA5SP33 | 0 | 0 | 0 | 0 | 1 | 0 |
| RNA5SP372 | 0 | 0 | 0 | 0 | 1 | 0 |
| RNA5SP394 | 0 | 0 | 0 | 0 | 1 | 0 |
| RNA5SP479 | 0 | 0 | 0 | 0 | 1 | 0 |
| RNASE4 | 0 | 0 | 0 | 0 | 1 | 0 |
| RNASEH1 | 1 | 0 | 0 | 0 | 1 | 0 |
| RNASEH2B | 0 | 0 | 0 | 0 | 1 | 0 |
| RNASEK | 0 | 0 | 1 | 0 | 0 | 0 |
| RNASEK-C17orf49 | 0 | 0 | 1 | 0 | 0 | 0 |
| RND3 | 1 | 0 | 0 | 0 | 0 | 0 |
| RNF103 | 0 | 0 | 0 | 0 | 1 | 0 |
| RNF103-CHMP3 | 0 | 0 | 0 | 0 | 1 | 0 |
| RNF11 | 1 | 0 | 0 | 1 | 1 | 0 |
| RNF114 | 1 | 0 | 1 | 1 | 1 | 0 |
| RNF121 | 0 | 0 | 0 | 0 | 1 | 0 |
| RNF13 | 0 | 0 | 1 | 0 | 0 | 0 |
| RNF138 | 0 | 0 | 0 | 1 | 0 | 0 |
| RNF138P1 | 0 | 0 | 0 | 0 | 0 | 1 |
| RNF139 | 1 | 0 | 0 | 0 | 1 | 0 |
| RNF14 | 1 | 0 | 0 | 1 | 0 | 0 |
| RNF141 | 0 | 0 | 0 | 0 | 1 | 0 |
| RNF144A | 1 | 0 | 0 | 0 | 1 | 0 |
| RNF146 | 0 | 0 | 0 | 1 | 1 | 0 |
| RNF149 | 0 | 0 | 0 | 1 | 0 | 0 |
| RNF151 | 0 | 0 | 0 | 0 | 1 | 0 |
| RNF165 | 0 | 0 | 1 | 0 | 0 | 0 |
| RNF166 | 0 | 0 | 0 | 0 | 1 | 0 |
| RNF170 | 0 | 0 | 0 | 0 | 1 | 0 |
| RNF182 | 0 | 0 | 0 | 0 | 1 | 0 |
| RNF187 | 0 | 0 | 1 | 0 | 0 | 0 |
| RNF19A | 0 | 0 | 0 | 0 | 1 | 0 |
| RNF212 | 0 | 0 | 1 | 0 | 0 | 0 |
| RNF213 | 0 | 0 | 1 | 0 | 0 | 0 |
| RNF216 | 0 | 0 | 1 | 0 | 0 | 0 |
| RNF219 | 1 | 0 | 0 | 0 | 1 | 0 |
| RNF220 | 0 | 0 | 0 | 0 | 1 | 0 |
| RNF24 | 0 | 0 | 1 | 0 | 0 | 0 |
| RNF34 | 0 | 0 | 0 | 0 | 1 | 0 |
| RNF38 | 1 | 0 | 0 | 1 | 0 | 0 |
| RNF4 | 0 | 0 | 1 | 0 | 0 | 0 |
| RNFT1 | 0 | 0 | 0 | 0 | 1 | 0 |
| RNFT2 | 0 | 0 | 0 | 0 | 1 | 0 |
| RNPS1 | 1 | 0 | 0 | 0 | 0 | 0 |
| RNU1-12P | 0 | 0 | 0 | 0 | 1 | 0 |
| RNU1-19P | 0 | 0 | 0 | 0 | 1 | 0 |
| RNU12 | 0 | 0 | 1 | 0 | 0 | 0 |
| RNU2-2P | 0 | 0 | 1 | 0 | 0 | 0 |
| RNU2-59P | 0 | 0 | 1 | 0 | 0 | 0 |
| RNU2-6P | 0 | 0 | 1 | 0 | 0 | 0 |
| RNU6-53 | 0 | 0 | 0 | 0 | 1 | 0 |
| RNU6-78 | 0 | 0 | 0 | 0 | 1 | 0 |
| RNU6-8 | 0 | 0 | 0 | 0 | 1 | 0 |
| RNU7-77P | 0 | 0 | 0 | 0 | 1 | 0 |
| RNY1 | 0 | 0 | 1 | 0 | 0 | 0 |
| RNY4P3 | 0 | 0 | 0 | 0 | 1 | 0 |
| RNY4P8 | 0 | 0 | 0 | 0 | 1 | 0 |
| ROMO1 | 1 | 0 | 0 | 0 | 0 | 0 |
| ROR2 | 0 | 0 | 1 | 0 | 0 | 0 |
| RP11-1012A1.4 | 0 | 0 | 0 | 0 | 1 | 0 |
| RP11-1035H13.3 | 1 | 0 | 0 | 0 | 0 | 0 |
| RP11-1100L3.4 | 0 | 0 | 0 | 0 | 1 | 0 |
| RP11-110H1.4 | 0 | 0 | 0 | 0 | 1 | 0 |
| RP11-139K1.2 | 0 | 0 | 0 | 0 | 1 | 0 |
| RP11-13N13.2 | 0 | 0 | 0 | 0 | 1 | 0 |
| RP11-143K11.5 | 0 | 0 | 0 | 0 | 0 | 1 |
| RP11-152N13.8 | 0 | 0 | 0 | 0 | 1 | 0 |
| RP11-155D18.12 | 0 | 0 | 0 | 0 | 1 | 0 |
| RP11-158K1.3 | 0 | 0 | 0 | 0 | 1 | 0 |
| RP11-159D12.5 | 1 | 0 | 0 | 0 | 0 | 0 |
| RP11-160C18.4 | 0 | 0 | 0 | 0 | 1 | 0 |
| RP11-164H5.1 | 0 | 0 | 0 | 0 | 1 | 0 |
| RP11-16C1.2 | 0 | 0 | 0 | 0 | 1 | 0 |
| RP11-170N16.2 | 0 | 0 | 0 | 0 | 1 | 0 |
| RP11-173D9.2 | 0 | 0 | 0 | 0 | 1 | 0 |
| RP11-176H8.1 | 0 | 0 | 0 | 0 | 1 | 0 |
| RP11-178F10.1 | 0 | 0 | 0 | 0 | 1 | 0 |
| RP11-178L8.4 | 0 | 0 | 0 | 0 | 1 | 0 |
| RP11-181C21.6 | 0 | 0 | 0 | 0 | 1 | 0 |
| RP11-181G12.4 | 0 | 0 | 0 | 0 | 1 | 0 |
| RP11-181G12.5 | 0 | 0 | 0 | 0 | 1 | 0 |
| RP11-182I10.3 | 0 | 0 | 0 | 0 | 1 | 0 |
| RP11-182I10.4 | 0 | 0 | 0 | 0 | 1 | 0 |
| RP11-192H23.4 | 0 | 0 | 0 | 0 | 1 | 0 |
| RP11-197P3.5 | 0 | 0 | 0 | 0 | 1 | 0 |
| RP11-198M6.5 | 0 | 0 | 0 | 0 | 1 | 0 |
| RP11-199F11.2 | 0 | 0 | 0 | 0 | 1 | 0 |
| RP11-205M20.7 | 0 | 0 | 0 | 0 | 1 | 0 |
| RP11-206L10.11 | 0 | 0 | 0 | 0 | 1 | 0 |
| RP11-214O1.2 | 0 | 0 | 0 | 0 | 1 | 0 |
| RP11-214O1.3 | 0 | 0 | 0 | 0 | 1 | 0 |
| RP11-214O14.1 | 0 | 0 | 0 | 0 | 1 | 0 |
| RP11-226L15.5 | 0 | 0 | 0 | 0 | 1 | 0 |
| RP11-234B24.6 | 0 | 0 | 0 | 0 | 1 | 0 |
| RP11-243M5.1 | 0 | 0 | 0 | 0 | 1 | 0 |
| RP11-252K23.1 | 0 | 0 | 0 | 0 | 1 | 0 |
| RP11-252K23.2 | 0 | 0 | 0 | 0 | 1 | 0 |
| RP11-258A12.3 | 0 | 0 | 0 | 0 | 1 | 0 |
| RP11-264B17.3 | 0 | 0 | 0 | 0 | 1 | 0 |
| RP11-274K13.5 | 0 | 0 | 0 | 0 | 1 | 0 |
| RP11-277L2.2 | 0 | 0 | 0 | 0 | 1 | 1 |
| RP11-27M24.2 | 0 | 0 | 0 | 0 | 1 | 0 |
| RP1-128M12.3 | 0 | 0 | 0 | 0 | 1 | 0 |
| RP11-290D2.4 | 0 | 0 | 0 | 0 | 1 | 0 |
| RP11-293I14.2 | 0 | 0 | 0 | 0 | 1 | 0 |
| RP11-295K3.1 | 0 | 0 | 0 | 0 | 1 | 0 |
| RP11-296A18.6 | 0 | 0 | 0 | 0 | 1 | 0 |
| RP11-296A18.7 | 0 | 0 | 0 | 0 | 1 | 0 |
| RP11-298I3.5 | 0 | 0 | 0 | 0 | 1 | 0 |
| RP11-302F12.2 | 0 | 0 | 0 | 0 | 1 | 0 |
| RP11-303E16.8 | 0 | 0 | 0 | 0 | 1 | 0 |
| RP11-304L19.3 | 0 | 0 | 0 | 0 | 1 | 0 |
| RP11-304L20.1 | 0 | 0 | 0 | 0 | 1 | 0 |
| RP11-307L3.2 | 0 | 0 | 0 | 0 | 1 | 0 |
| RP11-307P22.1 | 0 | 0 | 0 | 0 | 1 | 0 |
| RP1-130H16.18 | 0 | 0 | 0 | 0 | 1 | 0 |
| RP11-315D16.2 | 1 | 0 | 0 | 0 | 1 | 0 |
| RP11-318A15.7 | 1 | 0 | 0 | 0 | 0 | 0 |
| RP11-325K4.2 | 0 | 0 | 0 | 0 | 1 | 0 |
| RP11-325K4.3 | 0 | 0 | 0 | 0 | 1 | 0 |
| RP11-330L19.4 | 0 | 0 | 0 | 0 | 1 | 0 |
| RP11-343C2.3 | 1 | 0 | 0 | 0 | 0 | 0 |
| RP11-348K2.2 | 0 | 0 | 0 | 0 | 1 | 0 |
| RP11-351M8.2 | 0 | 0 | 0 | 0 | 1 | 0 |
| RP11-353N4.6 | 0 | 0 | 0 | 0 | 1 | 1 |
| RP11-357H14.19 | 0 | 0 | 0 | 0 | 1 | 0 |
| RP11-358L16.2 | 0 | 0 | 0 | 0 | 1 | 0 |
| RP11-35B20.1 | 0 | 0 | 0 | 0 | 1 | 0 |
| RP11-361A23.2 | 0 | 0 | 0 | 0 | 1 | 0 |
| RP11-361L15.4 | 0 | 0 | 0 | 0 | 1 | 0 |
| RP11-363E7.3 | 0 | 0 | 0 | 0 | 1 | 0 |
| RP11-371E8.4 | 0 | 0 | 0 | 0 | 1 | 0 |
| RP11-379K17.9 | 0 | 0 | 0 | 0 | 1 | 0 |
| RP11-380M21.2 | 0 | 0 | 0 | 0 | 1 | 0 |
| RP11-380M21.4 | 0 | 0 | 0 | 0 | 1 | 0 |
| RP11-380M3.1 | 0 | 0 | 0 | 0 | 1 | 0 |
| RP11-388C12.2 | 0 | 0 | 0 | 0 | 1 | 0 |
| RP11-388C12.8 | 0 | 0 | 0 | 0 | 1 | 0 |
| RP11-397O8.4 | 0 | 0 | 0 | 0 | 1 | 0 |
| RP11-407G23.4 | 0 | 0 | 0 | 0 | 1 | 0 |
| RP11-413E1.4 | 0 | 0 | 0 | 0 | 1 | 0 |
| RP11-416K24.2 | 0 | 0 | 0 | 0 | 1 | 0 |
| RP11-432J9.4 | 0 | 0 | 0 | 0 | 1 | 0 |
| RP11-434D12.1 | 0 | 0 | 0 | 0 | 1 | 0 |
| RP11-438N5.4 | 0 | 0 | 0 | 0 | 1 | 0 |
| RP11-44I10.6 | 0 | 0 | 0 | 0 | 1 | 0 |
| RP11-457K10.2 | 0 | 0 | 0 | 0 | 1 | 0 |
| RP11-457M11.2 | 0 | 0 | 0 | 0 | 1 | 0 |
| RP11-463D19.2 | 0 | 0 | 0 | 0 | 1 | 0 |
| RP11-464D20.2 | 0 | 0 | 0 | 0 | 0 | 1 |
| RP11-467L19.11 | 0 | 0 | 0 | 0 | 1 | 0 |
| RP11-468E2.2 | 0 | 0 | 0 | 0 | 1 | 0 |
| RP11-46A10.4 | 0 | 0 | 0 | 0 | 1 | 0 |
| RP11-46A10.5 | 0 | 0 | 0 | 0 | 1 | 0 |
| RP11-471L13.3 | 0 | 0 | 0 | 0 | 1 | 0 |
| RP11-472I20.1 | 0 | 0 | 0 | 0 | 1 | 0 |
| RP11-472I20.2 | 0 | 0 | 0 | 0 | 1 | 0 |
| RP11-474N24.2 | 0 | 0 | 0 | 0 | 1 | 0 |
| RP11-476C8.3 | 0 | 0 | 0 | 0 | 1 | 0 |
| RP11-47K11.2 | 0 | 0 | 0 | 0 | 1 | 0 |
| RP11-480I12.2 | 0 | 0 | 0 | 0 | 1 | 0 |
| RP11-483M24.2 | 0 | 0 | 0 | 0 | 1 | 0 |
| RP11-495P10.2 | 0 | 0 | 0 | 0 | 1 | 0 |
| RP11-503E24.2 | 0 | 0 | 0 | 0 | 1 | 0 |
| RP11-506B6.6 | 0 | 0 | 0 | 0 | 1 | 0 |
| RP11-512M8.5 | 0 | 0 | 0 | 0 | 1 | 0 |
| RP11-518L10.2 | 0 | 0 | 0 | 0 | 1 | 0 |
| RP11-519C12.1 | 0 | 0 | 0 | 0 | 1 | 0 |
| RP11-529H2.2 | 0 | 0 | 0 | 0 | 1 | 0 |
| RP11-552J9.5 | 0 | 0 | 0 | 0 | 1 | 0 |
| RP11-552M11.4 | 0 | 0 | 0 | 0 | 1 | 0 |
| RP11-566K19.8 | 0 | 0 | 0 | 0 | 1 | 0 |
| RP11-570P14.1 | 0 | 0 | 0 | 0 | 1 | 0 |
| RP11-571M6.15 | 0 | 0 | 0 | 0 | 1 | 0 |
| RP11-578F21.10 | 0 | 0 | 0 | 0 | 1 | 0 |
| RP11-606P2.1 | 0 | 0 | 0 | 0 | 1 | 0 |
| RP11-613D13.4 | 0 | 0 | 0 | 0 | 1 | 0 |
| RP11-613F22.5 | 0 | 0 | 0 | 0 | 1 | 0 |
| RP11-613F22.6 | 0 | 0 | 0 | 0 | 1 | 0 |
| RP11-613F22.8 | 0 | 0 | 0 | 0 | 1 | 0 |
| RP11-613M10.9 | 0 | 0 | 0 | 0 | 1 | 0 |
| RP11-615I2.7 | 0 | 0 | 0 | 0 | 1 | 0 |
| RP11-616M17.1 | 0 | 0 | 0 | 0 | 1 | 0 |
| RP11-618P17.4 | 1 | 0 | 0 | 0 | 0 | 0 |
| RP11-61N20.3 | 0 | 0 | 0 | 0 | 1 | 0 |
| RP11-631N16.2 | 0 | 0 | 0 | 0 | 1 | 0 |
| RP11-640M9.1 | 0 | 0 | 0 | 0 | 1 | 1 |
| RP11-649E7.7 | 0 | 0 | 0 | 0 | 1 | 0 |
| RP1-164F3.9 | 1 | 0 | 0 | 0 | 0 | 0 |
| RP11-664D7.4 | 1 | 0 | 0 | 0 | 0 | 0 |
| RP11-697E2.6 | 0 | 0 | 0 | 0 | 1 | 0 |
| RP11-697E2.7 | 0 | 0 | 0 | 0 | 1 | 0 |
| RP1-169K13.3 | 0 | 0 | 0 | 0 | 0 | 1 |
| RP11-6N17.9 | 0 | 0 | 0 | 0 | 1 | 0 |
| RP11-715F3.2 | 0 | 0 | 0 | 0 | 1 | 0 |
| RP11-718G2.3 | 0 | 0 | 0 | 0 | 1 | 0 |
| RP11-727A23.1 | 0 | 0 | 0 | 0 | 1 | 0 |
| RP11-727A23.8 | 0 | 0 | 0 | 0 | 1 | 0 |
| RP11-729L2.2 | 0 | 0 | 0 | 0 | 1 | 0 |
| RP11-73O6.4 | 0 | 0 | 0 | 0 | 1 | 0 |
| RP11-762I7.5 | 1 | 0 | 0 | 0 | 0 | 0 |
| RP11-767N6.7 | 0 | 0 | 0 | 0 | 1 | 0 |
| RP11-77K12.1 | 0 | 0 | 0 | 0 | 1 | 0 |
| RP11-793H13.10 | 0 | 0 | 0 | 0 | 1 | 0 |
| RP11-79D8.2 | 0 | 0 | 0 | 0 | 1 | 0 |
| RP11-806L2.5 | 0 | 0 | 0 | 0 | 1 | 0 |
| RP11-807G9.2 | 0 | 0 | 0 | 0 | 1 | 0 |
| RP11-80H5.5 | 0 | 0 | 0 | 0 | 1 | 0 |
| RP11-81K2.1 | 1 | 0 | 0 | 0 | 0 | 0 |
| RP11-831H9.11 | 0 | 0 | 0 | 0 | 1 | 0 |
| RP11-835E18.5 | 0 | 0 | 0 | 0 | 1 | 0 |
| RP11-841C19.1 | 0 | 0 | 0 | 0 | 1 | 0 |
| RP11-841C19.3 | 0 | 0 | 0 | 0 | 1 | 0 |
| RP11-84A14.4 | 0 | 0 | 0 | 0 | 1 | 0 |
| RP11-850P15.1 | 0 | 0 | 0 | 0 | 1 | 0 |
| RP11-876N24.5 | 0 | 0 | 0 | 0 | 1 | 0 |
| RP1-187N21.4 | 1 | 0 | 0 | 0 | 0 | 0 |
| RP11-886H22.1 | 0 | 0 | 0 | 0 | 1 | 0 |
| RP11-886P16.6 | 0 | 0 | 0 | 0 | 1 | 0 |
| RP11-887P2.6 | 0 | 0 | 0 | 0 | 1 | 0 |
| RP11-8J23.1 | 0 | 0 | 0 | 0 | 1 | 0 |
| RP11-8J9.1 | 0 | 0 | 0 | 0 | 1 | 0 |
| RP11-923I11.4 | 0 | 0 | 0 | 0 | 1 | 0 |
| RP11-923I11.5 | 0 | 0 | 0 | 0 | 1 | 0 |
| RP11-923I11.6 | 0 | 0 | 0 | 0 | 1 | 0 |
| RP11-93O14.2 | 0 | 0 | 0 | 0 | 1 | 0 |
| RP11-973H7.2 | 0 | 0 | 0 | 0 | 1 | 0 |
| RP11-98D18.3 | 0 | 0 | 0 | 0 | 1 | 0 |
| RP1-206D15.5 | 0 | 0 | 0 | 0 | 1 | 0 |
| RP1-257A7.4 | 0 | 0 | 0 | 0 | 1 | 0 |
| RP1-266L20.9 | 0 | 0 | 0 | 0 | 1 | 0 |
| RP13-15M17.1 | 0 | 0 | 0 | 0 | 1 | 0 |
| RP1-317E23.6 | 0 | 0 | 0 | 0 | 1 | 0 |
| RP13-371D18.2 | 0 | 0 | 0 | 0 | 1 | 0 |
| RP13-503K1.4 | 0 | 0 | 0 | 0 | 1 | 0 |
| RP13-512J5.1 | 1 | 0 | 0 | 0 | 1 | 0 |
| RP1-39G22.5 | 0 | 0 | 0 | 0 | 1 | 0 |
| RP1-4G17.5 | 0 | 0 | 0 | 0 | 1 | 0 |
| RP1-5O6.4 | 0 | 0 | 0 | 0 | 1 | 0 |
| RP1-8B22.1 | 0 | 0 | 0 | 0 | 1 | 0 |
| RP1-95L4.3 | 0 | 0 | 0 | 0 | 1 | 0 |
| RP2 | 0 | 0 | 0 | 0 | 1 | 0 |
| RP3-408N23.4 | 0 | 0 | 0 | 0 | 1 | 0 |
| RP3-449O17.1 | 0 | 0 | 0 | 0 | 1 | 0 |
| RP4-545K15.3 | 0 | 0 | 0 | 0 | 1 | 0 |
| RP4-564F22.2 | 0 | 0 | 0 | 0 | 1 | 0 |
| RP4-630A11.3 | 0 | 0 | 0 | 0 | 1 | 0 |
| RP4-639F20.3 | 0 | 0 | 0 | 0 | 1 | 0 |
| RP4-655L22.2 | 0 | 0 | 0 | 0 | 1 | 0 |
| RP4-655L22.4 | 0 | 0 | 0 | 0 | 1 | 0 |
| RP4-687K1.2 | 0 | 0 | 0 | 0 | 1 | 0 |
| RP4-740C4.5 | 0 | 0 | 0 | 0 | 1 | 0 |
| RP4-761J14.8 | 0 | 0 | 0 | 0 | 1 | 0 |
| RP5-1000K24.2 | 0 | 0 | 0 | 0 | 1 | 0 |
| RP5-1022P6.2 | 0 | 0 | 0 | 1 | 0 | 0 |
| RP5-1154L15.2 | 0 | 0 | 0 | 0 | 1 | 0 |
| RP5-1165K10.1 | 1 | 0 | 0 | 0 | 0 | 0 |
| RP5-892G5.2 | 0 | 0 | 0 | 0 | 1 | 0 |
| RP5-916O11.2 | 0 | 0 | 0 | 0 | 1 | 0 |
| RP6-149D17.1 | 0 | 0 | 0 | 0 | 1 | 0 |
| RP6-213H19.1 | 0 | 0 | 0 | 1 | 0 | 0 |
| RPA2 | 0 | 0 | 0 | 1 | 0 | 0 |
| RPAIN | 1 | 0 | 1 | 0 | 0 | 0 |
| RPE | 0 | 0 | 1 | 0 | 1 | 0 |
| RPIA | 0 | 0 | 0 | 1 | 0 | 0 |
| RPL10 | 1 | 0 | 0 | 0 | 0 | 0 |
| RPL10A | 1 | 0 | 0 | 0 | 0 | 0 |
| RPL11 | 1 | 0 | 0 | 0 | 0 | 0 |
| RPL12 | 1 | 0 | 1 | 0 | 0 | 0 |
| RPL13 | 1 | 0 | 0 | 1 | 1 | 0 |
| RPL13A | 1 | 0 | 1 | 0 | 0 | 0 |
| RPL14 | 1 | 0 | 0 | 0 | 0 | 0 |
| RPL15 | 1 | 0 | 0 | 0 | 0 | 0 |
| RPL17 | 1 | 0 | 0 | 0 | 0 | 0 |
| RPL17-C18orf32 | 1 | 0 | 0 | 0 | 1 | 0 |
| RPL18 | 1 | 0 | 0 | 0 | 0 | 0 |
| RPL18A | 1 | 0 | 0 | 1 | 0 | 0 |
| RPL19 | 1 | 0 | 0 | 0 | 0 | 0 |
| RPL21 | 1 | 0 | 0 | 0 | 0 | 0 |
| RPL21P10 | 0 | 0 | 0 | 0 | 1 | 0 |
| RPL22 | 1 | 0 | 0 | 0 | 1 | 0 |
| RPL23 | 1 | 0 | 0 | 0 | 0 | 0 |
| RPL23A | 1 | 0 | 0 | 0 | 0 | 0 |
| RPL23AP53 | 0 | 0 | 0 | 0 | 1 | 0 |
| RPL24 | 1 | 0 | 0 | 0 | 0 | 0 |
| RPL26 | 1 | 0 | 0 | 0 | 0 | 0 |
| RPL26L1 | 0 | 0 | 0 | 0 | 1 | 0 |
| RPL27 | 1 | 0 | 0 | 0 | 0 | 0 |
| RPL27A | 1 | 0 | 0 | 0 | 1 | 0 |
| RPL28 | 0 | 0 | 0 | 0 | 1 | 0 |
| RPL29 | 1 | 0 | 0 | 0 | 0 | 0 |
| RPL3 | 1 | 0 | 0 | 0 | 0 | 0 |
| RPL30 | 1 | 0 | 0 | 0 | 0 | 0 |
| RPL31 | 1 | 0 | 0 | 1 | 0 | 0 |
| RPL32 | 1 | 0 | 0 | 0 | 0 | 0 |
| RPL34 | 1 | 0 | 0 | 0 | 0 | 0 |
| RPL35 | 1 | 0 | 0 | 0 | 0 | 0 |
| RPL35A | 1 | 0 | 0 | 1 | 0 | 0 |
| RPL36 | 1 | 0 | 0 | 0 | 0 | 0 |
| RPL36A | 1 | 0 | 0 | 0 | 0 | 0 |
| RPL36AL | 1 | 0 | 0 | 0 | 0 | 0 |
| RPL37 | 1 | 0 | 0 | 0 | 0 | 0 |
| RPL37A | 1 | 0 | 0 | 0 | 0 | 0 |
| RPL38 | 1 | 0 | 0 | 0 | 0 | 0 |
| RPL39 | 1 | 0 | 0 | 0 | 0 | 0 |
| RPL39L | 1 | 0 | 0 | 0 | 0 | 0 |
| RPL4 | 1 | 0 | 0 | 0 | 0 | 0 |
| RPL41 | 1 | 0 | 0 | 0 | 0 | 0 |
| RPL5 | 1 | 0 | 1 | 0 | 0 | 0 |
| RPL6 | 1 | 0 | 0 | 0 | 0 | 0 |
| RPL7 | 1 | 0 | 0 | 0 | 0 | 0 |
| RPL7A | 1 | 0 | 0 | 0 | 0 | 0 |
| RPL7L1P8 | 0 | 0 | 0 | 0 | 1 | 0 |
| RPL8 | 1 | 0 | 0 | 0 | 0 | 0 |
| RPL9 | 1 | 0 | 0 | 0 | 0 | 0 |
| RPLP0 | 1 | 0 | 0 | 1 | 0 | 0 |
| RPLP1 | 1 | 0 | 0 | 0 | 0 | 0 |
| RPLP2 | 1 | 0 | 0 | 0 | 0 | 0 |
| RPN1 | 1 | 0 | 0 | 0 | 0 | 0 |
| RPN2 | 1 | 0 | 0 | 1 | 0 | 0 |
| RPP14 | 1 | 0 | 0 | 0 | 0 | 0 |
| RPPH1 | 0 | 0 | 1 | 0 | 0 | 0 |
| RPRD1B | 1 | 0 | 0 | 0 | 0 | 0 |
| RPS10 | 1 | 0 | 0 | 1 | 0 | 0 |
| RPS11 | 1 | 0 | 0 | 0 | 0 | 0 |
| RPS12 | 1 | 0 | 0 | 0 | 0 | 0 |
| RPS13 | 1 | 0 | 0 | 0 | 0 | 0 |
| RPS14 | 1 | 0 | 0 | 0 | 1 | 0 |
| RPS15 | 1 | 0 | 0 | 0 | 0 | 0 |
| RPS15A | 1 | 0 | 0 | 0 | 0 | 0 |
| RPS17 | 1 | 0 | 0 | 0 | 0 | 0 |
| RPS17L | 1 | 0 | 0 | 0 | 0 | 0 |
| RPS18 | 1 | 0 | 0 | 0 | 0 | 0 |
| RPS19 | 1 | 0 | 0 | 0 | 0 | 0 |
| RPS2 | 1 | 0 | 0 | 0 | 0 | 0 |
| RPS20 | 1 | 0 | 0 | 0 | 0 | 0 |
| RPS20P21 | 0 | 0 | 0 | 0 | 1 | 0 |
| RPS21 | 1 | 0 | 0 | 0 | 0 | 0 |
| RPS23 | 1 | 0 | 0 | 1 | 1 | 0 |
| RPS24 | 1 | 0 | 0 | 0 | 0 | 0 |
| RPS25 | 1 | 0 | 0 | 0 | 0 | 0 |
| RPS26 | 1 | 0 | 0 | 0 | 0 | 0 |
| RPS27 | 1 | 0 | 0 | 0 | 0 | 0 |
| RPS27A | 1 | 0 | 0 | 0 | 0 | 0 |
| RPS27L | 1 | 0 | 0 | 1 | 0 | 0 |
| RPS28 | 1 | 0 | 0 | 0 | 0 | 0 |
| RPS29 | 1 | 0 | 0 | 0 | 0 | 0 |
| RPS29P20 | 0 | 0 | 0 | 0 | 1 | 0 |
| RPS3 | 1 | 0 | 0 | 0 | 0 | 0 |
| RPS3A | 1 | 0 | 0 | 0 | 0 | 0 |
| RPS4X | 1 | 0 | 0 | 0 | 0 | 0 |
| RPS5 | 1 | 0 | 0 | 0 | 0 | 0 |
| RPS6 | 1 | 0 | 0 | 0 | 0 | 0 |
| RPS6KA3 | 1 | 0 | 0 | 1 | 0 | 0 |
| RPS7 | 1 | 0 | 0 | 0 | 0 | 0 |
| RPS8 | 1 | 0 | 1 | 0 | 1 | 0 |
| RPS9 | 1 | 0 | 0 | 1 | 0 | 0 |
| RPSA | 1 | 0 | 1 | 0 | 0 | 0 |
| RPSAP58 | 1 | 0 | 0 | 0 | 0 | 0 |
| RPTOR | 0 | 0 | 1 | 0 | 0 | 0 |
| RQCD1 | 1 | 0 | 0 | 1 | 0 | 0 |
| RRAGA | 1 | 0 | 0 | 0 | 1 | 0 |
| RRAGB | 0 | 0 | 0 | 0 | 1 | 0 |
| RRAGC | 1 | 0 | 0 | 0 | 0 | 0 |
| RREB1 | 0 | 1 | 1 | 0 | 0 | 0 |
| RRM2 | 1 | 0 | 0 | 0 | 1 | 0 |
| RRM2P3 | 0 | 0 | 0 | 0 | 1 | 0 |
| RRP1 | 0 | 1 | 0 | 0 | 0 | 0 |
| RRP15 | 1 | 0 | 0 | 0 | 0 | 0 |
| RSF1 | 1 | 0 | 0 | 0 | 0 | 0 |
| RSL24D1 | 1 | 0 | 0 | 0 | 0 | 0 |
| RSRC1 | 0 | 0 | 1 | 0 | 0 | 0 |
| RTDR1 | 0 | 1 | 0 | 0 | 0 | 0 |
| RTN3 | 0 | 0 | 0 | 0 | 1 | 0 |
| RTN3P1 | 0 | 0 | 0 | 0 | 1 | 0 |
| RTN4 | 1 | 0 | 1 | 1 | 0 | 0 |
| RTN4RL2 | 0 | 0 | 1 | 0 | 0 | 0 |
| RTP2 | 0 | 0 | 0 | 0 | 1 | 0 |
| RTP3 | 0 | 1 | 0 | 0 | 0 | 0 |
| RTTN | 0 | 0 | 1 | 0 | 0 | 0 |
| RUFY3 | 0 | 0 | 1 | 0 | 0 | 0 |
| RUNDC2B | 0 | 1 | 0 | 0 | 0 | 0 |
| RUNX1 | 1 | 0 | 0 | 0 | 0 | 0 |
| RUNX1T1 | 0 | 1 | 0 | 0 | 0 | 0 |
| RUSC2 | 0 | 0 | 1 | 0 | 0 | 0 |
| RUVBL1 | 1 | 0 | 0 | 0 | 0 | 0 |
| RWDD1 | 1 | 0 | 0 | 0 | 0 | 0 |
| RWDD4A | 0 | 0 | 0 | 1 | 0 | 0 |
| RXRA | 0 | 0 | 1 | 0 | 1 | 0 |
| RXRB | 0 | 0 | 1 | 0 | 0 | 0 |
| RYBP | 1 | 0 | 0 | 1 | 0 | 0 |
| RYK | 1 | 0 | 0 | 1 | 0 | 0 |
| RYR2 | 0 | 0 | 1 | 0 | 0 | 0 |
| S100A10 | 1 | 0 | 0 | 0 | 0 | 0 |
| S100A11 | 0 | 0 | 1 | 0 | 0 | 0 |
| S100A13 | 1 | 0 | 0 | 0 | 0 | 0 |
| S100A16 | 0 | 0 | 0 | 0 | 1 | 0 |
| S100A6 | 1 | 0 | 0 | 0 | 0 | 0 |
| SAAL1 | 0 | 0 | 0 | 0 | 1 | 0 |
| SAE1 | 1 | 0 | 0 | 0 | 1 | 0 |
| SAGE1 | 0 | 0 | 0 | 0 | 1 | 0 |
| SALL4 | 0 | 1 | 0 | 0 | 0 | 0 |
| SAMD1 | 1 | 0 | 1 | 0 | 0 | 0 |
| SAMD11 | 1 | 0 | 0 | 0 | 0 | 0 |
| SAMD4A | 1 | 0 | 0 | 0 | 0 | 0 |
| SAMD9 | 0 | 0 | 0 | 1 | 0 | 0 |
| SAMD9L | 0 | 0 | 0 | 1 | 0 | 0 |
| SAMSN1 | 1 | 0 | 0 | 1 | 0 | 0 |
| SAP18 | 1 | 0 | 1 | 0 | 1 | 0 |
| SARNP | 1 | 0 | 0 | 0 | 0 | 0 |
| SARS | 1 | 0 | 0 | 0 | 0 | 0 |
| SART3 | 1 | 0 | 0 | 0 | 0 | 0 |
| SAT1 | 0 | 0 | 0 | 1 | 0 | 0 |
| SATB2 | 1 | 0 | 0 | 0 | 0 | 0 |
| SAV1 | 0 | 0 | 0 | 1 | 0 | 0 |
| SAYSD1 | 0 | 0 | 0 | 0 | 1 | 0 |
| SBDS | 0 | 0 | 0 | 0 | 1 | 0 |
| SBF1 | 0 | 0 | 1 | 0 | 0 | 0 |
| SBF2 | 0 | 0 | 1 | 0 | 0 | 0 |
| SBNO1 | 1 | 0 | 0 | 0 | 0 | 0 |
| SCAMP1 | 0 | 0 | 0 | 0 | 1 | 0 |
| SCAMP2 | 0 | 0 | 0 | 0 | 1 | 0 |
| SCAMP3 | 0 | 0 | 0 | 0 | 1 | 0 |
| SCAMP4 | 0 | 0 | 0 | 0 | 1 | 0 |
| SCAND2 | 0 | 0 | 0 | 0 | 1 | 0 |
| SCAND3 | 0 | 0 | 0 | 0 | 1 | 0 |
| SCARB2 | 0 | 0 | 0 | 0 | 1 | 0 |
| SCARNA15 | 0 | 0 | 0 | 1 | 0 | 0 |
| SCARNA2 | 0 | 0 | 1 | 0 | 0 | 0 |
| SCARNA23 | 0 | 0 | 0 | 0 | 1 | 0 |
| SCARNA7 | 0 | 0 | 1 | 0 | 0 | 1 |
| SCARNA9 | 0 | 0 | 1 | 0 | 1 | 1 |
| SCD | 1 | 0 | 1 | 0 | 0 | 0 |
| SCD5 | 1 | 0 | 0 | 0 | 0 | 0 |
| SCN9A | 0 | 0 | 0 | 0 | 1 | 0 |
| SCOC | 1 | 0 | 0 | 1 | 1 | 0 |
| SDAD1 | 0 | 0 | 0 | 0 | 1 | 0 |
| SDC2 | 0 | 0 | 1 | 0 | 0 | 0 |
| SDC4 | 1 | 0 | 1 | 0 | 1 | 0 |
| SDCBP | 1 | 0 | 0 | 1 | 0 | 0 |
| SDE2 | 1 | 0 | 0 | 0 | 0 | 0 |
| SDHB | 1 | 0 | 0 | 0 | 0 | 0 |
| SDHC | 0 | 0 | 0 | 0 | 1 | 0 |
| SDHD | 0 | 0 | 0 | 0 | 1 | 0 |
| SDK2 | 1 | 0 | 0 | 0 | 0 | 0 |
| SDPR | 0 | 1 | 0 | 0 | 0 | 0 |
| SEC14L1 | 1 | 0 | 0 | 0 | 1 | 0 |
| SEC16A | 0 | 0 | 1 | 0 | 0 | 0 |
| SEC16B | 0 | 1 | 0 | 0 | 0 | 0 |
| SEC23B | 1 | 0 | 0 | 1 | 1 | 0 |
| SEC23IP | 0 | 0 | 0 | 0 | 1 | 0 |
| SEC24A | 1 | 0 | 0 | 1 | 0 | 0 |
| SEC31A | 0 | 0 | 0 | 1 | 0 | 0 |
| SEC61A1 | 1 | 0 | 0 | 0 | 1 | 0 |
| SEC61A2 | 0 | 0 | 0 | 0 | 1 | 1 |
| SEC61B | 1 | 0 | 0 | 0 | 1 | 0 |
| SEC61G | 1 | 0 | 0 | 0 | 0 | 0 |
| SEC63 | 0 | 0 | 1 | 0 | 0 | 0 |
| SEH1L | 1 | 0 | 1 | 1 | 1 | 0 |
| SEL1L | 1 | 0 | 1 | 0 | 0 | 0 |
| SEL1L2 | 0 | 1 | 0 | 0 | 0 | 0 |
| SELI | 0 | 0 | 0 | 1 | 0 | 0 |
| SELT | 1 | 0 | 0 | 0 | 1 | 0 |
| SEMA4B | 0 | 0 | 1 | 0 | 0 | 0 |
| SEMA4C | 0 | 0 | 0 | 0 | 1 | 0 |
| SEMA6B | 0 | 0 | 0 | 0 | 1 | 0 |
| SENP3 | 0 | 0 | 0 | 0 | 1 | 0 |
| SENP3-EIF4A1 | 0 | 0 | 0 | 0 | 1 | 0 |
| SENP7 | 0 | 0 | 1 | 1 | 0 | 0 |
| SEP15 | 1 | 0 | 0 | 0 | 1 | 0 |
| SEPT11 | 1 | 0 | 0 | 0 | 0 | 0 |
| SEPT2 | 1 | 0 | 0 | 0 | 1 | 0 |
| SEPT7 | 1 | 0 | 0 | 1 | 0 | 0 |
| SEPT8 | 1 | 0 | 0 | 0 | 0 | 0 |
| SEPT9 | 1 | 0 | 1 | 0 | 0 | 0 |
| SEPW1 | 1 | 0 | 0 | 0 | 1 | 0 |
| SERBP1 | 1 | 0 | 0 | 1 | 1 | 0 |
| SERF1A | 0 | 0 | 0 | 0 | 1 | 0 |
| SERF1B | 0 | 0 | 0 | 0 | 1 | 0 |
| SERF2 | 1 | 0 | 1 | 0 | 0 | 0 |
| SERINC1 | 1 | 0 | 1 | 0 | 0 | 1 |
| SERINC3 | 0 | 0 | 0 | 0 | 1 | 0 |
| SERINC5 | 0 | 0 | 0 | 0 | 1 | 0 |
| SERP1 | 1 | 0 | 0 | 1 | 1 | 0 |
| SERPINB13 | 0 | 0 | 0 | 0 | 1 | 0 |
| SERPINB6 | 0 | 0 | 1 | 0 | 0 | 0 |
| SERPINB9 | 0 | 0 | 0 | 1 | 1 | 0 |
| SERPINE2 | 1 | 0 | 0 | 1 | 0 | 0 |
| SERPINI1 | 0 | 0 | 0 | 0 | 1 | 0 |
| SERTAD2 | 1 | 0 | 0 | 0 | 1 | 0 |
| SERTAD3 | 0 | 0 | 0 | 0 | 1 | 0 |
| SESN1 | 0 | 0 | 0 | 0 | 1 | 0 |
| SET | 1 | 0 | 1 | 1 | 0 | 0 |
| SETD2 | 0 | 0 | 0 | 1 | 0 | 0 |
| SETD5 | 0 | 0 | 1 | 0 | 0 | 0 |
| SETD7 | 1 | 0 | 1 | 0 | 0 | 0 |
| SETP6 | 0 | 0 | 0 | 0 | 1 | 0 |
| SEZ6 | 1 | 0 | 0 | 0 | 0 | 0 |
| SF1 | 1 | 0 | 1 | 1 | 0 | 0 |
| SF3A3 | 1 | 0 | 0 | 0 | 0 | 0 |
| SF3B1 | 1 | 0 | 0 | 1 | 0 | 0 |
| SF3B14 | 1 | 0 | 0 | 0 | 0 | 0 |
| SF3B3 | 0 | 0 | 1 | 1 | 0 | 0 |
| SFMBT2 | 0 | 0 | 1 | 0 | 0 | 0 |
| SFPQ | 1 | 0 | 0 | 1 | 0 | 0 |
| SFRP4 | 1 | 0 | 0 | 0 | 0 | 0 |
| SFRS1 | 0 | 0 | 0 | 1 | 0 | 0 |
| SFRS18 | 0 | 0 | 0 | 1 | 0 | 0 |
| SFRS2 | 0 | 0 | 0 | 1 | 0 | 0 |
| SFRS2B | 0 | 0 | 0 | 1 | 0 | 0 |
| SFRS3 | 0 | 0 | 0 | 1 | 0 | 0 |
| SFRS5 | 0 | 0 | 0 | 1 | 0 | 0 |
| SFRS6 | 0 | 0 | 0 | 1 | 0 | 0 |
| SFT2D1 | 1 | 1 | 0 | 1 | 0 | 0 |
| SFT2D2 | 1 | 0 | 1 | 0 | 1 | 0 |
| SFXN1 | 0 | 0 | 0 | 1 | 1 | 0 |
| SGK1 | 1 | 0 | 1 | 0 | 0 | 0 |
| SGK196 | 1 | 0 | 0 | 0 | 1 | 0 |
| SGOL1 | 1 | 0 | 0 | 0 | 0 | 0 |
| SGPL1 | 1 | 0 | 0 | 0 | 1 | 0 |
| SGPP1 | 0 | 0 | 0 | 0 | 1 | 0 |
| SH2B1 | 0 | 0 | 0 | 0 | 1 | 0 |
| SH3BGR | 0 | 0 | 0 | 0 | 1 | 0 |
| SH3BGRL3 | 0 | 0 | 0 | 1 | 0 | 0 |
| SH3BP1 | 0 | 0 | 0 | 0 | 1 | 0 |
| SH3BP2 | 0 | 0 | 1 | 0 | 0 | 0 |
| SH3D19 | 0 | 0 | 1 | 0 | 0 | 0 |
| SH3GL3 | 0 | 0 | 0 | 0 | 1 | 0 |
| SH3RF1 | 0 | 0 | 1 | 0 | 0 | 0 |
| SH3TC1 | 0 | 1 | 0 | 0 | 0 | 0 |
| SHANK3 | 1 | 0 | 1 | 0 | 0 | 0 |
| SHB | 0 | 0 | 0 | 1 | 1 | 0 |
| SHC1 | 0 | 0 | 0 | 1 | 0 | 0 |
| SHC4 | 1 | 0 | 0 | 0 | 0 | 0 |
| SHISA5 | 1 | 0 | 1 | 0 | 0 | 0 |
| SHISA9 | 1 | 0 | 0 | 0 | 0 | 0 |
| SHOC2 | 1 | 0 | 0 | 1 | 0 | 0 |
| SHQ1 | 1 | 0 | 0 | 0 | 0 | 0 |
| SIAE | 1 | 0 | 0 | 0 | 0 | 0 |
| SIAH1 | 1 | 0 | 0 | 0 | 1 | 0 |
| SIAH1P1 | 0 | 0 | 0 | 0 | 1 | 0 |
| SIAH2 | 1 | 0 | 0 | 0 | 1 | 0 |
| SIGLEC11 | 0 | 0 | 0 | 0 | 1 | 0 |
| SIGMAR1 | 0 | 0 | 1 | 0 | 0 | 0 |
| SIPA1L1 | 0 | 0 | 1 | 0 | 0 | 0 |
| SIRT6 | 0 | 0 | 0 | 0 | 1 | 0 |
| SIRT7 | 0 | 0 | 1 | 0 | 1 | 0 |
| SKA2 | 1 | 0 | 0 | 0 | 0 | 0 |
| SKAP1 | 0 | 0 | 0 | 1 | 0 | 0 |
| SKI | 0 | 0 | 1 | 0 | 0 | 0 |
| SKIL | 1 | 1 | 1 | 0 | 1 | 0 |
| SKP1 | 1 | 0 | 0 | 0 | 1 | 0 |
| SKP1P1 | 0 | 0 | 0 | 0 | 1 | 0 |
| SLA2 | 1 | 0 | 0 | 0 | 0 | 0 |
| SLAIN2 | 0 | 0 | 0 | 1 | 0 | 0 |
| SLC10A4 | 0 | 0 | 0 | 0 | 1 | 0 |
| SLC11A2 | 0 | 0 | 0 | 0 | 1 | 0 |
| SLC12A2 | 0 | 0 | 0 | 0 | 1 | 0 |
| SLC12A5 | 0 | 0 | 0 | 0 | 1 | 0 |
| SLC12A6 | 0 | 0 | 0 | 1 | 0 | 0 |
| SLC15A3 | 0 | 1 | 0 | 0 | 0 | 0 |
| SLC15A4 | 0 | 0 | 0 | 0 | 1 | 0 |
| SLC16A1 | 1 | 0 | 1 | 1 | 0 | 0 |
| SLC17A5 | 0 | 0 | 0 | 0 | 1 | 0 |
| SLC17A7 | 0 | 0 | 0 | 0 | 1 | 0 |
| SLC18A2 | 1 | 0 | 0 | 0 | 0 | 0 |
| SLC18B1 | 0 | 0 | 1 | 0 | 1 | 0 |
| SLC19A2 | 0 | 0 | 0 | 0 | 1 | 0 |
| SLC1A2 | 0 | 1 | 0 | 0 | 0 | 0 |
| SLC1A3 | 0 | 0 | 1 | 0 | 0 | 0 |
| SLC1A4 | 0 | 0 | 0 | 0 | 1 | 0 |
| SLC1A5 | 1 | 0 | 1 | 0 | 0 | 0 |
| SLC20A1 | 1 | 0 | 0 | 0 | 0 | 0 |
| SLC20A2 | 1 | 0 | 0 | 0 | 1 | 0 |
| SLC22A23 | 0 | 0 | 0 | 0 | 1 | 0 |
| SLC22A5 | 0 | 0 | 0 | 0 | 1 | 0 |
| SLC23A2 | 0 | 0 | 0 | 0 | 1 | 0 |
| SLC24A1 | 0 | 0 | 0 | 0 | 1 | 0 |
| SLC24A6 | 0 | 0 | 0 | 0 | 1 | 0 |
| SLC25A1 | 1 | 0 | 1 | 0 | 0 | 0 |
| SLC25A12 | 1 | 0 | 0 | 0 | 1 | 0 |
| SLC25A13 | 0 | 0 | 1 | 0 | 0 | 0 |
| SLC25A14 | 0 | 0 | 0 | 0 | 1 | 0 |
| SLC25A15 | 0 | 0 | 0 | 0 | 1 | 0 |
| SLC25A17 | 0 | 0 | 1 | 0 | 1 | 0 |
| SLC25A22 | 0 | 0 | 0 | 0 | 1 | 0 |
| SLC25A24 | 1 | 0 | 0 | 0 | 1 | 0 |
| SLC25A26 | 0 | 0 | 0 | 0 | 1 | 0 |
| SLC25A3 | 1 | 0 | 0 | 0 | 0 | 0 |
| SLC25A30 | 0 | 1 | 0 | 0 | 1 | 0 |
| SLC25A33 | 0 | 0 | 0 | 0 | 1 | 0 |
| SLC25A36 | 0 | 1 | 0 | 0 | 0 | 0 |
| SLC25A37 | 0 | 1 | 0 | 0 | 0 | 0 |
| SLC25A39 | 1 | 0 | 1 | 0 | 1 | 0 |
| SLC25A4 | 0 | 0 | 0 | 0 | 1 | 0 |
| SLC25A40 | 0 | 0 | 1 | 0 | 0 | 0 |
| SLC25A44 | 1 | 0 | 0 | 1 | 1 | 0 |
| SLC25A46 | 1 | 0 | 0 | 1 | 0 | 0 |
| SLC25A5 | 1 | 0 | 0 | 0 | 0 | 0 |
| SLC25A51 | 0 | 0 | 0 | 0 | 1 | 0 |
| SLC25A53 | 1 | 0 | 0 | 0 | 0 | 0 |
| SLC25A6 | 0 | 0 | 0 | 1 | 0 | 0 |
| SLC26A2 | 1 | 0 | 0 | 0 | 0 | 0 |
| SLC26A5 | 0 | 0 | 0 | 0 | 1 | 0 |
| SLC26A6 | 0 | 0 | 1 | 0 | 0 | 0 |
| SLC27A5 | 0 | 0 | 0 | 0 | 1 | 0 |
| SLC29A1 | 1 | 0 | 1 | 0 | 1 | 0 |
| SLC2A3 | 0 | 0 | 0 | 1 | 0 | 0 |
| SLC30A1 | 0 | 0 | 0 | 1 | 1 | 0 |
| SLC30A3 | 0 | 0 | 0 | 0 | 1 | 0 |
| SLC30A5 | 1 | 0 | 0 | 0 | 1 | 0 |
| SLC30A6 | 0 | 0 | 0 | 0 | 1 | 0 |
| SLC30A7 | 1 | 0 | 1 | 0 | 1 | 0 |
| SLC31A1 | 0 | 0 | 1 | 0 | 0 | 0 |
| SLC35A1 | 0 | 0 | 0 | 1 | 1 | 0 |
| SLC35A2 | 0 | 0 | 0 | 0 | 1 | 0 |
| SLC35A3 | 0 | 0 | 1 | 1 | 1 | 0 |
| SLC35A5 | 0 | 0 | 0 | 0 | 1 | 0 |
| SLC35B1 | 0 | 0 | 0 | 1 | 0 | 0 |
| SLC35B3 | 0 | 0 | 0 | 0 | 1 | 0 |
| SLC35B4 | 1 | 0 | 1 | 0 | 1 | 0 |
| SLC35C2 | 0 | 0 | 0 | 0 | 1 | 0 |
| SLC35E1 | 1 | 0 | 0 | 1 | 1 | 0 |
| SLC35E3 | 0 | 0 | 0 | 0 | 1 | 0 |
| SLC35F2 | 1 | 0 | 0 | 0 | 0 | 0 |
| SLC35F5 | 0 | 0 | 0 | 0 | 1 | 0 |
| SLC35G1 | 0 | 0 | 0 | 0 | 1 | 0 |
| SLC36A4 | 0 | 0 | 0 | 0 | 1 | 0 |
| SLC37A3 | 0 | 0 | 0 | 0 | 1 | 0 |
| SLC38A1 | 0 | 0 | 0 | 0 | 1 | 0 |
| SLC38A2 | 1 | 0 | 1 | 1 | 0 | 0 |
| SLC38A5 | 0 | 0 | 1 | 0 | 0 | 0 |
| SLC38A9 | 0 | 0 | 0 | 0 | 1 | 1 |
| SLC39A10 | 1 | 0 | 1 | 1 | 0 | 0 |
| SLC39A14 | 1 | 0 | 0 | 0 | 1 | 0 |
| SLC39A3 | 0 | 0 | 0 | 0 | 1 | 0 |
| SLC39A6 | 0 | 0 | 0 | 0 | 1 | 0 |
| SLC39A9 | 1 | 0 | 1 | 0 | 1 | 0 |
| SLC3A1 | 1 | 0 | 0 | 0 | 0 | 0 |
| SLC40A1 | 0 | 0 | 0 | 0 | 1 | 0 |
| SLC41A1 | 0 | 1 | 0 | 0 | 0 | 0 |
| SLC41A3 | 0 | 0 | 0 | 0 | 1 | 0 |
| SLC43A2 | 0 | 0 | 0 | 0 | 1 | 0 |
| SLC44A1 | 1 | 0 | 0 | 0 | 0 | 0 |
| SLC44A2 | 0 | 0 | 0 | 1 | 0 | 0 |
| SLC45A4 | 0 | 0 | 0 | 0 | 1 | 0 |
| SLC46A3 | 0 | 0 | 0 | 0 | 1 | 0 |
| SLC4A11 | 0 | 1 | 0 | 0 | 0 | 0 |
| SLC4A5 | 1 | 0 | 0 | 0 | 0 | 0 |
| SLC4A7 | 1 | 0 | 1 | 0 | 1 | 0 |
| SLC51A | 1 | 0 | 0 | 0 | 0 | 0 |
| SLC52A2 | 0 | 0 | 0 | 0 | 1 | 0 |
| SLC5A3 | 1 | 0 | 0 | 0 | 0 | 0 |
| SLC5A6 | 0 | 0 | 0 | 0 | 1 | 0 |
| SLC6A6 | 1 | 0 | 1 | 0 | 0 | 0 |
| SLC6A9 | 0 | 0 | 1 | 0 | 0 | 0 |
| SLC7A1 | 0 | 0 | 1 | 0 | 1 | 0 |
| SLC7A11 | 0 | 0 | 1 | 0 | 0 | 0 |
| SLC7A5 | 0 | 0 | 1 | 0 | 0 | 0 |
| SLC7A6 | 0 | 0 | 1 | 1 | 1 | 0 |
| SLC7A6OS | 0 | 1 | 0 | 0 | 0 | 0 |
| SLC9A2 | 0 | 0 | 0 | 0 | 1 | 0 |
| SLC9A6 | 1 | 0 | 0 | 0 | 1 | 0 |
| SLC9B1 | 1 | 0 | 0 | 0 | 0 | 0 |
| SLC9B2 | 0 | 0 | 0 | 0 | 1 | 0 |
| SLCO4C1 | 0 | 0 | 0 | 0 | 1 | 0 |
| SLCO5A1 | 1 | 0 | 1 | 0 | 0 | 0 |
| SLIRP | 1 | 0 | 0 | 0 | 0 | 0 |
| SLMAP | 0 | 1 | 1 | 0 | 0 | 0 |
| SLMO2 | 1 | 0 | 1 | 1 | 0 | 0 |
| SMAD2 | 1 | 0 | 1 | 1 | 1 | 0 |
| SMAD4 | 0 | 0 | 1 | 0 | 1 | 0 |
| SMAD5 | 1 | 0 | 0 | 0 | 0 | 0 |
| SMAP1 | 0 | 0 | 0 | 1 | 0 | 0 |
| SMAP2 | 1 | 0 | 0 | 1 | 0 | 0 |
| SMARCA2 | 0 | 0 | 1 | 0 | 0 | 0 |
| SMARCAD1 | 1 | 0 | 0 | 0 | 0 | 0 |
| SMARCB1 | 0 | 0 | 1 | 0 | 0 | 0 |
| SMARCC1 | 1 | 0 | 1 | 0 | 0 | 0 |
| SMARCD1 | 0 | 1 | 0 | 0 | 0 | 0 |
| SMARCE1 | 1 | 0 | 0 | 0 | 1 | 0 |
| SMC1A | 1 | 0 | 1 | 0 | 0 | 0 |
| SMC3 | 0 | 0 | 0 | 0 | 1 | 0 |
| SMC4 | 0 | 0 | 0 | 0 | 1 | 0 |
| SMC5 | 1 | 0 | 1 | 1 | 0 | 0 |
| SMCHD1 | 0 | 0 | 1 | 1 | 0 | 0 |
| SMCR7L | 1 | 0 | 0 | 1 | 1 | 0 |
| SMEK2 | 1 | 1 | 0 | 1 | 0 | 0 |
| SMG1 | 1 | 0 | 0 | 0 | 0 | 0 |
| SMG6 | 0 | 0 | 1 | 0 | 0 | 0 |
| SMG7 | 1 | 0 | 1 | 0 | 0 | 0 |
| SMIM13 | 1 | 0 | 0 | 0 | 0 | 0 |
| SMIM15 | 1 | 0 | 0 | 0 | 0 | 0 |
| SMIM7 | 1 | 0 | 0 | 0 | 1 | 0 |
| SMN1 | 0 | 0 | 0 | 1 | 0 | 0 |
| SMNDC1 | 1 | 0 | 0 | 1 | 1 | 0 |
| SMOC1 | 0 | 0 | 1 | 0 | 0 | 0 |
| SMOX | 0 | 1 | 0 | 0 | 0 | 0 |
| SMS | 1 | 0 | 1 | 1 | 0 | 0 |
| SMYD3 | 0 | 0 | 1 | 0 | 0 | 0 |
| SNAI3-AS1 | 0 | 0 | 1 | 0 | 0 | 0 |
| SNAP23 | 1 | 0 | 0 | 1 | 0 | 0 |
| SNAP25 | 0 | 0 | 1 | 0 | 0 | 0 |
| SNAPC3 | 0 | 0 | 1 | 0 | 0 | 0 |
| SNCG | 0 | 0 | 1 | 0 | 0 | 0 |
| SND1 | 0 | 0 | 1 | 0 | 0 | 0 |
| SNED1 | 0 | 1 | 0 | 0 | 0 | 0 |
| SNHG1 | 0 | 0 | 0 | 1 | 1 | 0 |
| SNHG14 | 0 | 0 | 0 | 0 | 0 | 1 |
| SNHG17 | 0 | 0 | 1 | 0 | 0 | 0 |
| SNHG3 | 0 | 1 | 1 | 0 | 0 | 0 |
| SNHG5 | 0 | 0 | 0 | 0 | 1 | 0 |
| SNORA25 | 0 | 0 | 0 | 0 | 1 | 0 |
| SNORA2A | 0 | 0 | 0 | 0 | 1 | 1 |
| SNORA31 | 0 | 0 | 0 | 0 | 1 | 0 |
| SNORA32 | 0 | 0 | 0 | 0 | 0 | 1 |
| SNORA4 | 0 | 0 | 1 | 0 | 0 | 0 |
| SNORA43 | 0 | 0 | 0 | 0 | 1 | 1 |
| SNORA45 | 0 | 0 | 0 | 0 | 1 | 0 |
| SNORA46 | 0 | 0 | 0 | 0 | 1 | 0 |
| SNORA51 | 0 | 0 | 0 | 0 | 1 | 0 |
| SNORA58 | 0 | 0 | 0 | 0 | 1 | 0 |
| SNORA6 | 0 | 0 | 1 | 0 | 0 | 0 |
| SNORA62 | 0 | 0 | 1 | 0 | 0 | 0 |
| SNORA63 | 0 | 0 | 0 | 0 | 1 | 0 |
| SNORA65 | 0 | 0 | 1 | 0 | 0 | 0 |
| SNORA7 | 0 | 0 | 0 | 0 | 1 | 0 |
| SNORA70G | 0 | 0 | 0 | 0 | 0 | 1 |
| SNORA71 | 0 | 0 | 0 | 0 | 1 | 0 |
| SNORA71A | 0 | 0 | 0 | 0 | 1 | 0 |
| SNORA71B | 0 | 0 | 0 | 0 | 1 | 0 |
| SNORA71C | 0 | 0 | 0 | 0 | 1 | 0 |
| SNORA71D | 0 | 0 | 0 | 0 | 1 | 0 |
| SNORA73B | 0 | 0 | 1 | 0 | 0 | 0 |
| SNORA76 | 0 | 0 | 0 | 0 | 1 | 0 |
| SNORA79 | 0 | 0 | 0 | 0 | 1 | 0 |
| SNORA81 | 0 | 0 | 1 | 0 | 0 | 0 |
| SNORD112 | 0 | 0 | 0 | 0 | 1 | 0 |
| SNORD118 | 0 | 0 | 1 | 0 | 1 | 0 |
| SNORD12 | 0 | 0 | 1 | 0 | 0 | 0 |
| SNORD12C | 0 | 0 | 0 | 0 | 1 | 0 |
| SNORD25 | 0 | 0 | 0 | 0 | 1 | 0 |
| SNORD34 | 0 | 0 | 1 | 0 | 0 | 0 |
| SNORD3A | 0 | 0 | 1 | 0 | 0 | 0 |
| SNORD45A | 0 | 0 | 1 | 0 | 0 | 0 |
| SNORD45B | 0 | 0 | 0 | 0 | 1 | 0 |
| SNORD45C | 0 | 0 | 0 | 0 | 1 | 0 |
| SNORD46 | 0 | 0 | 0 | 0 | 1 | 1 |
| SNORD50B | 0 | 0 | 0 | 0 | 1 | 0 |
| SNORD64 | 0 | 0 | 0 | 0 | 0 | 1 |
| SNORD68 | 0 | 0 | 0 | 0 | 1 | 0 |
| SNORD69 | 0 | 0 | 1 | 0 | 0 | 0 |
| SNORD80 | 0 | 0 | 0 | 0 | 1 | 0 |
| SNORD96B | 0 | 0 | 0 | 0 | 1 | 0 |
| snoU13 | 0 | 0 | 0 | 0 | 1 | 1 |
| snoU2_19 | 0 | 0 | 0 | 0 | 1 | 0 |
| snoU2-30 | 0 | 0 | 0 | 0 | 1 | 1 |
| SNRK | 1 | 0 | 1 | 0 | 0 | 0 |
| SNRNP27 | 0 | 0 | 0 | 1 | 0 | 0 |
| SNRPB | 1 | 0 | 0 | 0 | 0 | 0 |
| SNRPB2 | 1 | 0 | 0 | 0 | 0 | 0 |
| SNRPD1 | 1 | 0 | 1 | 1 | 1 | 0 |
| SNRPD2 | 1 | 0 | 0 | 1 | 0 | 0 |
| SNRPD3 | 1 | 0 | 1 | 0 | 0 | 0 |
| SNRPE | 1 | 0 | 0 | 0 | 1 | 0 |
| SNRPG | 1 | 0 | 0 | 0 | 0 | 0 |
| SNTB2 | 1 | 0 | 0 | 0 | 0 | 0 |
| SNURF | 0 | 0 | 0 | 0 | 0 | 1 |
| SNX1 | 0 | 0 | 1 | 0 | 0 | 0 |
| SNX12 | 1 | 0 | 0 | 0 | 0 | 0 |
| SNX22 | 1 | 0 | 0 | 0 | 0 | 0 |
| SNX24 | 0 | 0 | 0 | 0 | 1 | 0 |
| SNX27 | 1 | 0 | 0 | 1 | 0 | 0 |
| SNX3 | 1 | 0 | 0 | 1 | 0 | 0 |
| SNX30 | 1 | 0 | 0 | 0 | 1 | 0 |
| SNX4 | 1 | 0 | 0 | 0 | 1 | 0 |
| SNX5 | 1 | 0 | 0 | 1 | 0 | 0 |
| SNX6 | 0 | 0 | 0 | 1 | 0 | 0 |
| SOAT1 | 0 | 0 | 0 | 0 | 1 | 0 |
| SOCS2 | 0 | 0 | 0 | 0 | 1 | 0 |
| SOCS3 | 0 | 0 | 0 | 1 | 0 | 0 |
| SOCS4 | 0 | 0 | 1 | 1 | 0 | 0 |
| SOCS5 | 1 | 0 | 0 | 0 | 0 | 0 |
| SOCS6 | 0 | 0 | 0 | 0 | 1 | 0 |
| SOCS7 | 0 | 0 | 0 | 0 | 1 | 0 |
| SOD1 | 1 | 0 | 0 | 0 | 0 | 0 |
| SOD2 | 1 | 0 | 0 | 0 | 0 | 0 |
| SOGA1 | 0 | 0 | 1 | 0 | 0 | 0 |
| SOHLH2 | 0 | 0 | 1 | 0 | 1 | 0 |
| SON | 0 | 0 | 0 | 1 | 0 | 0 |
| SOS2 | 0 | 0 | 0 | 1 | 0 | 0 |
| SOX10 | 1 | 0 | 0 | 0 | 0 | 0 |
| SOX13 | 1 | 1 | 1 | 0 | 0 | 0 |
| SOX4 | 1 | 0 | 0 | 0 | 0 | 0 |
| SP1 | 1 | 0 | 0 | 0 | 0 | 0 |
| SP100 | 0 | 0 | 0 | 1 | 0 | 0 |
| SP140L | 0 | 0 | 0 | 1 | 0 | 0 |
| SP3 | 1 | 0 | 0 | 1 | 0 | 0 |
| SPAG17 | 1 | 0 | 0 | 0 | 0 | 0 |
| SPAG7 | 0 | 0 | 0 | 1 | 0 | 0 |
| SPARC | 1 | 0 | 0 | 0 | 0 | 0 |
| SPATA2 | 0 | 0 | 0 | 0 | 1 | 0 |
| SPATA5 | 0 | 0 | 1 | 0 | 0 | 0 |
| SPATA5L1 | 0 | 0 | 1 | 0 | 0 | 0 |
| SPATS2L | 1 | 0 | 0 | 0 | 0 | 0 |
| SPCS1 | 1 | 0 | 0 | 0 | 0 | 0 |
| SPCS3 | 1 | 0 | 0 | 1 | 0 | 0 |
| SPDYA | 0 | 0 | 0 | 0 | 1 | 0 |
| SPECC1 | 1 | 0 | 1 | 0 | 0 | 0 |
| SPECC1L | 1 | 0 | 1 | 0 | 0 | 0 |
| SPECC1L-ADORA2A | 0 | 0 | 1 | 0 | 0 | 0 |
| SPEG | 0 | 0 | 0 | 0 | 1 | 0 |
| SPEN | 0 | 0 | 0 | 0 | 0 | 1 |
| SPG11 | 0 | 0 | 1 | 0 | 0 | 0 |
| SPG20 | 0 | 0 | 0 | 1 | 0 | 0 |
| SPG21 | 1 | 0 | 0 | 1 | 1 | 0 |
| SPHAR | 0 | 0 | 0 | 0 | 1 | 0 |
| SPIN1 | 1 | 0 | 1 | 1 | 1 | 0 |
| SPIN4 | 1 | 0 | 0 | 0 | 1 | 0 |
| SPIRE1 | 0 | 0 | 0 | 0 | 1 | 0 |
| SPNS1 | 0 | 0 | 0 | 0 | 1 | 0 |
| SPOCK1 | 0 | 0 | 1 | 0 | 0 | 0 |
| SPOP | 0 | 0 | 0 | 1 | 0 | 0 |
| SPOPL | 1 | 0 | 0 | 1 | 1 | 0 |
| SPPL2A | 0 | 0 | 0 | 0 | 1 | 0 |
| SPPL3 | 0 | 0 | 0 | 0 | 1 | 0 |
| SPR | 0 | 0 | 0 | 0 | 1 | 0 |
| SPRED1 | 1 | 0 | 0 | 0 | 0 | 0 |
| SPRED2 | 0 | 0 | 0 | 0 | 1 | 0 |
| SPRR1A | 0 | 1 | 0 | 0 | 0 | 0 |
| SPRR2A | 0 | 0 | 0 | 0 | 1 | 0 |
| SPRR2B | 0 | 0 | 0 | 0 | 1 | 0 |
| SPRR2D | 0 | 0 | 0 | 0 | 1 | 0 |
| SPRR2E | 0 | 0 | 0 | 0 | 1 | 0 |
| SPRR2F | 0 | 0 | 0 | 0 | 1 | 0 |
| SPRY1 | 1 | 0 | 0 | 0 | 0 | 0 |
| SPRY4 | 0 | 0 | 0 | 0 | 1 | 0 |
| SPRYD7 | 1 | 0 | 0 | 0 | 1 | 0 |
| SPTAN1 | 0 | 0 | 1 | 0 | 0 | 0 |
| SPTLC1 | 0 | 0 | 0 | 1 | 0 | 0 |
| SPTSSA | 1 | 0 | 1 | 0 | 1 | 0 |
| SQRDL | 1 | 0 | 0 | 0 | 0 | 0 |
| SQSTM1 | 1 | 0 | 1 | 0 | 0 | 0 |
| SR140 | 0 | 0 | 0 | 1 | 0 | 0 |
| SRD5A1 | 1 | 0 | 1 | 0 | 1 | 0 |
| SREBF2 | 0 | 0 | 1 | 0 | 0 | 0 |
| SREK1 | 1 | 0 | 0 | 0 | 0 | 0 |
| SRGAP1 | 1 | 0 | 0 | 0 | 0 | 0 |
| SRGAP3 | 0 | 1 | 0 | 0 | 0 | 0 |
| SRGN | 0 | 0 | 0 | 1 | 0 | 0 |
| SRI | 1 | 0 | 0 | 0 | 0 | 0 |
| SRM | 0 | 0 | 1 | 0 | 0 | 0 |
| SRP14 | 1 | 0 | 0 | 0 | 0 | 0 |
| SRP19 | 0 | 0 | 0 | 1 | 0 | 0 |
| SRP68 | 0 | 0 | 1 | 0 | 0 | 0 |
| SRP72 | 1 | 0 | 0 | 1 | 0 | 0 |
| SRP9 | 1 | 0 | 1 | 1 | 0 | 0 |
| SRPK1 | 1 | 0 | 1 | 0 | 0 | 0 |
| SRPK2 | 0 | 0 | 1 | 1 | 0 | 0 |
| SRPRB | 0 | 0 | 0 | 0 | 1 | 0 |
| SRR | 1 | 0 | 0 | 0 | 0 | 0 |
| SRRD | 0 | 0 | 0 | 0 | 1 | 0 |
| SRRM1 | 0 | 0 | 0 | 1 | 0 | 0 |
| SRRM2 | 1 | 0 | 1 | 0 | 0 | 0 |
| SRRT | 0 | 0 | 1 | 0 | 0 | 0 |
| SRSF1 | 1 | 0 | 0 | 0 | 0 | 0 |
| SRSF11 | 0 | 0 | 1 | 0 | 0 | 0 |
| SRSF2 | 1 | 0 | 0 | 0 | 0 | 0 |
| SRSF3 | 1 | 0 | 0 | 0 | 0 | 0 |
| SRSF6 | 1 | 0 | 0 | 0 | 0 | 0 |
| SRSF7 | 1 | 0 | 0 | 0 | 0 | 0 |
| SRSF8 | 0 | 0 | 0 | 0 | 1 | 0 |
| SRSF9 | 1 | 0 | 0 | 0 | 0 | 0 |
| SS18 | 1 | 0 | 0 | 1 | 0 | 0 |
| SS18L1 | 0 | 0 | 0 | 1 | 1 | 0 |
| SSB | 1 | 0 | 0 | 1 | 0 | 0 |
| SSBP1 | 0 | 0 | 1 | 0 | 0 | 0 |
| SSFA2 | 1 | 0 | 1 | 0 | 0 | 0 |
| SSH2 | 1 | 0 | 0 | 0 | 0 | 0 |
| SSH3 | 1 | 0 | 0 | 0 | 0 | 0 |
| SSR1 | 1 | 0 | 1 | 1 | 0 | 0 |
| SSR2 | 1 | 0 | 0 | 0 | 0 | 0 |
| SSR3 | 1 | 0 | 1 | 0 | 0 | 0 |
| SSRP1 | 0 | 0 | 0 | 0 | 0 | 1 |
| SSTR2 | 0 | 0 | 0 | 0 | 0 | 1 |
| SSX2IP | 0 | 0 | 1 | 0 | 0 | 0 |
| SSX5 | 0 | 0 | 0 | 0 | 1 | 0 |
| SSX9 | 0 | 0 | 0 | 0 | 1 | 0 |
| ST13 | 1 | 0 | 1 | 1 | 0 | 0 |
| ST20-MTHFS | 0 | 0 | 0 | 0 | 1 | 0 |
| ST3GAL5 | 0 | 0 | 0 | 0 | 1 | 0 |
| ST7 | 0 | 0 | 1 | 0 | 0 | 0 |
| ST7-OT4 | 0 | 0 | 1 | 0 | 0 | 0 |
| ST8SIA4 | 0 | 0 | 0 | 0 | 1 | 0 |
| STAG1 | 0 | 0 | 1 | 0 | 0 | 0 |
| STAG2 | 1 | 0 | 0 | 1 | 0 | 0 |
| STAP1 | 0 | 0 | 0 | 1 | 0 | 0 |
| STARD3NL | 0 | 0 | 0 | 1 | 1 | 0 |
| STARD4 | 1 | 0 | 0 | 0 | 0 | 0 |
| STARD7 | 1 | 0 | 0 | 0 | 0 | 0 |
| STAT1 | 0 | 0 | 0 | 1 | 0 | 0 |
| STAT2 | 0 | 0 | 0 | 1 | 0 | 0 |
| STAT3 | 1 | 0 | 0 | 0 | 0 | 0 |
| STAT5B | 1 | 0 | 0 | 0 | 0 | 0 |
| STAU1 | 1 | 0 | 0 | 1 | 0 | 0 |
| STC2 | 0 | 0 | 0 | 0 | 1 | 0 |
| STEAP4 | 0 | 0 | 1 | 0 | 0 | 0 |
| STIM1 | 0 | 1 | 0 | 0 | 1 | 0 |
| STK17A | 1 | 0 | 0 | 0 | 0 | 0 |
| STK19 | 0 | 0 | 0 | 0 | 1 | 0 |
| STK19P | 0 | 0 | 0 | 0 | 1 | 0 |
| STK36 | 0 | 0 | 0 | 0 | 1 | 0 |
| STK38 | 0 | 0 | 0 | 1 | 0 | 0 |
| STK4 | 1 | 0 | 0 | 1 | 0 | 0 |
| STMN1 | 1 | 0 | 1 | 0 | 0 | 0 |
| STOML1 | 0 | 1 | 0 | 0 | 0 | 0 |
| STOML2 | 0 | 0 | 0 | 1 | 0 | 0 |
| STON1 | 0 | 1 | 0 | 0 | 1 | 0 |
| STON1-GTF2A1L | 0 | 0 | 0 | 0 | 1 | 0 |
| STON2 | 0 | 1 | 0 | 0 | 0 | 0 |
| STOX2 | 0 | 0 | 1 | 0 | 0 | 0 |
| STRADA | 0 | 0 | 1 | 0 | 0 | 0 |
| STRAP | 1 | 0 | 0 | 0 | 0 | 0 |
| STRBP | 0 | 0 | 0 | 1 | 0 | 0 |
| STRN | 0 | 1 | 0 | 0 | 0 | 0 |
| STRN4 | 0 | 0 | 1 | 0 | 0 | 0 |
| STT3B | 0 | 0 | 1 | 0 | 0 | 0 |
| STX12 | 0 | 0 | 0 | 1 | 1 | 0 |
| STX16 | 1 | 0 | 1 | 0 | 0 | 0 |
| STX16-NPEPL1 | 0 | 0 | 1 | 0 | 0 | 0 |
| STX18-AS1 | 0 | 0 | 1 | 0 | 0 | 0 |
| STX1B | 0 | 0 | 0 | 0 | 1 | 0 |
| STX3 | 1 | 0 | 0 | 0 | 0 | 0 |
| STX6 | 1 | 0 | 0 | 0 | 1 | 0 |
| STXBP1 | 0 | 0 | 1 | 0 | 0 | 0 |
| STXBP5 | 0 | 0 | 1 | 0 | 0 | 0 |
| STYX | 1 | 0 | 0 | 0 | 1 | 0 |
| STYXL1 | 0 | 0 | 0 | 0 | 1 | 0 |
| SUB1 | 1 | 0 | 0 | 1 | 0 | 0 |
| SUB1P3 | 0 | 0 | 0 | 0 | 1 | 0 |
| SUCLA2 | 0 | 0 | 0 | 0 | 1 | 0 |
| SUCO | 1 | 0 | 0 | 0 | 0 | 0 |
| SUDS3 | 1 | 0 | 0 | 0 | 1 | 0 |
| SUGT1 | 0 | 0 | 1 | 0 | 0 | 0 |
| SUMF1 | 1 | 0 | 1 | 0 | 0 | 0 |
| SUMO1 | 1 | 0 | 0 | 1 | 0 | 0 |
| SUMO2 | 1 | 0 | 0 | 1 | 0 | 0 |
| SUMO3 | 1 | 0 | 0 | 1 | 0 | 0 |
| SUMO4 | 0 | 0 | 0 | 0 | 1 | 0 |
| SUN1 | 0 | 0 | 1 | 0 | 0 | 0 |
| SUN2 | 0 | 0 | 0 | 0 | 1 | 0 |
| SUPT16H | 1 | 0 | 1 | 0 | 0 | 0 |
| SUPT20H | 1 | 0 | 1 | 0 | 0 | 0 |
| SUPT6H | 0 | 0 | 1 | 0 | 0 | 0 |
| SUSD5 | 1 | 0 | 0 | 0 | 0 | 0 |
| SVIP | 0 | 0 | 0 | 1 | 0 | 0 |
| SYAP1 | 1 | 0 | 0 | 0 | 0 | 0 |
| SYBU | 0 | 0 | 0 | 0 | 1 | 0 |
| SYCE1L | 0 | 0 | 0 | 0 | 1 | 0 |
| SYF2 | 0 | 0 | 0 | 1 | 0 | 0 |
| SYK | 0 | 0 | 0 | 0 | 1 | 0 |
| SYMPK | 0 | 0 | 1 | 0 | 0 | 0 |
| SYNC | 1 | 0 | 0 | 0 | 0 | 0 |
| SYNCRIP | 1 | 0 | 0 | 0 | 0 | 0 |
| SYNE2 | 0 | 0 | 1 | 1 | 0 | 0 |
| SYNGR3 | 0 | 0 | 0 | 0 | 1 | 0 |
| SYPL1 | 1 | 0 | 1 | 1 | 0 | 0 |
| SYS1 | 0 | 0 | 0 | 0 | 1 | 0 |
| SYS1-DBNDD2 | 0 | 0 | 0 | 0 | 1 | 0 |
| SYT17 | 0 | 1 | 0 | 0 | 0 | 0 |
| TAB1 | 0 | 0 | 0 | 0 | 1 | 0 |
| TAB2 | 0 | 0 | 0 | 0 | 1 | 0 |
| TACC1 | 1 | 0 | 0 | 1 | 0 | 0 |
| TACC3 | 0 | 0 | 1 | 0 | 0 | 0 |
| TADA1L | 0 | 0 | 0 | 1 | 0 | 0 |
| TAF1 | 1 | 0 | 0 | 1 | 0 | 0 |
| TAF11 | 0 | 0 | 0 | 0 | 1 | 0 |
| TAF13 | 1 | 0 | 0 | 0 | 1 | 0 |
| TAF1L | 1 | 0 | 0 | 0 | 0 | 0 |
| TAF4B | 0 | 0 | 0 | 1 | 0 | 0 |
| TAF5 | 1 | 0 | 0 | 0 | 0 | 0 |
| TAF7 | 1 | 0 | 0 | 1 | 1 | 0 |
| TAF9B | 0 | 0 | 0 | 1 | 0 | 0 |
| TAGAP | 0 | 0 | 0 | 1 | 0 | 0 |
| TAGLN2 | 1 | 0 | 0 | 1 | 0 | 0 |
| TAMM41 | 0 | 0 | 1 | 0 | 0 | 0 |
| TANC2 | 1 | 0 | 1 | 0 | 0 | 0 |
| TANK | 0 | 0 | 0 | 1 | 0 | 0 |
| TAOK1 | 1 | 0 | 0 | 0 | 0 | 0 |
| TAOK2 | 0 | 0 | 1 | 0 | 0 | 0 |
| TAP1 | 0 | 0 | 0 | 1 | 0 | 0 |
| TAP2 | 0 | 0 | 1 | 0 | 1 | 0 |
| TAPT1 | 0 | 0 | 0 | 0 | 1 | 0 |
| TAPT1-AS1 | 0 | 0 | 1 | 0 | 0 | 0 |
| TARBP1 | 0 | 0 | 1 | 0 | 0 | 0 |
| TARDBP | 1 | 0 | 1 | 0 | 0 | 0 |
| TARP | 0 | 0 | 0 | 1 | 0 | 0 |
| TARS | 0 | 0 | 0 | 1 | 0 | 0 |
| TASP1 | 0 | 0 | 0 | 0 | 1 | 0 |
| TAT | 0 | 1 | 0 | 0 | 0 | 0 |
| TATDN1 | 0 | 0 | 0 | 1 | 0 | 0 |
| TATDN2 | 0 | 0 | 1 | 0 | 0 | 0 |
| TAX1BP1 | 1 | 0 | 1 | 0 | 0 | 0 |
| TBC1D10B | 0 | 0 | 1 | 0 | 1 | 0 |
| TBC1D13 | 1 | 0 | 0 | 0 | 1 | 0 |
| TBC1D14 | 0 | 0 | 1 | 0 | 0 | 0 |
| TBC1D16 | 0 | 1 | 0 | 0 | 0 | 0 |
| TBC1D24 | 0 | 1 | 0 | 0 | 0 | 0 |
| TBC1D28 | 0 | 0 | 0 | 0 | 1 | 0 |
| TBC1D3P1-DHX40P1 | 0 | 0 | 0 | 0 | 1 | 0 |
| TBC1D4 | 0 | 0 | 1 | 1 | 0 | 0 |
| TBC1D5 | 0 | 0 | 1 | 0 | 0 | 0 |
| TBC1D7 | 0 | 0 | 0 | 0 | 1 | 0 |
| TBCA | 1 | 0 | 0 | 0 | 0 | 0 |
| TBCB | 0 | 0 | 0 | 1 | 0 | 0 |
| TBCC | 0 | 0 | 0 | 0 | 1 | 0 |
| TBCD | 0 | 0 | 1 | 0 | 0 | 0 |
| TBL1X | 0 | 1 | 1 | 0 | 0 | 0 |
| TBL1XR1 | 1 | 0 | 1 | 0 | 0 | 0 |
| TBPL1 | 1 | 0 | 0 | 0 | 0 | 0 |
| TBX21 | 0 | 1 | 0 | 0 | 0 | 0 |
| tcag7.903 | 0 | 0 | 0 | 1 | 0 | 0 |
| TCEA1 | 1 | 0 | 0 | 1 | 0 | 0 |
| TCEAL7 | 0 | 0 | 0 | 0 | 1 | 0 |
| TCEAL8 | 1 | 0 | 0 | 0 | 0 | 0 |
| TCEANC | 0 | 0 | 0 | 0 | 1 | 0 |
| TCEB1 | 1 | 0 | 0 | 1 | 0 | 0 |
| TCERG1 | 0 | 0 | 1 | 1 | 0 | 0 |
| TCF20 | 0 | 0 | 1 | 0 | 1 | 0 |
| TCF7L2 | 1 | 0 | 1 | 0 | 1 | 0 |
| TCFL5 | 0 | 0 | 1 | 0 | 0 | 0 |
| TCP1 | 1 | 0 | 0 | 1 | 0 | 0 |
| TCTN2 | 0 | 0 | 0 | 0 | 1 | 0 |
| TDG | 0 | 0 | 0 | 1 | 0 | 0 |
| TDRD3 | 0 | 0 | 1 | 1 | 0 | 0 |
| TEAD1 | 1 | 0 | 1 | 0 | 0 | 0 |
| TENM3 | 0 | 0 | 1 | 0 | 0 | 0 |
| TERF2 | 0 | 0 | 1 | 0 | 0 | 0 |
| TERF2IP | 1 | 0 | 0 | 0 | 1 | 0 |
| TET2 | 0 | 1 | 0 | 0 | 0 | 0 |
| TET3 | 1 | 0 | 0 | 1 | 0 | 0 |
| TEX14 | 0 | 0 | 0 | 0 | 1 | 1 |
| TEX2 | 0 | 0 | 1 | 0 | 0 | 0 |
| TEX261 | 0 | 0 | 0 | 0 | 1 | 0 |
| TEX30 | 0 | 0 | 0 | 0 | 1 | 0 |
| TFAM | 0 | 0 | 0 | 1 | 0 | 0 |
| TFAP2A | 0 | 0 | 1 | 0 | 0 | 0 |
| TFCP2 | 1 | 0 | 0 | 0 | 0 | 0 |
| TFDP1 | 1 | 0 | 1 | 0 | 0 | 0 |
| TFDP2 | 0 | 0 | 1 | 0 | 0 | 0 |
| TFG | 0 | 0 | 1 | 0 | 0 | 0 |
| TFRC | 1 | 0 | 1 | 1 | 0 | 0 |
| TGDS | 0 | 0 | 1 | 0 | 1 | 0 |
| TGFBR1 | 1 | 0 | 1 | 0 | 1 | 0 |
| TGFBR2 | 1 | 0 | 0 | 1 | 0 | 0 |
| TGFBR3 | 0 | 0 | 1 | 1 | 0 | 0 |
| TGIF2LY | 0 | 1 | 0 | 0 | 0 | 0 |
| TGM4 | 0 | 1 | 0 | 0 | 0 | 0 |
| TGOLN2 | 1 | 0 | 1 | 0 | 0 | 0 |
| THBD | 0 | 0 | 0 | 0 | 1 | 0 |
| THBS1 | 1 | 0 | 0 | 0 | 0 | 0 |
| THNSL1 | 0 | 0 | 0 | 0 | 1 | 0 |
| THOC3 | 1 | 0 | 0 | 0 | 0 | 0 |
| THRAP3 | 1 | 0 | 0 | 0 | 0 | 0 |
| THUMPD1 | 1 | 0 | 0 | 1 | 1 | 0 |
| TIAF1 | 0 | 0 | 1 | 0 | 0 | 0 |
| TIAL1 | 0 | 0 | 1 | 0 | 0 | 0 |
| TICAM2 | 1 | 0 | 0 | 0 | 0 | 0 |
| TICRR | 0 | 0 | 1 | 0 | 0 | 0 |
| TIFA | 0 | 0 | 0 | 1 | 1 | 0 |
| TIGD1 | 0 | 0 | 0 | 0 | 1 | 0 |
| TIGIT | 0 | 0 | 0 | 1 | 0 | 0 |
| TIMM10 | 0 | 0 | 0 | 0 | 1 | 0 |
| TIMM10B | 0 | 0 | 1 | 0 | 0 | 0 |
| TIMM17A | 1 | 0 | 0 | 0 | 0 | 0 |
| TIMM17B | 0 | 1 | 0 | 0 | 0 | 0 |
| TIMM22 | 0 | 0 | 0 | 0 | 1 | 0 |
| TIMM8A | 0 | 0 | 0 | 0 | 1 | 0 |
| TIMMDC1 | 0 | 0 | 1 | 0 | 0 | 0 |
| TIMP2 | 1 | 0 | 1 | 0 | 0 | 0 |
| TIPRL | 0 | 0 | 0 | 1 | 0 | 0 |
| TLE1 | 0 | 1 | 0 | 0 | 0 | 0 |
| TLE4 | 0 | 0 | 0 | 0 | 1 | 0 |
| TM2D1 | 0 | 0 | 0 | 0 | 1 | 0 |
| TM2D2 | 0 | 0 | 0 | 0 | 1 | 0 |
| TM2D3 | 0 | 0 | 0 | 1 | 0 | 0 |
| TM4SF1 | 1 | 0 | 1 | 0 | 0 | 0 |
| TM4SF2 | 0 | 0 | 0 | 0 | 1 | 0 |
| TM4SF4 | 0 | 1 | 0 | 0 | 0 | 0 |
| TM6SF1 | 0 | 0 | 0 | 0 | 1 | 0 |
| TM9SF1 | 0 | 0 | 0 | 0 | 1 | 0 |
| TM9SF2 | 0 | 0 | 0 | 1 | 1 | 0 |
| TM9SF3 | 1 | 0 | 1 | 1 | 0 | 0 |
| TM9SF4 | 0 | 0 | 1 | 0 | 0 | 0 |
| TMA7 | 1 | 0 | 0 | 0 | 0 | 0 |
| TMBIM4 | 0 | 0 | 0 | 0 | 1 | 0 |
| TMBIM6 | 1 | 0 | 1 | 0 | 0 | 0 |
| TMC5 | 0 | 1 | 0 | 0 | 0 | 0 |
| TMC6 | 0 | 0 | 0 | 1 | 0 | 0 |
| TMCO1 | 1 | 0 | 1 | 1 | 1 | 0 |
| TMCO2 | 0 | 0 | 0 | 0 | 0 | 1 |
| TMCO3 | 0 | 0 | 0 | 0 | 1 | 0 |
| TMCO6 | 0 | 0 | 0 | 1 | 0 | 0 |
| TMED10 | 1 | 0 | 1 | 0 | 1 | 0 |
| TMED10P1 | 0 | 0 | 0 | 0 | 1 | 0 |
| TMED2 | 1 | 0 | 0 | 0 | 0 | 0 |
| TMED4 | 0 | 0 | 1 | 0 | 0 | 0 |
| TMED7 | 1 | 0 | 0 | 0 | 0 | 0 |
| TMED7-TICAM2 | 1 | 0 | 0 | 0 | 0 | 0 |
| TMED8 | 1 | 0 | 0 | 0 | 1 | 0 |
| TMEFF1 | 0 | 0 | 0 | 0 | 1 | 0 |
| TMEM101 | 0 | 0 | 0 | 0 | 1 | 0 |
| TMEM106B | 0 | 0 | 1 | 0 | 1 | 0 |
| TMEM107 | 1 | 0 | 1 | 0 | 1 | 0 |
| TMEM109 | 1 | 0 | 0 | 0 | 1 | 0 |
| TMEM110 | 0 | 0 | 0 | 0 | 1 | 0 |
| TMEM110-MUSTN1 | 0 | 0 | 0 | 0 | 1 | 0 |
| TMEM116 | 0 | 0 | 0 | 0 | 1 | 0 |
| TMEM120A | 0 | 0 | 0 | 0 | 1 | 0 |
| TMEM123 | 1 | 0 | 0 | 0 | 0 | 0 |
| TMEM126B | 0 | 0 | 0 | 1 | 0 | 0 |
| TMEM128 | 0 | 0 | 0 | 0 | 1 | 0 |
| TMEM136 | 0 | 0 | 0 | 0 | 1 | 0 |
| TMEM144 | 0 | 0 | 0 | 0 | 1 | 0 |
| TMEM14A | 0 | 0 | 0 | 0 | 1 | 0 |
| TMEM14B | 0 | 0 | 0 | 1 | 0 | 0 |
| TMEM150A | 0 | 0 | 0 | 0 | 1 | 0 |
| TMEM161B | 0 | 0 | 0 | 0 | 1 | 0 |
| TMEM164 | 1 | 0 | 0 | 0 | 1 | 0 |
| TMEM165 | 0 | 0 | 1 | 0 | 0 | 0 |
| TMEM167A | 1 | 0 | 0 | 1 | 0 | 0 |
| TMEM168 | 0 | 0 | 0 | 0 | 1 | 0 |
| TMEM175 | 0 | 0 | 0 | 0 | 1 | 0 |
| TMEM179B | 0 | 0 | 0 | 0 | 1 | 0 |
| TMEM18 | 0 | 0 | 0 | 0 | 1 | 0 |
| TMEM184B | 0 | 0 | 1 | 0 | 0 | 0 |
| TMEM184C | 0 | 0 | 0 | 0 | 1 | 0 |
| TMEM185A | 0 | 0 | 0 | 0 | 1 | 0 |
| TMEM185B | 0 | 0 | 0 | 0 | 1 | 0 |
| TMEM186 | 0 | 0 | 0 | 0 | 1 | 0 |
| TMEM188 | 0 | 0 | 0 | 1 | 0 | 0 |
| TMEM189 | 0 | 0 | 1 | 1 | 0 | 0 |
| TMEM189-UBE2V1 | 0 | 0 | 1 | 0 | 0 | 0 |
| TMEM19 | 1 | 0 | 0 | 1 | 1 | 0 |
| TMEM194B | 0 | 0 | 0 | 0 | 1 | 1 |
| TMEM199 | 0 | 0 | 0 | 0 | 1 | 0 |
| TMEM2 | 1 | 0 | 0 | 0 | 1 | 0 |
| TMEM203 | 0 | 0 | 0 | 0 | 1 | 0 |
| TMEM209 | 0 | 0 | 1 | 0 | 0 | 0 |
| TMEM212 | 1 | 0 | 0 | 0 | 0 | 0 |
| TMEM217 | 0 | 0 | 0 | 0 | 0 | 1 |
| TMEM218 | 0 | 0 | 0 | 0 | 1 | 0 |
| TMEM222 | 1 | 0 | 0 | 0 | 0 | 0 |
| TMEM237 | 1 | 0 | 0 | 0 | 1 | 0 |
| TMEM238 | 0 | 0 | 1 | 0 | 0 | 0 |
| TMEM241 | 0 | 0 | 0 | 0 | 1 | 0 |
| TMEM245 | 0 | 0 | 1 | 0 | 0 | 0 |
| TMEM248 | 1 | 0 | 1 | 0 | 0 | 0 |
| TMEM30A | 1 | 0 | 0 | 1 | 1 | 0 |
| TMEM30B | 0 | 0 | 0 | 0 | 1 | 0 |
| TMEM33 | 1 | 0 | 1 | 1 | 1 | 0 |
| TMEM38B | 0 | 0 | 0 | 1 | 0 | 0 |
| TMEM39B | 0 | 0 | 0 | 0 | 1 | 0 |
| TMEM41A | 0 | 0 | 0 | 0 | 1 | 0 |
| TMEM43 | 0 | 0 | 0 | 0 | 1 | 0 |
| TMEM45A | 0 | 0 | 0 | 0 | 1 | 0 |
| TMEM47 | 1 | 0 | 0 | 0 | 0 | 0 |
| TMEM48 | 1 | 0 | 0 | 0 | 1 | 0 |
| TMEM49 | 0 | 1 | 0 | 0 | 0 | 0 |
| TMEM5 | 0 | 0 | 0 | 0 | 1 | 0 |
| TMEM50B | 0 | 0 | 0 | 1 | 0 | 0 |
| TMEM55B | 0 | 0 | 0 | 0 | 1 | 0 |
| TMEM57 | 0 | 0 | 0 | 0 | 1 | 0 |
| TMEM59 | 1 | 0 | 0 | 0 | 0 | 0 |
| TMEM60 | 0 | 0 | 0 | 0 | 1 | 0 |
| TMEM62 | 0 | 0 | 0 | 0 | 1 | 0 |
| TMEM63A | 0 | 0 | 1 | 0 | 0 | 0 |
| TMEM64 | 1 | 0 | 0 | 1 | 1 | 0 |
| TMEM65 | 0 | 0 | 0 | 0 | 1 | 0 |
| TMEM66 | 1 | 0 | 0 | 0 | 0 | 0 |
| TMEM68 | 0 | 0 | 0 | 0 | 1 | 0 |
| TMEM69 | 0 | 0 | 0 | 1 | 0 | 0 |
| TMEM70 | 0 | 0 | 0 | 1 | 1 | 0 |
| TMEM79 | 0 | 0 | 0 | 0 | 1 | 0 |
| TMEM85 | 0 | 0 | 0 | 1 | 0 | 0 |
| TMEM86A | 0 | 0 | 0 | 0 | 1 | 0 |
| TMEM8B | 0 | 0 | 0 | 0 | 1 | 0 |
| TMEM9 | 0 | 0 | 0 | 0 | 1 | 0 |
| TMEM93 | 0 | 0 | 0 | 1 | 0 | 0 |
| TMEM97 | 0 | 0 | 1 | 0 | 0 | 0 |
| TMEM98 | 0 | 0 | 0 | 0 | 1 | 0 |
| TMEM99 | 0 | 0 | 0 | 0 | 1 | 0 |
| TMEM9B | 1 | 0 | 0 | 0 | 1 | 0 |
| TMOD3 | 0 | 0 | 0 | 1 | 0 | 0 |
| TMPO | 1 | 0 | 0 | 1 | 0 | 0 |
| TMPPE | 0 | 0 | 1 | 0 | 1 | 0 |
| TMSB10 | 1 | 0 | 0 | 0 | 0 | 0 |
| TMTC3 | 0 | 0 | 0 | 0 | 1 | 0 |
| TMTC4 | 1 | 0 | 0 | 1 | 1 | 0 |
| TMX1 | 1 | 0 | 0 | 1 | 0 | 0 |
| TMX2 | 1 | 0 | 0 | 0 | 0 | 0 |
| TMX3 | 1 | 0 | 1 | 1 | 1 | 0 |
| TMX4 | 0 | 0 | 0 | 0 | 1 | 0 |
| TNC | 1 | 0 | 0 | 0 | 0 | 0 |
| TNFAIP1 | 0 | 0 | 1 | 0 | 0 | 0 |
| TNFAIP8 | 0 | 0 | 0 | 1 | 0 | 0 |
| TNFRSF10A | 0 | 0 | 0 | 0 | 1 | 0 |
| TNFRSF10B | 0 | 0 | 0 | 0 | 1 | 0 |
| TNFRSF1A | 0 | 0 | 0 | 0 | 1 | 0 |
| TNFRSF1B | 0 | 0 | 0 | 1 | 1 | 0 |
| TNFRSF21 | 1 | 0 | 0 | 0 | 1 | 0 |
| TNFSF10 | 0 | 0 | 0 | 1 | 0 | 0 |
| TNFSF13B | 0 | 0 | 0 | 1 | 0 | 0 |
| TNFSF4 | 0 | 0 | 0 | 0 | 0 | 1 |
| TNFSF8 | 0 | 0 | 0 | 1 | 0 | 0 |
| TNFSF9 | 0 | 0 | 1 | 0 | 0 | 0 |
| TNIP1 | 0 | 0 | 1 | 1 | 0 | 0 |
| TNK1 | 0 | 1 | 0 | 0 | 0 | 0 |
| TNK2 | 0 | 0 | 1 | 0 | 0 | 0 |
| TNKS | 1 | 0 | 0 | 0 | 0 | 0 |
| TNKS2 | 1 | 0 | 0 | 1 | 0 | 0 |
| TNPO1 | 1 | 0 | 1 | 0 | 0 | 0 |
| TNPO2 | 1 | 0 | 0 | 0 | 0 | 0 |
| TNPO3 | 0 | 0 | 1 | 0 | 0 | 0 |
| TNRC18 | 0 | 0 | 1 | 0 | 0 | 0 |
| TNRC6A | 1 | 0 | 1 | 0 | 0 | 0 |
| TNRC6B | 1 | 0 | 1 | 0 | 0 | 0 |
| TNRC6C | 0 | 0 | 0 | 0 | 1 | 0 |
| TNS3 | 0 | 0 | 1 | 0 | 0 | 0 |
| TOM1L2 | 1 | 0 | 0 | 0 | 0 | 0 |
| TOMM20 | 1 | 0 | 1 | 1 | 0 | 0 |
| TOMM22 | 1 | 0 | 0 | 0 | 0 | 0 |
| TOMM34 | 1 | 0 | 0 | 0 | 0 | 0 |
| TOMM5 | 1 | 0 | 0 | 1 | 0 | 0 |
| TOMM6 | 1 | 0 | 0 | 0 | 0 | 0 |
| TOMM7 | 1 | 0 | 0 | 0 | 0 | 0 |
| TOMM70A | 1 | 0 | 0 | 0 | 1 | 0 |
| TOP1 | 1 | 0 | 0 | 1 | 0 | 0 |
| TOP2A | 1 | 0 | 1 | 0 | 0 | 0 |
| TOP2B | 1 | 0 | 0 | 0 | 0 | 0 |
| TOR1AIP2 | 1 | 0 | 0 | 0 | 0 | 0 |
| TP53 | 0 | 0 | 0 | 0 | 1 | 0 |
| TP53INP1 | 0 | 0 | 0 | 0 | 1 | 0 |
| TP53INP2 | 0 | 0 | 0 | 0 | 1 | 0 |
| TP53RK | 1 | 0 | 0 | 0 | 1 | 0 |
| TP53TG3 | 0 | 0 | 0 | 0 | 1 | 0 |
| TPBG | 0 | 0 | 0 | 0 | 1 | 0 |
| TPCN2 | 0 | 0 | 0 | 0 | 1 | 0 |
| TPD52 | 0 | 0 | 0 | 1 | 1 | 0 |
| TPGS2 | 1 | 0 | 1 | 0 | 0 | 0 |
| TPI1 | 1 | 0 | 0 | 0 | 0 | 0 |
| TPM1 | 1 | 0 | 0 | 0 | 0 | 0 |
| TPM2 | 0 | 0 | 1 | 0 | 0 | 0 |
| TPM3 | 1 | 0 | 0 | 0 | 0 | 0 |
| TPM4 | 1 | 0 | 0 | 0 | 0 | 0 |
| TPRN | 0 | 0 | 0 | 0 | 1 | 0 |
| TPT1 | 1 | 0 | 0 | 0 | 1 | 0 |
| TPT1-AS1 | 0 | 0 | 1 | 0 | 0 | 0 |
| TPX2 | 0 | 0 | 1 | 0 | 0 | 0 |
| TRA2A | 1 | 0 | 0 | 0 | 0 | 0 |
| TRA2B | 1 | 0 | 1 | 1 | 0 | 0 |
| TRAC | 0 | 0 | 0 | 1 | 0 | 0 |
| TRAF2 | 0 | 0 | 1 | 0 | 0 | 0 |
| TRAF3 | 0 | 0 | 1 | 0 | 0 | 0 |
| TRAF4 | 0 | 0 | 0 | 0 | 1 | 0 |
| TRAK1 | 0 | 0 | 1 | 0 | 0 | 0 |
| TRAM1 | 1 | 0 | 0 | 1 | 0 | 0 |
| TRAPPC10 | 1 | 0 | 0 | 1 | 0 | 0 |
| TRAPPC11 | 0 | 0 | 0 | 0 | 1 | 0 |
| TRAPPC2 | 0 | 0 | 0 | 0 | 1 | 0 |
| TRAPPC3 | 0 | 0 | 0 | 1 | 0 | 0 |
| TRIB1 | 1 | 0 | 0 | 0 | 1 | 0 |
| TRIB2 | 0 | 0 | 0 | 0 | 1 | 0 |
| TRIB3 | 0 | 0 | 1 | 0 | 0 | 0 |
| TRIM22 | 0 | 0 | 0 | 1 | 0 | 0 |
| TRIM25 | 0 | 0 | 1 | 0 | 0 | 0 |
| TRIM28 | 1 | 0 | 0 | 0 | 0 | 0 |
| TRIM32 | 0 | 0 | 0 | 0 | 1 | 0 |
| TRIM4 | 1 | 0 | 0 | 0 | 1 | 0 |
| TRIM5 | 0 | 0 | 0 | 0 | 1 | 0 |
| TRIM58 | 1 | 0 | 0 | 0 | 1 | 0 |
| TRIM59 | 0 | 0 | 0 | 0 | 1 | 0 |
| TRIM69 | 0 | 0 | 0 | 1 | 0 | 0 |
| TRIO | 0 | 0 | 1 | 0 | 0 | 0 |
| TRIP10 | 0 | 0 | 1 | 0 | 0 | 0 |
| TRIP12 | 1 | 0 | 0 | 0 | 0 | 0 |
| TRIP13 | 0 | 0 | 1 | 0 | 0 | 0 |
| TRIP6 | 1 | 0 | 0 | 0 | 0 | 0 |
| TRMT10C | 0 | 0 | 0 | 0 | 1 | 0 |
| TRMT112 | 1 | 0 | 0 | 0 | 0 | 0 |
| TRMT12 | 0 | 0 | 0 | 0 | 1 | 0 |
| TRMT13 | 0 | 0 | 1 | 0 | 0 | 0 |
| TRMT5 | 0 | 0 | 0 | 1 | 0 | 0 |
| TRMT6 | 0 | 0 | 0 | 1 | 0 | 0 |
| TRMU | 0 | 0 | 0 | 0 | 1 | 0 |
| TROVE2 | 1 | 0 | 0 | 0 | 0 | 0 |
| TRPC4AP | 0 | 0 | 1 | 0 | 0 | 0 |
| TRPM8 | 0 | 1 | 0 | 0 | 0 | 0 |
| TRPS1 | 1 | 0 | 0 | 0 | 0 | 0 |
| TRRAP | 1 | 0 | 1 | 0 | 0 | 0 |
| TRUB1 | 0 | 1 | 0 | 0 | 0 | 0 |
| TSC22D1 | 1 | 0 | 1 | 0 | 0 | 0 |
| TSC22D2 | 0 | 0 | 1 | 0 | 0 | 0 |
| TSC22D3 | 0 | 0 | 0 | 0 | 0 | 1 |
| TSEN15 | 0 | 0 | 0 | 0 | 1 | 0 |
| TSEN2 | 0 | 0 | 1 | 0 | 0 | 0 |
| TSFM | 0 | 0 | 0 | 0 | 1 | 0 |
| TSG101 | 0 | 0 | 0 | 0 | 1 | 0 |
| TSHZ1 | 1 | 0 | 0 | 0 | 1 | 0 |
| TSHZ2 | 0 | 0 | 1 | 0 | 0 | 0 |
| TSKU | 0 | 0 | 0 | 0 | 1 | 0 |
| TSN | 1 | 0 | 0 | 1 | 1 | 0 |
| TSNAX | 0 | 0 | 0 | 1 | 0 | 0 |
| TSPAN12 | 0 | 0 | 0 | 0 | 0 | 1 |
| TSPAN14 | 0 | 0 | 0 | 0 | 1 | 0 |
| TSPAN17 | 0 | 0 | 0 | 0 | 1 | 0 |
| TSPAN3 | 1 | 0 | 0 | 0 | 0 | 0 |
| TSPAN31 | 1 | 0 | 0 | 0 | 0 | 0 |
| TSPYL4 | 0 | 0 | 0 | 0 | 1 | 0 |
| TSR1 | 1 | 0 | 0 | 0 | 0 | 0 |
| TSR2 | 0 | 0 | 0 | 1 | 0 | 0 |
| TSTD1 | 0 | 0 | 0 | 0 | 0 | 1 |
| TTBK2 | 0 | 0 | 1 | 0 | 0 | 0 |
| TTC17 | 0 | 0 | 1 | 0 | 0 | 0 |
| TTC28 | 0 | 0 | 1 | 0 | 0 | 0 |
| TTC3 | 1 | 0 | 0 | 0 | 0 | 0 |
| TTC39C | 1 | 0 | 1 | 0 | 0 | 0 |
| TTC40 | 0 | 0 | 0 | 0 | 1 | 0 |
| TTF2 | 0 | 0 | 1 | 0 | 0 | 0 |
| TTLL10 | 0 | 0 | 0 | 0 | 1 | 0 |
| TTLL3 | 0 | 0 | 1 | 0 | 0 | 0 |
| TTLL9 | 0 | 0 | 0 | 0 | 0 | 1 |
| TUBA1A | 1 | 0 | 0 | 0 | 1 | 0 |
| TUBA1B | 1 | 0 | 0 | 0 | 0 | 0 |
| TUBA1C | 1 | 1 | 1 | 0 | 0 | 0 |
| TUBA4B | 0 | 0 | 0 | 0 | 1 | 0 |
| TUBB | 1 | 0 | 1 | 1 | 0 | 0 |
| TUBB3 | 1 | 0 | 0 | 0 | 0 | 0 |
| TUBB4B | 1 | 0 | 0 | 0 | 0 | 0 |
| TUBB7P | 0 | 0 | 0 | 0 | 1 | 0 |
| TUBD1 | 0 | 0 | 0 | 0 | 1 | 0 |
| TUBGCP3 | 0 | 0 | 1 | 0 | 0 | 0 |
| TUBGCP6 | 0 | 1 | 0 | 0 | 0 | 0 |
| TUFM | 1 | 0 | 0 | 0 | 0 | 0 |
| TUG1 | 0 | 0 | 1 | 0 | 0 | 0 |
| TULP4 | 1 | 1 | 0 | 0 | 1 | 0 |
| TUSC2 | 0 | 0 | 0 | 0 | 1 | 0 |
| TVP23B | 1 | 0 | 0 | 0 | 0 | 0 |
| TWF1 | 1 | 0 | 0 | 0 | 0 | 0 |
| TWISTNB | 1 | 0 | 0 | 0 | 1 | 0 |
| TWSG1 | 1 | 0 | 0 | 1 | 0 | 0 |
| TXLNA | 1 | 0 | 0 | 0 | 0 | 0 |
| TXLNG | 0 | 0 | 1 | 0 | 0 | 0 |
| TXN | 1 | 0 | 1 | 0 | 0 | 0 |
| TXN2 | 0 | 0 | 0 | 0 | 1 | 0 |
| TXNDC11 | 0 | 0 | 1 | 0 | 0 | 0 |
| TXNDC17 | 1 | 0 | 0 | 1 | 1 | 0 |
| TXNDC5 | 1 | 0 | 1 | 0 | 0 | 0 |
| TXNDC9 | 0 | 0 | 0 | 1 | 0 | 0 |
| TXNIP | 0 | 0 | 1 | 0 | 0 | 0 |
| TXNL4A | 0 | 0 | 0 | 1 | 1 | 0 |
| TXNRD1 | 1 | 0 | 0 | 0 | 0 | 0 |
| TXNRD3 | 0 | 0 | 0 | 0 | 1 | 0 |
| TXNRD3NB | 0 | 0 | 0 | 0 | 1 | 0 |
| TYMS | 1 | 0 | 0 | 0 | 0 | 0 |
| TYW1 | 0 | 0 | 1 | 0 | 0 | 0 |
| TYW1B | 0 | 0 | 1 | 0 | 0 | 0 |
| U1 | 0 | 0 | 0 | 0 | 1 | 1 |
| U11 | 0 | 0 | 0 | 0 | 1 | 0 |
| U2 | 0 | 0 | 0 | 0 | 0 | 1 |
| U2AF2 | 1 | 0 | 1 | 0 | 0 | 0 |
| U2SURP | 1 | 0 | 1 | 0 | 0 | 0 |
| U3 | 0 | 0 | 0 | 0 | 1 | 1 |
| U4 | 0 | 0 | 0 | 0 | 1 | 0 |
| U47924.19 | 0 | 0 | 0 | 0 | 1 | 0 |
| U6 | 0 | 0 | 0 | 0 | 1 | 1 |
| U7 | 0 | 0 | 0 | 0 | 1 | 0 |
| U8 | 0 | 0 | 0 | 0 | 1 | 0 |
| U91328.2 | 0 | 0 | 0 | 0 | 1 | 0 |
| UAP1 | 0 | 0 | 0 | 1 | 0 | 0 |
| UBA1 | 1 | 0 | 0 | 0 | 0 | 0 |
| UBA2 | 1 | 0 | 0 | 1 | 0 | 0 |
| UBA3 | 0 | 0 | 0 | 1 | 0 | 0 |
| UBA52 | 1 | 0 | 0 | 0 | 0 | 0 |
| UBAC2 | 0 | 0 | 1 | 0 | 1 | 0 |
| UBAC2-IT1 | 0 | 0 | 0 | 0 | 1 | 0 |
| UBAP1 | 0 | 0 | 1 | 1 | 0 | 0 |
| UBAP2 | 0 | 0 | 1 | 0 | 0 | 0 |
| UBAP2L | 1 | 0 | 1 | 0 | 1 | 0 |
| UBB | 1 | 0 | 0 | 0 | 0 | 0 |
| UBC | 1 | 0 | 0 | 0 | 0 | 0 |
| UBE2A | 1 | 0 | 0 | 1 | 1 | 0 |
| UBE2B | 0 | 0 | 0 | 1 | 0 | 0 |
| UBE2C | 1 | 0 | 1 | 0 | 0 | 0 |
| UBE2D1 | 0 | 0 | 0 | 1 | 1 | 0 |
| UBE2D2 | 0 | 0 | 1 | 1 | 0 | 0 |
| UBE2D3 | 0 | 0 | 1 | 1 | 0 | 0 |
| UBE2E1 | 1 | 0 | 0 | 1 | 0 | 0 |
| UBE2E2 | 0 | 0 | 1 | 0 | 0 | 0 |
| UBE2E3 | 1 | 0 | 0 | 1 | 1 | 0 |
| UBE2F | 0 | 0 | 0 | 1 | 0 | 0 |
| UBE2G1 | 1 | 0 | 0 | 1 | 0 | 0 |
| UBE2H | 0 | 0 | 1 | 0 | 1 | 0 |
| UBE2I | 0 | 0 | 0 | 0 | 1 | 0 |
| UBE2J1 | 0 | 0 | 0 | 1 | 0 | 0 |
| UBE2J2 | 0 | 0 | 0 | 0 | 1 | 0 |
| UBE2K | 1 | 0 | 1 | 1 | 0 | 0 |
| UBE2L3 | 0 | 0 | 1 | 0 | 0 | 0 |
| UBE2L6 | 0 | 0 | 0 | 1 | 1 | 0 |
| UBE2M | 1 | 0 | 0 | 0 | 0 | 0 |
| UBE2N | 1 | 0 | 0 | 1 | 0 | 0 |
| UBE2R2 | 0 | 0 | 1 | 1 | 0 | 0 |
| UBE2V1 | 0 | 0 | 1 | 0 | 0 | 0 |
| UBE2V2 | 0 | 0 | 1 | 1 | 0 | 0 |
| UBE2W | 1 | 1 | 0 | 0 | 1 | 0 |
| UBE2Z | 1 | 0 | 1 | 0 | 1 | 0 |
| UBE3A | 1 | 0 | 1 | 1 | 0 | 0 |
| UBE3C | 0 | 0 | 1 | 0 | 0 | 0 |
| UBE4A | 0 | 0 | 0 | 1 | 0 | 0 |
| UBIAD1 | 0 | 0 | 0 | 0 | 1 | 0 |
| UBL5 | 1 | 0 | 0 | 0 | 0 | 0 |
| UBLCP1 | 1 | 0 | 1 | 1 | 1 | 0 |
| UBN2 | 1 | 0 | 1 | 0 | 0 | 0 |
| UBOX5 | 0 | 0 | 0 | 0 | 1 | 0 |
| UBP1 | 0 | 0 | 0 | 1 | 0 | 0 |
| UBQLN1 | 1 | 0 | 0 | 1 | 0 | 0 |
| UBR7 | 0 | 0 | 0 | 0 | 1 | 0 |
| UBXN7 | 1 | 0 | 1 | 0 | 0 | 0 |
| UCHL5 | 0 | 0 | 1 | 0 | 0 | 0 |
| UCK1 | 0 | 0 | 0 | 0 | 1 | 0 |
| UCK2 | 0 | 0 | 0 | 0 | 1 | 0 |
| UFM1 | 0 | 0 | 0 | 1 | 1 | 0 |
| UGCG | 1 | 0 | 1 | 0 | 1 | 0 |
| UGDH | 1 | 0 | 0 | 0 | 0 | 0 |
| UGGT1 | 1 | 0 | 1 | 0 | 0 | 0 |
| UGGT2 | 0 | 0 | 1 | 0 | 0 | 0 |
| UGT8 | 1 | 0 | 0 | 0 | 1 | 0 |
| UHMK1 | 1 | 0 | 0 | 0 | 0 | 0 |
| UMPS | 0 | 0 | 0 | 0 | 1 | 0 |
| UNC119B | 0 | 0 | 0 | 0 | 1 | 0 |
| UNC13A | 0 | 1 | 0 | 0 | 0 | 0 |
| UNC45B | 1 | 0 | 0 | 0 | 0 | 0 |
| UNC50 | 0 | 0 | 0 | 0 | 1 | 0 |
| UNC5B | 0 | 0 | 1 | 0 | 0 | 0 |
| UNC5D | 1 | 0 | 0 | 0 | 0 | 0 |
| UNK | 0 | 0 | 0 | 0 | 1 | 0 |
| UPF3A | 0 | 0 | 1 | 0 | 0 | 0 |
| UPF3B | 1 | 0 | 0 | 0 | 0 | 0 |
| UQCR10 | 1 | 0 | 0 | 0 | 0 | 0 |
| UQCR11 | 1 | 0 | 1 | 0 | 0 | 0 |
| UQCRB | 0 | 0 | 0 | 0 | 1 | 0 |
| UQCRC2 | 0 | 0 | 0 | 1 | 0 | 0 |
| UQCRFS1 | 1 | 0 | 0 | 0 | 1 | 0 |
| UQCRH | 1 | 0 | 0 | 0 | 0 | 0 |
| UQCRQ | 1 | 0 | 0 | 0 | 0 | 0 |
| URB2 | 0 | 0 | 0 | 0 | 1 | 0 |
| USF1P1 | 0 | 0 | 0 | 0 | 1 | 0 |
| USH1G | 0 | 1 | 0 | 0 | 1 | 0 |
| USMG5 | 1 | 0 | 0 | 0 | 0 | 0 |
| USP1 | 0 | 0 | 1 | 1 | 0 | 0 |
| USP12 | 0 | 0 | 1 | 0 | 1 | 0 |
| USP13 | 1 | 0 | 0 | 0 | 0 | 0 |
| USP22 | 1 | 1 | 0 | 0 | 1 | 0 |
| USP24 | 0 | 0 | 1 | 0 | 0 | 0 |
| USP3 | 0 | 0 | 1 | 0 | 0 | 0 |
| USP30 | 0 | 1 | 0 | 0 | 1 | 0 |
| USP33 | 1 | 0 | 1 | 1 | 0 | 0 |
| USP34 | 1 | 0 | 1 | 1 | 0 | 0 |
| USP37 | 1 | 0 | 0 | 0 | 0 | 1 |
| USP38 | 0 | 0 | 0 | 1 | 0 | 0 |
| USP42 | 0 | 0 | 0 | 1 | 1 | 0 |
| USP51 | 0 | 0 | 0 | 0 | 1 | 0 |
| USP7 | 1 | 0 | 0 | 0 | 0 | 0 |
| USP8 | 0 | 0 | 0 | 1 | 0 | 0 |
| USP9X | 0 | 0 | 1 | 0 | 0 | 0 |
| UTP11L | 1 | 0 | 0 | 0 | 0 | 0 |
| UTP14C | 0 | 0 | 0 | 0 | 1 | 1 |
| UTP15 | 0 | 0 | 0 | 0 | 1 | 0 |
| UTP23 | 1 | 0 | 0 | 0 | 0 | 0 |
| UTX | 0 | 1 | 0 | 0 | 0 | 0 |
| UVSSA | 1 | 0 | 0 | 0 | 0 | 0 |
| VAC14 | 0 | 1 | 0 | 0 | 0 | 0 |
| VAMP2 | 0 | 1 | 1 | 1 | 0 | 0 |
| VAMP3 | 1 | 0 | 0 | 1 | 1 | 0 |
| VAMP7 | 0 | 0 | 0 | 1 | 1 | 0 |
| VANGL1 | 0 | 0 | 1 | 0 | 1 | 0 |
| VAPA | 1 | 0 | 1 | 1 | 0 | 0 |
| VAPB | 1 | 0 | 0 | 0 | 1 | 0 |
| VAT1 | 1 | 0 | 1 | 0 | 0 | 0 |
| VAV2 | 1 | 0 | 0 | 0 | 0 | 0 |
| VDAC1 | 1 | 0 | 0 | 1 | 0 | 0 |
| VDAC1P4 | 0 | 0 | 0 | 0 | 1 | 0 |
| VDAC2 | 1 | 0 | 0 | 0 | 0 | 0 |
| VEGFC | 0 | 0 | 0 | 0 | 1 | 0 |
| VEZF1 | 1 | 0 | 0 | 0 | 0 | 0 |
| VGLL4 | 1 | 0 | 0 | 0 | 0 | 0 |
| VHL | 1 | 0 | 0 | 0 | 1 | 1 |
| VIM | 1 | 0 | 0 | 0 | 0 | 0 |
| VKORC1L1 | 0 | 0 | 1 | 1 | 1 | 0 |
| VMA21 | 1 | 0 | 0 | 1 | 0 | 0 |
| VMP1 | 0 | 0 | 1 | 0 | 1 | 0 |
| VPS13A | 0 | 0 | 1 | 0 | 0 | 0 |
| VPS13C | 0 | 0 | 1 | 0 | 0 | 0 |
| VPS24 | 0 | 0 | 0 | 1 | 0 | 0 |
| VPS26A | 1 | 0 | 0 | 0 | 0 | 0 |
| VPS29 | 1 | 0 | 0 | 1 | 0 | 0 |
| VPS35 | 1 | 0 | 1 | 0 | 1 | 0 |
| VPS36 | 1 | 0 | 0 | 0 | 0 | 0 |
| VPS54 | 0 | 0 | 0 | 1 | 0 | 0 |
| VPS72 | 0 | 0 | 0 | 1 | 0 | 0 |
| VPS9D1-AS1 | 0 | 0 | 1 | 0 | 0 | 0 |
| VRK1 | 0 | 0 | 1 | 0 | 0 | 0 |
| VSX2 | 1 | 0 | 0 | 0 | 0 | 0 |
| VTI1A | 0 | 0 | 1 | 0 | 0 | 0 |
| VTRNA1-1 | 0 | 0 | 1 | 0 | 0 | 0 |
| VWA8 | 0 | 0 | 1 | 0 | 0 | 0 |
| WAC | 0 | 0 | 1 | 1 | 0 | 0 |
| WARS | 0 | 0 | 1 | 1 | 0 | 0 |
| WASF1 | 0 | 0 | 0 | 0 | 1 | 0 |
| WASL | 0 | 0 | 1 | 0 | 0 | 0 |
| WBP11 | 0 | 0 | 0 | 1 | 0 | 0 |
| WBP4 | 0 | 0 | 0 | 0 | 1 | 0 |
| WBP5 | 1 | 0 | 0 | 0 | 0 | 0 |
| WDFY4 | 1 | 0 | 0 | 0 | 0 | 0 |
| WDR1 | 1 | 0 | 0 | 0 | 0 | 0 |
| WDR11 | 0 | 0 | 1 | 0 | 0 | 0 |
| WDR12 | 0 | 0 | 0 | 0 | 1 | 0 |
| WDR27 | 0 | 0 | 1 | 0 | 0 | 0 |
| WDR3 | 1 | 0 | 0 | 0 | 0 | 0 |
| WDR34 | 0 | 0 | 0 | 0 | 1 | 0 |
| WDR36 | 1 | 0 | 0 | 0 | 0 | 0 |
| WDR37 | 1 | 0 | 1 | 0 | 1 | 0 |
| WDR4 | 1 | 0 | 0 | 0 | 0 | 0 |
| WDR42A | 0 | 0 | 0 | 0 | 1 | 0 |
| WDR44 | 0 | 0 | 1 | 1 | 0 | 0 |
| WDR45B | 0 | 0 | 1 | 0 | 0 | 0 |
| WDR5 | 0 | 0 | 0 | 0 | 1 | 0 |
| WDR51B | 0 | 0 | 0 | 1 | 0 | 0 |
| WDR54 | 0 | 0 | 0 | 0 | 1 | 0 |
| WDR59 | 0 | 1 | 0 | 0 | 0 | 0 |
| WDR5B | 0 | 0 | 0 | 0 | 1 | 0 |
| WDR6 | 0 | 0 | 0 | 0 | 1 | 0 |
| WDR68 | 0 | 0 | 0 | 1 | 0 | 0 |
| WDR74 | 1 | 0 | 1 | 0 | 0 | 0 |
| WDR77 | 1 | 0 | 0 | 0 | 1 | 0 |
| WDR82 | 1 | 0 | 1 | 0 | 1 | 0 |
| WDR82P1 | 0 | 0 | 0 | 0 | 1 | 0 |
| WDR83OS | 0 | 0 | 0 | 0 | 1 | 0 |
| WDR92 | 0 | 0 | 0 | 0 | 1 | 0 |
| WDTC1 | 0 | 0 | 0 | 0 | 1 | 0 |
| WEE1 | 1 | 0 | 0 | 0 | 1 | 0 |
| WFDC1 | 0 | 0 | 1 | 0 | 0 | 0 |
| WFDC12 | 0 | 0 | 0 | 0 | 1 | 0 |
| WHSC1 | 0 | 0 | 1 | 0 | 0 | 0 |
| WIBG | 0 | 0 | 0 | 1 | 0 | 0 |
| WIPF2 | 1 | 0 | 0 | 0 | 1 | 0 |
| WIPI1 | 0 | 0 | 0 | 1 | 0 | 0 |
| WIPI2 | 0 | 1 | 1 | 0 | 0 | 0 |
| WNK1 | 1 | 0 | 0 | 1 | 0 | 0 |
| WSB1 | 0 | 0 | 0 | 1 | 0 | 0 |
| WSB2 | 1 | 0 | 0 | 1 | 0 | 0 |
| WT1 | 0 | 0 | 0 | 0 | 1 | 0 |
| WTAP | 1 | 1 | 0 | 0 | 0 | 0 |
| WWC2 | 0 | 0 | 1 | 0 | 0 | 0 |
| WWP1 | 0 | 0 | 0 | 1 | 0 | 0 |
| WWTR1 | 1 | 0 | 1 | 0 | 0 | 0 |
| XAF1 | 0 | 0 | 0 | 1 | 0 | 0 |
| XCL1 | 0 | 0 | 0 | 1 | 0 | 0 |
| XGPY2 | 0 | 1 | 0 | 0 | 0 | 0 |
| XIAP | 1 | 0 | 1 | 1 | 1 | 1 |
| XK | 0 | 0 | 0 | 0 | 1 | 0 |
| XPNPEP3 | 0 | 0 | 0 | 0 | 1 | 0 |
| XPO1 | 1 | 0 | 1 | 1 | 0 | 0 |
| XPO4 | 1 | 0 | 1 | 0 | 0 | 0 |
| XPO5 | 0 | 0 | 1 | 0 | 0 | 0 |
| XPOT | 1 | 0 | 0 | 1 | 0 | 0 |
| XPR1 | 0 | 0 | 1 | 0 | 0 | 0 |
| XRCC2 | 1 | 0 | 1 | 0 | 0 | 0 |
| XRCC5 | 1 | 0 | 0 | 0 | 0 | 0 |
| XRCC6 | 1 | 0 | 0 | 0 | 0 | 0 |
| XRN1 | 1 | 0 | 1 | 1 | 0 | 0 |
| XXbac-BPG116M5.15 | 0 | 0 | 0 | 0 | 1 | 0 |
| XYLB | 0 | 0 | 1 | 0 | 0 | 0 |
| Y_RNA | 0 | 0 | 0 | 0 | 1 | 1 |
| YAE1D1 | 0 | 0 | 0 | 0 | 1 | 0 |
| YAP1 | 1 | 0 | 0 | 0 | 0 | 0 |
| YARS | 0 | 0 | 1 | 1 | 0 | 0 |
| YBX1 | 1 | 0 | 0 | 1 | 0 | 0 |
| YBX3 | 1 | 0 | 0 | 0 | 0 | 0 |
| YEATS2 | 0 | 0 | 1 | 0 | 0 | 0 |
| YIPF4 | 0 | 0 | 0 | 0 | 1 | 0 |
| YIPF5 | 0 | 0 | 0 | 1 | 1 | 0 |
| YJEFN3 | 1 | 0 | 0 | 0 | 0 | 0 |
| YLPM1 | 0 | 0 | 1 | 0 | 0 | 0 |
| YME1L1 | 0 | 0 | 0 | 1 | 0 | 0 |
| YOD1 | 1 | 0 | 0 | 0 | 0 | 0 |
| YPEL1 | 1 | 0 | 0 | 0 | 0 | 0 |
| YPEL2 | 0 | 0 | 0 | 0 | 0 | 1 |
| YPEL3 | 0 | 0 | 0 | 0 | 1 | 0 |
| YPEL5 | 0 | 0 | 0 | 1 | 1 | 0 |
| YRDC | 0 | 0 | 0 | 0 | 1 | 0 |
| YTHDF2 | 1 | 0 | 0 | 0 | 0 | 0 |
| YTHDF3 | 0 | 0 | 0 | 0 | 1 | 0 |
| YWHAB | 1 | 0 | 1 | 0 | 1 | 0 |
| YWHAE | 1 | 0 | 1 | 0 | 0 | 0 |
| YWHAG | 1 | 0 | 0 | 0 | 0 | 0 |
| YWHAQ | 1 | 0 | 0 | 1 | 0 | 0 |
| YWHAZ | 1 | 0 | 1 | 1 | 0 | 0 |
| YY1 | 1 | 0 | 0 | 1 | 0 | 0 |
| YY1AP1 | 0 | 0 | 0 | 1 | 0 | 0 |
| Z69890.1 | 0 | 0 | 0 | 0 | 1 | 0 |
| ZBED4 | 0 | 0 | 0 | 0 | 1 | 0 |
| ZBED5 | 0 | 0 | 0 | 0 | 1 | 0 |
| ZBTB2 | 0 | 0 | 0 | 1 | 0 | 0 |
| ZBTB20 | 0 | 0 | 1 | 0 | 0 | 0 |
| ZBTB22 | 0 | 0 | 0 | 0 | 1 | 0 |
| ZBTB33 | 1 | 0 | 0 | 0 | 1 | 0 |
| ZBTB34 | 0 | 0 | 0 | 0 | 1 | 0 |
| ZBTB38 | 0 | 0 | 0 | 1 | 0 | 0 |
| ZBTB39 | 0 | 0 | 0 | 0 | 1 | 0 |
| ZBTB41 | 0 | 0 | 0 | 1 | 0 | 0 |
| ZBTB44 | 0 | 0 | 0 | 1 | 0 | 0 |
| ZBTB49 | 0 | 0 | 1 | 0 | 0 | 0 |
| ZBTB5 | 0 | 0 | 0 | 0 | 1 | 0 |
| ZBTB7C | 0 | 0 | 0 | 0 | 1 | 0 |
| ZC3H11A | 1 | 0 | 1 | 1 | 0 | 0 |
| ZC3H13 | 1 | 0 | 1 | 0 | 0 | 0 |
| ZC3H14 | 0 | 0 | 1 | 0 | 0 | 0 |
| ZC3H15 | 1 | 0 | 0 | 1 | 0 | 0 |
| ZC3H6 | 0 | 0 | 0 | 0 | 1 | 0 |
| ZC3H8 | 0 | 0 | 0 | 1 | 0 | 0 |
| ZC3HAV1 | 0 | 0 | 1 | 0 | 0 | 0 |
| ZC3HAV1L | 0 | 0 | 0 | 1 | 0 | 0 |
| ZCCHC10 | 0 | 0 | 0 | 0 | 1 | 0 |
| ZCCHC2 | 0 | 0 | 1 | 0 | 1 | 0 |
| ZCCHC24 | 0 | 0 | 1 | 0 | 1 | 0 |
| ZCCHC3 | 1 | 0 | 0 | 0 | 1 | 0 |
| ZCCHC6 | 0 | 1 | 0 | 0 | 0 | 0 |
| ZCCHC7 | 0 | 0 | 1 | 0 | 0 | 0 |
| ZCWPW1 | 0 | 0 | 0 | 0 | 1 | 0 |
| ZDHHC11 | 0 | 0 | 1 | 0 | 0 | 0 |
| ZDHHC13 | 0 | 0 | 0 | 0 | 1 | 0 |
| ZDHHC15 | 0 | 0 | 0 | 0 | 1 | 0 |
| ZDHHC17 | 0 | 1 | 0 | 0 | 0 | 0 |
| ZDHHC2 | 0 | 0 | 0 | 0 | 1 | 0 |
| ZDHHC20 | 0 | 0 | 1 | 1 | 1 | 0 |
| ZDHHC20-IT1 | 0 | 0 | 0 | 0 | 1 | 0 |
| ZDHHC21 | 0 | 0 | 0 | 0 | 0 | 1 |
| ZDHHC3 | 0 | 0 | 1 | 0 | 0 | 0 |
| ZDHHC5 | 0 | 0 | 0 | 0 | 1 | 0 |
| ZDHHC8 | 0 | 0 | 0 | 0 | 1 | 0 |
| ZEB1 | 0 | 0 | 1 | 0 | 0 | 0 |
| ZFAND2A | 0 | 0 | 0 | 0 | 1 | 0 |
| ZFAND3 | 0 | 0 | 1 | 0 | 0 | 0 |
| ZFAND5 | 1 | 0 | 0 | 1 | 0 | 0 |
| ZFAND6 | 1 | 0 | 1 | 1 | 1 | 0 |
| ZFAS1 | 0 | 0 | 1 | 0 | 0 | 0 |
| ZFHX3 | 1 | 0 | 1 | 0 | 0 | 0 |
| ZFP161 | 0 | 0 | 0 | 0 | 1 | 0 |
| ZFP3 | 0 | 0 | 0 | 0 | 1 | 0 |
| ZFP36L1 | 1 | 0 | 0 | 1 | 0 | 0 |
| ZFP90 | 0 | 0 | 0 | 1 | 1 | 0 |
| ZFP91 | 1 | 0 | 0 | 0 | 0 | 0 |
| ZFPL1 | 0 | 1 | 0 | 0 | 0 | 0 |
| ZFR | 1 | 0 | 1 | 0 | 0 | 0 |
| ZFX | 1 | 0 | 1 | 0 | 1 | 0 |
| ZFYVE21 | 0 | 0 | 0 | 0 | 1 | 0 |
| ZHX1-C8ORF76 | 0 | 0 | 0 | 0 | 1 | 0 |
| ZIC5 | 0 | 0 | 1 | 0 | 0 | 0 |
| ZKSCAN1 | 0 | 0 | 1 | 0 | 0 | 0 |
| ZMAT2 | 1 | 0 | 1 | 1 | 0 | 0 |
| ZMAT3 | 1 | 0 | 1 | 0 | 0 | 0 |
| ZMIZ1 | 1 | 0 | 0 | 0 | 0 | 0 |
| ZMPSTE24 | 1 | 0 | 1 | 0 | 1 | 0 |
| ZMYM2 | 0 | 0 | 1 | 1 | 0 | 0 |
| ZMYM3 | 0 | 0 | 1 | 0 | 0 | 0 |
| ZMYND11 | 0 | 0 | 0 | 1 | 0 | 0 |
| ZMYND19 | 0 | 0 | 0 | 0 | 1 | 0 |
| ZNF114 | 1 | 0 | 0 | 0 | 0 | 0 |
| ZNF117 | 0 | 1 | 0 | 0 | 0 | 0 |
| ZNF136 | 0 | 0 | 0 | 0 | 1 | 0 |
| ZNF140 | 0 | 1 | 0 | 0 | 0 | 0 |
| ZNF146 | 1 | 0 | 1 | 0 | 1 | 0 |
| ZNF148 | 0 | 0 | 1 | 0 | 0 | 0 |
| ZNF160 | 0 | 0 | 0 | 1 | 0 | 0 |
| ZNF180 | 0 | 0 | 0 | 0 | 1 | 0 |
| ZNF189 | 0 | 0 | 1 | 0 | 0 | 0 |
| ZNF192 | 0 | 0 | 0 | 0 | 1 | 0 |
| ZNF195 | 0 | 0 | 1 | 0 | 0 | 0 |
| ZNF197 | 0 | 0 | 0 | 0 | 1 | 0 |
| ZNF207 | 0 | 0 | 1 | 0 | 0 | 0 |
| ZNF217 | 0 | 0 | 1 | 0 | 1 | 0 |
| ZNF219 | 0 | 0 | 1 | 0 | 0 | 0 |
| ZNF22 | 0 | 0 | 0 | 1 | 1 | 0 |
| ZNF238 | 0 | 0 | 0 | 0 | 1 | 0 |
| ZNF250 | 0 | 0 | 0 | 0 | 1 | 0 |
| ZNF254 | 0 | 0 | 1 | 0 | 0 | 0 |
| ZNF257 | 0 | 0 | 0 | 0 | 1 | 0 |
| ZNF264 | 0 | 0 | 0 | 0 | 1 | 1 |
| ZNF267 | 0 | 0 | 0 | 1 | 0 | 0 |
| ZNF275 | 0 | 0 | 0 | 0 | 1 | 0 |
| ZNF276 | 0 | 1 | 1 | 0 | 0 | 0 |
| ZNF280C | 0 | 0 | 0 | 1 | 0 | 0 |
| ZNF280D | 1 | 0 | 0 | 0 | 0 | 0 |
| ZNF282 | 0 | 0 | 1 | 0 | 0 | 0 |
| ZNF286A | 0 | 0 | 0 | 0 | 1 | 0 |
| ZNF286B | 0 | 0 | 0 | 0 | 1 | 0 |
| ZNF292 | 0 | 0 | 0 | 1 | 0 | 0 |
| ZNF300 | 0 | 0 | 0 | 0 | 1 | 0 |
| ZNF302 | 0 | 0 | 0 | 1 | 0 | 0 |
| ZNF304 | 0 | 0 | 0 | 0 | 1 | 0 |
| ZNF320 | 0 | 0 | 0 | 0 | 1 | 0 |
| ZNF322 | 0 | 0 | 0 | 0 | 1 | 0 |
| ZNF333 | 0 | 0 | 0 | 0 | 1 | 0 |
| ZNF337 | 0 | 0 | 1 | 0 | 0 | 0 |
| ZNF33A | 0 | 0 | 1 | 0 | 1 | 0 |
| ZNF33B | 0 | 0 | 0 | 0 | 1 | 0 |
| ZNF346 | 1 | 0 | 0 | 0 | 0 | 0 |
| ZNF350 | 0 | 0 | 0 | 0 | 1 | 0 |
| ZNF37A | 1 | 0 | 0 | 0 | 0 | 0 |
| ZNF383 | 0 | 0 | 0 | 0 | 1 | 0 |
| ZNF404 | 0 | 0 | 0 | 0 | 0 | 1 |
| ZNF407 | 0 | 0 | 1 | 0 | 0 | 0 |
| ZNF417 | 1 | 0 | 0 | 0 | 0 | 0 |
| ZNF428 | 0 | 0 | 0 | 0 | 1 | 0 |
| ZNF436 | 0 | 0 | 0 | 0 | 1 | 0 |
| ZNF451 | 1 | 0 | 1 | 1 | 0 | 0 |
| ZNF460 | 1 | 0 | 1 | 0 | 0 | 0 |
| ZNF471 | 1 | 0 | 0 | 0 | 0 | 0 |
| ZNF483 | 1 | 0 | 0 | 0 | 0 | 0 |
| ZNF490 | 0 | 0 | 0 | 0 | 1 | 0 |
| ZNF501 | 0 | 0 | 0 | 0 | 1 | 0 |
| ZNF518B | 0 | 0 | 0 | 1 | 0 | 0 |
| ZNF532 | 1 | 0 | 0 | 0 | 0 | 0 |
| ZNF536 | 0 | 0 | 1 | 0 | 0 | 0 |
| ZNF540 | 0 | 0 | 0 | 0 | 1 | 0 |
| ZNF542 | 0 | 0 | 0 | 0 | 1 | 0 |
| ZNF548 | 0 | 0 | 1 | 0 | 0 | 0 |
| ZNF560 | 0 | 1 | 0 | 0 | 0 | 0 |
| ZNF562 | 0 | 0 | 0 | 0 | 1 | 0 |
| ZNF565 | 1 | 0 | 0 | 0 | 0 | 0 |
| ZNF573 | 0 | 1 | 0 | 0 | 1 | 1 |
| ZNF574 | 0 | 0 | 0 | 0 | 1 | 0 |
| ZNF579 | 0 | 0 | 1 | 0 | 0 | 0 |
| ZNF583 | 0 | 0 | 0 | 0 | 1 | 0 |
| ZNF585A | 0 | 0 | 0 | 0 | 1 | 0 |
| ZNF585B | 0 | 0 | 0 | 0 | 1 | 0 |
| ZNF587B | 1 | 0 | 0 | 0 | 0 | 0 |
| ZNF609 | 0 | 0 | 1 | 0 | 0 | 0 |
| ZNF611 | 1 | 0 | 0 | 0 | 0 | 0 |
| ZNF621 | 0 | 0 | 1 | 0 | 0 | 0 |
| ZNF623 | 1 | 0 | 0 | 0 | 1 | 0 |
| ZNF627 | 0 | 0 | 0 | 0 | 1 | 0 |
| ZNF638 | 0 | 0 | 0 | 1 | 0 | 0 |
| ZNF644 | 0 | 0 | 0 | 1 | 0 | 0 |
| ZNF652 | 1 | 0 | 0 | 0 | 0 | 0 |
| ZNF655 | 1 | 0 | 1 | 0 | 1 | 0 |
| ZNF664 | 1 | 0 | 0 | 0 | 0 | 0 |
| ZNF669 | 0 | 0 | 0 | 0 | 1 | 0 |
| ZNF670 | 0 | 0 | 0 | 0 | 1 | 0 |
| ZNF678 | 0 | 1 | 0 | 0 | 0 | 0 |
| ZNF691 | 0 | 0 | 0 | 0 | 1 | 0 |
| ZNF697 | 1 | 0 | 0 | 0 | 0 | 0 |
| ZNF703 | 1 | 0 | 0 | 0 | 0 | 0 |
| ZNF706 | 1 | 0 | 0 | 0 | 1 | 0 |
| ZNF718 | 0 | 0 | 0 | 0 | 0 | 1 |
| ZNF720 | 0 | 0 | 0 | 0 | 1 | 0 |
| ZNF726 | 0 | 0 | 0 | 0 | 1 | 0 |
| ZNF736 | 0 | 0 | 1 | 0 | 0 | 0 |
| ZNF738 | 0 | 0 | 0 | 0 | 1 | 0 |
| ZNF746 | 0 | 0 | 1 | 0 | 0 | 0 |
| ZNF75D | 0 | 0 | 0 | 0 | 1 | 0 |
| ZNF76 | 0 | 0 | 1 | 0 | 0 | 0 |
| ZNF764 | 0 | 0 | 0 | 0 | 1 | 0 |
| ZNF77 | 0 | 0 | 0 | 0 | 1 | 0 |
| ZNF770 | 1 | 0 | 0 | 1 | 0 | 0 |
| ZNF772 | 1 | 0 | 0 | 0 | 0 | 1 |
| ZNF782 | 0 | 0 | 1 | 0 | 0 | 0 |
| ZNF791 | 0 | 0 | 0 | 1 | 0 | 0 |
| ZNF793 | 1 | 0 | 0 | 0 | 0 | 0 |
| ZNF80 | 0 | 0 | 0 | 0 | 0 | 1 |
| ZNF800 | 0 | 0 | 1 | 0 | 0 | 0 |
| ZNF805 | 1 | 0 | 0 | 0 | 1 | 0 |
| ZNF828 | 0 | 0 | 0 | 1 | 0 | 0 |
| ZNF830 | 0 | 0 | 0 | 0 | 1 | 0 |
| ZNF831 | 0 | 0 | 1 | 0 | 0 | 0 |
| ZNF84 | 0 | 1 | 0 | 0 | 0 | 0 |
| ZNF85 | 0 | 0 | 0 | 0 | 1 | 0 |
| ZNF852 | 0 | 0 | 1 | 0 | 0 | 0 |
| ZNFX1-AS1 | 0 | 0 | 0 | 0 | 1 | 0 |
| ZNRF2 | 0 | 0 | 1 | 0 | 0 | 0 |
| ZP3 | 0 | 0 | 1 | 0 | 0 | 0 |
| ZRANB2 | 1 | 0 | 0 | 1 | 0 | 0 |
| ZSCAN22 | 0 | 0 | 0 | 0 | 1 | 0 |
| ZSCAN23 | 0 | 0 | 0 | 0 | 0 | 1 |
| ZSWIM6 | 0 | 0 | 1 | 0 | 0 | 0 |
| ZWINT | 1 | 0 | 0 | 0 | 1 | 0 |
| ZXDB | 0 | 0 | 0 | 0 | 1 | 0 |
| ZYG11A | 0 | 0 | 1 | 0 | 0 | 0 |
| ZYX | 0 | 0 | 1 | 0 | 0 | 0 |
| ZZZ3 | 0 | 0 | 1 | 0 | 0 | 0 |
