# Supplementary material for: Systems analysis identifies melanoma-enriched pro-oncogenic networks controlled by the RNA binding protein CELF1
Source: Nat Commun. 2017 Dec 21;8:2249. doi: 10.1038/s41467-017-02353-y (PMC5740069; doi:10.1038/s41467-017-02353-y)
Supplement: Supplementary file 6 — Supplementary Data 4 [file 41467_2017_2353_MOESM6_ESM.docx]

| **CELF1-bound transcripts in melanoma cells identified by RIP-Seq *with* canonical CUG repeats** | | | |  | **CELF1-bound transcripts in melanoma cells identified by RIP-Seq *without* canonical CUG repeats** | | | |
| --- | --- | --- | --- | --- | --- | --- | --- | --- |
| **Introns** | **5UTR** | **CDS** | **3UTR** |  | **Introns** | **5UTR** | **CDS** | **3UTR** |
| SEPT2 | CRIPAK | SEPT2 | MARCH5 |  | AC087645.1 | AC015987.2 | MARCH6 | SEPT15 |
| SEPT8 | GPM6A | AC015987.2 | MARCH6 |  | AGXT2L2 | AC114546.1 | SEPT7 | AAK1 |
| SEPT9 | HSPA8 | AC119673.1 | MARCH7 |  | AL162431.1 | AC119673.1 | SEPT9 | AC006465.3 |
| SEPT11 | MBNL1 | AC120194.1 | SEPT2 |  | ALDOA | AC120194.1 | AC087645.1 | AC015987.2 |
| AAK1 | PTP4A2 | ACSL3 | SEPT7 |  | AP1M1 | ANXA2 | ACLY | AC073346.2 |
| ABHD12 | UGT8 | ACSL4 | SEPT8 |  | APH1A | ARSI | ACTR2 | AC104841.2 |
| ABR | ZC3H11A | ACTB | SEPT9 |  | ARHGDIA | BEND3 | ADPRHL1 | AC119673.1 |
| AC004076.9 |  | ACTG1 | SEPT11 |  | CERS2 | CALR | AKIRIN1 | AC120194.1 |
| AC007401.2 |  | ACTR3 | AASDHPPT |  | CNGA4 | CANX | ALDOA | ACAT2 |
| AC024592.12 |  | AHNAK | ABCC9 |  | COL6A2 | CSDE1 | AMD1 | ACOT13 |
| AC073610.5 |  | ANLN | ABHD2 |  | CTNNB1 | EIF3E | ANXA1 | ADAR |
| ACSM3 |  | AP1M1 | ABL2 |  | CTSB | EIF4A1 | ANXA2 | ADSS |
| ACTN4 |  | AP2S1 | ACBD3 |  | EI24 | ERO1L | ANXA5 | AGBL5 |
| AHNAK |  | ARL2BP | ACBD7 |  | EIF1 | FBLN2 | AP000350.10 | AHCY |
| AHRR |  | ARL6IP1 | ACLY |  | EIF4A1 | FTL | AP000350.4 | AKR1B1 |
| AK2 |  | ARPP19 | ACSL3 |  | ENOSF1 | G3BP1 | APH1A | ALDOA |
| AKIRIN1 |  | ATP13A3 | ACSL4 |  | FASTKD2 | GAPDH | ARF4 | AMD1 |
| AMY2B |  | ATP2A2 | ACTB |  | GLRX3 | HES6 | ARHGDIA | ANAPC13 |
| ANXA2 |  | ATP5B | ACTG1 |  | GPS2 | HN1 | ARPC2 | ANXA1 |
| ANXA5 |  | ATP5E | ACTR2 |  | HDGF | HNRPLL | ARPC5 | ANXA2 |
| AP2S1 |  | ATP5F1 | ACTR3 |  | HES6 | HSP90AA1 | ATAD1 | ANXA5 |
| ARF3 |  | ATP5G3 | ADAM10 |  | HSP90B1 | KPNB1 | ATF4 | AP000350.10 |
| ARF4 |  | ATP5L | ADAM9 |  | LMNA | MAPK6 | ATP1B3 | AP000350.4 |
| ARIH2 |  | ATP6V0B | ADH5 |  | LYPLA2 | MORF4L2 | ATP5A1 | AP1S1 |
| ARL10 |  | ATP6V1A | ADPGK |  | MAZ | MTHFD2 | ATP6V1G1 | AP1S2 |
| ARL2BP |  | AZIN1 | AFF1 |  | MIB2 | MTRNR2L2 | AUP1 | AP2M1 |
| ARL6IP1 |  | B2M | AFF4 |  | NCSTN | MTRNR2L8 | B4GALT5 | AP2S1 |
| ARMC2 |  | BIRC5 | AGFG1 |  | NDUFA13 | MYL6 | BAG6 | AP3B1 |
| ARPC4-TTLL3 |  | BTF3L4 | AGPAT5 |  | PIP5K1C | NACA | BCL2L2-PABPN1 | AP3M1 |
| ARPC5 |  | BZW1 | AGPAT9 |  | PPEF2 | NAP1L1 | BSG | AP3S1 |
| ARSI |  | CAPZA1 | AHNAK |  | PRRX2 | NELFB | BTF3 | APH1A |
| ASB3 |  | CBFB | AK2 |  | PSMA4 | NKD2 | BUB3 | APMAP |
| ASPH |  | CBX3 | AK4 |  | PTMA | NME2 | C16orf72 | ARF4 |
| ASTN2 |  | CCND1 | AKAP11 |  | PTP4A1 | NPM1 | C1QBP | ARPC2 |
| ATG16L1 |  | CCNI | AKAP12 |  | PTPN6 | NPTX1 | CALM2 | ATF4 |
| ATP1B3 |  | CCT5 | AKAP2 |  | RCC1 | PAPOLA | CALR | ATP1B3 |
| ATP2A2 |  | CCT8 | AKIRIN1 |  | RCCD1 | PPARG | CANX | ATP5B |
| ATP5A1 |  | CD44 | AKT2 |  | RPL18 | PTP4A1 | CCDC50 | ATP5C1 |
| ATP5B |  | CD59 | ALDH9A1 |  | RPL18A | RABEP2 | CCNB1 | ATP5EP2 |
| ATP5E |  | CDC42 | ALKBH5 |  | RPL6 | RAN | CCT2 | ATP5G2 |
| ATP5L |  | CDK1 | ALS2CR8 |  | RPL7A | RBBP4 | CCT3 | ATP5G3 |
| ATP6V0E1 |  | CDK6 | AMOTL1 |  | RPL8 | RND3 | CCT4 | ATP5I |
| ATP7A |  | CELF1 | ANKFY1 |  | RPS11 | RP11-159D12.5 | CCT6A | ATP6V0E1 |
| ATPIF1 |  | CFL1 | ANKRD52 |  | SHANK3 | RPL10 | CCT7 | ATP6V1C2 |
| AVL9 |  | CGGBP1 | ANLN |  | SLC30A5 | RPL23A | CD63 | ATP6V1E1 |
| B4GALT1 |  | CHSY1 | ANP32A |  | SMCR7L | RPL27 | CDC42EP5 | ATP6V1F |
| BACH1 |  | CISD2 | AP1G1 |  | SOX10 | RPL36A | CDK2AP1 | ATP6V1G1 |
| BAG6 |  | CLASP2 | AP1M1 |  | SRSF1 | RPL37A | CHCHD2 | AUP1 |
| BCL2L14 |  | CLTA | API5 |  | TMEM33 | RPL4 | CLIC1 | B2M |
| BMI1 |  | CLTC | APLP2 |  | TPM4 | RPL5 | CLIC4 | B3GNT4 |
| BOLA2B |  | CMAS | APP |  | TUBA1B | RPL6 | CNGA4 | BAX |
| BRI3 |  | CMTM4 | APTX |  | TUBB | RPL7A | CNN3 | BCL2L2-PABPN1 |
| BRWD1 |  | CNBP | ARCN1 |  | UBB | RPN1 | CORO1C | BEST1 |
| BTF3L4 |  | CNOT6 | ARF1 |  | UBC | RPS11 | COX6C | BRI3 |
| BTRC |  | COPRS | ARF3 |  | USP7 | RPS18 | COX7A2 | BRK1 |
| BUB3 |  | COPS7A | ARF6 |  | VSX2 | RPS19 | CPM | BZW2 |
| BZW1 |  | COX4I1 | ARFGEF2 |  |  | RPS20 | CREBL2 | C11orf31 |
| C11orf65 |  | COX7B | ARHGAP12 |  |  | RPS3 | CRTAP | C14orf2 |
| C16orf52 |  | COX7C | ARID5B |  |  | RPS3A | CSDE1 | C17orf103 |
| C16orf72 |  | CSK | ARIH1 |  |  | RRAGC | CSNK1A1 | C18orf25 |
| C1orf228 |  | CTNNB1 | ARL1 |  |  | RYK | CSNK2A2 | C1orf43 |
| CALR |  | CYCS | ARL2BP |  |  | SLC25A1 | CTNNA1 | C1orf56 |
| CALU |  | DCBLD2 | ARL5A |  |  | SNRPD2 | DAZAP2 | C1QBP |
| CAND2 |  | DDX5 | ARL6IP1 |  |  | SPARC | DDOST | C2orf15 |
| CANX |  | DNAJA1 | ARL8B |  |  | SSH3 | DDX17 | C5orf45 |
| CARD8 |  | DYNLL2 | ARMCX6 |  |  | TARDBP | DDX3X | CALR |
| CBFB |  | EEF1A1 | ARPC4 |  |  | TCP1 | DNAJB1 | CAP1 |
| CBX3 |  | EEF1G | ARPC5 |  |  | TFDP1 | DSTN | CAPN2 |
| CCBL2 |  | EEF2 | ARPP19 |  |  | TFRC | EEF1B2 | CAPRIN1 |
| CCNB1IP1 |  | EI24 | ARRDC3 |  |  | TPI1 | EIF1AD | CAV1 |
| CCND1 |  | EIF1 | ASAP1 |  |  | TUBA1A | EIF2S2 | CCDC73 |
| CCT2 |  | EIF2S3 | ASH1L |  |  | TUBA1B | EIF3E | CCNB1 |
| CCT3 |  | EIF3M | ASPH |  |  | TUBB | EIF3G | CCNI |
| CCT8 |  | EIF4A2 | ASTN2 |  |  | TXNRD1 | EIF3I | CCT2 |
| CD164 |  | EIF4E2 | ATAD1 |  |  | USP33 | EIF3K | CCT3 |
| CD44 |  | EIF4H | ATF1 |  |  | WDR74 | EIF3L | CCT4 |
| CD46 |  | EIF5AL1 | ATF2 |  |  | XRCC6 | EIF4A1 | CCT6A |
| CD59 |  | ELAVL1 | ATF7IP |  |  | YWHAZ | EIF4B | CCT7 |
| CDC42SE2 |  | ERH | ATG7 |  |  | ZNF146 | EIF4G1 | CD151 |
| CDCA3 |  | FAM91A1 | ATL3 |  |  |  | EIF4G2 | CD63 |
| CDCA4 |  | FASN | ATM |  |  |  | EIF5A | CDK2 |
| CDH8 |  | FBXW11 | ATMIN |  |  |  | ENO1 | CDK4 |
| CDK1 |  | FKBP1A | ATOX1 |  |  |  | ENO2 | CEACAM8 |
| CDV3 |  | FNDC3B | ATP13A3 |  |  |  | ERO1L | CERS2 |
| CELF1 |  | FRMD6 | ATP1B1 |  |  |  | ETF1 | CLIC1 |
| CFTR |  | G3BP1 | ATP2A2 |  |  |  | FAU | CLIP4 |
| CHD3 |  | GLS | ATP5A1 |  |  |  | FERMT2 | CNN3 |
| CLASP1 |  | GNA13 | ATP5E |  |  |  | FGF13 | CNPY2 |
| CLIP4 |  | GNB2L1 | ATP5F1 |  |  |  | FTH1 | COLGALT1 |
| CLTC |  | GNPDA1 | ATP6AP1 |  |  |  | FTL | COPA |
| CMTM4 |  | GPI | ATP6AP2 |  |  |  | FUS | COPB1 |
| CNGB3 |  | H3F3A | ATP6V0B |  |  |  | GAPDH | COPB2 |
| CNPY2 |  | HDDC2 | ATP6V0D1 |  |  |  | GBE1 | COPZ1 |
| CNRIP1 |  | HEATR3 | ATP6V1A |  |  |  | GCOM1 | COX5B |
| COPB2 |  | HIF1A | ATP6V1B2 |  |  |  | GNAS | COX6A1 |
| CORO1C |  | HIST1H2AG | ATP6V1C1 |  |  |  | GNB1 | COX6B1 |
| COX7A2 |  | HIST1H2BC | ATP8B2 |  |  |  | GSTP1 | COX7A2 |
| COX7A2L |  | HIST2H2AA3 | ATP9A |  |  |  | H2AFV | COX8A |
| CPM |  | HIST2H3A | ATPIF1 |  |  |  | H2AFZ | CPM |
| CRTAP |  | HIST2H3C | ATRX |  |  |  | HDGF | CTNNA1 |
| CSDE1 |  | HNRNPA1 | ATXN7 |  |  |  | HDLBP | CTNNB1 |
| CSGALNACT1 |  | HNRNPA1L2 | ATXN7L3B |  |  |  | HIF1AN | CXorf57 |
| CSNK2A2 |  | HNRNPA2B1 | AURKB |  |  |  | HINT1 | DAD1 |
| CTBP2 |  | HSP90AA1 | AZIN1 |  |  |  | HIST1H1B | DAP3 |
| CTC-203F4.1 |  | HSPA4 | B4GALT1 |  |  |  | HIST1H1C | DDX1 |
| CTD-2510F5.6 |  | HSPA5 | B4GALT5 |  |  |  | HIST1H2AH | DDX21 |
| CTNNA1 |  | HSPA8 | B4GALT6 |  |  |  | HIST1H2AI | DDX24 |
| CTNNA3 |  | IMPAD1 | BACH1 |  |  |  | HIST1H2BO | DDX56 |
| CTSC |  | ITGA6 | BAG4 |  |  |  | HIST1H3B | DHX15 |
| CYB5D2 |  | ITGB8 | BAG5 |  |  |  | HIST1H3F | DKC1 |
| DAGLB |  | JUND | BAZ2A |  |  |  | HIST1H3H | DNAJB1 |
| DAZAP2 |  | KPNA2 | BCAP31 |  |  |  | HIST1H4C | DTYMK |
| DCBLD2 |  | LAPTM4B | BCLAF1 |  |  |  | HIST1H4D | DYNC1H1 |
| DDX3X |  | LARP1 | BIRC2 |  |  |  | HIST1H4E | DYNC1I2 |
| DHFR |  | LIMS1 | BIRC5 |  |  |  | HIST1H4H | DYNLL1 |
| DIDO1 |  | LONP2 | BLCAP |  |  |  | HIST1H4I | DYNLL2 |
| DLG2 |  | MAPK6 | BLMH |  |  |  | HIST1H4J | EEF1G |
| DMD |  | MAPKAPK2 | BLOC1S5-TXNDC5 |  |  |  | HIST1H4K | EEF2 |
| DNAJB11 |  | MATR3 | BLOC1S6 |  |  |  | HIST2H2AA4 | EIF1B |
| DNMT1 |  | MBNL1 | BMPR2 |  |  |  | HIST2H2AC | EIF3B |
| DOLPP1 |  | MDM2 | BNIP2 |  |  |  | HIST2H3D | EIF3D |
| DPP6 |  | MFSD1 | BNIP3 |  |  |  | HIST2H4A | EIF3G |
| DPYSL2 |  | MLEC | BRD4 |  |  |  | HIST2H4B | EIF3I |
| DTWD2 |  | MMADHC | BRI3BP |  |  |  | HMGA1 | EIF4A1 |
| E2F3 |  | MOB1A | BRWD1 |  |  |  | HMGB1 | EIF4A2 |
| ECH1 |  | MORF4L1 | BTBD1 |  |  |  | HNRNPC | EIF4A3 |
| EEF1E1 |  | MSN | BTBD7 |  |  |  | HNRNPH1 | EIF4G1 |
| EEF2 |  | MTHFD2 | BTF3 |  |  |  | HNRNPK | EIF5A |
| EFCAB11 |  | MTPN | BTF3L4 |  |  |  | HNRNPU | EIF6 |
| EFCAB14 |  | MYL6 | BTG1 |  |  |  | HNRPDL | ENO1 |
| EIF2B5 |  | NDUFC2 | BUB3 |  |  |  | HOXA4 | ENSA |
| EIF2S2 |  | NEK7 | BZW1 |  |  |  | HSP90AB1 | EPDR1 |
| EIF3E |  | NHP2L1 | C11orf58 |  |  |  | HSP90B1 | ERH |
| EIF4A2 |  | NONO | C12orf23 |  |  |  | HSPA9 | ERLEC1 |
| EIF4G2 |  | NQO1 | C14orf166 |  |  |  | HSPD1 | FAM120A |
| ELAVL1 |  | NR3C1 | C16orf52 |  |  |  | HSPE1 | FAM129B |
| ENO2 |  | NUCKS1 | C1orf216 |  |  |  | IFRG15 | FAM166A |
| ENSA |  | NUP62 | C20orf24 |  |  |  | ITGB1 | FAM83D |
| EPPIN |  | NUP93 | C21orf91 |  |  |  | KDELR2 | FAM96A |
| EPPIN-WFDC6 |  | OAZ1 | C4orf46 |  |  |  | KIF5B | FAU |
| ERCC1 |  | OSBPL8 | C5orf15 |  |  |  | KLF16 | FERMT2 |
| ERO1LB |  | PABPC1 | C5orf22 |  |  |  | KPNB1 | FHL2 |
| EYA4 |  | PABPC3 | C5orf24 |  |  |  | LAMP2 | FTH1 |
| EYS |  | PABPN1 | C5orf51 |  |  |  | LDHA | FTL |
| FAM122B |  | PAFAH1B2 | C6orf62 |  |  |  | LDHB | GABARAP |
| FAM160B1 |  | PARP1 | C7orf73 |  |  |  | LGALS1 | GABARAPL2 |
| FAM189A1 |  | PCBP1 | CADM1 |  |  |  | LMNA | GAPDH |
| FAM20B |  | PCBP2 | CALD1 |  |  |  | LSM14A | GCN1L1 |
| FAM219A |  | PDZD8 | CALM1 |  |  |  | LUZP6 | GCOM1 |
| FBLN2 |  | PEA15 | CALM2 |  |  |  | LYPLA2 | GGPS1 |
| FBRSL1 |  | PITHD1 | CALU |  |  |  | MAPRE1 | GLO1 |
| FBXO10 |  | POLR2M | CAMK2D |  |  |  | MAT2A | GLRX3 |
| FGF13 |  | POTEE | CAMSAP2 |  |  |  | MAZ | GNAS |
| FICD |  | PPP1CB | CAND1 |  |  |  | MDH1 | GNB2L1 |
| FKBP4 |  | PPP1CC | CANX |  |  |  | MEAF6 | GPR56 |
| FLJ27365 |  | PPP3CB | CAPZA1 |  |  |  | METTL24 | GPS2 |
| FNDC3B |  | PPP4R2 | CAPZA2 |  |  |  | MIF | GPX1 |
| FNTA |  | PRKAR1A | CARD8 |  |  |  | MORF4L2 | GSTP1 |
| FRK |  | PRKDC | CASC3 |  |  |  | MRPL19 | H2AFZ |
| FTH1 |  | PRPF8 | CASD1 |  |  |  | MRPL3 | H3F3B |
| FUT5 |  | PRSS23 | CASP2 |  |  |  | MTCH2 | HDAC1 |
| FXR1 |  | PSMA7 | CAV2 |  |  |  | MYL12B | HDGF |
| GATC |  | PSME3 | CBFB |  |  |  | MZT2B | HINT1 |
| GCOM1 |  | PTAR1 | CBL |  |  |  | NACA | HIST1H1B |
| GLB1 |  | PTMA | CBX1 |  |  |  | NAMPT | HIST1H1C |
| GLS |  | PTP4A1 | CBX3 |  |  |  | NAP1L1 | HIST1H2AB |
| GNB2L1 |  | PTP4A2 | CBX5 |  |  |  | NCL | HIST1H2AM |
| GNG2 |  | PTTG1 | CBX6 |  |  |  | NGFRAP1 | HIST1H2BC |
| GNPDA1 |  | RAB10 | CCDC50 |  |  |  | NME1 | HIST1H2BL |
| GOSR2 |  | RAB1A | CCDC90B |  |  |  | NME1-NME2 | HIST1H3B |
| GPATCH2L |  | RAC1 | CCND1 |  |  |  | NME2 | HIST1H3F |
| GPM6A |  | RAD21 | CCNT1 |  |  |  | NPM1 | HIST1H3J |
| GPR75-ASB3 |  | RAN | CCT5 |  |  |  | NUFIP2 | HIST1H4B |
| GYG2 |  | RAP2A | CCT8 |  |  |  | ODC1 | HIST1H4D |
| H2AFV |  | RAP2B | CD164 |  |  |  | ORAOV1 | HIST1H4H |
| HDDC2 |  | RBBP7 | CD2AP |  |  |  | P4HB | HIST1H4K |
| HELLS |  | RCN2 | CD44 |  |  |  | PAFAH1B1 | HIST2H2AA3 |
| HFM1 |  | RFK | CD46 |  |  |  | PAPOLA | HIST2H2AA4 |
| HHAT |  | RND3 | CD47 |  |  |  | PCNA | HIST2H2AB |
| HIBADH |  | RNF139 | CD59 |  |  |  | PDGFA | HIST2H4B |
| HIST1H2BC |  | RPL10 | CD9 |  |  |  | PDIA3 | HIST4H4 |
| HIST1H2BJ |  | RPL10A | CDC25B |  |  |  | PDIA6 | HMGA1 |
| HIST1H2BK |  | RPL13 | CDC27 |  |  |  | PFDN5 | HMGA2 |
| HN1 |  | RPL15 | CDC34 |  |  |  | PFN1 | HMGN3 |
| HN1L |  | RPL22 | CDC42 |  |  |  | PFN2 | HMGN4 |
| HNRNPA1 |  | RPL23 | CDC42SE1 |  |  |  | PGAM1 | HN1 |
| HNRNPC |  | RPL26 | CDC42SE2 |  |  |  | PGK1 | HNRNPA1 |
| HNRNPH1 |  | RPL27A | CDC73 |  |  |  | PHB | HNRNPA1L2 |
| HOOK3 |  | RPL31 | CDCA3 |  |  |  | PIGY | HNRNPAB |
| HOXA3 |  | RPL32 | CDCA4 |  |  |  | PKM | HNRNPM |
| HSBP1 |  | RPL36 | CDK1 |  |  |  | PLS3 | HPCAL1 |
| HSP90AA1 |  | RPL37 | CDK14 |  |  |  | PPIA | HSD17B12 |
| HSPA8 |  | RPL37A | CDK17 |  |  |  | PPIAL4G | HSP90AB1 |
| HSPD1 |  | RPL4 | CDK2AP1 |  |  |  | PPIB | HSP90B1 |
| IGF2R |  | RPL41 | CDK5R1 |  |  |  | PPP2R1A | HSPA8 |
| IL1RAP |  | RPL7 | CDK6 |  |  |  | PRDX1 | HSPA9 |
| IL4I1 |  | RPLP0 | CDKN1A |  |  |  | PRDX6 | HSPD1 |
| IMPAD1 |  | RPN1 | CDS2 |  |  |  | PSAP | IFI16 |
| IPO7 |  | RPS13 | CDV3 |  |  |  | PSMA4 | ILF2 |
| IQCG |  | RPS18 | CEBPB |  |  |  | PSMB1 | IMMT |
| JUP |  | RPS20 | CEBPG |  |  |  | PSMB3 | IMP3 |
| KAT8 |  | RPS23 | CELF1 |  |  |  | PSMB4 | IMP4 |
| KCNH8 |  | RPS27 | CELF2 |  |  |  | PSMD2 | IRAK1 |
| KCNK15 |  | RPS27A | CENPI |  |  |  | PSMD8 | KIF2C |
| KCNMA1 |  | RPS27L | CERS5 |  |  |  | RANBP1 | KPNA2 |
| KCTD2 |  | RPS3 | CERS6 |  |  |  | RBBP4 | KTN1 |
| KIAA1456 |  | RPS3A | CFL1 |  |  |  | RBX1 | LAMTOR5 |
| KPNA4 |  | RPS4X | CGGBP1 |  |  |  | RCC2 | LARS |
| KTN1 |  | RPS5 | CHCHD2 |  |  |  | RHEB | LDHA |
| LAMP2 |  | RPS6 | CHD9 |  |  |  | RNASEH1 | LDHB |
| LAMTOR1 |  | RRM2 | CHORDC1 |  |  |  | RP11-1035H13.3 | LGALS1 |
| LARP4 |  | SAP18 | CHP1 |  |  |  | RP1-164F3.9 | LIMA1 |
| LDHA |  | SBNO1 | CHSY1 |  |  |  | RP1-187N21.4 | LPP |
| LEPR |  | SEC23B | CHTOP |  |  |  | RPL11 | LYPLA2 |
| LONP2 |  | SEH1L | CISD2 |  |  |  | RPL12 | MAB21L3 |
| LPIN2 |  | SERF2 | CIT |  |  |  | RPL13A | MAGEA12 |
| LRRC59 |  | SERINC1 | CKAP4 |  |  |  | RPL14 | MAGEA3 |
| LRRTM3 |  | SGK196 | CKS1B |  |  |  | RPL17 | MAGEA4 |
| LRTOMT |  | SHISA5 | CKS2 |  |  |  | RPL17-C18orf32 | MAGEA6 |
| LSM14A |  | SIAH1 | CLASP2 |  |  |  | RPL18 | MANF |
| LZIC |  | SLC16A1 | CLIC4 |  |  |  | RPL18A | MAPRE1 |
| M6PR |  | SLC1A5 | CLINT1 |  |  |  | RPL19 | MARCKS |
| MAD1L1 |  | SLC20A1 | CLN6 |  |  |  | RPL21 | MARCKSL1 |
| MAP2K5 |  | SLC25A1 | CLNS1A |  |  |  | RPL23A | MAT2A |
| MAP3K13 |  | SMS | CLPTM1L |  |  |  | RPL24 | MAZ |
| MATR3 |  | SNRPB2 | CLTA |  |  |  | RPL27 | MCL1 |
| MAX |  | SNRPD2 | CLTC |  |  |  | RPL29 | MCM7 |
| MBNL1 |  | SPARC | CMAS |  |  |  | RPL3 | MDH1 |
| MDH1 |  | SPRED1 | CMIP |  |  |  | RPL30 | MFSD1 |
| MDM2 |  | SRSF3 | CMPK1 |  |  |  | RPL34 | MIA |
| MEAF6 |  | SSB | CMTM4 |  |  |  | RPL35 | MIF |
| MED28 |  | SSR3 | CMTM6 |  |  |  | RPL35A | MINOS1 |
| METTL21A |  | STARD7 | CMTM7 |  |  |  | RPL36A | MLLT6 |
| METTL5 |  | STK4 | CNBP |  |  |  | RPL38 | MORF4L2 |
| MLEC |  | SUB1 | CNIH |  |  |  | RPL5 | MRPL3 |
| MLPH |  | SUMO3 | CNOT6 |  |  |  | RPL6 | MRPL30 |
| MME |  | TARDBP | CNOT6L |  |  |  | RPL7A | MRPL51 |
| MOB4 |  | TCEB1 | COMMD10 |  |  |  | RPL8 | MRPS24 |
| MORF4L2 |  | TEAD1 | COPRS |  |  |  | RPL9 | MTCH2 |
| MPRIP |  | TFDP1 | COPS7A |  |  |  | RPLP1 | MYL12A |
| MRPL19 |  | TFRC | COPS8 |  |  |  | RPLP2 | MYL12B |
| MRPL3 |  | THOC3 | CORO1C |  |  |  | RPS10 | MYL6 |
| MRPS6 |  | TM4SF1 | COTL1 |  |  |  | RPS11 | MZT2B |
| MSTO1 |  | TMCO1 | COX4I1 |  |  |  | RPS12 | NACA |
| MTAP |  | TMED10 | COX7A2L |  |  |  | RPS14 | NACA2 |
| MTHFD2 |  | TMED2 | COX7B |  |  |  | RPS15 | NBPF16 |
| MTMR6 |  | TMEM167A | COX7C |  |  |  | RPS15A | NDNL2 |
| MUM1 |  | TMEM30A | CPNE3 |  |  |  | RPS17 | NDUFA1 |
| MYEOV2 |  | TMEM33 | CPNE8 |  |  |  | RPS17L | NDUFA13 |
| MYL6 |  | TMEM64 | CPOX |  |  |  | RPS19 | NDUFB2 |
| MYO1D |  | TMPO | CPSF6 |  |  |  | RPS2 | NDUFB3 |
| NAP1L1 |  | TMX1 | CREB3L2 |  |  |  | RPS24 | NDUFB9 |
| NAPEPLD |  | TNPO1 | CREB5 |  |  |  | RPS25 | NDUFS5 |
| NBL1 |  | TOP2A | CREBL2 |  |  |  | RPS26 | NGFRAP1 |
| NCAPD2 |  | TPI1 | CRIM1 |  |  |  | RPS28 | NME1 |
| NDUFA11 |  | TRIM58 | CRIP2 |  |  |  | RPS29 | NME1-NME2 |
| NDUFB2 |  | TSN | CRK |  |  |  | RPS7 | NME2 |
| NDUFB9 |  | TUBB | CRKL |  |  |  | RPS8 | NME7 |
| NDUFC2-KCTD14 |  | UBA2 | CRTAP |  |  |  | RPS9 | NOC2L |
| NEDD8-MDP1 |  | UBA52 | CSDE1 |  |  |  | RPSA | NOLC1 |
| NHSL2 |  | UBC | CSE1L |  |  |  | RPSAP58 | NOP56 |
| NKIRAS1 |  | UBE2E3 | CSNK1A1 |  |  |  | RTN4 | NOP58 |
| NMD3 |  | UBL5 | CSNK1E |  |  |  | RYK | NOTCH2NL |
| NME2 |  | UHMK1 | CSNK2A2 |  |  |  | S100A6 | NPC2 |
| NONO |  | UQCRFS1 | CTBP1 |  |  |  | SELT | NPM1 |
| NOTCH2 |  | USP34 | CTBP2 |  |  |  | SERBP1 | NPTN |
| NOTCH2NL |  | VDAC1 | CTDSP2 |  |  |  | SERPINE2 | NUDC |
| NPC2 |  | VHL | CTDSPL2 |  |  |  | SET | NUDT3 |
| NPM1 |  | VMA21 | CTGF |  |  |  | SFPQ | OAZ2 |
| NQO1 |  | WTAP | CTSB |  |  |  | SLC25A3 | ODC1 |
| NR3C1 |  | XPO1 | CTSC |  |  |  | SLC25A5 | OST4 |
| NREP |  | XRCC6 | CUL4B |  |  |  | SLC9B1 | OSTC |
| NUFIP2 |  | YWHAQ | CYCS |  |  |  | SLIRP | P2RY11 |
| ODF2L |  | ZC3H11A | DAZAP2 |  |  |  | SNRPB | P4HB |
| OR2W3 |  | ZFAND5 | DBI |  |  |  | SNX4 | PABPC1 |
| ORAOV1 |  | ZFAND6 | DCAF12 |  |  |  | SNX5 | PCNA |
| OTUD7A |  | ZFP91 | DCAF16 |  |  |  | SOD1 | PDAP1 |
| P4HB |  | ZFR | DCAF17 |  |  |  | SRP9 | PDCD5 |
| PABPC1 |  | ZNF460 | DCAF7 |  |  |  | SRSF1 | PDCD6 |
| PABPC1L |  |  | DCBLD2 |  |  |  | SRSF9 | PDIA6 |
| PAFAH1B2 |  |  | DCK |  |  |  | SSR1 | PFDN5 |
| PANK4 |  |  | DCP2 |  |  |  | SSR2 | PFN1 |
| PCYT1A |  |  | DCTN4 |  |  |  | STRAP | PGAM4 |
| PDCD6 |  |  | DCUN1D4 |  |  |  | STX16 | PIP4K2B |
| PDE3A |  |  | DDB1 |  |  |  | SUMO2 | PKM |
| PDS5A |  |  | DDX17 |  |  |  | SYNCRIP | PLEKHA2 |
| PEX1 |  |  | DDX3X |  |  |  | TBL1XR1 | PLEKHA5 |
| PEX11B |  |  | DDX3Y |  |  |  | TCP1 | POLR3K |
| PFDN5 |  |  | DDX5 |  |  |  | TM9SF3 | POTEE |
| PFN2 |  |  | DEK |  |  |  | TMBIM6 | PPIA |
| PGK1 |  |  | DENND6A |  |  |  | TMEM123 | PPIB |
| PHB |  |  | DENR |  |  |  | TMEM19 | PPP1R14B |
| PHB2 |  |  | DEPDC1 |  |  |  | TMEM48 | PPP1R2 |
| PHC1 |  |  | DESI2 |  |  |  | TMSB10 | PRDX1 |
| PHC3 |  |  | DGCR2 |  |  |  | TMTC4 | PRDX4 |
| PHKB |  |  | DGUOK |  |  |  | TNC | PRDX6 |
| PID1 |  |  | DHX36 |  |  |  | TPT1 | PRMT1 |
| PKM |  |  | DHX40 |  |  |  | TRA2B | PRMT5 |
| PLEKHA2 |  |  | DHX9 |  |  |  | TRAM1 | PRNP |
| PLEKHB2 |  |  | DIABLO |  |  |  | TRIM28 | PRR3 |
| PLXDC1 |  |  | DICER1 |  |  |  | TUBA1A | PSMA2 |
| PMEL |  |  | DIP2B |  |  |  | TUBA1B | PSMA3 |
| PMF1 |  |  | DLG1 |  |  |  | TUBA1C | PSMB1 |
| PMF1-BGLAP |  |  | DLX1 |  |  |  | TUBB4B | PSMB4 |
| POLDIP3 |  |  | DMXL1 |  |  |  | TXN | PSMB5 |
| POLR2F |  |  | DNAJA1 |  |  |  | TXNRD1 | PSMC1 |
| POLR2M |  |  | DNAJA2 |  |  |  | UBA1 | PSMD1 |
| POLR3G |  |  | DNAJB11 |  |  |  | UBB | PSMD13 |
| PPARG |  |  | DNAJC5 |  |  |  | UBE2C | PSMD2 |
| PPP1CC |  |  | DNER |  |  |  | UQCRH | PTBP1 |
| PPP1R8 |  |  | DPY19L1 |  |  |  | UQCRQ | PUM1 |
| PPP2CB |  |  | DPY19L3 |  |  |  | USP22 | PXN |
| PPP3CB |  |  | DPY19L4 |  |  |  | USP33 | RAB12 |
| PPP6R3 |  |  | DPYSL2 |  |  |  | UVSSA | RAB2A |
| PRDX1 |  |  | DR1 |  |  |  | VDAC2 | RAB5C |
| PRKAR1A |  |  | DSTN |  |  |  | VIM | RAB7A |
| PRR14L |  |  | DTX3L |  |  |  | VPS35 | RACGAP1 |
| PRSS23 |  |  | DUSP11 |  |  |  | WDR82 | RBBP7 |
| PSMA1 |  |  | DUSP5 |  |  |  | XPO4 | RBM15 |
| PSMB5 |  |  | DUSP6 |  |  |  | XRCC5 | RBM3 |
| PSMD8 |  |  | DUSP7 |  |  |  | YBX1 | RBM8A |
| PSME3 |  |  | DUT |  |  |  | YWHAB | RCC2 |
| PTGR1 |  |  | DYNC1LI2 |  |  |  | YWHAE | REXO1L1 |
| PVRL3 |  |  | DYRK2 |  |  |  | YWHAZ | REXO2 |
| QDPR |  |  | E2F3 |  |  |  |  | RHEB |
| QSER1 |  |  | EDARADD |  |  |  |  | RHOA |
| RAB1A |  |  | EEF1A1 |  |  |  |  | RNASEH1 |
| RAB5C |  |  | EEF1E1 |  |  |  |  | RNF139 |
| RABEP2 |  |  | EEF2K |  |  |  |  | ROMO1 |
| RACGAP1 |  |  | EFCAB14 |  |  |  |  | RP5-1165K10.1 |
| RAD23B |  |  | EFHD2 |  |  |  |  | RPAIN |
| RAPH1 |  |  | EFNA5 |  |  |  |  | RPL12 |
| RBBP4 |  |  | EFR3A |  |  |  |  | RPL13A |
| RBBP7 |  |  | EI24 |  |  |  |  | RPL14 |
| RBM15 |  |  | EID1 |  |  |  |  | RPL17 |
| RBMX |  |  | EIF1 |  |  |  |  | RPL17-C18orf32 |
| RDX |  |  | EIF1AD |  |  |  |  | RPL18 |
| RFX7 |  |  | EIF1AX |  |  |  |  | RPL18A |
| RNF14 |  |  | EIF2AK2 |  |  |  |  | RPL23A |
| RNF144A |  |  | EIF2S1 |  |  |  |  | RPL24 |
| RP11-1035H13.3 |  |  | EIF2S2 |  |  |  |  | RPL26 |
| RP11-159D12.5 |  |  | EIF2S3 |  |  |  |  | RPL27A |
| RP11-315D16.2 |  |  | EIF3A |  |  |  |  | RPL29 |
| RP11-318A15.7 |  |  | EIF3E |  |  |  |  | RPL3 |
| RP11-343C2.3 |  |  | EIF3F |  |  |  |  | RPL30 |
| RP11-618P17.4 |  |  | EIF3L |  |  |  |  | RPL35 |
| RP1-164F3.9 |  |  | EIF4B |  |  |  |  | RPL35A |
| RP11-664D7.4 |  |  | EIF4E |  |  |  |  | RPL36 |
| RP11-81K2.1 |  |  | EIF4E2 |  |  |  |  | RPL36AL |
| RP1-187N21.4 |  |  | EIF4EBP2 |  |  |  |  | RPL39 |
| RP13-512J5.1 |  |  | EIF4G2 |  |  |  |  | RPL9 |
| RPL10 |  |  | EIF4H |  |  |  |  | RPLP0 |
| RPL12 |  |  | EIF5 |  |  |  |  | RPS10 |
| RPL15 |  |  | EIF5AL1 |  |  |  |  | RPS11 |
| RPL17-C18orf32 |  |  | ELAVL1 |  |  |  |  | RPS14 |
| RPL22 |  |  | ELK3 |  |  |  |  | RPS15 |
| RPL23 |  |  | ELK4 |  |  |  |  | RPS17 |
| RPL27A |  |  | ELL2 |  |  |  |  | RPS17L |
| RPL30 |  |  | ELOF1 |  |  |  |  | RPS2 |
| RPL31 |  |  | ELOVL5 |  |  |  |  | RPS21 |
| RPL37 |  |  | EMC1 |  |  |  |  | RPS28 |
| RPL37A |  |  | ENAH |  |  |  |  | RPS29 |
| RPL4 |  |  | ENC1 |  |  |  |  | RPS3A |
| RPL9 |  |  | EOGT |  |  |  |  | RPS5 |
| RPLP0 |  |  | EPB41L3 |  |  |  |  | RPS8 |
| RPS2 |  |  | EPPIN |  |  |  |  | RPS9 |
| RPS20 |  |  | EPT1 |  |  |  |  | RPSA |
| RPS23 |  |  | ERGIC3 |  |  |  |  | RSL24D1 |
| RPS24 |  |  | ERO1L |  |  |  |  | S100A10 |
| RPS29 |  |  | ETF1 |  |  |  |  | S100A13 |
| RPS3 |  |  | ETFA |  |  |  |  | S100A6 |
| RPS3A |  |  | ETNK1 |  |  |  |  | SAMD11 |
| RPS5 |  |  | ETS1 |  |  |  |  | SCOC |
| RPS9 |  |  | ETV5 |  |  |  |  | SDHB |
| RUVBL1 |  |  | EVI5 |  |  |  |  | SEC61B |
| SAMSN1 |  |  | EZR |  |  |  |  | SF3A3 |
| SARNP |  |  | FABP5 |  |  |  |  | SF3B14 |
| SARS |  |  | FAM105B |  |  |  |  | SFT2D1 |
| SCD5 |  |  | FAM122B |  |  |  |  | SLA2 |
| SDK2 |  |  | FAM126A |  |  |  |  | SLC25A12 |
| SEC14L1 |  |  | FAM134A |  |  |  |  | SLC25A3 |
| SEH1L |  |  | FAM160B1 |  |  |  |  | SLC25A39 |
| SERF2 |  |  | FAM178A |  |  |  |  | SLC25A46 |
| SETD7 |  |  | FAM199X |  |  |  |  | SLC25A5 |
| SEZ6 |  |  | FAM219A |  |  |  |  | SLC30A5 |
| SFRP4 |  |  | FAM3C |  |  |  |  | SLC39A14 |
| SGOL1 |  |  | FAM49B |  |  |  |  | SLC4A5 |
| SHC4 |  |  | FAM60A |  |  |  |  | SLC6A6 |
| SHOC2 |  |  | FAM91A1 |  |  |  |  | SLIRP |
| SKP1 |  |  | FAR1 |  |  |  |  | SMARCC1 |
| SLC18A2 |  |  | FAT1 |  |  |  |  | SNRPD3 |
| SLC20A2 |  |  | FBXL17 |  |  |  |  | SNRPG |
| SLC25A3 |  |  | FBXO21 |  |  |  |  | SNX22 |
| SLC25A53 |  |  | FBXO5 |  |  |  |  | SOD1 |
| SLC26A2 |  |  | FBXW11 |  |  |  |  | SOX10 |
| SLC39A14 |  |  | FCF1 |  |  |  |  | SPATS2L |
| SLC39A9 |  |  | FEM1C |  |  |  |  | SPCS1 |
| SLC3A1 |  |  | FGF13 |  |  |  |  | SPG21 |
| SLC44A1 |  |  | FKBP1A |  |  |  |  | SRI |
| SLC51A |  |  | FKBP4 |  |  |  |  | SRP9 |
| SLC9B1 |  |  | FMNL2 |  |  |  |  | SRR |
| SLCO5A1 |  |  | FNBP1L |  |  |  |  | SRSF1 |
| SLIRP |  |  | FNBP4 |  |  |  |  | SRSF9 |
| SMEK2 |  |  | FNDC3B |  |  |  |  | STRAP |
| SMIM7 |  |  | FOXJ3 |  |  |  |  | SYNC |
| SMS |  |  | FOXK1 |  |  |  |  | TAF13 |
| SNAP23 |  |  | FOXK2 |  |  |  |  | TAF1L |
| SNRPD3 |  |  | FRMD6 |  |  |  |  | TAGLN2 |
| SOD2 |  |  | FRS2 |  |  |  |  | TCEA1 |
| SPAG17 |  |  | FUBP1 |  |  |  |  | TCF7L2 |
| SPARC |  |  | FUS |  |  |  |  | TCP1 |
| SPECC1 |  |  | FXR1 |  |  |  |  | TERF2IP |
| SPRY1 |  |  | FYTTD1 |  |  |  |  | TIMM17A |
| SQRDL |  |  | G2E3 |  |  |  |  | TMA7 |
| SSR1 |  |  | G3BP1 |  |  |  |  | TMEM109 |
| SSR2 |  |  | G3BP2 |  |  |  |  | TMEM212 |
| STMN1 |  |  | GBE1 |  |  |  |  | TMSB10 |
| SUMF1 |  |  | GDI1 |  |  |  |  | TMX2 |
| SUMO2 |  |  | GEMIN6 |  |  |  |  | TOMM5 |
| SUPT20H |  |  | GFM1 |  |  |  |  | TOMM6 |
| TAF1 |  |  | GFPT1 |  |  |  |  | TOP2B |
| TARDBP |  |  | GHITM |  |  |  |  | TPM1 |
| TCEB1 |  |  | GID8 |  |  |  |  | TPM4 |
| TCP1 |  |  | GINS1 |  |  |  |  | TPT1 |
| TEAD1 |  |  | GIT2 |  |  |  |  | TRA2A |
| TERF2IP |  |  | GLS |  |  |  |  | TRAM1 |
| TFRC |  |  | GLTP |  |  |  |  | TRIM28 |
| THOC3 |  |  | GMFB |  |  |  |  | TRIP6 |
| THUMPD1 |  |  | GNA13 |  |  |  |  | TSR1 |
| TICAM2 |  |  | GNAI3 |  |  |  |  | TUBA1A |
| TMED7-TICAM2 |  |  | GNAQ |  |  |  |  | TUBA1B |
| TMEM19 |  |  | GNB1 |  |  |  |  | TUBA1C |
| TMEM222 |  |  | GNB4 |  |  |  |  | TUBB3 |
| TMEM64 |  |  | GNG12 |  |  |  |  | TUBB4B |
| TMPO |  |  | GNG2 |  |  |  |  | TUFM |
| TMTC4 |  |  | GNPDA1 |  |  |  |  | TXN |
| TNC |  |  | GNPNAT1 |  |  |  |  | TXNDC17 |
| TNPO1 |  |  | GOLIM4 |  |  |  |  | TYMS |
| TNRC6A |  |  | GOLT1B |  |  |  |  | U2AF2 |
| TOP2A |  |  | GPCPD1 |  |  |  |  | UBB |
| TOR1AIP2 |  |  | GPD2 |  |  |  |  | UBE2A |
| TPGS2 |  |  | GPI |  |  |  |  | UBE2C |
| TRIM4 |  |  | GRSF1 |  |  |  |  | UBE2M |
| TROVE2 |  |  | GRWD1 |  |  |  |  | UBN2 |
| TRRAP |  |  | GSK3B |  |  |  |  | UQCR10 |
| TSHZ1 |  |  | GTF2A1 |  |  |  |  | UQCR11 |
| TTC3 |  |  | GTF2E2 |  |  |  |  | UQCRH |
| TTC39C |  |  | GTPBP3 |  |  |  |  | UQCRQ |
| TUBB3 |  |  | GULP1 |  |  |  |  | USMG5 |
| UBA2 |  |  | H2AFV |  |  |  |  | VAMP3 |
| UBA52 |  |  | H2AFY |  |  |  |  | VIM |
| UBAP2L |  |  | H3F3A |  |  |  |  | VPS26A |
| UBE2C |  |  | HAUS2 |  |  |  |  | VPS29 |
| UBE2W |  |  | HCFC1 |  |  |  |  | WNK1 |
| UGT8 |  |  | HDAC2 |  |  |  |  | XRCC2 |
| UHMK1 |  |  | HDDC2 |  |  |  |  | XRCC5 |
| UNC45B |  |  | HDLBP |  |  |  |  | XRCC6 |
| UNC5D |  |  | HEATR3 |  |  |  |  | YBX1 |
| USP13 |  |  | HELLS |  |  |  |  | YBX3 |
| USP33 |  |  | HERPUD1 |  |  |  |  | ZCCHC3 |
| UTP23 |  |  | HIATL1 |  |  |  |  | ZNF146 |
| WDFY4 |  |  | HIF1A |  |  |  |  | ZNF805 |
| WDR4 |  |  | HIF1AN |  |  |  |  |  |
| WEE1 |  |  | HIGD1A |  |  |  |  |  |
| XRCC6 |  |  | HIGD2A |  |  |  |  |  |
| YJEFN3 |  |  | HINT3 |  |  |  |  |  |
| YPEL1 |  |  | HIPK1 |  |  |  |  |  |
| YWHAE |  |  | HIPK2 |  |  |  |  |  |
| YWHAZ |  |  | HIST2H3C |  |  |  |  |  |
| ZC3H11A |  |  | HK2 |  |  |  |  |  |
| ZMIZ1 |  |  | HLA-B |  |  |  |  |  |
| ZNF114 |  |  | HLTF |  |  |  |  |  |
| ZNF280D |  |  | HMG20A |  |  |  |  |  |
| ZNF346 |  |  | HMGB1 |  |  |  |  |  |
| ZNF417 |  |  | HMGB2 |  |  |  |  |  |
| ZNF451 |  |  | HMGCR |  |  |  |  |  |
| ZNF483 |  |  | HMGN1 |  |  |  |  |  |
| ZNF565 |  |  | HMGN2 |  |  |  |  |  |
| ZNF587B |  |  | HMOX1 |  |  |  |  |  |
| ZNF706 |  |  | HN1L |  |  |  |  |  |
| ZNF772 |  |  | HNRNPA0 |  |  |  |  |  |
| ZNF793 |  |  | HNRNPA2B1 |  |  |  |  |  |
|  |  |  | HNRNPA3 |  |  |  |  |  |
|  |  |  | HNRNPC |  |  |  |  |  |
|  |  |  | HNRNPD |  |  |  |  |  |
|  |  |  | HNRNPF |  |  |  |  |  |
|  |  |  | HNRNPH1 |  |  |  |  |  |
|  |  |  | HNRNPH2 |  |  |  |  |  |
|  |  |  | HNRNPK |  |  |  |  |  |
|  |  |  | HNRNPL |  |  |  |  |  |
|  |  |  | HNRNPR |  |  |  |  |  |
|  |  |  | HNRNPU |  |  |  |  |  |
|  |  |  | HNRPDL |  |  |  |  |  |
|  |  |  | HOOK3 |  |  |  |  |  |
|  |  |  | HOXB7 |  |  |  |  |  |
|  |  |  | HP1BP3 |  |  |  |  |  |
|  |  |  | HPRT1 |  |  |  |  |  |
|  |  |  | HSBP1 |  |  |  |  |  |
|  |  |  | HSP90AA1 |  |  |  |  |  |
|  |  |  | HSPA13 |  |  |  |  |  |
|  |  |  | HSPA1A |  |  |  |  |  |
|  |  |  | HSPA1B |  |  |  |  |  |
|  |  |  | HSPE1 |  |  |  |  |  |
|  |  |  | HSPH1 |  |  |  |  |  |
|  |  |  | IDS |  |  |  |  |  |
|  |  |  | IER2 |  |  |  |  |  |
|  |  |  | IER3IP1 |  |  |  |  |  |
|  |  |  | IER5 |  |  |  |  |  |
|  |  |  | IL1RAP |  |  |  |  |  |
|  |  |  | IL6ST |  |  |  |  |  |
|  |  |  | ILF3 |  |  |  |  |  |
|  |  |  | IMPAD1 |  |  |  |  |  |
|  |  |  | INCENP |  |  |  |  |  |
|  |  |  | INHBA |  |  |  |  |  |
|  |  |  | INIP |  |  |  |  |  |
|  |  |  | INO80D |  |  |  |  |  |
|  |  |  | INPP5A |  |  |  |  |  |
|  |  |  | IPO7 |  |  |  |  |  |
|  |  |  | IQGAP1 |  |  |  |  |  |
|  |  |  | IRF2BP2 |  |  |  |  |  |
|  |  |  | IRF2BPL |  |  |  |  |  |
|  |  |  | ISCA1 |  |  |  |  |  |
|  |  |  | IST1 |  |  |  |  |  |
|  |  |  | ITGA6 |  |  |  |  |  |
|  |  |  | ITGAV |  |  |  |  |  |
|  |  |  | ITGB1 |  |  |  |  |  |
|  |  |  | ITGB8 |  |  |  |  |  |
|  |  |  | ITM2B |  |  |  |  |  |
|  |  |  | JKAMP |  |  |  |  |  |
|  |  |  | JUND |  |  |  |  |  |
|  |  |  | KBTBD2 |  |  |  |  |  |
|  |  |  | KCMF1 |  |  |  |  |  |
|  |  |  | KCNJ5 |  |  |  |  |  |
|  |  |  | KCTD10 |  |  |  |  |  |
|  |  |  | KCTD20 |  |  |  |  |  |
|  |  |  | KDELR2 |  |  |  |  |  |
|  |  |  | KDM5A |  |  |  |  |  |
|  |  |  | KDM6B |  |  |  |  |  |
|  |  |  | KHDRBS1 |  |  |  |  |  |
|  |  |  | KIAA0101 |  |  |  |  |  |
|  |  |  | KIAA0355 |  |  |  |  |  |
|  |  |  | KIAA1456 |  |  |  |  |  |
|  |  |  | KIAA1715 |  |  |  |  |  |
|  |  |  | KIF13A |  |  |  |  |  |
|  |  |  | KIF18B |  |  |  |  |  |
|  |  |  | KIF1C |  |  |  |  |  |
|  |  |  | KIF5B |  |  |  |  |  |
|  |  |  | KIFAP3 |  |  |  |  |  |
|  |  |  | KLHDC10 |  |  |  |  |  |
|  |  |  | KLHDC7A |  |  |  |  |  |
|  |  |  | KLHL8 |  |  |  |  |  |
|  |  |  | KLHL9 |  |  |  |  |  |
|  |  |  | KPNB1 |  |  |  |  |  |
|  |  |  | LAMP2 |  |  |  |  |  |
|  |  |  | LAMTOR1 |  |  |  |  |  |
|  |  |  | LAMTOR3 |  |  |  |  |  |
|  |  |  | LAPTM4A |  |  |  |  |  |
|  |  |  | LAPTM4B |  |  |  |  |  |
|  |  |  | LARP1 |  |  |  |  |  |
|  |  |  | LARP4 |  |  |  |  |  |
|  |  |  | LASP1 |  |  |  |  |  |
|  |  |  | LBR |  |  |  |  |  |
|  |  |  | LDLRAD3 |  |  |  |  |  |
|  |  |  | LDOC1L |  |  |  |  |  |
|  |  |  | LEF1 |  |  |  |  |  |
|  |  |  | LEPROT |  |  |  |  |  |
|  |  |  | LETM1 |  |  |  |  |  |
|  |  |  | LHFPL2 |  |  |  |  |  |
|  |  |  | LIMS1 |  |  |  |  |  |
|  |  |  | LIN52 |  |  |  |  |  |
|  |  |  | LIN7C |  |  |  |  |  |
|  |  |  | LMNB1 |  |  |  |  |  |
|  |  |  | LMO4 |  |  |  |  |  |
|  |  |  | LNPEP |  |  |  |  |  |
|  |  |  | LONP2 |  |  |  |  |  |
|  |  |  | LRPPRC |  |  |  |  |  |
|  |  |  | LRRC58 |  |  |  |  |  |
|  |  |  | LRRC59 |  |  |  |  |  |
|  |  |  | LRRC8A |  |  |  |  |  |
|  |  |  | LSM14A |  |  |  |  |  |
|  |  |  | LSM14B |  |  |  |  |  |
|  |  |  | LSM4 |  |  |  |  |  |
|  |  |  | LSM5 |  |  |  |  |  |
|  |  |  | LYPLA1 |  |  |  |  |  |
|  |  |  | LZIC |  |  |  |  |  |
|  |  |  | M6PR |  |  |  |  |  |
|  |  |  | MAFG |  |  |  |  |  |
|  |  |  | MAGEB10 |  |  |  |  |  |
|  |  |  | MAGT1 |  |  |  |  |  |
|  |  |  | MAP1LC3B |  |  |  |  |  |
|  |  |  | MAP3K13 |  |  |  |  |  |
|  |  |  | MAP4 |  |  |  |  |  |
|  |  |  | MAPK1 |  |  |  |  |  |
|  |  |  | MAPK14 |  |  |  |  |  |
|  |  |  | MAPK6 |  |  |  |  |  |
|  |  |  | MAPKAPK2 |  |  |  |  |  |
|  |  |  | MAT2B |  |  |  |  |  |
|  |  |  | MATR3 |  |  |  |  |  |
|  |  |  | MAX |  |  |  |  |  |
|  |  |  | MBNL1 |  |  |  |  |  |
|  |  |  | MCAM |  |  |  |  |  |
|  |  |  | MCM2 |  |  |  |  |  |
|  |  |  | MCM3 |  |  |  |  |  |
|  |  |  | MCMDC2 |  |  |  |  |  |
|  |  |  | MDH2 |  |  |  |  |  |
|  |  |  | MDM2 |  |  |  |  |  |
|  |  |  | MEAF6 |  |  |  |  |  |
|  |  |  | MECOM |  |  |  |  |  |
|  |  |  | MED28 |  |  |  |  |  |
|  |  |  | MED29 |  |  |  |  |  |
|  |  |  | MELK |  |  |  |  |  |
|  |  |  | MESDC2 |  |  |  |  |  |
|  |  |  | METAP2 |  |  |  |  |  |
|  |  |  | METTL21A |  |  |  |  |  |
|  |  |  | METTL5 |  |  |  |  |  |
|  |  |  | METTL9 |  |  |  |  |  |
|  |  |  | MGAT5 |  |  |  |  |  |
|  |  |  | MIB1 |  |  |  |  |  |
|  |  |  | MIDN |  |  |  |  |  |
|  |  |  | MKRN1 |  |  |  |  |  |
|  |  |  | MLEC |  |  |  |  |  |
|  |  |  | MMADHC |  |  |  |  |  |
|  |  |  | MMGT1 |  |  |  |  |  |
|  |  |  | MMP14 |  |  |  |  |  |
|  |  |  | MMS22L |  |  |  |  |  |
|  |  |  | MOB1A |  |  |  |  |  |
|  |  |  | MOB4 |  |  |  |  |  |
|  |  |  | MORF4L1 |  |  |  |  |  |
|  |  |  | MPRIP |  |  |  |  |  |
|  |  |  | MRFAP1 |  |  |  |  |  |
|  |  |  | MRFAP1L1 |  |  |  |  |  |
|  |  |  | MRPL19 |  |  |  |  |  |
|  |  |  | MRPL42 |  |  |  |  |  |
|  |  |  | MSI2 |  |  |  |  |  |
|  |  |  | MSL2 |  |  |  |  |  |
|  |  |  | MSN |  |  |  |  |  |
|  |  |  | MTAP |  |  |  |  |  |
|  |  |  | MTDH |  |  |  |  |  |
|  |  |  | MTHFD2 |  |  |  |  |  |
|  |  |  | MTMR4 |  |  |  |  |  |
|  |  |  | MTMR6 |  |  |  |  |  |
|  |  |  | MTMR9 |  |  |  |  |  |
|  |  |  | MTPN |  |  |  |  |  |
|  |  |  | MTRNR2L1 |  |  |  |  |  |
|  |  |  | MTRNR2L10 |  |  |  |  |  |
|  |  |  | MYLK |  |  |  |  |  |
|  |  |  | MYO1B |  |  |  |  |  |
|  |  |  | NAA20 |  |  |  |  |  |
|  |  |  | NAA50 |  |  |  |  |  |
|  |  |  | NAB1 |  |  |  |  |  |
|  |  |  | NACC1 |  |  |  |  |  |
|  |  |  | NACC2 |  |  |  |  |  |
|  |  |  | NAMPT |  |  |  |  |  |
|  |  |  | NAP1L1 |  |  |  |  |  |
|  |  |  | NAPB |  |  |  |  |  |
|  |  |  | NAPG |  |  |  |  |  |
|  |  |  | NBN |  |  |  |  |  |
|  |  |  | NCAPD2 |  |  |  |  |  |
|  |  |  | NCKAP1 |  |  |  |  |  |
|  |  |  | NCL |  |  |  |  |  |
|  |  |  | NCOA3 |  |  |  |  |  |
|  |  |  | NDRG3 |  |  |  |  |  |
|  |  |  | NDUFA4 |  |  |  |  |  |
|  |  |  | NDUFB4 |  |  |  |  |  |
|  |  |  | NDUFC2 |  |  |  |  |  |
|  |  |  | NDUFS2 |  |  |  |  |  |
|  |  |  | NECAP1 |  |  |  |  |  |
|  |  |  | NEDD8 |  |  |  |  |  |
|  |  |  | NEK4 |  |  |  |  |  |
|  |  |  | NEK7 |  |  |  |  |  |
|  |  |  | NF2 |  |  |  |  |  |
|  |  |  | NFAT5 |  |  |  |  |  |
|  |  |  | NFIB |  |  |  |  |  |
|  |  |  | NFYC |  |  |  |  |  |
|  |  |  | NGRN |  |  |  |  |  |
|  |  |  | NHP2L1 |  |  |  |  |  |
|  |  |  | NMD3 |  |  |  |  |  |
|  |  |  | NOL9 |  |  |  |  |  |
|  |  |  | NONO |  |  |  |  |  |
|  |  |  | NOTCH2 |  |  |  |  |  |
|  |  |  | NPAS2 |  |  |  |  |  |
|  |  |  | NQO1 |  |  |  |  |  |
|  |  |  | NR3C1 |  |  |  |  |  |
|  |  |  | NRAS |  |  |  |  |  |
|  |  |  | NREP |  |  |  |  |  |
|  |  |  | NSF |  |  |  |  |  |
|  |  |  | NT5DC3 |  |  |  |  |  |
|  |  |  | NUCKS1 |  |  |  |  |  |
|  |  |  | NUDCD3 |  |  |  |  |  |
|  |  |  | NUDT19 |  |  |  |  |  |
|  |  |  | NUDT21 |  |  |  |  |  |
|  |  |  | NUFIP2 |  |  |  |  |  |
|  |  |  | NUP153 |  |  |  |  |  |
|  |  |  | NUP62 |  |  |  |  |  |
|  |  |  | NUP93 |  |  |  |  |  |
|  |  |  | NUPL1 |  |  |  |  |  |
|  |  |  | NXPE3 |  |  |  |  |  |
|  |  |  | OAZ1 |  |  |  |  |  |
|  |  |  | ODF2L |  |  |  |  |  |
|  |  |  | OPA1 |  |  |  |  |  |
|  |  |  | OPHN1 |  |  |  |  |  |
|  |  |  | OSBP |  |  |  |  |  |
|  |  |  | OSBPL10 |  |  |  |  |  |
|  |  |  | OSBPL3 |  |  |  |  |  |
|  |  |  | OSBPL8 |  |  |  |  |  |
|  |  |  | OTUD4 |  |  |  |  |  |
|  |  |  | OTUD6B |  |  |  |  |  |
|  |  |  | PABPN1 |  |  |  |  |  |
|  |  |  | PACS2 |  |  |  |  |  |
|  |  |  | PAFAH1B1 |  |  |  |  |  |
|  |  |  | PAFAH1B2 |  |  |  |  |  |
|  |  |  | PAGR1 |  |  |  |  |  |
|  |  |  | PAK2 |  |  |  |  |  |
|  |  |  | PALM2-AKAP2 |  |  |  |  |  |
|  |  |  | PANK3 |  |  |  |  |  |
|  |  |  | PAPD5 |  |  |  |  |  |
|  |  |  | PAPOLA |  |  |  |  |  |
|  |  |  | PAPOLG |  |  |  |  |  |
|  |  |  | PARK7 |  |  |  |  |  |
|  |  |  | PARN |  |  |  |  |  |
|  |  |  | PARP1 |  |  |  |  |  |
|  |  |  | PCBP1 |  |  |  |  |  |
|  |  |  | PCBP2 |  |  |  |  |  |
|  |  |  | PCGF3 |  |  |  |  |  |
|  |  |  | PCNP |  |  |  |  |  |
|  |  |  | PCYT1A |  |  |  |  |  |
|  |  |  | PDCD10 |  |  |  |  |  |
|  |  |  | PDE12 |  |  |  |  |  |
|  |  |  | PDGFA |  |  |  |  |  |
|  |  |  | PDLIM5 |  |  |  |  |  |
|  |  |  | PDS5A |  |  |  |  |  |
|  |  |  | PDZD8 |  |  |  |  |  |
|  |  |  | PEA15 |  |  |  |  |  |
|  |  |  | PEBP1 |  |  |  |  |  |
|  |  |  | PEG10 |  |  |  |  |  |
|  |  |  | PERP |  |  |  |  |  |
|  |  |  | PEX11B |  |  |  |  |  |
|  |  |  | PEX26 |  |  |  |  |  |
|  |  |  | PFDN2 |  |  |  |  |  |
|  |  |  | PFKFB3 |  |  |  |  |  |
|  |  |  | PFN2 |  |  |  |  |  |
|  |  |  | PGAM1 |  |  |  |  |  |
|  |  |  | PGK1 |  |  |  |  |  |
|  |  |  | PGM2 |  |  |  |  |  |
|  |  |  | PGRMC1 |  |  |  |  |  |
|  |  |  | PHACTR4 |  |  |  |  |  |
|  |  |  | PHC1 |  |  |  |  |  |
|  |  |  | PHC3 |  |  |  |  |  |
|  |  |  | PHF6 |  |  |  |  |  |
|  |  |  | PHF8 |  |  |  |  |  |
|  |  |  | PHLDA1 |  |  |  |  |  |
|  |  |  | PHTF2 |  |  |  |  |  |
|  |  |  | PIGS |  |  |  |  |  |
|  |  |  | PIK3R1 |  |  |  |  |  |
|  |  |  | PIKFYVE |  |  |  |  |  |
|  |  |  | PITHD1 |  |  |  |  |  |
|  |  |  | PJA2 |  |  |  |  |  |
|  |  |  | PLEKHA3 |  |  |  |  |  |
|  |  |  | PLEKHB2 |  |  |  |  |  |
|  |  |  | PLS3 |  |  |  |  |  |
|  |  |  | PMEPA1 |  |  |  |  |  |
|  |  |  | PMP22 |  |  |  |  |  |
|  |  |  | PNN |  |  |  |  |  |
|  |  |  | PNPO |  |  |  |  |  |
|  |  |  | POLDIP3 |  |  |  |  |  |
|  |  |  | POLE3 |  |  |  |  |  |
|  |  |  | POLR2M |  |  |  |  |  |
|  |  |  | POMP |  |  |  |  |  |
|  |  |  | POU2F1 |  |  |  |  |  |
|  |  |  | PPAT |  |  |  |  |  |
|  |  |  | PPIC |  |  |  |  |  |
|  |  |  | PPIL4 |  |  |  |  |  |
|  |  |  | PPIP5K2 |  |  |  |  |  |
|  |  |  | PPM1A |  |  |  |  |  |
|  |  |  | PPP1CB |  |  |  |  |  |
|  |  |  | PPP1CC |  |  |  |  |  |
|  |  |  | PPP1R12A |  |  |  |  |  |
|  |  |  | PPP1R15B |  |  |  |  |  |
|  |  |  | PPP2CA |  |  |  |  |  |
|  |  |  | PPP2CB |  |  |  |  |  |
|  |  |  | PPP2R1A |  |  |  |  |  |
|  |  |  | PPP2R4 |  |  |  |  |  |
|  |  |  | PPP3CB |  |  |  |  |  |
|  |  |  | PPP3R1 |  |  |  |  |  |
|  |  |  | PPP4R2 |  |  |  |  |  |
|  |  |  | PPP6C |  |  |  |  |  |
|  |  |  | PPP6R3 |  |  |  |  |  |
|  |  |  | PRDX3 |  |  |  |  |  |
|  |  |  | PREPL |  |  |  |  |  |
|  |  |  | PRKAA1 |  |  |  |  |  |
|  |  |  | PRKAR1A |  |  |  |  |  |
|  |  |  | PRKD3 |  |  |  |  |  |
|  |  |  | PRKDC |  |  |  |  |  |
|  |  |  | PRPF4B |  |  |  |  |  |
|  |  |  | PRPF8 |  |  |  |  |  |
|  |  |  | PRPS1 |  |  |  |  |  |
|  |  |  | PRR14L |  |  |  |  |  |
|  |  |  | PRRC2B |  |  |  |  |  |
|  |  |  | PRRG4 |  |  |  |  |  |
|  |  |  | PRSS23 |  |  |  |  |  |
|  |  |  | PSAP |  |  |  |  |  |
|  |  |  | PSAT1 |  |  |  |  |  |
|  |  |  | PSD3 |  |  |  |  |  |
|  |  |  | PSMA4 |  |  |  |  |  |
|  |  |  | PSMA5 |  |  |  |  |  |
|  |  |  | PSMA6 |  |  |  |  |  |
|  |  |  | PSMA7 |  |  |  |  |  |
|  |  |  | PSMD10 |  |  |  |  |  |
|  |  |  | PSMD11 |  |  |  |  |  |
|  |  |  | PSMD12 |  |  |  |  |  |
|  |  |  | PSMD3 |  |  |  |  |  |
|  |  |  | PSME3 |  |  |  |  |  |
|  |  |  | PTAR1 |  |  |  |  |  |
|  |  |  | PTBP3 |  |  |  |  |  |
|  |  |  | PTCH2 |  |  |  |  |  |
|  |  |  | PTGES3 |  |  |  |  |  |
|  |  |  | PTMA |  |  |  |  |  |
|  |  |  | PTP4A1 |  |  |  |  |  |
|  |  |  | PTP4A2 |  |  |  |  |  |
|  |  |  | PTPN1 |  |  |  |  |  |
|  |  |  | PTPN11 |  |  |  |  |  |
|  |  |  | PTPN12 |  |  |  |  |  |
|  |  |  | PURA |  |  |  |  |  |
|  |  |  | PURB |  |  |  |  |  |
|  |  |  | PYGB |  |  |  |  |  |
|  |  |  | PYGO2 |  |  |  |  |  |
|  |  |  | PYURF |  |  |  |  |  |
|  |  |  | QDPR |  |  |  |  |  |
|  |  |  | QKI |  |  |  |  |  |
|  |  |  | QSER1 |  |  |  |  |  |
|  |  |  | RAB10 |  |  |  |  |  |
|  |  |  | RAB11A |  |  |  |  |  |
|  |  |  | RAB14 |  |  |  |  |  |
|  |  |  | RAB18 |  |  |  |  |  |
|  |  |  | RAB1A |  |  |  |  |  |
|  |  |  | RAB23 |  |  |  |  |  |
|  |  |  | RAB2B |  |  |  |  |  |
|  |  |  | RABL2B |  |  |  |  |  |
|  |  |  | RAC1 |  |  |  |  |  |
|  |  |  | RAD21 |  |  |  |  |  |
|  |  |  | RAD23B |  |  |  |  |  |
|  |  |  | RAN |  |  |  |  |  |
|  |  |  | RANBP1 |  |  |  |  |  |
|  |  |  | RANBP6 |  |  |  |  |  |
|  |  |  | RAP1B |  |  |  |  |  |
|  |  |  | RAP2A |  |  |  |  |  |
|  |  |  | RAP2B |  |  |  |  |  |
|  |  |  | RAPH1 |  |  |  |  |  |
|  |  |  | RASA2 |  |  |  |  |  |
|  |  |  | RASSF3 |  |  |  |  |  |
|  |  |  | RBBP4 |  |  |  |  |  |
|  |  |  | RBFOX2 |  |  |  |  |  |
|  |  |  | RBM22 |  |  |  |  |  |
|  |  |  | RBM25 |  |  |  |  |  |
|  |  |  | RBM27 |  |  |  |  |  |
|  |  |  | RBM39 |  |  |  |  |  |
|  |  |  | RBM5 |  |  |  |  |  |
|  |  |  | RBMS1 |  |  |  |  |  |
|  |  |  | RBMX |  |  |  |  |  |
|  |  |  | RBMXL1 |  |  |  |  |  |
|  |  |  | RBPJ |  |  |  |  |  |
|  |  |  | RBX1 |  |  |  |  |  |
|  |  |  | RC3H1 |  |  |  |  |  |
|  |  |  | RC3H2 |  |  |  |  |  |
|  |  |  | RCAN1 |  |  |  |  |  |
|  |  |  | RCCD1 |  |  |  |  |  |
|  |  |  | RCN1 |  |  |  |  |  |
|  |  |  | RCN2 |  |  |  |  |  |
|  |  |  | RCOR1 |  |  |  |  |  |
|  |  |  | RDX |  |  |  |  |  |
|  |  |  | REEP3 |  |  |  |  |  |
|  |  |  | REST |  |  |  |  |  |
|  |  |  | RFK |  |  |  |  |  |
|  |  |  | RFWD3 |  |  |  |  |  |
|  |  |  | RFX7 |  |  |  |  |  |
|  |  |  | RIF1 |  |  |  |  |  |
|  |  |  | RLIM |  |  |  |  |  |
|  |  |  | RMND5A |  |  |  |  |  |
|  |  |  | RND3 |  |  |  |  |  |
|  |  |  | RNF11 |  |  |  |  |  |
|  |  |  | RNF114 |  |  |  |  |  |
|  |  |  | RNF144A |  |  |  |  |  |
|  |  |  | RNF219 |  |  |  |  |  |
|  |  |  | RNF38 |  |  |  |  |  |
|  |  |  | RNPS1 |  |  |  |  |  |
|  |  |  | RP11-762I7.5 |  |  |  |  |  |
|  |  |  | RPL15 |  |  |  |  |  |
|  |  |  | RPL22 |  |  |  |  |  |
|  |  |  | RPL23 |  |  |  |  |  |
|  |  |  | RPL31 |  |  |  |  |  |
|  |  |  | RPL32 |  |  |  |  |  |
|  |  |  | RPL37 |  |  |  |  |  |
|  |  |  | RPL37A |  |  |  |  |  |
|  |  |  | RPL39L |  |  |  |  |  |
|  |  |  | RPL4 |  |  |  |  |  |
|  |  |  | RPL41 |  |  |  |  |  |
|  |  |  | RPL7 |  |  |  |  |  |
|  |  |  | RPN1 |  |  |  |  |  |
|  |  |  | RPN2 |  |  |  |  |  |
|  |  |  | RPP14 |  |  |  |  |  |
|  |  |  | RPRD1B |  |  |  |  |  |
|  |  |  | RPS13 |  |  |  |  |  |
|  |  |  | RPS15A |  |  |  |  |  |
|  |  |  | RPS20 |  |  |  |  |  |
|  |  |  | RPS23 |  |  |  |  |  |
|  |  |  | RPS24 |  |  |  |  |  |
|  |  |  | RPS27 |  |  |  |  |  |
|  |  |  | RPS27L |  |  |  |  |  |
|  |  |  | RPS3 |  |  |  |  |  |
|  |  |  | RPS4X |  |  |  |  |  |
|  |  |  | RPS6 |  |  |  |  |  |
|  |  |  | RPS6KA3 |  |  |  |  |  |
|  |  |  | RQCD1 |  |  |  |  |  |
|  |  |  | RRAGA |  |  |  |  |  |
|  |  |  | RRM2 |  |  |  |  |  |
|  |  |  | RRP15 |  |  |  |  |  |
|  |  |  | RSF1 |  |  |  |  |  |
|  |  |  | RTN4 |  |  |  |  |  |
|  |  |  | RUNX1 |  |  |  |  |  |
|  |  |  | RWDD1 |  |  |  |  |  |
|  |  |  | RYBP |  |  |  |  |  |
|  |  |  | RYK |  |  |  |  |  |
|  |  |  | SAE1 |  |  |  |  |  |
|  |  |  | SAMD1 |  |  |  |  |  |
|  |  |  | SAMD4A |  |  |  |  |  |
|  |  |  | SAP18 |  |  |  |  |  |
|  |  |  | SARNP |  |  |  |  |  |
|  |  |  | SART3 |  |  |  |  |  |
|  |  |  | SATB2 |  |  |  |  |  |
|  |  |  | SBNO1 |  |  |  |  |  |
|  |  |  | SCD |  |  |  |  |  |
|  |  |  | SCD5 |  |  |  |  |  |
|  |  |  | SDC4 |  |  |  |  |  |
|  |  |  | SDCBP |  |  |  |  |  |
|  |  |  | SDE2 |  |  |  |  |  |
|  |  |  | SEC23B |  |  |  |  |  |
|  |  |  | SEC24A |  |  |  |  |  |
|  |  |  | SEC61A1 |  |  |  |  |  |
|  |  |  | SEC61G |  |  |  |  |  |
|  |  |  | SEH1L |  |  |  |  |  |
|  |  |  | SEL1L |  |  |  |  |  |
|  |  |  | SELT |  |  |  |  |  |
|  |  |  | SEPW1 |  |  |  |  |  |
|  |  |  | SERBP1 |  |  |  |  |  |
|  |  |  | SERF2 |  |  |  |  |  |
|  |  |  | SERINC1 |  |  |  |  |  |
|  |  |  | SERP1 |  |  |  |  |  |
|  |  |  | SERPINE2 |  |  |  |  |  |
|  |  |  | SERTAD2 |  |  |  |  |  |
|  |  |  | SET |  |  |  |  |  |
|  |  |  | SETD7 |  |  |  |  |  |
|  |  |  | SF1 |  |  |  |  |  |
|  |  |  | SF3B1 |  |  |  |  |  |
|  |  |  | SFPQ |  |  |  |  |  |
|  |  |  | SFT2D2 |  |  |  |  |  |
|  |  |  | SGK1 |  |  |  |  |  |
|  |  |  | SGK196 |  |  |  |  |  |
|  |  |  | SGPL1 |  |  |  |  |  |
|  |  |  | SHISA5 |  |  |  |  |  |
|  |  |  | SHISA9 |  |  |  |  |  |
|  |  |  | SHOC2 |  |  |  |  |  |
|  |  |  | SHQ1 |  |  |  |  |  |
|  |  |  | SIAE |  |  |  |  |  |
|  |  |  | SIAH1 |  |  |  |  |  |
|  |  |  | SIAH2 |  |  |  |  |  |
|  |  |  | SKA2 |  |  |  |  |  |
|  |  |  | SKIL |  |  |  |  |  |
|  |  |  | SKP1 |  |  |  |  |  |
|  |  |  | SLC16A1 |  |  |  |  |  |
|  |  |  | SLC1A5 |  |  |  |  |  |
|  |  |  | SLC20A1 |  |  |  |  |  |
|  |  |  | SLC25A1 |  |  |  |  |  |
|  |  |  | SLC25A24 |  |  |  |  |  |
|  |  |  | SLC25A44 |  |  |  |  |  |
|  |  |  | SLC26A2 |  |  |  |  |  |
|  |  |  | SLC29A1 |  |  |  |  |  |
|  |  |  | SLC30A7 |  |  |  |  |  |
|  |  |  | SLC35B4 |  |  |  |  |  |
|  |  |  | SLC35E1 |  |  |  |  |  |
|  |  |  | SLC35F2 |  |  |  |  |  |
|  |  |  | SLC38A2 |  |  |  |  |  |
|  |  |  | SLC39A10 |  |  |  |  |  |
|  |  |  | SLC39A9 |  |  |  |  |  |
|  |  |  | SLC44A1 |  |  |  |  |  |
|  |  |  | SLC4A7 |  |  |  |  |  |
|  |  |  | SLC5A3 |  |  |  |  |  |
|  |  |  | SLC9A6 |  |  |  |  |  |
|  |  |  | SLMO2 |  |  |  |  |  |
|  |  |  | SMAD2 |  |  |  |  |  |
|  |  |  | SMAD5 |  |  |  |  |  |
|  |  |  | SMAP2 |  |  |  |  |  |
|  |  |  | SMARCAD1 |  |  |  |  |  |
|  |  |  | SMARCE1 |  |  |  |  |  |
|  |  |  | SMC1A |  |  |  |  |  |
|  |  |  | SMC5 |  |  |  |  |  |
|  |  |  | SMCR7L |  |  |  |  |  |
|  |  |  | SMG1 |  |  |  |  |  |
|  |  |  | SMG7 |  |  |  |  |  |
|  |  |  | SMIM13 |  |  |  |  |  |
|  |  |  | SMIM15 |  |  |  |  |  |
|  |  |  | SMIM7 |  |  |  |  |  |
|  |  |  | SMNDC1 |  |  |  |  |  |
|  |  |  | SMS |  |  |  |  |  |
|  |  |  | SNAP23 |  |  |  |  |  |
|  |  |  | SNRK |  |  |  |  |  |
|  |  |  | SNRPB |  |  |  |  |  |
|  |  |  | SNRPB2 |  |  |  |  |  |
|  |  |  | SNRPD1 |  |  |  |  |  |
|  |  |  | SNRPD2 |  |  |  |  |  |
|  |  |  | SNRPE |  |  |  |  |  |
|  |  |  | SNTB2 |  |  |  |  |  |
|  |  |  | SNX12 |  |  |  |  |  |
|  |  |  | SNX27 |  |  |  |  |  |
|  |  |  | SNX3 |  |  |  |  |  |
|  |  |  | SNX30 |  |  |  |  |  |
|  |  |  | SNX4 |  |  |  |  |  |
|  |  |  | SNX5 |  |  |  |  |  |
|  |  |  | SOCS5 |  |  |  |  |  |
|  |  |  | SOD2 |  |  |  |  |  |
|  |  |  | SOX13 |  |  |  |  |  |
|  |  |  | SOX4 |  |  |  |  |  |
|  |  |  | SP1 |  |  |  |  |  |
|  |  |  | SP3 |  |  |  |  |  |
|  |  |  | SPARC |  |  |  |  |  |
|  |  |  | SPCS3 |  |  |  |  |  |
|  |  |  | SPECC1L |  |  |  |  |  |
|  |  |  | SPIN1 |  |  |  |  |  |
|  |  |  | SPIN4 |  |  |  |  |  |
|  |  |  | SPOPL |  |  |  |  |  |
|  |  |  | SPRED1 |  |  |  |  |  |
|  |  |  | SPRYD7 |  |  |  |  |  |
|  |  |  | SPTSSA |  |  |  |  |  |
|  |  |  | SQSTM1 |  |  |  |  |  |
|  |  |  | SRD5A1 |  |  |  |  |  |
|  |  |  | SREK1 |  |  |  |  |  |
|  |  |  | SRGAP1 |  |  |  |  |  |
|  |  |  | SRP14 |  |  |  |  |  |
|  |  |  | SRP72 |  |  |  |  |  |
|  |  |  | SRPK1 |  |  |  |  |  |
|  |  |  | SRRM2 |  |  |  |  |  |
|  |  |  | SRSF2 |  |  |  |  |  |
|  |  |  | SRSF3 |  |  |  |  |  |
|  |  |  | SRSF6 |  |  |  |  |  |
|  |  |  | SRSF7 |  |  |  |  |  |
|  |  |  | SS18 |  |  |  |  |  |
|  |  |  | SSB |  |  |  |  |  |
|  |  |  | SSFA2 |  |  |  |  |  |
|  |  |  | SSH2 |  |  |  |  |  |
|  |  |  | SSR1 |  |  |  |  |  |
|  |  |  | SSR2 |  |  |  |  |  |
|  |  |  | SSR3 |  |  |  |  |  |
|  |  |  | ST13 |  |  |  |  |  |
|  |  |  | STAG2 |  |  |  |  |  |
|  |  |  | STARD4 |  |  |  |  |  |
|  |  |  | STARD7 |  |  |  |  |  |
|  |  |  | STAT3 |  |  |  |  |  |
|  |  |  | STAT5B |  |  |  |  |  |
|  |  |  | STAU1 |  |  |  |  |  |
|  |  |  | STK17A |  |  |  |  |  |
|  |  |  | STK4 |  |  |  |  |  |
|  |  |  | STMN1 |  |  |  |  |  |
|  |  |  | STX3 |  |  |  |  |  |
|  |  |  | STX6 |  |  |  |  |  |
|  |  |  | STYX |  |  |  |  |  |
|  |  |  | SUB1 |  |  |  |  |  |
|  |  |  | SUCO |  |  |  |  |  |
|  |  |  | SUDS3 |  |  |  |  |  |
|  |  |  | SUMO1 |  |  |  |  |  |
|  |  |  | SUMO2 |  |  |  |  |  |
|  |  |  | SUMO3 |  |  |  |  |  |
|  |  |  | SUPT16H |  |  |  |  |  |
|  |  |  | SUSD5 |  |  |  |  |  |
|  |  |  | SYAP1 |  |  |  |  |  |
|  |  |  | SYNCRIP |  |  |  |  |  |
|  |  |  | SYPL1 |  |  |  |  |  |
|  |  |  | TACC1 |  |  |  |  |  |
|  |  |  | TAF1 |  |  |  |  |  |
|  |  |  | TAF5 |  |  |  |  |  |
|  |  |  | TAF7 |  |  |  |  |  |
|  |  |  | TANC2 |  |  |  |  |  |
|  |  |  | TAOK1 |  |  |  |  |  |
|  |  |  | TARDBP |  |  |  |  |  |
|  |  |  | TAX1BP1 |  |  |  |  |  |
|  |  |  | TBC1D13 |  |  |  |  |  |
|  |  |  | TBCA |  |  |  |  |  |
|  |  |  | TBL1XR1 |  |  |  |  |  |
|  |  |  | TBPL1 |  |  |  |  |  |
|  |  |  | TCEAL8 |  |  |  |  |  |
|  |  |  | TEAD1 |  |  |  |  |  |
|  |  |  | TET3 |  |  |  |  |  |
|  |  |  | TFCP2 |  |  |  |  |  |
|  |  |  | TFDP1 |  |  |  |  |  |
|  |  |  | TFRC |  |  |  |  |  |
|  |  |  | TGFBR1 |  |  |  |  |  |
|  |  |  | TGFBR2 |  |  |  |  |  |
|  |  |  | TGOLN2 |  |  |  |  |  |
|  |  |  | THBS1 |  |  |  |  |  |
|  |  |  | THOC3 |  |  |  |  |  |
|  |  |  | THRAP3 |  |  |  |  |  |
|  |  |  | THUMPD1 |  |  |  |  |  |
|  |  |  | TIMP2 |  |  |  |  |  |
|  |  |  | TM4SF1 |  |  |  |  |  |
|  |  |  | TM9SF3 |  |  |  |  |  |
|  |  |  | TMBIM6 |  |  |  |  |  |
|  |  |  | TMCO1 |  |  |  |  |  |
|  |  |  | TMED10 |  |  |  |  |  |
|  |  |  | TMED2 |  |  |  |  |  |
|  |  |  | TMED7 |  |  |  |  |  |
|  |  |  | TMED8 |  |  |  |  |  |
|  |  |  | TMEM107 |  |  |  |  |  |
|  |  |  | TMEM123 |  |  |  |  |  |
|  |  |  | TMEM164 |  |  |  |  |  |
|  |  |  | TMEM167A |  |  |  |  |  |
|  |  |  | TMEM19 |  |  |  |  |  |
|  |  |  | TMEM2 |  |  |  |  |  |
|  |  |  | TMEM237 |  |  |  |  |  |
|  |  |  | TMEM248 |  |  |  |  |  |
|  |  |  | TMEM30A |  |  |  |  |  |
|  |  |  | TMEM33 |  |  |  |  |  |
|  |  |  | TMEM47 |  |  |  |  |  |
|  |  |  | TMEM48 |  |  |  |  |  |
|  |  |  | TMEM59 |  |  |  |  |  |
|  |  |  | TMEM64 |  |  |  |  |  |
|  |  |  | TMEM66 |  |  |  |  |  |
|  |  |  | TMEM9B |  |  |  |  |  |
|  |  |  | TMPO |  |  |  |  |  |
|  |  |  | TMX1 |  |  |  |  |  |
|  |  |  | TMX3 |  |  |  |  |  |
|  |  |  | TNC |  |  |  |  |  |
|  |  |  | TNFRSF21 |  |  |  |  |  |
|  |  |  | TNKS |  |  |  |  |  |
|  |  |  | TNKS2 |  |  |  |  |  |
|  |  |  | TNPO1 |  |  |  |  |  |
|  |  |  | TNPO2 |  |  |  |  |  |
|  |  |  | TNRC6A |  |  |  |  |  |
|  |  |  | TNRC6B |  |  |  |  |  |
|  |  |  | TOM1L2 |  |  |  |  |  |
|  |  |  | TOMM20 |  |  |  |  |  |
|  |  |  | TOMM22 |  |  |  |  |  |
|  |  |  | TOMM34 |  |  |  |  |  |
|  |  |  | TOMM7 |  |  |  |  |  |
|  |  |  | TOMM70A |  |  |  |  |  |
|  |  |  | TOP1 |  |  |  |  |  |
|  |  |  | TOP2A |  |  |  |  |  |
|  |  |  | TOR1AIP2 |  |  |  |  |  |
|  |  |  | TP53RK |  |  |  |  |  |
|  |  |  | TPGS2 |  |  |  |  |  |
|  |  |  | TPI1 |  |  |  |  |  |
|  |  |  | TPM3 |  |  |  |  |  |
|  |  |  | TRA2B |  |  |  |  |  |
|  |  |  | TRAPPC10 |  |  |  |  |  |
|  |  |  | TRIB1 |  |  |  |  |  |
|  |  |  | TRIM4 |  |  |  |  |  |
|  |  |  | TRIM58 |  |  |  |  |  |
|  |  |  | TRIP12 |  |  |  |  |  |
|  |  |  | TRMT112 |  |  |  |  |  |
|  |  |  | TROVE2 |  |  |  |  |  |
|  |  |  | TRPS1 |  |  |  |  |  |
|  |  |  | TSC22D1 |  |  |  |  |  |
|  |  |  | TSN |  |  |  |  |  |
|  |  |  | TSPAN3 |  |  |  |  |  |
|  |  |  | TSPAN31 |  |  |  |  |  |
|  |  |  | TTC3 |  |  |  |  |  |
|  |  |  | TUBB |  |  |  |  |  |
|  |  |  | TULP4 |  |  |  |  |  |
|  |  |  | TVP23B |  |  |  |  |  |
|  |  |  | TWF1 |  |  |  |  |  |
|  |  |  | TWISTNB |  |  |  |  |  |
|  |  |  | TWSG1 |  |  |  |  |  |
|  |  |  | TXLNA |  |  |  |  |  |
|  |  |  | TXNDC5 |  |  |  |  |  |
|  |  |  | TXNRD1 |  |  |  |  |  |
|  |  |  | U2SURP |  |  |  |  |  |
|  |  |  | UBA2 |  |  |  |  |  |
|  |  |  | UBA52 |  |  |  |  |  |
|  |  |  | UBAP2L |  |  |  |  |  |
|  |  |  | UBC |  |  |  |  |  |
|  |  |  | UBE2E1 |  |  |  |  |  |
|  |  |  | UBE2E3 |  |  |  |  |  |
|  |  |  | UBE2G1 |  |  |  |  |  |
|  |  |  | UBE2K |  |  |  |  |  |
|  |  |  | UBE2N |  |  |  |  |  |
|  |  |  | UBE2W |  |  |  |  |  |
|  |  |  | UBE2Z |  |  |  |  |  |
|  |  |  | UBE3A |  |  |  |  |  |
|  |  |  | UBL5 |  |  |  |  |  |
|  |  |  | UBLCP1 |  |  |  |  |  |
|  |  |  | UBQLN1 |  |  |  |  |  |
|  |  |  | UBXN7 |  |  |  |  |  |
|  |  |  | UGCG |  |  |  |  |  |
|  |  |  | UGDH |  |  |  |  |  |
|  |  |  | UGGT1 |  |  |  |  |  |
|  |  |  | UGT8 |  |  |  |  |  |
|  |  |  | UHMK1 |  |  |  |  |  |
|  |  |  | UPF3B |  |  |  |  |  |
|  |  |  | USP22 |  |  |  |  |  |
|  |  |  | USP34 |  |  |  |  |  |
|  |  |  | USP37 |  |  |  |  |  |
|  |  |  | UTP11L |  |  |  |  |  |
|  |  |  | VAPA |  |  |  |  |  |
|  |  |  | VAPB |  |  |  |  |  |
|  |  |  | VAT1 |  |  |  |  |  |
|  |  |  | VAV2 |  |  |  |  |  |
|  |  |  | VDAC1 |  |  |  |  |  |
|  |  |  | VDAC2 |  |  |  |  |  |
|  |  |  | VEZF1 |  |  |  |  |  |
|  |  |  | VGLL4 |  |  |  |  |  |
|  |  |  | VHL |  |  |  |  |  |
|  |  |  | VMA21 |  |  |  |  |  |
|  |  |  | VPS35 |  |  |  |  |  |
|  |  |  | VPS36 |  |  |  |  |  |
|  |  |  | WBP5 |  |  |  |  |  |
|  |  |  | WDR1 |  |  |  |  |  |
|  |  |  | WDR3 |  |  |  |  |  |
|  |  |  | WDR36 |  |  |  |  |  |
|  |  |  | WDR37 |  |  |  |  |  |
|  |  |  | WDR77 |  |  |  |  |  |
|  |  |  | WDR82 |  |  |  |  |  |
|  |  |  | WEE1 |  |  |  |  |  |
|  |  |  | WIPF2 |  |  |  |  |  |
|  |  |  | WSB2 |  |  |  |  |  |
|  |  |  | WTAP |  |  |  |  |  |
|  |  |  | WWTR1 |  |  |  |  |  |
|  |  |  | XIAP |  |  |  |  |  |
|  |  |  | XPO1 |  |  |  |  |  |
|  |  |  | XPO4 |  |  |  |  |  |
|  |  |  | XPOT |  |  |  |  |  |
|  |  |  | XRN1 |  |  |  |  |  |
|  |  |  | YAP1 |  |  |  |  |  |
|  |  |  | YOD1 |  |  |  |  |  |
|  |  |  | YTHDF2 |  |  |  |  |  |
|  |  |  | YWHAB |  |  |  |  |  |
|  |  |  | YWHAE |  |  |  |  |  |
|  |  |  | YWHAG |  |  |  |  |  |
|  |  |  | YWHAQ |  |  |  |  |  |
|  |  |  | YWHAZ |  |  |  |  |  |
|  |  |  | YY1 |  |  |  |  |  |
|  |  |  | ZBTB33 |  |  |  |  |  |
|  |  |  | ZC3H11A |  |  |  |  |  |
|  |  |  | ZC3H13 |  |  |  |  |  |
|  |  |  | ZC3H15 |  |  |  |  |  |
|  |  |  | ZFAND5 |  |  |  |  |  |
|  |  |  | ZFAND6 |  |  |  |  |  |
|  |  |  | ZFHX3 |  |  |  |  |  |
|  |  |  | ZFP36L1 |  |  |  |  |  |
|  |  |  | ZFP91 |  |  |  |  |  |
|  |  |  | ZFR |  |  |  |  |  |
|  |  |  | ZFX |  |  |  |  |  |
|  |  |  | ZMAT2 |  |  |  |  |  |
|  |  |  | ZMAT3 |  |  |  |  |  |
|  |  |  | ZMPSTE24 |  |  |  |  |  |
|  |  |  | ZNF346 |  |  |  |  |  |
|  |  |  | ZNF37A |  |  |  |  |  |
|  |  |  | ZNF451 |  |  |  |  |  |
|  |  |  | ZNF460 |  |  |  |  |  |
|  |  |  | ZNF471 |  |  |  |  |  |
|  |  |  | ZNF532 |  |  |  |  |  |
|  |  |  | ZNF611 |  |  |  |  |  |
|  |  |  | ZNF623 |  |  |  |  |  |
|  |  |  | ZNF652 |  |  |  |  |  |
|  |  |  | ZNF655 |  |  |  |  |  |
|  |  |  | ZNF664 |  |  |  |  |  |
|  |  |  | ZNF697 |  |  |  |  |  |
|  |  |  | ZNF703 |  |  |  |  |  |
|  |  |  | ZNF706 |  |  |  |  |  |
|  |  |  | ZNF770 |  |  |  |  |  |
|  |  |  | ZNF772 |  |  |  |  |  |
|  |  |  | ZNF793 |  |  |  |  |  |
|  |  |  | ZRANB2 |  |  |  |  |  |
|  |  |  | ZWINT |  |  |  |  |  |
