# Supplementary material for: Systems analysis identifies melanoma-enriched pro-oncogenic networks controlled by the RNA binding protein CELF1
Source: Nat Commun. 2017 Dec 21;8:2249. doi: 10.1038/s41467-017-02353-y (PMC5740069; doi:10.1038/s41467-017-02353-y)
Supplement: Supplementary file 7 — Supplementary Data 5 [file 41467_2017_2353_MOESM7_ESM.docx]

| **Deregulated at RNA Level upon CELF1 depletion (HJAY data) - *with* CUG repeats in 3'UTR** | |  | **Deregulated at RNA Level upon CELF1 depletion (HJAY data) - *without* CUG repeats in 3'UTR** | |
| --- | --- | --- | --- | --- |
| **Upregulated** | **Downregulated** |  | **Upregulated** | **Downregulated** |
| AGPAT9 | ACSL3 |  | ABHD12 | ACAT2 |
| APLP2 | AGFG1 |  | ACOT13 | AGBL5 |
| ATP13A3 | AHNAK |  | BAX | AMD1 |
| ATP6AP1 | AK4 |  | BRI3 | CCNB1 |
| B4GALT1 | ANLN |  | ERCC1 | CDK2 |
| B4GALT6 | AURKB |  | ERLEC1 | CNRIP1 |
| BIRC2 | BIRC5 |  | GLB1 | DHFR |
| BLCAP | C4orf46 |  | HIST1H4H | DMD |
| C5orf15 | CALD1 |  | HMGA2 | DNMT1 |
| CCDC90B | CCDC50 |  | HPCAL1 | ENO2 |
| CDKN1A | CDC25B |  | IGF2R | ERO1LB |
| CTSB | CDCA4 |  | MARCKSL1 | FAM20B |
| DCP2 | CDK1 |  | MFSD1 | FAM83D |
| DGCR2 | CELF1 |  | MRPS6 | FASN |
| DUSP5 | CENPI |  | NPC2 | GLO1 |
| DYNC1LI2 | CIT |  | SLC20A2 | HIST1H1B |
| EIF4E2 | CKAP4 |  | TPM1 | HIST1H2AB |
| ENAH | CKS1B |  | UBN2 | HIST1H2AH |
| ENC1 | CMTM6 |  | ZNF280D | HIST1H2AI |
| FAM3C | CNOT6L |  | ZNF805 | HIST1H2AM |
| FAT1 | CREB3L2 |  |  | HIST1H2BC |
| FEM1C | CRIM1 |  |  | HIST1H2BL |
| FNBP1L | CSE1L |  |  | HIST1H2BO |
| FNDC3B | CYCS |  |  | HIST1H3B |
| FRS2 | DCAF12 |  |  | HIST1H3F |
| GPCPD1 | DEK |  |  | HIST1H4B |
| IL1RAP | DEPDC1 |  |  | HIST1H4C |
| INHBA | DESI2 |  |  | HIST1H4D |
| KDM6B | DHX40 |  |  | HIST1H4E |
| KIF13A | DPYSL2 |  |  | HIST1H4I |
| KIFAP3 | DUT |  |  | HIST2H2AA4 |
| LHFPL2 | EFNA5 |  |  | KIF2C |
| MAP1LC3B | ELOVL5 |  |  | MCM7 |
| MAPK6 | ETV5 |  |  | MME |
| MDM2 | FBXO5 |  |  | NOLC1 |
| MMP14 | GINS1 |  |  | NUDT3 |
| NACC2 | GNPDA1 |  |  | PCNA |
| NCOA3 | GNPNAT1 |  |  | PHKB |
| PCGF3 | GPD2 |  |  | PPARG |
| PDGFA | H2AFV |  |  | PTTG1 |
| PEX11B | HELLS |  |  | RACGAP1 |
| PFKFB3 | HIF1AN |  |  | RCC1 |
| PIK3R1 | HK2 |  |  | SGOL1 |
| PLEKHA3 | HMGB2 |  |  | SLCO5A1 |
| PNN | HPRT1 |  |  | TYMS |
| PPIC | IRF2BPL |  |  | UBE2C |
| PRSS23 | KIF18B |  |  | USP13 |
| RAB18 | LBR |  |  | XRCC2 |
| RPS27L | LIN52 |  |  | ZNF114 |
| RRAGA | LMNB1 |  |  |  |
| SDC4 | LNPEP |  |  |  |
| SDCBP | LSM5 |  |  |  |
| SEPW1 | MAT2B |  |  |  |
| SERPINE2 | MCM2 |  |  |  |
| SLC20A1 | MCM3 |  |  |  |
| SOD2 | MELK |  |  |  |
| SQSTM1 | METAP2 |  |  |  |
| STAT3 | MMS22L |  |  |  |
| TIMP2 | MTHFD2 |  |  |  |
| TMEM59 | NAPG |  |  |  |
| TSC22D1 | NCAPD2 |  |  |  |
| UHMK1 | NFIB |  |  |  |
| VAT1 | NPAS2 |  |  |  |
| WEE1 | NRAS |  |  |  |
| YOD1 | PARP1 |  |  |  |
| ZNF652 | PDS5A |  |  |  |
|  | PFN2 |  |  |  |
|  | PGRMC1 |  |  |  |
|  | PPAT |  |  |  |
|  | PRKDC |  |  |  |
|  | PSAT1 |  |  |  |
|  | RANBP1 |  |  |  |
|  | RCAN1 |  |  |  |
|  | RCCD1 |  |  |  |
|  | RNPS1 |  |  |  |
|  | RPRD1B |  |  |  |
|  | RQCD1 |  |  |  |
|  | RRM2 |  |  |  |
|  | RWDD1 |  |  |  |
|  | SCD |  |  |  |
|  | SCD5 |  |  |  |
|  | SDE2 |  |  |  |
|  | SLC1A5 |  |  |  |
|  | SLC25A44 |  |  |  |
|  | SLC26A2 |  |  |  |
|  | SLC29A1 |  |  |  |
|  | SMC1A |  |  |  |
|  | SPIN4 |  |  |  |
|  | STARD4 |  |  |  |
|  | TMEM48 |  |  |  |
|  | TMEM9B |  |  |  |
|  | TMPO |  |  |  |
|  | TOM1L2 |  |  |  |
|  | TOP2A |  |  |  |
|  | TSN |  |  |  |
|  | VPS35 |  |  |  |
|  | WWTR1 |  |  |  |
|  | ZWINT |  |  |  |
