# Supplementary material for: Systems analysis identifies melanoma-enriched pro-oncogenic networks controlled by the RNA binding protein CELF1
Source: Nat Commun. 2017 Dec 21;8:2249. doi: 10.1038/s41467-017-02353-y (PMC5740069; doi:10.1038/s41467-017-02353-y)
Supplement: Supplementary file 8 — Supplementary Data 6 [file 41467_2017_2353_MOESM8_ESM.docx]

| **Gene** | **Alternative names** | **Function Summary** |
| --- | --- | --- |
| **AURKA** | AIK; ARK1; AURA; BTAK; STK6; STK7; STK15; PPP1R47 | A cell cycle-regulated kinase that involved in microtubule formation and/or stabilization at the spindle pole during chromosome segregation. Found at the centrosome in interphase cells and at the spindle poles in mitosis. |
| AURKB | AIK2; AIM1; ARK2; AurB; IPL1; STK5; AIM-1; STK12; PPP1R48; aurkb-sv1; aurkb-sv2 | Participates in the regulation of alignment and segregation of chromosomes during mitosis and meiosis through association with microtubules. |
| **BIRC5** | API4; EPR-1 | Member of the inhibitor of apoptosis gene family, which encode negative regulatory proteins that prevent apoptotic cell death. |
| **CCNB1** | CCNB | A regulatory protein involved in mitosis. Forms complexes with p34(cdc2) to form the maturation-promoting factor (MPF). Two alternative transcripts have been found, a constitutively expressed transcript and a cell cycle-regulated transcript, that is expressed predominantly during G2/M phase. |
| **CDC20** | CDC20A; p55CDC; bA276H19.3 | Acts as a regulatory protein interacting with several other proteins at multiple points in the cell cycle. It is required for two microtubule-dependent processes, nuclear movement prior to anaphase and chromosome separation. |
| **CDC6** | CDC18L; HsCDC6; MGORS5; HsCDC18 | A regulator at the early steps of DNA replication. Localizes in cell nucleus during cell cyle G1, but translocates to the cytoplasm at the start of S phase which is regulate by phsphorylation. |
| **CDK1** | CDC2; CDC28A; P34CDC2 | A member of the Ser/Thr protein kinase family. Catalytic subunit of M-phase promoting factor (MPF), which is essential for G1/S and G2/M phase transitions of eukaryotic cell cycle. The kinase activity of CDK1 is controlled by cyclin accumulation and destruction through the cell cycle. The phosphorylation and dephosphorylation of CDK1 also play important regulatory roles in cell cycle control. |
| CKS1B | CKS1; ckshs1; PNAS-16; PNAS-18 | Binds to the catalytic subunit of the cyclin dependent kinases and is essential for their biological function. |
| CLSPN | HClaspin | An essential upstream regulator of checkpoint kinase 1 and triggers a checkpoint arrest of the cell cycle in response to replicative stress or DNA damage. Also required for efficient DNA replication during a normal S phase. |
| **DHFR** | DYR; DHFRP1 | Converts dihydrofolate into tetrahydrofolate, a methyl group shuttle required for the de novo synthesis of purines, thymidylic acid, and certain amino acids. |
| **DNA2** | DNA2L; hDNA2 | A member of the DNA2/NAM7 helicase family. Involved in the maintenance of mitochondrial and nuclear DNA stability. |
| ESPL1 | ESP1; SEPA | Initiates the final separation of sister chromatids. |
| EXO1 | HEX1; hExoI | Has 5' to 3' exonuclease activity as well as an RNase H activity. Similar to the *S. cerevisiae* protein Exo1 which interacts with Msh2 and which is involved in mismatch repair and recombination. |
| **FANCA** | FA; FA1; FAA; FAH; FA-H; FACA; FANCH | Member of the Fanconi anemia complementation group A. Fanconi anemia is a genetically heterogeneous recessive disorder characterized by cytogenetic instability, hypersensitivity to DNA crosslinking agents, increased chromosomal breakage, and defective DNA repair. |
| **FANCD2** | FA4; FAD; FACD; FAD2; FA-D2; FANCD | Member of the Fanconi anemia complementation group D2. Fanconi anemia is a genetically heterogeneous recessive disorder characterized by cytogenetic instability, hypersensitivity to DNA crosslinking agents, increased chromosomal breakage, and defective DNA repair. Monoubiquinated in response to DNA damage, resulting in its localization to nuclear foci with BRCA1 and BRCA2 which are involved in homology-directed DNA repair. |
| FANCI | KIAA1794 | Member of the Fanconi anemia complementation group I. Fanconi anemia is a genetically heterogeneous recessive disorder characterized by cytogenetic instability, hypersensitivity to DNA crosslinking agents, increased chromosomal breakage, and defective DNA repair. |
| FEN1 | MF1; RAD2; FEN-1 | Removes 5' overhanging flaps in DNA repair and processes the 5' ends of Okazaki fragments in lagging strand DNA synthesis. |
| **FOXM1** | MPP2; HFH11; HNF-3; INS-1; MPP-2; PIG29; FKHL16; FOXM1B; HFH-11; TRIDENT; MPHOSPH2 | A transcriptional activator involved in cell proliferation. Phosphorylated in M phase and regulates the expression of several cell cycle genes, such as cyclin B1 and cyclin D1. |
| GINS2 | PSF2; Pfs2; HSPC037 | Subunit of GINS complex which is essential for the initiation of DNA replication. |
| GINS4 | SLD5 | Subunit of GINS complex which is essential for the initiation of DNA replication. |
| MCM2 | BM28; CCNL1; CDCL1; cdc19; DFNA70; D3S3194; MITOTIN | Subunit of the MCM2-7 protein complex involved in the initiation of eukaryotic genome replication. Forms a complex with MCM4, 6, and 7, and regulates the helicase activity of the complex. Regulated by, protein kinases CDC2 and CDC7 through phosphorylation. |
| MCM3 | HCC5; P1.h; RLFB; P1-MCM3 | Subunit of the MCM2-7 protein complex involved in the initiation of eukaryotic genome replication. Interacts directly with MCM5/CDC46. |
| **MCM4** | NKCD; CDC21; CDC54; NKGCD; hCdc21; P1-CDC21 | Subunit of the MCM2-7 protein complex involved in the initiation of eukaryotic genome replication. Phosphorylation of MCM4 by CDC2 kinase reduces the DNA helicase activity and chromatin binding of the MCM complex. |
| MCM5 | CDC46; MGORS8; P1-CDC46 | Subunit of the MCM2-7 protein complex involved in the initiation of eukaryotic genome replication. Very similar to the CDC46 protein from S. cerevisiae, a protein involved in the initiation of DNA replication. Upregulated in the transition from the G0 to G1/S phase of the cell cycle and may actively participate in cell cycle regulation. |
| **MCM6** | Mis5; P105MCM; MCG40308 | Subunit of the MCM2-7 protein complex involved in the initiation of eukaryotic genome replication. SNPs of MCM6 associated with differential transcriptional activation of the promoter of the neighboring lactase gene and, thereby, influence lactose intolerance in early adulthood. |
| MCM7 | MCM2; CDC47; P85MCM; P1CDC47; PNAS146; PPP1R104; P1.1-MCM3 | Subunit of the MCM2-7 protein complex involved in the initiation of eukaryotic genome replication. CDK4 associates with MCM7, and may regulate the binding of this protein with the tumorsuppressor protein RB1/RB. |
| **POLA1** | NSX; POLA; p180 | Catalytic subunit of DNA polymerase, which is part of the DNA polymerase alpha complex. The catalytic subunit plays an essential role in the initiation of DNA replication. |
| POLA2 | DNA polymerase alpha 70 kDa subunit | May play an essential role at the early stage of chromosomal DNA replication by coupling the polymerase alpha/primase complex to the cellular replication machinery. |
| PRKDC | HYRC; p350; DNAPK; DNPK1; HYRC1; IMD26; XRCC7; DNA-PKcs | Catalytic subunit of the DNA-dependent protein kinase. Functions with the Ku70/Ku80 heterodimer protein in DNA double strand break repair and recombination. |
| **RFC4** | A1; RFC37 | Subunit of RFC complex which consists of five distinct subunits (140, 40, 38, 37, and 36 kDa) and is required for DNA replication. . Forms a core complex with the 36 and 40 kDa subunits which possesses DNA-dependent ATPase activity. |
| **RFC5** | RFC36 | Smallest subunit of the replication factor C complex, which consists of five distinct subunits (140, 40, 38, 37, and 36 kDa) and is required for DNA replication. Interacts with the C-terminal region of proliferating cell nuclear antigen and is required to open and load proliferating cell nuclear antigen onto DNA during S phase. |
| RPA2 | REPA2; RPA32; RP-A p32; RP-A p34 | Part of the heterotrimeric replication protein A complex (RPA/RP-A). Binds and stabilizes single-stranded DNA intermediates, that form during DNA replication or upon DNA stress. Prevents their reannealing and in parallel, recruits and activates different proteins and complexes involved in DNA metabolism. |
| **RPA3** | REPA3; RP-A p14 | Part of the heterotrimeric replication protein A complex (RPA/RP-A). Binds and stabilizes single-stranded DNA intermediates, that form during DNA replication or upon DNA stress. Prevents their reannealing and in parallel, recruits and activates different proteins and complexes involved in DNA metabolism. |
| SOX10 | DOM; WS4; PCWH; WS2E; WS4C | Transcription factor involved in the regulation of embryonic development and in the determination of the cell fate. A nucleocytoplasmic shuttle protein and is important for neural crest and peripheral nervous system development. |
| TIMELESS | TIM; TIM1; hTIM | Involved in cell survival after damage or stress, increase in DNA polymerase epsilon activity, maintenance of telomere length, and epithelial cell morphogenesis. Plays a role in the circadian rhythm autoregulatory loop. |
| **TOP2A** | TOP2; TP2A | Enzyme that controls and alters the topologic states of DNA during transcription. Involved in chromosome condensation, chromatid separation, and the relief of torsional stress that occurs during DNA transcription and replication. Catalyzes the transient breaking and rejoining of two strands of duplex DNA which allows the strands to pass through one another, thus altering the topology of DNA. |
| **UBE2C** | UBCH10; dJ447F3.2 | A member of the E2 ubiquitin-conjugating enzyme family. Required for the destruction of mitotic cyclins and for cell cycle progression, and may be involved in cancer progression. |
