# Supplementary material for: Systems analysis identifies melanoma-enriched pro-oncogenic networks controlled by the RNA binding protein CELF1
Source: Nat Commun. 2017 Dec 21;8:2249. doi: 10.1038/s41467-017-02353-y (PMC5740069; doi:10.1038/s41467-017-02353-y)
Supplement: Supplementary file 9 — Supplementary Data 7 [file 41467_2017_2353_MOESM9_ESM.docx]

| **TECHNIQUE** | **REFERENCE** |
| --- | --- |
| ***Bioinformatic analyses for RNA-Seq and RIP-Seq*** | |
| Fastq | Cock PJ, et al. *Nucleic Acids Res* **38**, 1767-1771 (2010). |
| FastQC v0.11.0 | Andrews S. http://www.bioinformatics.babraham.ac.uk/projects/fastqc |
| TopHat-2.0.10 and Cufflinks 2.2.1 | Trapnell C, et al. Nat Protoc 7, 562-578 (2012). |
| Bowtie 1.0.0 | Langmead B, et al. Genome Biol 10, R25 (2009) |
| Samtools 0.1.1.9 | Li H, et al. Bioinformatics 25, 2078-2079 (2009). |
| Piranha 1.2.1 | Uren PJ, et al. Bioinformatics 28, 3013-3020 (2012). |
| PeakAnalyzer 1.4 | Salmon-Divon M, et al. BMC Bioinformatics 11, 415 (2010). |
| GRCh37.72 (Ensembl) | Chen Y, et al. BMC Genomics 11, 293 (2010). |
| BEDtools 2.16.2 | Quinlan AR. Curr Protoc Bioinformatics 47, 11 12 11-11 12 34 (2014). |
| Sequence Searcher | Marass F, Upton C. . BMC Res Notes 2, 14 (2009). |
| DREME | Bailey TL.Bioinformatics 27, 1653-1659 (2011). |
| ***Whole genome human junction arrays*** | |
| HJAY background correction, probe selection and Expression and alternative splicing analyses | Wang E, et al. PLoS One 7, e51266 (2012); Gandoura, S. et al. J Hepatol 58, 936-948 (2013). |
| FAST DB 2013_1 | de la Grange P, et al. Nucleic Acids Res 33, 4276-4284 (2005); de la Grange P, et al. BMC Bioinformatics 8, 180 (2007). |
| ***iTRAQ*** | |
| Protein fraction collection and methanol-chloroform precipitation and FASP sample digestion | Wisniewski JR, et al. Nat Methods 6, 359-362 (2009). |
| Solid phase extraction and salt removal, Isobar and GSEA | Ernoult E, et al. Proteome Sci 6, 27 (2008). |
| Mass spectra internal recalibration | Breitwieser FP, et al. J Proteome Res 10, 2758-2766 (2011). |
| ProteomeXchange Consortium | Vizcaino JA, et al. Nat Biotechnol 32, 223-226 (2014). |
| PRIDE | Vizcaino JA, et al. Nucleic Acids Res 41, D1063-1069 (2013). |
| ***GSEA, networks, heatmaps and Venn diagrams*** | |
| Cytoscape v3.2.1 | Shannon P, et al. Genome Res 13, 2498-2504 (2003). |
| ClueGO v2.1.7 | Bindea G, et al. Bioinformatics 25, 1091-1093 (2009). |
| Kolmogorov-Smirnoff correction for multiple testing | Subramanian A, et al. Proc Natl Acad Sci U S A 102, 15545-15550 (2005). |
| STRING | Jensen LJ, et al. Nucleic Acids Res 37, D412-416 (2009). |
| InteractiVenn | Heberle H, et al. BMC Bioinformatics 16, 169 (2015). |
| jvenn | Bardou P, et al. BMC Bioinformatics 15, 293 (2014). |
